# Supplementary figures and images for: Characterization of Enterobacter phage vB_EcRAM-01, a new Pseudotevenvirus against Enterobacter cloacae, isolated in an urban river in Panama
Source: PLoS One. 2024 Dec 31;19(12):e0310824. doi: 10.1371/journal.pone.0310824 (PMC11687723; doi:10.1371/journal.pone.0310824)

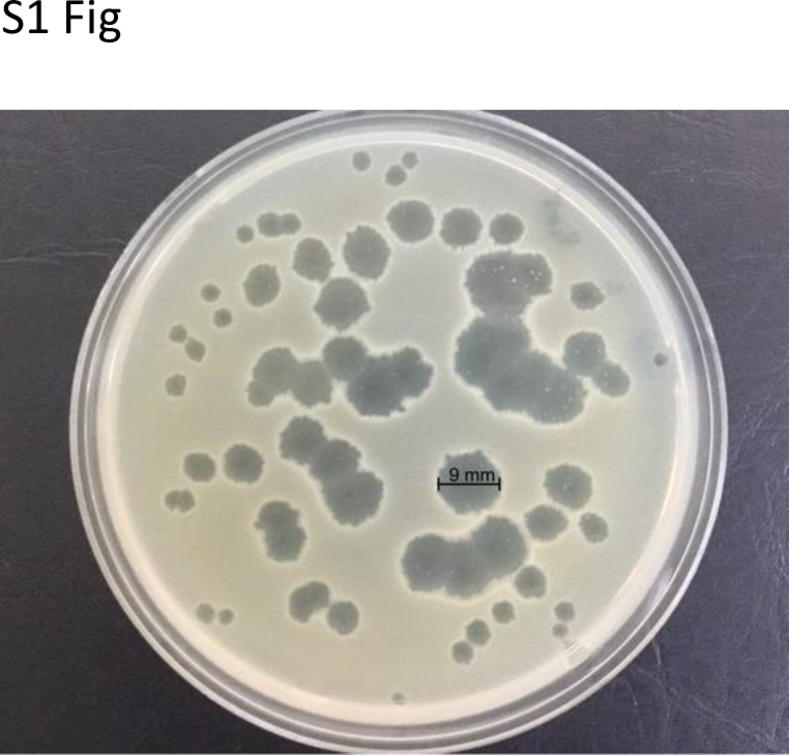

Supplement: S1 Fig — According to the double agar plate method, the bacterial lawn of the E. cloacae complex is infected by bacteriophages. After purification, lysis PFU measuring approximately 9 mm (shown in the figure), rough-edged and completely clear, were obtained. (TIF) [file pone.0310824.s001.tif]

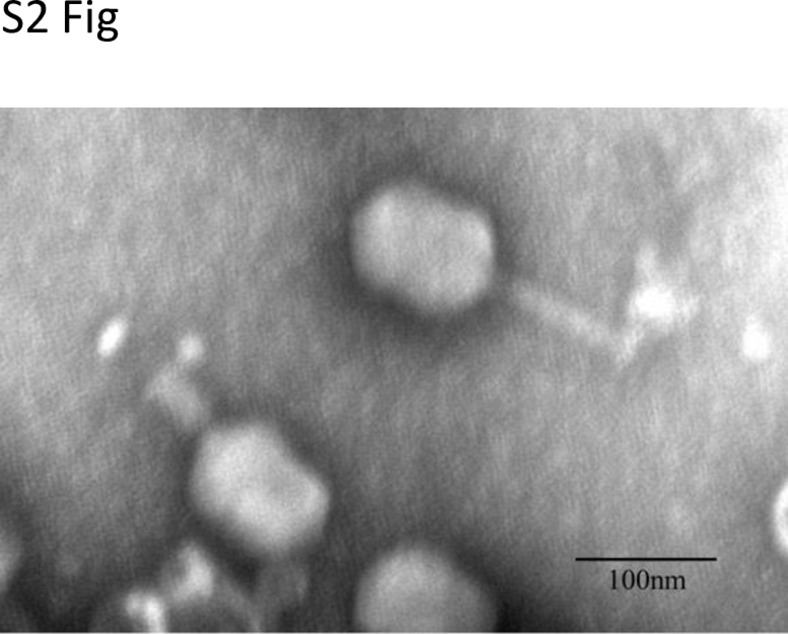

Supplement: S2 Fig — A representative micrograph of a purified sample of the bacteriophage is shown (scale bar: 100nm). (TIF) [file pone.0310824.s002.tif]

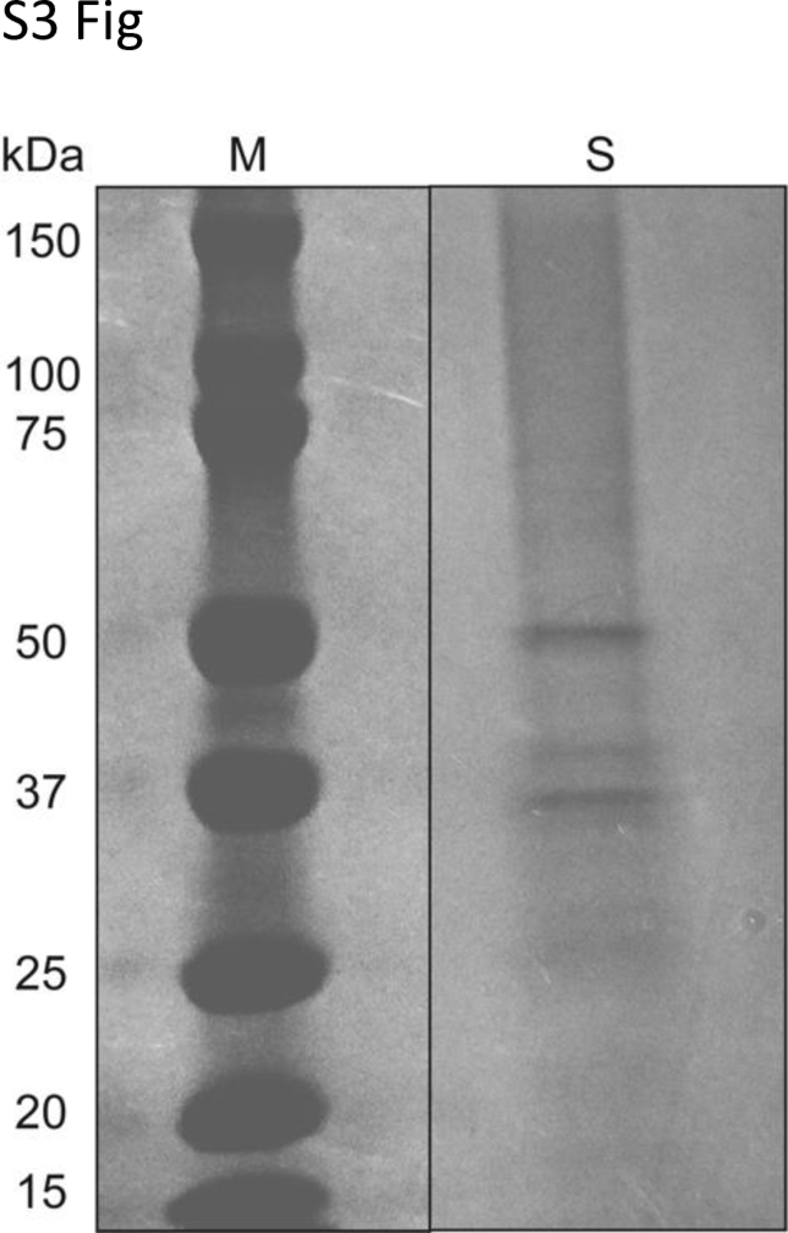

Supplement: S3 Fig — M: Precision Plus protein ladder., S: Phage sample. Ladder molecular weights are indicated on the left. (TIF) [file pone.0310824.s003.tif]
