# Supplementary material for: Characterization of Enterobacter phage vB_EcRAM-01, a new Pseudotevenvirus against Enterobacter cloacae, isolated in an urban river in Panama
Source: PLoS One. 2024 Dec 31;19(12):e0310824. doi: 10.1371/journal.pone.0310824 (PMC11687723; doi:10.1371/journal.pone.0310824)
Supplement: S1 Table — (DOCX) [file pone.0310824.s004.docx]

S1 Table Annotation of Enterobacter phage vB_EcRAM-01 genes

| **Name** | **Type** | **Minimum** | **Maximum** | **Length** | **Direction** | **Min (with gaps)** | **Max (with gaps)** | **Length (with gaps)** | **product** | **protein_id** | **E-Value** | **Identity** |
| --- | --- | --- | --- | --- | --- | --- | --- | --- | --- | --- | --- | --- |
| hypothetical protein CDS | CDS | 177,478 | 177,768 | 291 | reverse | 178,25 | 178,54 | 291 | hypothetical protein | UJB55401.1 | 4,00E-46 | 79.12% |
| hypothetical protein CDS | CDS | 177,188 | 177,463 | 276 | reverse | 177,96 | 178,235 | 276 | hypothetical protein | UJB55400.1 | 8,00E-54 | 90.11% |
| holin CDS | CDS | 176,564 | 177,205 | 642 | forward | 177,336 | 177,977 | 642 | holin | UJB55399.1 | 1,00E-152 | 98.59% |
| receptor recognizing protein CDS | CDS | 175,699 | 176,511 | 813 | forward | 176,471 | 177,283 | 813 | receptor recognizing protein | UJB55398.1 | 0.0 | 98.89% |
| hypothetical protein CDS | CDS | 173,46 | 175,673 | 2,214 | forward | 174,232 | 176,445 | 2,214 | hypothetical protein | UJB55397.1 | 0.0 | 98.91% |
| hypothetical protein CDS | CDS | 171,193 | 173,397 | 2,205 | forward | 171,965 | 174,169 | 2,205 | hypothetical protein | UJB55396.1 | 0.0 | 87.13% |
| hypothetical protein CDS | CDS | 169,546 | 171,141 | 1,596 | forward | 170,318 | 171,913 | 1,596 | hypothetical protein | UJB55395.1 | 0.0 | 99.06% |
| long tail fiber proximal subunit CDS | CDS | 165,881 | 169,54 | 3,66 | forward | 166,653 | 170,312 | 3,66 | long tail fiber proximal subunit | UJB55394.1 | 0.0 | 99.10% |
| hypothetical protein CDS | CDS | 164,397 | 165,824 | 1,428 | forward | 165,169 | 166,596 | 1,428 | hypothetical protein | UJB55393.1 | 0.0 | 99.37% |
| hypothetical protein CDS | CDS | 164,062 | 164,4 | 339 | forward | 164,834 | 165,172 | 339 | hypothetical protein | UJB55392.1 | 1,00E-73 | 99.11% |
| glutaredoxin CDS | CDS | 163,783 | 164,061 | 279 | forward | 164,555 | 164,833 | 279 | glutaredoxin | UJB55391.1 | 6,00E-60 | 98.91% |
| hypothetical protein CDS | CDS | 163,089 | 163,823 | 735 | forward | 163,861 | 164,595 | 735 | hypothetical protein | UJB55390.1 | 3,00E-178 | 99.59% |
| hypothetical protein CDS | CDS | 162,04 | 163,041 | 1,002 | forward | 162,812 | 163,813 | 1,002 | hypothetical protein | UJB55389.1 | 0.0 | 97.80% |
| hypothetical protein CDS | CDS | 161,919 | 162,08 | 162 | forward | 162,691 | 162,852 | 162 | hypothetical protein | UJB55388.1 | 4,00E-28 | 98.11% |
| ribonucleotide reductase of class III (anaerobic) CDS | CDS | 161,371 | 161,862 | 492 | forward | 162,143 | 162,634 | 492 | ribonucleotide reductase of class III (anaerobic) | UJB55387.1 | 2,00E-117 | 99.39% |
| hypothetical protein CDS | CDS | 160,841 | 161,371 | 531 | forward | 161,613 | 162,143 | 531 | hypothetical protein | UJB55386.1 | 5,00E-121 | 97.73% |
| hypothetical protein CDS | CDS | 160,65 | 160,844 | 195 | forward | 161,422 | 161,616 | 195 | hypothetical protein | UJB55385.1 | 1,00E-37 | 98.44% |
| hypothetical protein CDS | CDS | 160,32 | 160,604 | 285 | forward | 161,092 | 161,376 | 285 | hypothetical protein | UJB55384.1 | 6,00E-63 | 98.94% |
| hypothetical protein CDS | CDS | 159,379 | 160,326 | 948 | forward | 160,151 | 161,098 | 948 | hypothetical protein | UJB55383.1 | 0.0 | 99.05% |
| hypothetical protein CDS | CDS | 158,753 | 159,349 | 597 | forward | 159,525 | 160,121 | 597 | hypothetical protein | UJB55382.1 | 6,00E-134 | 97.98% |
| hypothetical protein CDS | CDS | 158,243 | 158,743 | 501 | forward | 159,015 | 159,515 | 501 | hypothetical protein | UJB55381.1 | 2,00E-113 | 96.97% |
| hypothetical protein CDS | CDS | 158,004 | 158,246 | 243 | forward | 158,776 | 159,018 | 243 | hypothetical protein | UJB55380.1 | 7,00E-52 | 97.50% |
| hypothetical protein CDS | CDS | 157,489 | 158,007 | 519 | forward | 158,261 | 158,779 | 519 | hypothetical protein | UJB55379.1 | 2,00E-113 | 92.44% |
| anaerobic ribonucleoside-triphosphate reductase CDS | CDS | 155,295 | 157,418 | 2,124 | forward | 156,067 | 158,19 | 2,124 | anaerobic ribonucleoside-triphosphate reductase | UJB55378.1 | 0.0 | 96.46% |
| recombination endonuclease VII CDS | CDS | 154,816 | 155,298 | 483 | forward | 155,588 | 156,07 | 483 | recombination endonuclease VII | UJB55377.1 | 2,00E-115 | 99.38% |
| thioredoxin CDS | CDS | 154,514 | 154,783 | 270 | forward | 155,286 | 155,555 | 270 | thioredoxin | UJB55376.1 | 6,00E-59 | 98.88% |
| DNA adenine methylase CDS | CDS | 153,656 | 154,507 | 852 | forward | 154,428 | 155,279 | 852 | DNA adenine methylase | UJB55375.1 | 0.0 | 99.65% |
| hypothetical protein CDS | CDS | 153,351 | 153,659 | 309 | forward | 154,123 | 154,431 | 309 | hypothetical protein | UJB55374.1 | 2,00E-67 | 99.02% |
| putative nicotinate phosphoribosyltransferase CDS | CDS | 151,937 | 153,37 | 1,434 | forward | 152,709 | 154,142 | 1,434 | putative nicotinate phosphoribosyltransferase | UJB55373.1 | 0.0 | 99.79% |
| hypothetical protein CDS | CDS | 151,449 | 151,913 | 465 | forward | 152,221 | 152,685 | 465 | hypothetical protein | UJB55372.1 | 8,00E-110 | 99.35% |
| hypothetical protein CDS | CDS | 151,128 | 151,439 | 312 | forward | 151,9 | 152,211 | 312 | hypothetical protein | UJB55371.1 | 3,00E-59 | 100.00% |
| hypothetical protein CDS | CDS | 150,626 | 151,084 | 459 | forward | 151,398 | 151,856 | 459 | hypothetical protein | UJB55370.1 | 5,00E-96 | 90.79% |
| hypothetical protein CDS | CDS | 150,335 | 150,556 | 222 | forward | 151,107 | 151,328 | 222 | hypothetical protein | UJB55369.1 | 1,00E-45 | 100.00% |
| hypothetical protein CDS | CDS | 149,975 | 150,334 | 360 | forward | 150,747 | 151,106 | 360 | hypothetical protein | UJB55368.1 | 7,00E-81 | 99.16% |
| RNA ligase 2 CDS | CDS | 148,939 | 149,985 | 1,047 | forward | 149,711 | 150,757 | 1,047 | RNA ligase 2 | UJB55367.1 | 0.0 | 98.56% |
| postulated decoy of host sigma70 or sigmaS CDS | CDS | 148,403 | 148,936 | 534 | forward | 149,175 | 149,708 | 534 | postulated decoy of host sigma70 or sigmaS | UJB55366.1 | 2,00E-124 | 98.31% |
| hypothetical protein CDS | CDS | 148,048 | 148,392 | 345 | forward | 148,82 | 149,164 | 345 | hypothetical protein | UJB55365.1 | 1,00E-65 | 87.72% |
| hypothetical protein CDS | CDS | 147,719 | 148,045 | 327 | forward | 148,491 | 148,817 | 327 | hypothetical protein | UJB55364.1 | 1,00E-65 | 92.59% |
| hypothetical protein CDS | CDS | 147,357 | 147,722 | 366 | forward | 148,129 | 148,494 | 366 | hypothetical protein | UJB55363.1 | 8,00E-85 | 99.17% |
| hypothetical protein CDS | CDS | 147,191 | 147,364 | 174 | forward | 147,963 | 148,136 | 174 | hypothetical protein | UJB55362.1 | 6,00E-31 | 94.74% |
| hypothetical protein CDS | CDS | 146,877 | 147,194 | 318 | forward | 147,649 | 147,966 | 318 | hypothetical protein | UJB55361.1 | 3,00E-71 | 99.05% |
| hypothetical protein CDS | CDS | 145,971 | 146,84 | 870 | forward | 146,743 | 147,612 | 870 | hypothetical protein | UJB55360.1 | 0.0 | 97.23% |
| hypothetical protein CDS | CDS | 145,023 | 145,958 | 936 | reverse | 145,795 | 146,73 | 936 | hypothetical protein | UJB55359.1 | 0.0 | 100.00% |
| hypothetical protein CDS | CDS | 144,509 | 144,973 | 465 | reverse | 145,281 | 145,745 | 465 | hypothetical protein | UJB55358.1 | 2,00E-107 | 99.35% |
| hypothetical protein CDS | CDS | 143,974 | 144,474 | 501 | forward | 144,746 | 145,246 | 501 | hypothetical protein | UJB55357.1 | 1,00E-120 | 99.40% |
| head decoration protein CDS | CDS | 143,45 | 143,953 | 504 | forward | 144,219 | 144,725 | 507 | head decoration protein | UJB55356.1 | 5,00E-114 | 98.20% |
| hypothetical protein CDS | CDS | 142,85 | 143,422 | 573 | forward | 143,618 | 144,19 | 573 | hypothetical protein | UJB55355.1 | 4,00E-137 | 98.95% |
| hypothetical protein CDS | CDS | 142,463 | 142,81 | 348 | forward | 143,23 | 143,577 | 348 | hypothetical protein | UJB55354.1 | 2,00E-78 | 99.13% |
| hypothetical protein CDS | CDS | 141,734 | 142,393 | 660 | forward | 142,501 | 143,16 | 660 | hypothetical protein | UJB55353.1 | 1,00E-153 | 95.89% |
| DNA helicase CDS | CDS | 140,164 | 141,663 | 1,5 | reverse | 140,928 | 142,43 | 1,503 | DNA helicase | UJB55352.1 | 0.0 | 99.80% |
| DNA helicase CDS | CDS | 139,924 | 140,154 | 231 | reverse | 140,688 | 140,918 | 231 | DNA helicase | UJB55351.1 | 1,00E-44 | 97.37% |
| hypothetical protein CDS | CDS | 139,694 | 139,792 | 99 | reverse | 140,457 | 140,556 | 100 | hypothetical protein | UJB55350.1 |  |  |
| hypothetical protein CDS | CDS | 139,527 | 139,709 | 183 | forward | 140,284 | 140,472 | 189 | hypothetical protein | UJB55349.1 | 3,00E-35 | 98.33% |
| hypothetical protein CDS | CDS | 139,103 | 139,525 | 423 | forward | 139,859 | 140,282 | 424 | hypothetical protein | UJB55348.1 | 5,00E-95 | 99.29% |
| capsid vertex protein CDS | CDS | 137,538 | 138,836 | 1,299 | forward | 138,269 | 139,584 | 1,316 | capsid vertex protein | UJB55347.1 | 0.0 | 99.54% |
| major capsid protein CDS | CDS | 135,87 | 137,444 | 1,575 | forward | 136,577 | 138,172 | 1,596 | major capsid protein | UJB55346.1 | 0.0 | 99.81% |
| prohead assembly (scaffolding) protein CDS | CDS | 135,054 | 135,848 | 795 | forward | 135,756 | 136,555 | 800 | prohead assembly (scaffolding) protein | UJB55345.1 | 0.0 | 98.86% |
| prohead assembly (scaffolding) protein CDS | CDS | 134,368 | 135,027 | 660 | forward | 135,065 | 135,729 | 665 | prohead assembly (scaffolding) protein | UJB55344.1 | 7,00E-154 | 99.09% |
| prohead core protein CDS | CDS | 133,958 | 134,365 | 408 | forward | 134,655 | 135,062 | 408 | prohead core protein | UJB55343.1 | 2,00E-89 | 99.26% |
| prohead core protein CDS | CDS | 133,685 | 133,945 | 261 | forward | 134,376 | 134,642 | 267 | prohead core protein | UJB55342.1 | 3,00E-46 | 95.35% |
| capsid vertex protein CDS | CDS | 132,111 | 133,685 | 1,575 | forward | 132,786 | 134,376 | 1,591 | capsid vertex protein | UJB55341.1 | 0.0 | 99.81% |
| tail tube protein CDS | CDS | 131,572 | 132,057 | 486 | forward | 132,239 | 132,726 | 488 | tail tube protein | UJB55340.1 | 2,00E-116 | 99.38% |
| tail sheath protein CDS | CDS | 129,534 | 131,525 | 1,992 | forward | 130,171 | 132,189 | 2,019 | tail sheath protein | UJB55339.1 | 0.0 | 99.85% |
| large terminase protein CDS | CDS | 127,686 | 129,515 | 1,83 | forward | 128,318 | 130,151 | 1,834 | large terminase protein | UJB55338.1 | 0.0 | 99.84% |
| small terminase protein CDS | CDS | 127,184 | 127,717 | 534 | forward | 127,784 | 128,349 | 566 | small terminase protein | UJB55337.1 | 1,00E-124 | 99.44% |
| tail sheath stabilizer and completion protein CDS | CDS | 126,36 | 127,184 | 825 | forward | 126,942 | 127,784 | 843 | tail sheath stabilizer and completion protein | UJB55336.1 | 0.0 | 99.64% |
| neck protein CDS | CDS | 125,532 | 126,278 | 747 | forward | 126,106 | 126,856 | 751 | neck protein | UJB55335.1 | 7,00E-180 | 97.98% |
| neck protein CDS | CDS | 124,599 | 125,522 | 924 | forward | 125,14 | 126,096 | 957 | neck protein | UJB55334.1 | 0.0 | 99.67% |
| hypothetical protein CDS | CDS | 122,534 | 124,564 | 2,031 | forward | 123,061 | 125,104 | 2,044 | hypothetical protein | UJB55333.1 | 0.0 | 98.37% |
| tail fibers protein CDS | CDS | 121,131 | 122,522 | 1,392 | forward | 121,637 | 123,048 | 1,412 | tail fibers protein | UJB55332.1 | 0.0 | 93.95% |
| baseplate wedge subunit and tail pin CDS | CDS | 120,456 | 121,121 | 666 | forward | 120,957 | 121,627 | 671 | baseplate wedge subunit and tail pin | UJB55331.1 | 7,00E-158 | 97.74% |
| baseplate wedge subunit and tail pin CDS | CDS | 118,639 | 120,456 | 1,818 | forward | 119,137 | 120,957 | 1,821 | baseplate wedge subunit and tail pin | UJB55330.1 | 0.0 | 98.84% |
| baseplate wedge tail fiber connector CDS | CDS | 117,779 | 118,642 | 864 | forward | 118,277 | 119,14 | 864 | baseplate wedge tail fiber connector | UJB55329.1 | 0.0 | 99.65% |
| baseplate wedge subunit CDS | CDS | 116,776 | 117,768 | 993 | forward | 117,273 | 118,265 | 993 | baseplate wedge subunit | UJB55328.1 | 0.0 | 99.39% |
| baseplate wedge subunit CDS | CDS | 113,692 | 116,775 | 3,084 | forward | 114,188 | 117,272 | 3,085 | baseplate wedge subunit | UJB55327.1 | 0.0 | 99.51% |
| baseplate wedge subunit CDS | CDS | 111,701 | 113,611 | 1,911 | forward | 112,192 | 114,107 | 1,916 | baseplate wedge subunit | UJB55326.1 | 0.0 | 99.84% |
| hypothetical protein CDS | CDS | 109,593 | 111,677 | 2,085 | forward | 110,083 | 112,168 | 2,086 | hypothetical protein | UJB55325.1 | 0.0 | 99.42% |
| baseplate hub structural protein / lysozyme R CDS | CDS | 107,824 | 109,584 | 1,761 | forward | 108,308 | 110,074 | 1,767 | baseplate hub structural protein / lysozyme R | UJB55324.1 | 0.0 | 99.66% |
| baseplate wedge protein CDS | CDS | 107,273 | 107,827 | 555 | forward | 107,757 | 108,311 | 555 | baseplate wedge protein | UJB55323.1 | 2,00E-132 | 98.91% |
| head completion protein CDS | CDS | 106,761 | 107,228 | 468 | reverse | 107,244 | 107,712 | 469 | head completion protein | UJB55322.1 | 6,00E-110 | 99.35% |
| DNA end protector protein CDS | CDS | 105,885 | 106,739 | 855 | reverse | 106,368 | 107,222 | 855 | DNA end protector protein | UJB55321.1 | 0.0 | 96.48% |
| hypothetical protein CDS | CDS | 105,241 | 105,849 | 609 | forward | 105,724 | 106,332 | 609 | hypothetical protein | UJB55320.1 | 1,00E-141 | 98.02% |
| tail completion protein CDS | CDS | 104,419 | 104,964 | 546 | reverse | 104,902 | 105,447 | 546 | tail completion protein | UJB55319.1 | 3,00E-130 | 98.34% |
| dNMP kinase CDS | CDS | 103,754 | 104,422 | 669 | reverse | 104,237 | 104,905 | 669 | dNMP kinase | UJB55318.1 | 5,00E-153 | 92.79% |
| tail fiber assembly chaperone CDS | CDS | 103,484 | 103,75 | 267 | reverse | 103,967 | 104,233 | 267 | tail fiber assembly chaperone | UJB55317.1 | 9,00E-52 | 94.32% |
| hypothetical protein CDS | CDS | 102,802 | 103,425 | 624 | reverse | 103,285 | 103,908 | 624 | hypothetical protein | UJB55316.1 | 3,00E-147 | 98.07% |
| hypothetical protein CDS | CDS | 102,241 | 102,798 | 558 | reverse | 102,724 | 103,281 | 558 | hypothetical protein | UJB55315.1 | 7,00E-129 | 96.22% |
| hypothetical protein CDS | CDS | 102,034 | 102,174 | 141 | reverse | 102,517 | 102,657 | 141 | hypothetical protein | UJB55314.1 | 2,00E-25 | 97.83% |
| hypothetical protein CDS | CDS | 101,804 | 101,944 | 141 | reverse | 102,287 | 102,427 | 141 | hypothetical protein | UJB55313.1 | 4,00E-22 | 95.65% |
| hypothetical protein CDS | CDS | 101,455 | 101,793 | 339 | reverse | 101,938 | 102,276 | 339 | hypothetical protein | UJB55312.1 | 7,00E-77 | 98.21% |
| hypothetical protein CDS | CDS | 101,162 | 101,458 | 297 | reverse | 101,645 | 101,941 | 297 | hypothetical protein | UJB55311.1 | 1,00E-66 | 98.98% |
| hypothetical protein CDS | CDS | 100,894 | 101,172 | 279 | reverse | 101,377 | 101,655 | 279 | hypothetical protein | UJB55310.1 | 1,00E-58 | 97.83% |
| hypothetical protein CDS | CDS | 100,658 | 100,897 | 240 | reverse | 101,141 | 101,38 | 240 | hypothetical protein | UJB55309.1 | 1,00E-49 | 98.73% |
| nudix hydrolase CDS | CDS | 100,245 | 100,658 | 414 | reverse | 100,728 | 101,141 | 414 | nudix hydrolase | UJB55308.1 | 6,00E-95 | 97.81% |
| hypothetical protein CDS | CDS | 99,981 | 100,145 | 165 | forward | 100,464 | 100,628 | 165 | hypothetical protein | UJB55307.1 |  |  |
| hypothetical protein CDS | CDS | 99,606 | 99,971 | 366 | reverse | 100,089 | 100,454 | 366 | hypothetical protein | UJB55306.1 | 9,00E-55 | 98.84% |
| hypothetical protein CDS | CDS | 99,262 | 99,6 | 339 | reverse | 99,745 | 100,083 | 339 | hypothetical protein | UJB55305.1 | 3,00E-73 | 98.21% |
| hypothetical protein CDS | CDS | 98,742 | 99,269 | 528 | reverse | 99,225 | 99,752 | 528 | hypothetical protein | UJB55304.1 | 3,00E-129 | 99.43% |
| hypothetical protein CDS | CDS | 98,428 | 98,745 | 318 | reverse | 98,911 | 99,228 | 318 | hypothetical protein | UJB55303.1 | 4,00E-70 | 98.10% |
| hypothetical protein CDS | CDS | 98,012 | 98,386 | 375 | forward | 98,495 | 98,869 | 375 | hypothetical protein | UJB55302.1 | 7,00E-62 | 87.83% |
| hypothetical protein CDS | CDS | 97,561 | 97,965 | 405 | reverse | 98,044 | 98,448 | 405 | hypothetical protein | UJB55301.1 | 2,00E-94 | 97.76% |
| hypothetical protein CDS | CDS | 96,974 | 97,564 | 591 | reverse | 97,457 | 98,047 | 591 | hypothetical protein | UJB55300.1 | 5,00E-140 | 96.94% |
| hypothetical protein CDS | CDS | 96,724 | 96,972 | 249 | reverse | 97,207 | 97,455 | 249 | hypothetical protein | UJB55299.1 | 1,00E-51 | 95.12% |
| hypothetical protein CDS | CDS | 96,356 | 96,634 | 279 | reverse | 96,839 | 97,117 | 279 | hypothetical protein | UJB55298.1 | 3,00E-60 | 100.00% |
| hypothetical protein CDS | CDS | 96,172 | 96,345 | 174 | reverse | 96,655 | 96,828 | 174 | hypothetical protein | UJB55297.1 | 6,00E-32 | 94.74% |
| hypothetical protein CDS | CDS | 95,866 | 96,117 | 252 | reverse | 96,349 | 96,6 | 252 | hypothetical protein | UJB55296.1 | 7,00E-52 | 98.80% |
| hypothetical protein CDS | CDS | 95,723 | 95,869 | 147 | reverse | 96,206 | 96,352 | 147 | hypothetical protein | UJB55295.1 | 6,00E-25 | 97.92% |
| hypothetical protein CDS | CDS | 95,526 | 95,726 | 201 | reverse | 96,009 | 96,209 | 201 | hypothetical protein | UJB55294.1 | 1,00E-36 | 94.12% |
| hypothetical protein CDS | CDS | 95,359 | 95,529 | 171 | reverse | 95,842 | 96,012 | 171 | hypothetical protein | UJB55293.1 | 2,00E-31 | 92.86% |
| hypothetical protein CDS | CDS | 95,039 | 95,311 | 273 | reverse | 95,522 | 95,794 | 273 | hypothetical protein | UJB55292.1 | 3,00E-56 | 100.00% |
| hypothetical protein CDS | CDS | 94,836 | 95,042 | 207 | reverse | 95,319 | 95,525 | 207 | hypothetical protein | UJB55291.1 | 3,00E-42 | 98.53% |
| hypothetical protein CDS | CDS | 93,674 | 94,882 | 1,209 | reverse | 94,157 | 95,365 | 1,209 | hypothetical protein | UJB55290.1 | 0.0 | 98.51% |
| hypothetical protein CDS | CDS | 93,381 | 93,674 | 294 | reverse | 93,864 | 94,157 | 294 | hypothetical protein | UJB55289.1 | 3,00E-63 | 97.94% |
| hypothetical protein CDS | CDS | 93,061 | 93,381 | 321 | reverse | 93,544 | 93,864 | 321 | hypothetical protein | UJB55288.1 | 2,00E-56 | 84.26% |
| hypothetical protein CDS | CDS | 92,717 | 93,064 | 348 | reverse | 93,2 | 93,547 | 348 | hypothetical protein | UJB55287.1 | 3,00E-78 | 99.13% |
| hypothetical protein CDS | CDS | 92,28 | 92,72 | 441 | reverse | 92,763 | 93,203 | 441 | hypothetical protein | UJB55286.1 | 9,00E-100 | 95.21% |
| hypothetical protein CDS | CDS | 92,013 | 92,276 | 264 | reverse | 92,496 | 92,759 | 264 | hypothetical protein | UJB55285.1 | 7,00E-56 | 98.85% |
| hypothetical protein CDS | CDS | 91,677 | 91,94 | 264 | reverse | 92,16 | 92,423 | 264 | hypothetical protein | UJB55284.1 | 3,00E-57 | 97.70% |
| hypothetical protein CDS | CDS | 91,32 | 91,64 | 321 | reverse | 91,803 | 92,123 | 321 | hypothetical protein | UJB55283.1 | 4,00E-71 | 99.06% |
| hypothetical protein CDS | CDS | 90,857 | 91,291 | 435 | reverse | 91,34 | 91,774 | 435 | hypothetical protein | UJB55282.1 | 2,00E-96 | 96.40% |
| hypothetical protein CDS | CDS | 90,394 | 90,789 | 396 | reverse | 90,877 | 91,272 | 396 | hypothetical protein | UJB55281.1 | 3,00E-89 | 99.24% |
| hypothetical protein CDS | CDS | 89,914 | 90,384 | 471 | reverse | 90,397 | 90,867 | 471 | hypothetical protein | UJB55280.1 | 3,00E-112 | 99.36% |
| hypothetical protein CDS | CDS | 88,517 | 89,86 | 1,344 | reverse | 89 | 90,343 | 1,344 | hypothetical protein | UJB55279.1 | 0.0 | 97.76% |
| hypothetical protein CDS | CDS | 88,284 | 88,478 | 195 | reverse | 88,767 | 88,961 | 195 | hypothetical protein | UJB55278.1 | 9,00E-38 | 96.88% |
| hypothetical protein CDS | CDS | 88,1 | 88,276 | 177 | reverse | 88,583 | 88,759 | 177 | hypothetical protein | UJB55277.1 | 2,00E-29 | 87.93% |
| hypothetical protein CDS | CDS | 87,534 | 88,103 | 570 | reverse | 88,017 | 88,586 | 570 | hypothetical protein | UJB55276.1 | 9,00E-137 | 99.47% |
| hypothetical protein CDS | CDS | 86,835 | 87,482 | 648 | reverse | 87,318 | 87,965 | 648 | hypothetical protein | UJB55275.1 | 4,00E-157 | 99.07% |
| hypothetical protein CDS | CDS | 86,363 | 86,767 | 405 | reverse | 86,846 | 87,25 | 405 | hypothetical protein | UJB55274.1 | 2,00E-92 | 98.51% |
| hypothetical protein CDS | CDS | 85,75 | 86,43 | 681 | reverse | 86,233 | 86,913 | 681 | hypothetical protein | UJB55273.1 | 4,00E-156 | 92.92% |
| hypothetical protein CDS | CDS | 85,417 | 85,74 | 324 | reverse | 85,9 | 86,223 | 324 | hypothetical protein | UJB55272.1 | 5,00E-69 | 97.20% |
| hypothetical protein CDS | CDS | 85,271 | 85,417 | 147 | reverse | 85,754 | 85,9 | 147 | hypothetical protein | UJB55271.1 | 3,00E-26 | 97.92% |
| hypothetical protein CDS | CDS | 85,179 | 85,274 | 96 | reverse | 85,662 | 85,757 | 96 | hypothetical protein | UJB55270.1 | 2,00E-08 | 80.65% |
| hypothetical protein CDS | CDS | 85,084 | 85,182 | 99 | reverse | 85,567 | 85,665 | 99 | hypothetical protein | UJB55269.1 | 6,00E-08 | 78.12% |
| hypothetical protein CDS | CDS | 84,603 | 85,1 | 498 | reverse | 85,086 | 85,583 | 498 | hypothetical protein | UJB55268.1 | 5,00E-108 | 90.30% |
| hypothetical protein CDS | CDS | 84,43 | 84,606 | 177 | reverse | 84,913 | 85,089 | 177 | hypothetical protein | UJB55267.1 | 5,00E-31 | 98.28% |
| hypothetical protein CDS | CDS | 84,221 | 84,433 | 213 | reverse | 84,704 | 84,916 | 213 | hypothetical protein | UJB55266.1 | 8,00E-42 | 98.57% |
| thymidine kinase CDS | CDS | 83,436 | 84,104 | 669 | reverse | 83,919 | 84,587 | 669 | thymidine kinase | UJB55265.1 | 1,00E-149 | 92.79% |
| hypothetical protein CDS | CDS | 83,013 | 83,405 | 393 | reverse | 83,496 | 83,888 | 393 | hypothetical protein | UJB55264.1 | 1,00E-90 | 98.46% |
| hypothetical protein CDS | CDS | 82,621 | 82,905 | 285 | reverse | 83,104 | 83,388 | 285 | hypothetical protein | UJB55263.1 | 5,00E-64 | 98.94% |
| hypothetical protein CDS | CDS | 82,228 | 82,617 | 390 | reverse | 82,711 | 83,1 | 390 | hypothetical protein | UJB55262.1 | 8,00E-89 | 100.00% |
| hypothetical protein CDS | CDS | 81,239 | 82,165 | 927 | reverse | 81,722 | 82,648 | 927 | hypothetical protein | UJB55261.1 | 0.0 | 99.67% |
| hypothetical protein CDS | CDS | 81,088 | 81,186 | 99 | reverse | 81,571 | 81,669 | 99 | hypothetical protein | UJB55260.1 | 7,00E-10 | 100.00% |
| hypothetical protein CDS | CDS | 80,007 | 81,062 | 1,056 | reverse | 80,49 | 81,545 | 1,056 | hypothetical protein | UJB55259.1 | 0.0 | 96.87% |
| hypothetical protein CDS | CDS | 79,913 | 80,005 | 93 | reverse | 80,396 | 80,488 | 93 | hypothetical protein | UJB55258.1 | 2,00E-15 | 80.00% |
| hypothetical protein CDS | CDS | 79,653 | 79,838 | 186 | reverse | 80,136 | 80,321 | 186 | hypothetical protein | UJB55257.1 | 8,00E-32 | 93.44% |
| hypothetical protein CDS | CDS | 79,384 | 79,653 | 270 | reverse | 79,867 | 80,136 | 270 | hypothetical protein | UJB55256.1 | 9,00E-60 | 98.88% |
| hypothetical protein CDS | CDS | 78,83 | 79,387 | 558 | reverse | 79,313 | 79,87 | 558 | hypothetical protein | UJB55255.1 | 2,00E-124 | 92.97% |
| hypothetical protein CDS | CDS | 78,582 | 78,833 | 252 | reverse | 79,065 | 79,316 | 252 | hypothetical protein | UJB55254.1 | 9,00E-53 | 96.39% |
| hypothetical protein CDS | CDS | 78,19 | 78,585 | 396 | reverse | 78,673 | 79,068 | 396 | hypothetical protein | UJB55253.1 | 1,00E-76 | 87.02% |
| hypothetical protein CDS | CDS | 77,201 | 78,181 | 981 | reverse | 77,684 | 78,664 | 981 | hypothetical protein | UJB55252.1 | 0.0 | 96.63% |
| hypothetical protein CDS | CDS | 76,941 | 77,204 | 264 | reverse | 77,424 | 77,687 | 264 | hypothetical protein | UJB55251.1 | 9,00E-45 | 84.09% |
| hypothetical protein CDS | CDS | 76,22 | 76,918 | 699 | forward | 76,703 | 77,401 | 699 | hypothetical protein | UJB55250.1 | 4,00E-152 | 90.09% |
| DNA-cytosine methyltransferase CDS | CDS | 75,284 | 76,192 | 909 | reverse | 75,767 | 76,675 | 909 | DNA-cytosine methyltransferase | UJB55249.1 | 0.0 | 98.01% |
| hypothetical protein CDS | CDS | 74,85 | 75,287 | 438 | reverse | 75,333 | 75,77 | 438 | hypothetical protein | UJB55248.1 | 2,00E-97 | 95.83% |
| hypothetical protein CDS | CDS | 74,593 | 74,808 | 216 | reverse | 75,076 | 75,291 | 216 | hypothetical protein | UJB55247.1 | 4,00E-43 | 97.18% |
| hypothetical protein CDS | CDS | 74,265 | 74,552 | 288 | reverse | 74,748 | 75,035 | 288 | hypothetical protein | UJB55246.1 | 7,00E-55 | 87.37% |
| hypothetical protein CDS | CDS | 74,028 | 74,222 | 195 | reverse | 74,511 | 74,705 | 195 | hypothetical protein | UJB55245.1 | 4,00E-30 | 90.62% |
| hypothetical protein CDS | CDS | 73,745 | 74,017 | 273 | reverse | 74,228 | 74,5 | 273 | hypothetical protein | UJB55244.1 | 4,00E-56 | 94.44% |
| hypothetical protein CDS | CDS | 73,506 | 73,748 | 243 | reverse | 73,989 | 74,231 | 243 | hypothetical protein | UJB55243.1 | 7,00E-49 | 96.25% |
| hypothetical protein CDS | CDS | 73,276 | 73,416 | 141 | reverse | 73,759 | 73,899 | 141 | hypothetical protein | UJB55242.1 | 2,00E-25 | 100.00% |
| hypothetical protein CDS | CDS | 72,767 | 73,279 | 513 | reverse | 73,25 | 73,762 | 513 | hypothetical protein | UJB55241.1 | 7,00E-124 | 98.82% |
| hypothetical protein CDS | CDS | 72,432 | 72,767 | 336 | reverse | 72,915 | 73,25 | 336 | hypothetical protein | UJB55240.1 | 1,00E-73 | 99.10% |
| hypothetical protein CDS | CDS | 70,185 | 72,431 | 2,247 | reverse | 70,668 | 72,914 | 2,247 | hypothetical protein | UJB55239.1 | 0.0 | 98.40% |
| hypothetical protein CDS | CDS | 69,521 | 70,144 | 624 | reverse | 70,004 | 70,627 | 624 | hypothetical protein | UJB55238.1 | 5,00E-135 | 94.20% |
| hypothetical protein CDS | CDS | 69,333 | 69,524 | 192 | reverse | 69,816 | 70,007 | 192 | hypothetical protein | UJB55237.1 | 2,00E-34 | 95.24% |
| hypothetical protein CDS | CDS | 68,866 | 69,336 | 471 | reverse | 69,349 | 69,819 | 471 | hypothetical protein | UJB55236.1 | 2,00E-104 | 96.15% |
| hypothetical protein CDS | CDS | 68,736 | 68,876 | 141 | reverse | 69,219 | 69,359 | 141 | hypothetical protein | UJB55235.1 | 1,00E-21 | 91.30% |
| hypothetical protein CDS | CDS | 68,442 | 68,732 | 291 | reverse | 68,925 | 69,215 | 291 | hypothetical protein | UJB55234.1 | 1,00E-62 | 98.96% |
| hypothetical protein CDS | CDS | 68,149 | 68,361 | 213 | reverse | 68,627 | 68,842 | 216 | hypothetical protein | UJB55233.1 | 4,00E-40 | 98.57% |
| hypothetical protein CDS | CDS | 67,922 | 68,152 | 231 | reverse | 68,4 | 68,63 | 231 | hypothetical protein | UJB55232.1 | 9,00E-47 | 98.68% |
| hypothetical protein CDS | CDS | 67,414 | 67,911 | 498 | reverse | 67,891 | 68,389 | 499 | hypothetical protein | UJB55231.1 | 2,00E-115 | 98.79% |
| baseplate wedge subunit CDS | CDS | 66,995 | 67,387 | 393 | forward | 67,471 | 67,864 | 394 | baseplate wedge subunit | UJB55230.1 | 1,00E-88 | 98.46% |
| baseplate hub subunit CDS | CDS | 66,434 | 66,994 | 561 | forward | 66,909 | 67,47 | 562 | baseplate hub subunit | UJB55229.1 | 2,00E-130 | 97.31% |
| hypothetical protein CDS | CDS | 65,701 | 66,441 | 741 | forward | 66,171 | 66,916 | 746 | hypothetical protein | UJB55228.1 | 5,00E-179 | 99.19% |
| baseplate hub protein CDS | CDS | 64,81 | 65,673 | 864 | forward | 65,28 | 66,143 | 864 | baseplate hub protein | UJB55227.1 | 0.0 | 99.65% |
| baseplate tail tube cap CDS | CDS | 63,715 | 64,8 | 1,086 | forward | 64,184 | 65,27 | 1,087 | baseplate tail tube cap | UJB55226.1 | 0.0 | 98.89% |
| hypothetical protein CDS | CDS | 61,958 | 63,703 | 1,746 | forward | 62,422 | 64,172 | 1,751 | hypothetical protein | UJB55225.1 | 0.0 | 97.93% |
| baseplate distal hub subunit CDS | CDS | 61,389 | 61,961 | 573 | forward | 61,844 | 62,425 | 582 | baseplate distal hub subunit | UJB55224.1 | 6,00E-133 | 99.47% |
| baseplate hub subunit CDS | CDS | 60,222 | 61,325 | 1,104 | reverse | 60,675 | 61,78 | 1,106 | baseplate hub subunit | UJB55223.1 | 0.0 | 97.00% |
| hypothetical protein CDS | CDS | 59,898 | 60,158 | 261 | forward | 60,351 | 60,611 | 261 | hypothetical protein | UJB55222.1 | 1,00E-35 | 96.61% |
| hypothetical protein CDS | CDS | 59,617 | 59,901 | 285 | forward | 60,07 | 60,354 | 285 | hypothetical protein | UJB55221.1 | 4,00E-60 | 97.87% |
| hypothetical protein CDS | CDS | 59,382 | 59,624 | 243 | forward | 59,835 | 60,077 | 243 | hypothetical protein | UJB55220.1 | 1,00E-52 | 98.75% |
| hypothetical protein CDS | CDS | 59,037 | 59,303 | 267 | forward | 59,49 | 59,756 | 267 | hypothetical protein | UJB55219.1 | 2,00E-53 | 98.86% |
| hypothetical protein CDS | CDS | 58,955 | 59,05 | 96 | forward | 59,408 | 59,503 | 96 | hypothetical protein | UJB55218.1 | 1,00E-12 | 96.77% |
| DNA ligase CDS | CDS | 57,421 | 58,944 | 1,524 | forward | 57,874 | 59,397 | 1,524 | DNA ligase | UJB55217.1 | 0.0 | 99.41% |
| hypothetical protein CDS | CDS | 56,91 | 57,443 | 534 | forward | 57,363 | 57,896 | 534 | hypothetical protein | UJB55216.1 | 2,00E-128 | 99.44% |
| hypothetical protein CDS | CDS | 56,581 | 56,829 | 249 | forward | 57,034 | 57,282 | 249 | hypothetical protein | UJB55215.1 | 1,00E-46 | 92.68% |
| hypothetical protein CDS | CDS | 56,309 | 56,578 | 270 | forward | 56,762 | 57,031 | 270 | hypothetical protein | UJB55214.1 | 3,00E-57 | 98.88% |
| hypothetical protein CDS | CDS | 56,094 | 56,312 | 219 | forward | 56,547 | 56,765 | 219 | hypothetical protein | UJB55213.1 | 1,00E-34 | 84.29% |
| hypothetical protein CDS | CDS | 55,877 | 56,083 | 207 | forward | 56,33 | 56,536 | 207 | hypothetical protein | UJB55212.1 | 4,00E-39 | 98.53% |
| hypothetical protein CDS | CDS | 55,437 | 55,88 | 444 | forward | 55,89 | 56,333 | 444 | hypothetical protein | UJB55211.1 | 9,00E-106 | 99.32% |
| hypothetical protein CDS | CDS | 55,285 | 55,437 | 153 | forward | 55,738 | 55,89 | 153 | hypothetical protein | UJB55210.1 | 4,00E-21 | 86.00% |
| hypothetical protein CDS | CDS | 55,033 | 55,209 | 177 | forward | 55,486 | 55,662 | 177 | hypothetical protein | UJB55209.1 | 5,00E-33 | 96.61% |
| hypothetical protein CDS | CDS | 54,821 | 55,051 | 231 | forward | 55,274 | 55,504 | 231 | hypothetical protein | UJB55208.1 | 1,00E-44 | 92.11% |
| hypothetical protein CDS | CDS | 54,576 | 54,824 | 249 | forward | 55,029 | 55,277 | 249 | hypothetical protein | UJB55207.1 | 1,00E-49 | 97.56% |
| hypothetical protein CDS | CDS | 54,304 | 54,576 | 273 | forward | 54,757 | 55,029 | 273 | hypothetical protein | UJB55206.1 | 2,00E-45 | 98.67% |
| hypothetical protein CDS | CDS | 54,116 | 54,346 | 231 | forward | 54,569 | 54,799 | 231 | hypothetical protein | UJB55205.1 | 3,00E-47 | 98.68% |
| hypothetical protein CDS | CDS | 53,823 | 54,113 | 291 | forward | 54,276 | 54,566 | 291 | hypothetical protein | UJB55204.1 | 8,00E-61 | 98.96% |
| hypothetical protein CDS | CDS | 53,484 | 53,753 | 270 | forward | 53,937 | 54,206 | 270 | hypothetical protein | UJB55203.1 | 2,00E-39 | 100.00% |
| hypothetical protein CDS | CDS | 53,151 | 53,432 | 282 | forward | 53,604 | 53,885 | 282 | hypothetical protein | UJB55202.1 | 9,00E-61 | 98.92% |
| hypothetical protein CDS | CDS | 52,585 | 53,151 | 567 | forward | 53,038 | 53,604 | 567 | hypothetical protein | UJB55201.1 | 3,00E-133 | 97.33% |
| hypothetical protein CDS | CDS | 52,123 | 52,542 | 420 | forward | 52,576 | 52,995 | 420 | hypothetical protein | UJB55200.1 | 7,00E-97 | 99.28% |
| hypothetical protein CDS | CDS | 51,827 | 52,111 | 285 | forward | 52,28 | 52,564 | 285 | hypothetical protein | UJB55199.1 | 4,00E-62 | 97.87% |
| hypothetical protein CDS | CDS | 51,261 | 51,512 | 252 | forward | 51,714 | 51,965 | 252 | hypothetical protein | UJB55198.1 | 3,00E-51 | 98.80% |
| hypothetical protein CDS | CDS | 50,901 | 51,257 | 357 | forward | 51,354 | 51,71 | 357 | hypothetical protein | UJB55197.1 | 3,00E-76 | 99.15% |
| dCMP deaminase CDS | CDS | 50,384 | 50,908 | 525 | forward | 50,837 | 51,361 | 525 | dCMP deaminase | UJB55196.1 | 7,00E-126 | 98.85% |
| hypothetical protein CDS | CDS | 50,127 | 50,387 | 261 | forward | 50,58 | 50,84 | 261 | hypothetical protein | UJB55195.1 | 8,00E-57 | 98.84% |
| hypothetical protein CDS | CDS | 49,864 | 50,13 | 267 | forward | 50,317 | 50,583 | 267 | hypothetical protein | UJB55194.1 | 3,00E-54 | 97.73% |
| polynucleotide kinase CDS | CDS | 48,916 | 49,803 | 888 | forward | 49,369 | 50,256 | 888 | polynucleotide kinase | UJB55193.1 | 0.0 | 99.32% |
| hypothetical protein CDS | CDS | 48,725 | 48,919 | 195 | forward | 49,178 | 49,372 | 195 | hypothetical protein | UJB55192.1 |  |  |
| hypothetical protein CDS | CDS | 48,447 | 48,764 | 318 | forward | 48,9 | 49,217 | 318 | hypothetical protein | UJB55191.1 | 3,00E-67 | 96.19% |
| hypothetical protein CDS | CDS | 48,124 | 48,45 | 327 | forward | 48,577 | 48,903 | 327 | hypothetical protein | UJB55190.1 | 1,00E-63 | 89.81% |
| RNA ligase CDS | CDS | 46,97 | 48,127 | 1,158 | forward | 47,423 | 48,58 | 1,158 | RNA ligase | UJB55189.1 | 0.0 | 97.92% |
| homing endonuclease CDS | CDS | 46,609 | 47,028 | 420 | forward | 47,062 | 47,481 | 420 | homing endonuclease | UJB55188.1 | 3,00E-95 | 95.68% |
| hypothetical protein CDS | CDS | 46,298 | 46,609 | 312 | forward | 46,751 | 47,062 | 312 | hypothetical protein | UJB55187.1 | 6,00E-69 | 99.03% |
| ribonucleotide reductase of class Ia (aerobic), beta subunit CDS | CDS | 45,664 | 46,326 | 663 | forward | 46,117 | 46,779 | 663 | ribonucleotide reductase of class Ia (aerobic), beta subunit | UJB55186.1 | 1,00E-161 | 100.00% |
| ribonucleotide reductase of class Ia (aerobic), beta subunit CDS | CDS | 45,024 | 45,629 | 606 | forward | 45,476 | 46,082 | 607 | ribonucleotide reductase of class Ia (aerobic), beta subunit | UJB55185.1 | 2,00E-144 | 99.50% |
| ribonucleotide reductase of class Ia (aerobic), alpha subunit CDS | CDS | 42,737 | 44,986 | 2,25 | forward | 43,177 | 45,435 | 2,259 | ribonucleotide reductase of class Ia (aerobic), alpha subunit | UJB55184.1 | 0.0 | 99.87% |
| thymidylate synthase CDS | CDS | 41,752 | 42,696 | 945 | forward | 42,178 | 43,136 | 959 | thymidylate synthase | UJB55183.1 | 0.0 | 98.94% |
| dihydrofolate reductase CDS | CDS | 41,259 | 41,84 | 582 | forward | 41,672 | 42,272 | 601 | dihydrofolate reductase | UJB55182.1 | 6,00E-135 | 94.30% |
| hypothetical protein CDS | CDS | 41,005 | 41,262 | 258 | forward | 41,416 | 41,675 | 260 | hypothetical protein | UJB55181.1 | 4,00E-55 | 100.00% |
| hypothetical protein CDS | CDS | 40,769 | 41,008 | 240 | forward | 41,174 | 41,419 | 246 | hypothetical protein | UJB55180.1 | 3,00E-47 | 96.20% |
| hypothetical protein CDS | CDS | 40,452 | 40,784 | 333 | forward | 40,845 | 41,189 | 345 | hypothetical protein | UJB55179.1 | 1,00E-70 | 92.73% |
| hypothetical protein CDS | CDS | 38,688 | 40,391 | 1,704 | forward | 39,039 | 40,781 | 1,743 | hypothetical protein | UJB55178.1 | 0.0 | 98.77% |
| hypothetical protein CDS | CDS | 38,425 | 38,616 | 192 | forward | 38,775 | 38,966 | 192 | hypothetical protein | UJB55177.1 | 2,00E-34 | 93.65% |
| recombination endonuclease subunit CDS | CDS | 36,728 | 38,431 | 1,704 | forward | 37,072 | 38,781 | 1,71 | recombination endonuclease subunit | UJB55176.1 | 0.0 | 97.53% |
| hypothetical protein CDS | CDS | 36,523 | 36,753 | 231 | forward | 36,856 | 37,098 | 243 | hypothetical protein | UJB55175.1 | 1,00E-47 | 100.00% |
| recombination endonuclease subunit CDS | CDS | 35,451 | 36,47 | 1,02 | forward | 35,773 | 36,797 | 1,025 | recombination endonuclease subunit | UJB55174.1 | 0.0 | 98.82% |
| hypothetical protein CDS | CDS | 35,08 | 35,424 | 345 | forward | 35,373 | 35,745 | 373 | hypothetical protein | UJB55173.1 | 1,00E-72 | 95.61% |
| hypothetical protein CDS | CDS | 34,869 | 35,096 | 228 | forward | 35,159 | 35,389 | 231 | hypothetical protein | UJB55172.1 | 5,00E-44 | 98.67% |
| sigma factor for late transcription CDS | CDS | 34,33 | 34,872 | 543 | forward | 34,619 | 35,162 | 544 | sigma factor for late transcription | UJB55171.1 | 3,00E-124 | 100.00% |
| hypothetical protein CDS | CDS | 34,053 | 34,199 | 147 | forward | 34,322 | 34,485 | 164 | hypothetical protein | UJB55170.1 |  |  |
| ribonuclease CDS | CDS | 33,105 | 34,04 | 936 | reverse | 33,359 | 34,309 | 951 | ribonuclease | UJB55169.1 | 0.0 | 99.04% |
| dsDNA binding protein CDS | CDS | 32,832 | 33,095 | 264 | reverse | 33,082 | 33,349 | 268 | dsDNA binding protein | UJB55168.1 | 7,00E-46 | 85.06% |
| hypothetical protein CDS | CDS | 32,579 | 32,83 | 252 | reverse | 32,828 | 33,08 | 253 | hypothetical protein | UJB55167.1 | 2,00E-53 | 100.00% |
| loader of DNA helicase CDS | CDS | 31,917 | 32,501 | 585 | reverse | 32,165 | 32,75 | 586 | loader of DNA helicase | UJB55166.1 | 2,00E-137 | 100.00% |
| putative single-stranded DNA binding protein CDS | CDS | 30,933 | 31,913 | 981 | reverse | 31,148 | 32,161 | 1,014 | putative single-stranded DNA binding protein | UJB55165.1 | 0.0 | 98.16% |
| RNA polymerase binding protein CDS | CDS | 30,603 | 30,887 | 285 | reverse | 30,793 | 31,095 | 303 | RNA polymerase binding protein | UJB55164.1 | 1,00E-62 | 98.94% |
| hypothetical protein CDS | CDS | 29,917 | 30,582 | 666 | reverse | 30,106 | 30,772 | 667 | hypothetical protein | UJB55163.1 | 2,00E-156 | 99.10% |
| DNA polymerase clamp loader subunit CDS | CDS | 28,854 | 29,852 | 999 | reverse | 29,025 | 30,035 | 1,011 | DNA polymerase clamp loader subunit | UJB55162.1 | 0.0 | 99.40% |
| DNA polymerase clamp loader subunit CDS | CDS | 28,28 | 28,852 | 573 | reverse | 28,446 | 29,023 | 578 | DNA polymerase clamp loader subunit | UJB55161.1 | 1,00E-135 | 99.47% |
| endoribonuclease translational repressor of early genes CDS | CDS | 27,908 | 28,276 | 369 | reverse | 28,065 | 28,442 | 378 | endoribonuclease translational repressor of early genes | UJB55160.1 | 1,00E-82 | 100.00% |
| hypothetical protein CDS | CDS | 27,522 | 27,839 | 318 | reverse | 27,65 | 27,99 | 341 | hypothetical protein | UJB55159.1 | 5,00E-69 | 99.05% |
| hypothetical protein CDS | CDS | 24,824 | 27,529 | 2,706 | reverse | 24,944 | 27,657 | 2,714 | hypothetical protein | UJB55158.1 | 0.0 | 99.56% |
| recombination protein CDS | CDS | 23,598 | 24,743 | 1,146 | reverse | 23,69 | 24,862 | 1,173 | recombination protein | UJB55157.1 | 0.0 | 99.21% |
| head assembly chaperone protein CDS | CDS | 23,253 | 23,558 | 306 | reverse | 23,333 | 23,64 | 308 | head assembly chaperone protein | UJB55156.1 | 9,00E-65 | 99.01% |
| DNA helicase CDS | CDS | 21,804 | 23,243 | 1,44 | reverse | 21,88 | 23,323 | 1,444 | DNA helicase | UJB55155.1 | 0.0 | 99.79% |
| hypothetical protein CDS | CDS | 21,559 | 21,768 | 210 | reverse | 21,624 | 21,842 | 219 | hypothetical protein | UJB55154.1 | 4,00E-34 | 98.55% |
| hypothetical protein CDS | CDS | 21,225 | 21,479 | 255 | reverse | 21,273 | 21,532 | 260 | hypothetical protein | UJB55153.1 | 1,00E-52 | 98.81% |
| hypothetical protein CDS | CDS | 20,899 | 21,129 | 231 | reverse | 20,939 | 21,176 | 238 | hypothetical protein | UJB55152.1 | 4,00E-42 | 98.68% |
| hypothetical protein CDS | CDS | 20,507 | 20,902 | 396 | reverse | 20,543 | 20,942 | 400 | hypothetical protein | UJB55151.1 | 3,00E-87 | 97.71% |
| hypothetical protein CDS | CDS | 19,792 | 20,571 | 780 | reverse | 19,811 | 20,607 | 797 | hypothetical protein | UJB55150.1 | 0.0 | 95.02% |
| hypothetical protein CDS | CDS | 19,292 | 19,786 | 495 | reverse | 19,299 | 19,805 | 507 | hypothetical protein | UJB55149.1 | 1,00E-113 | 96.93% |
| hypothetical protein CDS | CDS | 19,144 | 19,305 | 162 | reverse | 19,144 | 19,324 | 181 | hypothetical protein | UJB55148.1 | 6,00E-19 | 88.89% |
| hypothetical protein CDS | CDS | 18,926 | 19,147 | 222 | reverse | 18,926 | 19,147 | 222 | hypothetical protein | UJB55147.1 | 9,00E-45 | 93.15% |
| hypothetical protein CDS | CDS | 18,738 | 18,929 | 192 | reverse | 18,738 | 18,929 | 192 | hypothetical protein | UJB55146.1 | 2,00E-35 | 93.65% |
| DNA primase subunit CDS | CDS | 17,71 | 18,738 | 1,029 | reverse | 17,71 | 18,738 | 1,029 | DNA primase subunit | UJB55145.1 | 0.0 | 99.71% |
| dUTPase CDS | CDS | 17,169 | 17,768 | 600 | reverse | 17,169 | 17,768 | 600 | dUTPase | UJB55144.1 | 6,00E-129 | 100.00% |
| hypothetical protein CDS | CDS | 16,987 | 17,169 | 183 | reverse | 16,987 | 17,169 | 183 | hypothetical protein | UJB55143.1 | 2,00E-29 | 90.00% |
| hypothetical protein CDS | CDS | 16,703 | 16,99 | 288 | reverse | 16,703 | 16,99 | 288 | hypothetical protein | UJB55142.1 | 4,00E-62 | 98.95% |
| DNA helicase CDS | CDS | 15,375 | 16,706 | 1,332 | reverse | 15,375 | 16,706 | 1,332 | DNA helicase | UJB55141.1 | 0.0 | 99.77% |
| hypothetical protein CDS | CDS | 15,037 | 15,348 | 312 | reverse | 15,037 | 15,348 | 312 | hypothetical protein | UJB55140.1 | 2,00E-67 | 96.12% |
| hypothetical protein CDS | CDS | 14,744 | 15,052 | 309 | reverse | 14,744 | 15,052 | 309 | hypothetical protein | UJB55139.1 | 3,00E-64 | 97.96% |
| hypothetical protein CDS | CDS | 14,076 | 14,747 | 672 | reverse | 14,076 | 14,747 | 672 | hypothetical protein | UJB55138.1 | 5,00E-161 | 98.21% |
| hypothetical protein CDS | CDS | 13,798 | 14,079 | 282 | reverse | 13,798 | 14,079 | 282 | hypothetical protein | UJB55137.1 | 5,00E-62 | 98.92% |
| hypothetical protein CDS | CDS | 13,586 | 13,729 | 144 | reverse | 13,586 | 13,729 | 144 | hypothetical protein | UJB55136.1 | 4,00E-21 | 95.74% |
| hypothetical protein CDS | CDS | 13,366 | 13,575 | 210 | reverse | 13,366 | 13,575 | 210 | hypothetical protein | UJB55135.1 | 1,00E-39 | 89.86% |
| hypothetical protein CDS | CDS | 13,058 | 13,369 | 312 | reverse | 13,058 | 13,369 | 312 | hypothetical protein | UJB55134.1 | 2,00E-65 | 94.17% |
| hypothetical protein CDS | CDS | 12,808 | 12,969 | 162 | reverse | 12,808 | 12,969 | 162 | hypothetical protein | UJB55133.1 | 8,00E-27 | 100.00% |
| hypothetical protein CDS | CDS | 12,608 | 12,82 | 213 | reverse | 12,608 | 12,82 | 213 | hypothetical protein | UJB55132.1 | 6,00E-40 | 100.00% |
| hypothetical protein CDS | CDS | 12,448 | 12,615 | 168 | reverse | 12,448 | 12,615 | 168 | hypothetical protein | UJB55131.1 | 2,00E-29 | 98.18% |
| hypothetical protein CDS | CDS | 12,16 | 12,372 | 213 | reverse | 12,16 | 12,372 | 213 | hypothetical protein | UJB55130.1 | 7,00E-41 | 98.57% |
| hypothetical protein CDS | CDS | 12,008 | 12,163 | 156 | reverse | 12,008 | 12,163 | 156 | hypothetical protein | UJB55129.1 | 1,00E-27 | 100.00% |
| hypothetical protein CDS | CDS | 11,783 | 12,004 | 222 | reverse | 11,783 | 12,004 | 222 | hypothetical protein | UJB55128.1 | 2,00E-41 | 94.52% |
| hypothetical protein CDS | CDS | 11,404 | 11,736 | 333 | reverse | 11,404 | 11,736 | 333 | hypothetical protein | UJB55127.1 | 1,00E-73 | 98.18% |
| hypothetical protein CDS | CDS | 10,781 | 11,371 | 591 | reverse | 10,781 | 11,371 | 591 | hypothetical protein | UJB55126.1 | 2,00E-142 | 97.96% |
| hypothetical protein CDS | CDS | 10,485 | 10,784 | 300 | reverse | 10,485 | 10,784 | 300 | hypothetical protein | UJB55125.1 | 6,00E-67 | 98.99% |
| hypothetical protein CDS | CDS | 9,893 | 10,498 | 606 | reverse | 9,893 | 10,498 | 606 | hypothetical protein | UJB55124.1 | 2,00E-144 | 96.52% |
| hypothetical protein CDS | CDS | 9,438 | 9,872 | 435 | reverse | 9,438 | 9,872 | 435 | hypothetical protein | UJB55123.1 | 6,00E-103 | 99.31% |
| hypothetical protein CDS | CDS | 9,274 | 9,438 | 165 | reverse | 9,274 | 9,438 | 165 | hypothetical protein | UJB55122.1 | 1,00E-29 | 96.30% |
| topoisomerase II large subunit CDS | CDS | 7,309 | 9,204 | 1,896 | reverse | 7,309 | 9,204 | 1,896 | topoisomerase II large subunit | UJB55121.1 | 0.0 | 99.68% |
| DNA topoisomerase subunit CDS | CDS | 6,023 | 7,312 | 1,29 | reverse | 6,023 | 7,312 | 1,29 | DNA topoisomerase subunit | UJB55120.1 | 0.0 | 99.77% |
| hypothetical protein CDS | CDS | 5,412 | 5,951 | 540 | reverse | 5,412 | 5,951 | 540 | hypothetical protein | UJB55119.1 | 5,00E-131 | 99.44% |
| hypothetical protein CDS | CDS | 5,179 | 5,415 | 237 | reverse | 5,179 | 5,415 | 237 | hypothetical protein | UJB55118.1 | 2,00E-48 | 97.44% |
| hypothetical protein CDS | CDS | 2,919 | 5,168 | 2,25 | reverse | 2,919 | 5,168 | 2,25 | hypothetical protein | UJB55117.1 | 0.0 | 98.93% |
| rIIB protein CDS | CDS | 2,017 | 2,919 | 903 | reverse | 2,017 | 2,919 | 903 | rIIB protein | UJB55116.1 | 0.0 | 99.67% |
| hypothetical protein CDS | CDS | 1,555 | 1,971 | 417 | reverse | 1,555 | 1,971 | 417 | hypothetical protein | UJB55115.1 | 4,00E-92 | 97.83% |
| nucleoid disruption protein CDS | CDS | 1,143 | 1,583 | 441 | reverse | 1,143 | 1,583 | 441 | nucleoid disruption protein | UJB55114.1 | 4,00E-102 | 99.32% |
| hypothetical protein CDS | CDS | 880 | 1,134 | 255 | reverse | 880 | 1,134 | 255 | hypothetical protein | UJB55113.1 | 3,00E-50 | 98.81% |
| hypothetical protein CDS | CDS | 394 | 810 | 417 | reverse | 394 | 810 | 417 | hypothetical protein | UJB55112.1 | 1,00E-94 | 97.10% |
| hypothetical protein CDS | CDS | 170 | 397 | 228 | reverse | 170 | 397 | 228 | hypothetical protein | UJB55111.1 | 4,00E-46 | 94.59% |
