# Supplementary material for: Characterization of Enterobacter phage vB_EcRAM-01, a new Pseudotevenvirus against Enterobacter cloacae, isolated in an urban river in Panama
Source: PLoS One. 2024 Dec 31;19(12):e0310824. doi: 10.1371/journal.pone.0310824 (PMC11687723; doi:10.1371/journal.pone.0310824)
Supplement: Attachment — Submitted filename: PONE-D-23-39735_R1review.pdf [file pone.0310824.s006.pdf]

PLOS ONE

Characterization of Enterobacter phage vB_EcRAM-01, a new Pseudotevenvirus against Enterobacter cloacae , isolated in an urban river in Panama.

--Manuscript Draft--

**Manuscript Number:**	PONE-D-23-39735R1
**Article Type:**	Research Article
**Full Title:**	Characterization of Enterobacter phage vB_EcRAM-01, a new Pseudotevenvirus against Enterobacter cloacae , isolated in an urban river in Panama.
**Short Title:**	Characterization a new Pseudotevenvirus against Enterobacter cloacae
**Corresponding Author:**	Álex Omar Matínez-Torres, Doctor<br>Universidad de Panamá: Universidad de Panama<br>PANAMA
**Keywords:**	Bacteriophage, E. cloacae complex, phage therapy, molecular characterization, whole genome sequencing, "de novo" assembly
**Abstract:**	The Enterobacter cloacae complex, a prominent bacterium responsible worldwide for most bloodstream infections in the hospital environment, has shown broad-spectrum antibiotic resistance, including carbapenems. Therefore, bacteriophages have again attracted the attention of the science and medical community as an alternative to control MDR bacteria. In this study, water samples from Río Abajo River, in Panama City, Panama, were collected, for phage isolation, purification, characterization and propagation against the E. cloacae complex. As result, a phage produced clear and round plaque-forming units indicating a lytic phage was isolated. Further analyses concluded that this phage is stable at temperatures between 25°C and 50°C, it remains infective in a pH range between 7 to 11, with high sensitivity to UV light. Remarkedly, it exhibits a narrow host specificity only infecting E. cloacae. Whole genome sequencing revealed that is a myovirus with a genome size of 178,477 bp, a G-C content of 45.8%, and containing approximately 294 genes. Among them, protein-encoding genes involved in morphology, inactivation, adsorption to cells, DNA injection and lytic enzymes were identified. Additionally, the genome contained two tRNA sequences. Genes that encode holins and endolysins, typical of lytic bacteriophages, were also present. A whole-genome sequencing analysis indicated that, according to the genus demarcation criteria, this phage belongs to a novel species within the Family Straboviridae, called genusPseudotevenvirus.
**Order of Authors:**	Jordi Querol-Audi, Ph.D.
	Álex Omar Matínez-Torres, Doctor
	Ednner Victoria-Blanco, Magister
	Jean Pierre González-Gómez
	Juan Raul Medina-Sánchez
	Alexander Martínez
	Nohelia Castro del Campo
	Cristóbal Cháidez-Quirós
**Response to Reviewers:**	Panama, Panama, March 23, 2024<br>Response to the Editor and reviewers:<br><br>1. Please ensure that your manuscript meets PLOS ONE's style requirements,

Powered by Editorial Manager® and ProduXion Manager® from Aries Systems Corporation

including those for file naming. The PLOS ONE style templates can be found at https://journals.plos.org/plosone/s/file?id=wjVg/PLOSOne_formatting_sample_main_body.pdf and https://journals.plos.org/plosone/s/file?id=ba62/PLOSOne_formatting_sample_title_authors_affiliations.pdf

•Style requirements have been carefully reviewed.

2. We note that the grant information you provided in the 'Funding Information' and 'Financial Disclosure' sections do not match.

When you resubmit, please ensure that you provide the correct grant numbers for the awards you received for your study in the 'Funding Information' section.
•Grant numbers have been corrected (see below).

3. Thank you for stating the following financial disclosure:
"This work was funded in part by the Sistema Nacional de Investigación (SNI) of the Secretaría Nacional de Ciencia, Tecnología e Innovación (SENACYT, Panama), through grants 87-2019, 81-2019 and FID18-044, and also from the Water, Protected Areas and Wildlife Trust of the Ministry of Environment of Panama through grant 026-45-2019 and grant 111130406 from the Ministry of Economy and Finance of Panama."

Please state what role the funders took in the study. If the funders had no role, please state: ""The funders had no role in study design, data collection and analysis, decision to publish, or preparation of the manuscript.""
If this statement is not correct you must amend it as needed.
Please include this amended Role of Funder statement in your cover letter; we will change the online submission form on your behalf.
•The role of funders has been included

4. Thank you for stating the following in the Acknowledgments Section of your manuscript:
"This work was funded in part by the Sistema Nacional de Investigación (SNI) of the Secretaría Nacional de Ciencia, Tecnología e Innovación (SENACYT, Panama), through grants 87-2019, 81-2019 and FID18-044, and also from the Water, Protected Areas and Wildlife Trust of the Ministry of Environment of Panama through grant 026-45-2019 and grant 111130406 from the Ministry of Economy and Finance of Panama. Clinical bacterial strains were provided by José E. Moreno, MSc., of the Microbiology Division of Laboratorio Central de Referencia en Salud Pública, Instituto Conmemorativo Gorgas de Estudios de Salud (ICGES) and the Department of Microbiology, Hospital Santo Tomás."

We note that you have provided funding information that is not currently declared in your Funding Statement. However, funding information should not appear in the Acknowledgments section or other areas of your manuscript. We will only publish funding information present in the Funding Statement section of the online submission form.
Please remove any funding-related text from the manuscript and let us know how you would like to update your Funding Statement. Currently, your Funding Statement reads as follows:
"This work was funded in part by the Sistema Nacional de Investigación (SNI) of the Secretaría Nacional de Ciencia, Tecnología e Innovación (SENACYT, Panama), through grants 87-2019, 81-2019 and FID18-044, and also from the Water, Protected Areas and Wildlife Trust of the Ministry of Environment of Panama through grant 026-45-2019 and grant 111130406 from the Ministry of Economy and Finance of Panama."

Please include your amended statements within your cover letter; we will change the online submission form on your behalf.

•This has been included in the cover letter and removed from the acknowledgments

section.

5. Please amend the manuscript submission data (via Edit Submission) to include author Alex Omar Martínez-Torres, Cristóbal Chaidez-Quiróz, Nohelia Castro del Campo, Alexander A. Martínez, Juan Raúl Medina-Sánchez, Jean Pierre González-Gómez and Ednner E. Victoria-Blanco.
•Author's information has been included.

6. Please include captions for your Supporting Information files at the end of your manuscript, and update any in-text citations to match accordingly. Please see our Supporting Information guidelines for more information: http://journals.plos.org/plosone/s/supporting-information.
•Figure captions for Supporting Information have been included at the end of the revised version.

Reviewer#1

The paper offers a structured framework for characterization of Enterobacter phage vB_EcRAM-01, a new Pseudotevenvirus against Enterobacter cloacae, isolated in an urban river in Panama.
The authors have conducted many right experiments, but other experiments must be carried out to complete characterization of isolated phage and show the importance of this work. To help the authors improve their manuscript, we have included both general and detailed comments below:
1. It is necessary to describe phage vB_EcRAM-01 infection cycle.
•We respectfully acknowledge the reviewer's comment regarding the description of the infection cycle of phage vB_EcRAM-01. While we appreciate the importance of understanding the infection dynamics of bacteriophages, we opted not to include a detailed description of the infection cycle using methodologies such as the one-step curve. This decision was based on several factors: Firstly, the high variability in results due to technical errors can significantly impact the accuracy of burst size measurements. Secondly, these techniques require a substantial amount of material, which may not be feasible in all research settings. Instead, we focused our characterization efforts on essential tests such as host range determination, bacteriolytic activity assays, and genomic sequencing, which collectively provide valuable insights into the potential therapeutic applications of phage vB_EcRAM-01.

2. Morphological characterization of phage using transmission electron microscopy (TEM) should be done.
•The TEM micrograph (S2 Fig) as well as a description of the overall morphology of the virus has been included in the Bacteriophage Isolation, Host Range Analysis and Morphology Characterization section (lines 178-181).

3. Bacterial reduction assay should be carried out.
•The Bacterial Reduction assay was performed and included in the Bacteriolytic Activity section (Fig 3; lines 196-203) following the reviewer's recommendation.

4. How did you perform purification of phage particles?
•Our phage purification process involved serial dilution of filtrates exhibiting lytic activity against E. cloacae. Specifically, 100 µL of the phage dilution was mixed with an overnight culture of the host bacterium in 3 mL of soft agar (TSB with 0.4% agar), poured onto TSA plates, and incubated at 37 °C for 24 h. The phage plates, selected based on size and clarity, were transferred to microtubes containing 1 mL of nanopure water and this process was repeated at least three times to confirm that the isolated phages were descendants of a single virion.
This has been included in the Materials and Methos section.

5. Have you identified the structural proteins of isolated phage by SDS-PAGE gel electrophoresis? It will be great to have the proteomic analysis of this phage.
•Following the reviewer's suggestion we visualized the main protein components of the

isolated phage. The image has been included in the new version (S4 Fig) as well as a description of the results (lanes 207-216).

6. Please state the GenBank accession no. of phage genome.
•The GenBank accession no. was declared in the Data availability statement section.

7. What is the percentage of a G + C content of phage genomic DNA.
•The GC content was declared in the Comparative Genomics and Phylogenetic Analysis section.

8. Please describe distinguished genes found in the genome of phage vB_EcRAM-01.
•Additional information on the main traits found in the genome were added in the discussion section.

9. Correct the list of references. Some citations are not present in the reference list.
•References have been corrected in the revised version.

Reviewer #2:
This manuscript provides data for the biological characterization and genomic analysis of Enterobacter cloacae phage vB_EcRAM-01. Results presented in this study contribute to a greater understanding of the biology of phages and expand our knowledge of Enterobacter cloacae phages. However, the overall format of the article is not consistent with the requirements of the official website of PLOS ONE, please download the manuscript formatting guidelines from the official website of PLOS ONE and modify the article format according to the guidelines.

Major comments

1. Line 19- Enterobacter cloacae should not be abbreviated when it first appears in the keyword section.
•This correction has been made in the current version.

2. Line 39- The font of Enterobacteriaceae should be italic, please check the full text.
•Enterobacteriaceae was italicized.

3. Line 108-110- The experimental conditions in the text are 25 ℃, 50 ℃, 70 ℃ and 90 ℃, but Figure 2B shows 25 ℃, 37 ℃, 50 ℃ and 70 ℃. The temperature unit is not written in Figure 2B. The range of pH in Figure 2C is also inconsistent with the text.
•The correction was made.

4. Figure 2- The unit of the horizontal coordinate of Figure 2 B should be Time (min).
•This has been corrected in the current version.

5. Figure 2- In Figure 2 B, why does the number of phage vB_EcRAM-01 gradually increase at 37 °C?
•Reviewer is right. There was an error when calculating UFP per milliliter when undoing the 10-fold dilutions. This has been fixed in the current version.

6. Line 180- Please write down the correct unit of temperature.
•The correction was made.

7. Line 187-215, 257-266- The description of the genome of phage vB_EcRAM-01 is relatively simple. Please provide more details about the genome.
•Additional information on the main traits found in the genome were added in the discussion section.

8. Figure 3- Please add colors to phage EBPL and phage Margaery to better compare phage vB_EcRAM-01 with these two phages.
•Colors were added to the figure.

9. Figure 4, Figure 5- Please change the font of the genus names in italics on Figures 4 and 5.
•Genus names were italicized.

Additional Information:	
**Question**	**Response**
**Financial Disclosure**<br><br>Enter a financial disclosure statement that describes the sources of funding for the work included in this submission. Review the submission guidelines for detailed requirements. View published research articles from *PLOS ONE* for specific examples.<br><br>This statement is required for submission and **will appear in the published article** if the submission is accepted. Please make sure it is accurate.<br><br>**Funded studies**<br>Enter a statement with the following details:<br>• Initials of the authors who received each award<br>• Grant numbers awarded to each author<br>• The full name of each funder<br>• URL of each funder website<br>• Did the sponsors or funders play any role in the study design, data collection and analysis, decision to publish, or preparation of the manuscript?<br><br>Did you receive funding for this work?	Yes
Please add funding details.<br>as follow-up to "**Financial Disclosure**<br><br>Enter a financial disclosure statement that describes the sources of funding for the work included in this submission. Review the submission guidelines for detailed requirements. View published research articles from *PLOS ONE* for specific examples.<br><br>This statement is required for submission and **will appear in the published article** if the submission is accepted. Please make sure it is accurate.	This work was funded in part by the Sistema Nacional de Investigación (SNI) of the Secretaría Nacional de Ciencia, Tecnología e Innovación (SENACYT, Panama), through grants 87-2019, 81-2019 and FID18-044, and also from the Water, Protected Areas and Wildlife Trust of the Ministry of Environment of Panama through grant 026-45-2019 and grant 111130406 from the Ministry of Economy and Finance of Panama.

Powered by Editorial Manager® and ProduXion Manager® from Aries Systems Corporation

**Funded studies**<br>Enter a statement with the following details:<br>• Initials of the authors who received each award<br>• Grant numbers awarded to each author<br>• The full name of each funder<br>• URL of each funder website<br>• Did the sponsors or funders play any role in the study design, data collection and analysis, decision to publish, or preparation of the manuscript?<br><br>Did you receive funding for this work?"	
Please select the country of your main research funder (please select carefully as in some cases this is used in fee calculation).<br>as follow-up to "**Financial Disclosure**<br><br>Enter a financial disclosure statement that describes the sources of funding for the work included in this submission. Review the submission guidelines for detailed requirements. View published research articles from *PLOS ONE* for specific examples.<br><br>This statement is required for submission and **will appear in the published article** if the submission is accepted. Please make sure it is accurate.<br><br>**Funded studies**<br>Enter a statement with the following details:<br>• Initials of the authors who received each award<br>• Grant numbers awarded to each author<br>• The full name of each funder<br>• URL of each funder website<br>• Did the sponsors or funders play any role in the study design, data collection and analysis, decision to publish, or preparation of the manuscript?<br><br>Did you receive funding for this work?"	PANAMA - PA
**Competing Interests**	The authors have declared that no competing interests exist.

Use the instructions below to enter a competing interest statement for this submission. On behalf of all authors, disclose any competing interests that could be perceived to bias this work—acknowledging all financial support and any other relevant financial or non-financial competing interests.<br><br>This statement is required for submission and **will appear in the published article** if the submission is accepted. Please make sure it is accurate and that any funding sources listed in your Funding Information later in the submission form are also declared in your Financial Disclosure statement.<br><br>View published research articles from *PLOS ONE* for specific examples.<br><br>**NO authors have competing interests**<br><br>Enter: *The authors have declared that no competing interests exist.*<br><br>**Authors with competing interests**<br><br>Enter competing interest details beginning with this statement:<br><br>*I have read the journal's policy and the authors of this manuscript have the following competing interests: [insert competing interests here]*<br><br>* typeset	
**Ethics Statement**<br><br>Enter an ethics statement for this submission. This statement is required if the study involved:<br><br>• Human participants<br>• Human specimens or tissue<br>• Vertebrate animals or cephalopods<br>• Vertebrate embryos or tissues	The Bioethics Committee of the Universidad de Panamá with the endorsement of the Dirección General de Salud Pública of the Ministerio de Salud (Ministry of Health), approved this study (Sequential: 617). The samples were analyzed anonymously.

• Field research<br><br>Write "N/A" if the submission does not require an ethics statement.<br><br>General guidance is provided below. Consult the submission guidelines for detailed instructions. **Make sure that all information entered here is included in the Methods section of the manuscript.**	

**Format for specific study types**<br><br>**Human Subject Research (involving human participants and/or tissue)**<br>• Give the name of the institutional review board or ethics committee that approved the study<br>• Include the approval number and/or a statement indicating approval of this research<br>• Indicate the form of consent obtained (written/oral) or the reason that consent was not obtained (e.g. the data were analyzed anonymously)<br><br>**Animal Research (involving vertebrate animals, embryos or tissues)**<br>• Provide the name of the Institutional Animal Care and Use Committee (IACUC) or other relevant ethics board that reviewed the study protocol, and indicate whether they approved this research or granted a formal waiver of ethical approval<br>• Include an approval number if one was obtained<br>• If the study involved *non-human primates*, add *additional details* about animal welfare and steps taken to ameliorate suffering<br>• If anesthesia, euthanasia, or any kind of animal sacrifice is part of the study, include briefly which substances and/or methods were applied<br><br>**Field Research**<br><br>Include the following details if this study involves the collection of plant, animal, or other materials from a natural setting:<br>• Field permit number<br>• Name of the institution or relevant body that granted permission	
**Data Availability**<br><br>Authors are required to make all data underlying the findings described fully available, without restriction, and from the time of publication. PLOS allows rare exceptions to address legal and ethical concerns. See the PLOS Data Policy and FAQ for detailed information.	Yes - all data are fully available without restriction

A Data Availability Statement describing where the data can be found is required at submission. Your answers to this question constitute the Data Availability Statement and **will be published in the article**, if accepted.<br><br>**Important:** Stating 'data available on request from the author' is not sufficient. If your data are only available upon request, select 'No' for the first question and explain your exceptional situation in the text box.<br><br>Do the authors confirm that all data underlying the findings described in their manuscript are fully available without restriction?	
**Describe where the data may be found in full sentences. If you are copying our sample text, replace any instances of XXX with the appropriate details.**<br><br>• If the data are **held or will be held in a public repository**, include URLs, accession numbers or DOIs. If this information will only be available after acceptance, indicate this by ticking the box below. For example: *All XXX files are available from the XXX database (accession number(s) XXX, XXX.*).<br>• If the data are all contained **within the manuscript and/or Supporting Information files**, enter the following: *All relevant data are within the manuscript and its Supporting Information files.*<br>• If neither of these applies but you are able to provide **details of access elsewhere**, with or without limitations, please do so. For example:<br><br>*Data cannot be shared publicly because of [XXX]. Data are available from the XXX Institutional Data Access / Ethics Committee (contact via XXX) for researchers who meet the criteria for access to confidential data.*<br><br>*The data underlying the results presented in the study are available from (include the name of the third party*	The datasets presented in this study can be found in online repositories. The names of the repository/repositories and accession number(s) can be found at https://www.ncbi.nlm.nih.gov/genbank/ , OL551674, SAMN23426491, SRR18344380.

and contact information or URL).<br>• This text is appropriate if the data are owned by a third party and authors do not have permission to share the data.<br><br>* typeset	
Additional data availability information:	Tick here if the URLs/accession numbers/DOIs will be available only after acceptance of the manuscript for publication so that we can ensure their inclusion before publication.

Manuscript Click here to access/download;Manuscript;VICTORIA-BLANCO ET AL Plos.docx

Characterization of Enterobacter phage vB_EcRAM-01, a new *Pseudotevenvirus* against *Enterobacter cloacae*, isolated in an urban river in Panama.

Ednner E. Victoria-Blanco[1,2,7,†], Jean Pierre González-Gómez[4,†], Juan Raúl Medina-Sánchez[2], Alexander A. Martínez[1,6,7], Nohelia Castro del Campo[4], Cristóbal Chaidez-Quiróz[4], Jordi Querol-Audi[1,2,5,7,§], Alex Omar Martínez-Torres[2,3,7,§]

1Programa de Maestría en Ciencias Biomédicas, Facultad de Medicina, Universidad de Panamá, Panamá.

2Laboratorio de Microbiología Experimental y Aplicada y Microbiología de Aguas (LAMEXA-LAMA), Universidad de Panamá, Panamá.

3Departamento de Microbiología y Parasitología, Facultad de Ciencias Naturales, Exactas y Tecnología, Universidad de Panamá, Panamá.

4Laboratorio Nacional para la Investigación en Inocuidad Alimentaria (LANIIA), Centro de Investigación en Alimentación y Desarrollo, A.C. (CIAD), Culiacán, Sinaloa, México.

5Departamento de Bioquímica y Nutrición, Facultad de Medicina, Universidad de Panamá, Panamá.

6Genomics and Proteomics Research Department, Gorgas Memorial Institute of Health Studies, Panama, Panama.

7Sistema Nacional de Investigación (SNI). Secretaría Nacional de Ciencia, Tecnología e Innovación (SENACYT), Panamá.

†Both authors contributed equally to this work and share first authorship.

*** Correspondence:**
[§]Corresponding author: Alex Omar Martínez-Torres: alex.martinez@ up.ac.pa and Jordi Querol-Audi: jordi.querol@up.ac.pa.

Bacteriophage, *E. cloacae complex*, phage therapy, molecular characterization, whole genome sequencing, "de novo" assembly.

Abstract

The *Enterobacter cloacae* complex, a prominent bacterium responsible worldwide for most bloodstream infections in the hospital environment, has shown broad-spectrum antibiotic resistance, including carbapenems. Therefore, bacteriophages have again attracted the attention of the science and medical community as an alternative to control MDR bacteria. In this study, water samples from Río Abajo River, in Panama City, Panama, were collected, for phage isolation, purification, characterization and propagation against the *E. cloacae* complex. As result, a phage produced clear and round plaque-forming units indicating a lytic phage was isolated. Further analyses concluded that this phage is stable at temperatures between 25°C and 50°C, it remains infective in a pH range between 7 to 11, with high sensitivity to UV light. Remarkedly, it exhibits a narrow host specificity only infecting *E. cloacae*. Whole genome sequencing revealed that is a myovirus with a genome size of 178,477 bp, a G-C content of 45.8%, and containing approximately 294 genes. Among them, protein-encoding genes involved in morphology, inactivation, adsorption to cells, DNA injection and lytic enzymes were identified. Additionally, the genome contained two tRNA sequences. Genes that encode holins and endolysins, typical of lytic bacteriophages, were also present. A whole-genome sequencing

analysis indicated that, according to the genus demarcation criteria, this phage belongs to a novel species within the Family *Straboviridae*, called genus *Pseudotevenvirus*.

Introduction

Enterobacteriaceae are ubiquitous in nosocomial settings and can play important roles in the burden of disease in the urinary tract, lower respiratory tract, intra-abdominal organs, and skin infections. The most relevant members of this Gram-negative bacterial group are *Klebsiella pneumoniae*, *Escherichia coli*, and *Enterobacter* spp. The severity of the infections is intensified by the resistance level to most available antibiotics, including carbapenem and cephalosporins (1). Special attention to *Enterobacter* is due to the intrinsic resistance to ampicillin and broad-spectrum cephalosporin which reduces the alternatives for antibiotic treatments. *Enterobacter* belongs to the ESKAPE group (acronym for *Enterococcus faecium*, *Staphylococcus aureus*, *Klebsiella pneumoniae*, *Acinetobacter baumannii*, *Pseudomonas aeruginosa*, and *Enterobacter* spp), related worldwide to severe nosocomial infections with a highly increasing multi-drug resistance. A problem of epidemiological importance is the presence of AmpC-producing strains together with BLEE-encoding plasmids and colistin co-resistance in *E. cloacae* complex (ECC) strains (Liu et al., 2021; Davin-Regli et al., 2019; Mezzatesta et al., 2012). The continuous emergence of multi-drug resistant strains have hampered the effectiveness of antibiotic treatments. Therefore, one possible approach to address this problem is the use of bacteriophages. In itself, the magnitude of the impact of phage predation on pathogenic bacteria has been demonstrated. In recent years, phage therapy has been successfully applied to *E. coli* O157:H7, causative agent of hemorrhagic colitis and hemolytic uremic syndrome (4–6). The use of phages has also been studied in the treatment of peritoneal infections, otitis externa, in the eradication of *P. aeruginosa* in patients with cystic fibrosis (Morello *et al*., 2011; Debarbieux *et al*., 2010), in the treatment of urinary infections or osteomyelitis caused by *S. aureus* (9). The favorable effects of phage therapy may even exceed its antibacterial activity. A surprising number of data has demonstrated the *in vivo* activity of phages such as the adenovirus growth suppression mediated by bacteriophage T4 (10) or development inhibition of *P. aeruginosa* by phage Pf4 (11).

The principal advantage of employing phages in therapy and biological control lies in their specificity towards bacterial targets. Those that do not infect commensal bacteria can be selected, therefore posing no threat to humans and animals. Their widespread presence in nature, their versatility, and their inherent resilience have elevated their status from mere research organisms to potentially viable and indispensable tools in the battle against growing antimicrobial resistance. However, the transfer of resistance and virulence genes, and other genes associated with negative outcomes through bacteriophages, constituted to an entire research domain aimed at mitigating any potential adverse impacts (12,13). For these reasons, this study is aimed to biologically and genetically isolate and characterize a phage against the *E. cloacae* complex.

Materials and Methods

Bacterial Strains and Growth Conditions. The host strain ATCC 23355 of *E. cloacae* complex used for the isolation and proliferation of the phage was the same as previously reported (14). In addition, *S. aureus, E. coli, E. aerogenes, E. cloacae, and P. aeruginosa*, used for host range determination, were provided (in February and November, 2019) by the Human Microbiology Department, Faculty of Medicine, Universidad de Panamá; Hospital Santo Tomas, and Complejo Hospitalario Dr. Arnulfo Arias Madrid. The *E. cloacae* host strain, along with twenty-seven clinical isolates of *E. cloacae,* were tested for antibiotic susceptibility using the Vitek II Automated Microbiological System (BioMérieux, Marcy l'Étoile, France) (results shown in Supplementary Table 1). The breakpoints were applied according to the Clinical and Laboratory Standards Institute (CLSI) guidelines (15).

Bioethical Considerations. The Bioethics Committee of the Universidad de Panamá with the endorsement of the Dirección General de Salud Pública of the Ministerio de Salud (Ministry of Health), approved this study (Sequential: 617). All the bacterial isolates used in this study were kindly provided by the medical institutions mentioned above and authors did not participate in a clinical trial, or obtained samples for the purposes of this study, neither had access to any information that could identify individual participants during or after data collection.

Phage Isolation. The Enterobacter phage vB_EcRAM-01 was isolated from waters of Rio Abajo river in Panama City, Panamá (coordinates, 9°00′55.2″N 79°29′25.6″W), using *E. cloacae* strain ATCC 23355 as a propagation host, which was grown previously in tryptic soy broth at 37°C for 24 h. Phage isolation, propagation, and purification were performed using the soft-agar overlay method (Victoria-Blanco *et al*., 2022).

Host Range of vB_EcRAM-01 against Clinical *E. Cloacae* Isolates: Spot Test and Efficiency of Plating (EOP). Clinical isolates of *E. cloacae, S. aureus, E. coli, P. aeruginosa*, and *E. aerogenes*, along with *E. cloacae* and *E. aerogenes* ATCC reference strains, were cultured until reaching the exponential growth phase (OD_{600nm} 0.6). Subsequently, 200 - 500 µL of each culture were mixed with 3 mL of top agar containing 10 mM $CaCl_2$ and plated onto a 1.5% nutrient agar plate. After solidification, a single droplet (10 µL) from each phage dilution (10^{-1} to 10^{-8}) was applied onto the agar (16).The droplets were allowed to dry for 10 min and plates were incubated at 37°C for 24 h - 48 h. A phage titer in PFU/mL was calculated for each strain tested (Stephenson, 2010). Phage virulence was assessed using the efficiency of the plating (EOP) method. The EOP was determined by dividing the phage titer at the final dilution on the test strain by the phage titer on its original isolation strain. Based on these calculations, phages were categorized into highly virulent (0.1 < EOP > 1.00), avirulent but still active (EOP < 0.001), or completely avirulent (no plaques were observed).

Effects of UV Light, Temperature and pH on the Viability of Phage vB_EcRAM-01. Purified vB_EcRAM-01 was exposed to different physicochemical conditions (UV light, temperature and pH) Suspensions were exposed to ultraviolet light at a distance of 50 cm for 10, 20, 40, 60, 70, and 90 s. To determine the effect of temperature, phage suspensions were placed at 25ºC, 50°C, 70ºC and 80ºC, respectively. pH stability was determined by mixing assay vB_EcRAM-01 phage samples resuspended in SM-gelatine buffer with buffer solutions ranging from pH 2 to pH 14. Phage viability was determined by counting the number of lysis PFU using the double-layer agar technique and the experiments were carried out in triplicates (18,19).

Bacteriophage DNA Isolation and Genome Sequencing. The QIAamp MinElute Virus Spin kit (QIAGEN, Valencia, CA) was used to extract DNA, according to the manufacturer´s instructions. The purity was assessed spectrophotometrically at 260 and 280 nm, using a BioSpec-nano Micro-volume UV-Vis Spectrophotometer (Shimadzu, Kyoto, Japan).

The DNA libraries were prepared using the Nextera XT DNA Library Preparation Kit (Illumina, San Diego, CA, USA) through fragmentation, PCR amplification, PCR cleanup, and library normalization. The genome was sequenced using the Illumina MiSeq platform (2 x 250 bp paired-end reads). Raw reads (629,372 reads in total) were checked for quality using FastQC (www.bioinformatics.Babraham.ac.UK/projects/fastqc) and trimmed using Timmomatic v0.39 (20). The genome was sequenced at the Genomics and Proteomics Research Department of ICGES using the MiSeq Illumina sequencer (2 x 250 bp paired-end reads) (ThermoFisher Scientific Waltham, Massachusetts USA).

Genome Annotations, Comparative Genomics and Phylogenetic Analyses. The genome was assembled through SPAdes v3.15.1 (21), resulting in a single contig with 8.96-fold coverage (21). We mapped the reads against the contig obtained using Bowtie v0.9.6 (http://bowtie-bio.sourceforge.net/index.shtml) to corroborate the sequence ends (22). The phage genome annotation was performed using RASTk v2.0 (23–25). Gene calling was performed through GeneMarkS v4.28

(http://exon.gatech.edu/GeneMark/genemarks.cgi) (26), and Glimmer v3.02 (http://ccb.jhu.edu/software/glimmer/index.shtml) (27,28).

Several methods were used to annotate the ORF functions, including the protein basic local alignment search tool (blastp) on the NCBI server (https://blast.ncbi.nlm.nih.gov/Blast.cgi) based on the search of the non-redundant protein sequence database (29), Hhpred using structural/domain database (https://toolkit.tuebingen.mpg.de/tools/hhpred) (30–33) and HMMER v2.41.1 through HMM database (34,35). (https://toolkit.tuebingen.mpg.de/tools/hhpred). Using a non-redundant protein matrix, employing Markov hidden model profiles (36), the annotations were manually curated using the Geneious v9.1.8 platform employing default parameters. Identification of transfer RNAs was performed using tRNA scan-SE v2.07 (http://lowelab.ucsc.edu/tRNAscan-SE/) and ARAGORN v1.2.41 (37) software (http://www.ansikte.se/ARAGORN/). Comparative analysis between genomes was performed using Mauve progressive alignment v2.3.1 (38) (http://darlinglab.org/mauve/mauve.html) employing default parameters.

Pairwise comparison of nucleotide sequences was performed using the Genome-BLAST Distance Phylogeny (GBDP) method of the VICTOR (Virus Classification and Tree Building Online Resource) platform (39) (https://ggdc.dsmz.de/victor.php), in the recommended environments for prokaryotic viruses (39). The resulting intergenomic distances were used to infer a branch-supported balanced minimum evolution tree using FASTME, including SPR post-processing (40) using D6 formula. Branch support was inferred from 100 pseudo-bootstrap replicates each. Trees were rooted at the midpoint (41), visualized using FigTree; personalized with taxon boundaries at species, genus, and family scales; and estimated with the iTOL program (42) (https://itol.embl.de/). The recommended clustering threshold is thirthy nine (39) and an F-value (fraction of links required for cluster fusion) of 0.5 (Meier-Kolthoff *et al*., 2014).

Determination of taxonomy and viral replication cycle type was achieved using PATRIC v3.6.9 (43) (https://patricbrc.org/) and PhageAI (https://phage.ai/) server (Tynecki *et al*., 2020). The genome comparison between Enterobacter phage vB_EcRAM-01 and close relatives was performed using EasyFig v2.2.2 (Sullivan *et al*., 2011). At the end, the intergenomic similarities amongst complete viral genomes were calculated through the VIRIDIC web tool (Moraru *et al*., 2020) with blastn default settings.

Statistical Analysis.

Statistically significant differences were determined using ANOVA. Differences in group means were compared using Tukey's HSD test. A P-value below 0.05 was considered statistically significant. Statistical analysis was conducted using R version 4.3.1(45).

Results

Bacteriophage Isolation and Host Range Analysis

In order to evaluate the host specificity of vB_EcRAM-01, a comprehensive assessment was conducted involving a total of thirty-three strains: two ATCC strains alongside a diverse selection of, both, Gram-negative and Gram-positive clinical isolates (Figure 1). Notably, vB_EcRAM-01 exhibited the capacity to infect all the *E. cloacae* isolates with varying degrees of efficacy while displaying negligible antimicrobial activity (efficiency < 0.10) against the other species tested, which included *E. coli*, *E. aerogenes*, *A. baumannii*, *S. aureus*, and *P. aeruginosa*. Upon infecting the reference strain, the clear plaque-forming units (PFUs), characterized by smooth perimeters, measured within the range of 3 mm to 7 mm in diameter, a hallmark indicative of its lytic phage attributes (Figure S1).

Environmental Stress Resistance: Stability Against UV Light, Temperature, pH.

Exposure to UV light (365 nm) decreased the infectivity of phage vB_ECRAM-01 over time. Results showed an exponential decay resulting in complete inactivation when incubated for more than one minute (Figure 2A).

The phage exhibited lytic activity when subjected to temperatures ranging from 25°C to 50°C, even after being incubated for 90 minutes (Figure 2B). However, when exposed to 70°C, the phage became completely inactivated after only 20 min of incubation and, at 80 C, it lost its infectivity entirely.
The stability of vB_EcRAM-01 was determined at various pH values. It maintained its infectivity in a pH range between pH 7.5 and pH 11. The best conditions were found between pH 9 and 10 (Figure 2C). Incubation under acidic pH resulted in a significant reduction of viral proliferation. The phage was not able to replicate in a pH greater than 11 or less than 3. Overall, the best activity was found in alkaline media.

Comparative Genomics and Phylogenetic Analysis

The genomic attributes of phage vB_EcRAM-01 were delineated as follows: the genome spans 178,477 base pairs, hosting a total of 293 genes. Among these, 86 genes were implicated in anticipated functions encompassing morphology, inactivation, cellular adsorption, DNA injection, and host lysis. Remarkably, the genome of vB_EcRAM-01 was lacking discernible genes associated with resistance, virulence, or allergenicity. The genomic blueprint encodes two transfer RNAs (tRNAs), one corresponding to methionine (cat) and the other to glycine (tcc). A pertinent insight is the substantial sequence identity—90.11%—shared with other phages (Figure 3) (14).
To assign a taxonomic classification to phage vB_EcRAM-01, a phylogenetic tree based on whole-genome was constructed, and intergenomic similarities were calculated between the closely related genomes reported in GenBank. The phylogenetic tree and intergenomic similarity heatmap revealed that phage vB_EcRAM-01 is a new member of the *Pseudotevenvirus* genus within the *Straboviridae* family.
The GBDP phylogenetic tree using the D6 formula with an average support of 31%, identifying ten and nine clusters, respectively, at the genus level. At the family level, there were five and six clusters, respectively (Figure 4).
Nucleotide identity analysis (ANI) was done on the 28 genomes with the highest homology in the GenBank database using VIRIDIC (46). According to the results of the heat mapping, phage vB_EcRAM-01 shares less than 95% nucleotide identity with previously reported genomes, thereby establishing it as a novel species within the Family *Straboviridae*, genus *Pseudotevenvirus*, according to the genus demarcation criteria (>70% nucleotide identity over the entire genome length) recommended by the ICTV (Figure 5).
Comparative BLASTn analysis of the complete genome of vB_EcRAM-01 against phages in the GenBank database revealed that vB_EcRAM-01 has a high degree of coverage and identity with the Enterobacter phages ENC22 (OL355128.1), ENC7 (OL355125.1), ENC25 (OL355127.1), EBPL (MT341500.1). In VIRIDIC analysis, the phages shared collinear gene blocks that are identical in length between the two genomes. In comparison, other blocks revealed a high degree of similarity between this phage and others, including Cronobacter phage vB_CsaM IeE (NC_048646.1), Cronobacter phage vB_CsaM IeN (KX431560.1), Citrobacter phage Margaery (KT381880.1), and myoviral members of the subfamily *Tevenvirinae*.

Discussion

In Panama, seven out of the eight bacterial pathogens responsible for 80% of the reported infections concerning the emergence of resistance in a hospital level (ESKAPE) has been identified: *S. aureus* (16%), *Enterococcus* spp (14.4%), *E. coli* (12%), coagulase-negative *Staphylococcus* (11%), *K. pneumoniae* and *K. oxytoca* (8%), *P. aeruginosa* (8%), and *Enterobacter* spp. (5%). Several microorganisms developed antibiotic resistance to the more common antibiotics to treat these bacterial infections like piperacillin-tazobactam, cephalosporins, nitrofurans, and carbapenems (47,48).
Therefore, in comparison to the usage of antibiotics, phage therapy is a viable option. While scientific publications on phage isolation have increased in recent years, only a small number of phages have been commercialized (49). One of the primary reasons is a lack of adequate characterization, which is

required to detect and mitigate the hazards associated with phage therapy (Abedon, 2018b; Hyman and Abedon, 2019). Moreover, the biological characteristics of a phage determines its stability and therefore, its optimum administration forms (50).

As a first step, vB_EcRAM-01was subjected to environmental stress tests, such as temperature, UV radiation and pH, factors that play a role in phage survival (51,52). The results indicate that vB_EcRAM-01 is stable at temperatures ranging from 25°C to 50°C, although its replication rate is highest at 37°C. This aligns with findings observed in Myoviridae phages (53,54) exhibiting a comparable stability profile within a broad temperature range. However, it deviates from the EBP phage, known for its resilience across a wider temperature span (55). Notably, inactivation under UV light transpires rapidly, underscoring the necessity of safeguarding possible clinical applications from sunlight exposure. Interestingly, vB_EcRAM-01 maintains its infectivity across expansive pH and temperature ranges, implying potential robustness within the human body. Enhancements through administration systems designed to shield the phage from gastrointestinal pH could potentially further increase its utility and stability in therapeutic applications (56).

The primary objective of this study was to investigate the host specificity (host range) of phage vB_EcRAM-01 concerning isolates within the *E. cloacae* complex. We hypothesized that vB_ECRAM-01 would exhibit a broad tropism, potentially affecting various bacterial species, regardless of the presence or absence of receptor molecules or intracellular restriction mechanisms. Accurate identification of the host bacteria's specificity for bacteriophage vB_EcRAM-01 is crucial for a comprehensive characterization of this bacteriophage in order to evaluate a potential therapeutic use. Our observations indicate that this bacteriophage displayed reactivity mainly with its host strain. These results are in line with findings reported for phages TSE1, TSE2, and TSE3 (57). *E. coli* displayed some sensitivity but did not exhibit complete lysis. Exceptions to this pattern were observed with myophages ENC22, ENC7, and ENC25, which demonstrated the ability to infect diverse *Enterobacter* species (58).

Apart from meeting the requirements of being innocuous to normal microbiota, therapeutic use involves the employment of fully lytic phages that lack undesired virulence factor genes or antibiotic resistance genes (59). The molecular investigation of phage vB_EcRAM-01 confirmed that it was a strictly lytic phage. The genome contained genes coding for holins and endolysins. However, it lacked genes associated with lysogeny, such as integrases, transposons, and excitons (60).

The evolutionary context designated it as a T4-type myophage spanning a genome length of 178,477 base pairs. Computational analysis further classified it as a viral entity within the Class *Caudoviricetes*, *Strabovviridae* family, and *Pseudotevenvirus* genus (60).

By scanning the GenBank database for homologous sequences, a 92% sequence homology with myophage EBPL was observed, a phage that also infects *E. cloacae* (55), and 99.16% sequence homology with myophages ENC7, ENC25, and ENC22 (58).

According to genotypic characteristics (61), vB_EcRAM-01, like an EBP phage, is a *Myoviridae* phage (55). The vB_EcRAM-01 virus was termed a bacterial infecting virus isolated from Rio Abajo waters that suppressed *E. cloacae*, a myophage, according to the informal phage nomenclature criteria (62–64).

In this study, the genome-wide syntenic analysis indicated that both phages had comparable rearrangements but differed slightly in nucleotide sequence. Additionally, a phylogenetic study of whole-genome sequences showed that both phages diverged from a common ancestor but experienced many nucleotides substitution events since then (65). Given that EBPL was previously described as a singleton phage in the myophage phylogenetic tree (66), phylogenetic and nucleotide identity analyses indicated that vB_EcRAM-01 might represent a previously unreported species.

Therefore, vB_EcRAM-01 is physiologically and molecularly stable under a wide temperature and pH range. However, it has a restricted host range. Although it had intense lytic activity against the *E. cloacae* complex, isolation was deemed necessary for more efficient eradication.

In Conclusion, this study identified a lytic bacteriophage. The characterization enhances comprehension of this novel phage, and through its phylogenetic relatives, it may exhibit a myovirus-like morphology, as demonstrated by insights from next-generation sequencing. This sequencing elucidates that vB_EcRAM-01 aligns with the Myoviridae family in accordance with the conventional taxonomic classification (67). According to the phylogenetic analysis previously described, this virus is a new member of the family *Straboviridae* and the genus *Pseudotevenvirus* according to the new classification (68). The vB_EcRAM-01 strain is also a viable candidate for many clinical applications, including phage therapy and biologic control or decontamination, due to its host specificity, the genetic link, and lack of lysogenic genes.

DATA AVAILABILITY STATEMENT

The datasets presented in this study can be found in online repositories. The names of the repository/repositories and accession number(s) can be found at https://www.ncbi.nlm.nih.gov/genbank/ , OL551674, SAMN23426491, SRR18344380.

AUTHOR CONTRIBUTIONS

EEVB and JPGG drafted the manuscript, while JRMS conducted the statistical analysis of the results. NCC and CCHQ, along with JQA and AOMT, contributed to data analysis and manuscript revisions. EEVB, JQA, and AOMT secured project funding. All authors reviewed and approved the submitted manuscript.

ACKNOWLEDGMENTS

This work was funded in part by the Sistema Nacional de Investigación (SNI) of the Secretaría Nacional de Ciencia, Tecnología e Innovación (SENACYT, Panama), through grants 87-2019, 81-2019 and FID18-044, and also from the Water, Protected Areas and Wildlife Trust of the Ministry of Environment of Panama through grant 026-45-2019 and grant 111130406 from the Ministry of Economy and Finance of Panama. Clinical bacterial strains were provided by José E. Moreno, MSc., of the Microbiology Division of Laboratorio Central de Referencia en Salud Pública, Instituto Conmemorativo Gorgas de Estudios de Salud (ICGES) and the Department of Microbiology, Hospital Santo Tomás.
Additionally, we would like to thank Claudia González Msc., Jessica Gondola, Ambar Moreno, and Oris Chavarria of the Research Department of Genomics and Proteomics of ICGES for providing us with NGS Illumina sequencing. Furthermore, we would like to express our gratitude to Leandra Gómez, Msc. of Department of Pharmacology at the Faculty of Medicine, Universidad de Panama, for her invaluable contribution in reviewing the bioethical considerations within this research. Also Bs. Tatiana A. Martínez González for her contribution to the English revision of the present work.

CONFLICT OF INTEREST

The authors declare that the research was conducted in the absence of any commercial or financial relationships that could be construed as a potential conflict of interest.

PUBLISHER’S NOTE

All claims expressed in this article are solely those of the authors and do not necessarily represent those of their affiliated organizations, or those of the publisher, the editors and the reviewers. Any product that may be evaluated in this article, or claim that may be made by its manufacturer, is not guaranteed or endorsed by the publisher.

REFERENCES

1. WHO. Global priority list of antibiotic-resistant bacteria to guide research, discovery, and development of new antibiotics. 2017.

2. Davin-Regli A, Lavigne JP, Pagès JM. Enterobacter spp.: Update on Taxonomy, Clinical Aspects, and Emerging Antimicrobial Resistance. Clin Microbiol Rev [Internet]. 2019 Jul 17 [cited 2021 Jun 14];32(4). Available from: https://www.ncbi.nlm.nih.gov/pmc/articles/PMC6750132/

3. Mezzatesta ML, Gona F, Stefani S. *Enterobacter cloacae* complex: clinical impact and emerging antibiotic resistance. Future Microbiology. 2012 Jul;7(7):887–902.

4. Amarillas L, Rubí-Rangel L, Chaidez C, González-Robles A, Lightbourn-Rojas L, León-Félix J. Isolation and Characterization of phiLLS, a Novel Phage with Potential Biocontrol Agent against Multidrug-Resistant Escherichia coli. Frontiers in Microbiology [Internet]. 2017 [cited 2022 Nov 2];8. Available from: https://www.frontiersin.org/articles/10.3389/fmicb.2017.01355

5. López-Cuevas O, Castro-Del Campo N, León-Félix J, Valdez-Torres B, Chaidez C. Evaluation of Bacteriophage Av-08 for Simultaneous Biocontrol of Salmonella Montevideo and Escherichia Coli O157:h7 in Experimentally Contaminated Chicken Skin. Journal of Food Safety. 2012;32(3):305–10.

6. Sharma M, Patel JR, Conway WS, Ferguson S, Sulakvelidze A. Effectiveness of Bacteriophages in Reducing Escherichia coli O157:H7 on Fresh-Cut Cantaloupes and Lettuce. Journal of Food Protection. 2009 Jul;72(7):1481–5.

7. Debarbieux L, Leduc D, Maura D, Morello E, Criscuolo A, Grossi O, et al. Bacteriophages Can Treat and Prevent *Pseudomonas aeruginosa* Lung Infections. J INFECT DIS. 2010 Apr;201(7):1096–104.

8. Morello E, Saussereau E, Maura D, Huerre M, Touqui L, Debarbieux L. Pulmonary Bacteriophage Therapy on Pseudomonas aeruginosa Cystic Fibrosis Strains: First Steps Towards Treatment and Prevention. Aziz R, editor. PLoS ONE. 2011 Feb 15;6(2):e16963.

9. Abedon ST. Commentary: Phage Therapy of Staphylococcal Chronic Osteomyelitis in Experimental Animal Model. Front Microbiol [Internet]. 2016 Aug 10 [cited 2019 Jul 1];7. Available from: http://journal.frontiersin.org/Article/10.3389/fmicb.2016.01251/abstract

10. Przybylski M, Dzieciątkowski T, Borysowski J, Międzybrodzki R, Górski A. Inhibitory Effects of Bacteriophage Preparations on Adenoviral Replication. INT. 2019;62(1):37–44.

11. Casto A, Hurwitz A, Kou K, Mansour G, Policke R, Schmidt A, et al. Bacteriophages: The Answer to Antibiotic Resistance? James Madison Undergraduate Research Journal. 2016;3:36–41.

12. Hassan AY, Lin JT, Ricker N, Anany H. The Age of Phage: Friend or Foe in the New Dawn of Therapeutic and Biocontrol Applications? Pharmaceuticals. 2021 Mar;14(3):199.

13. Häusler T. Viruses vs. Superbugs [Internet]. Vol. 1. London: Palgrave Macmillan UK; 2006 [cited 2019 Jul 9]. Available from: http://link.springer.com/10.1007/978-0-230-55228-9

14. Victoria-Blanco EE, González-Gómez JP, Quiroz E, Martínez AA, González C, Castro del Campo N, et al. Complete Genome Sequence of Enterobacter Phage vB_EcRAM-01, a New

Pseudotevenvirus against the Enterobacter cloacae Complex. Microbiology Resource Announcements. 2022 May 10;1–3.

15. CLSI. CLSI Performance Standards for Antimicrobial Susceptibility Testing, CLSI Guideline M100. 28th ed. Wayne, PA: Clinical and Laboratory Standards Institute; 2018.

16. Khan Mirzaei M, Nilsson AS. Isolation of Phages for Phage Therapy: A Comparison of Spot Tests and Efficiency of Plating Analyses for Determination of Host Range and Efficacy. Schuch R, editor. PLoS ONE. 2015 Mar 11;10(3):e0118557.

17. Stephenson, Frank H. Calculations for Molecular Biology and Biotechnology [Internet]. second edition. Vol. 1. USA: Elsevier/AP, Academic Press is an imprint of Elsevier; 2010. Available from: Vetbiotech.um.ac.ir

18. Flores V. Caracterización de bacteriófagos líticos de Salmonella enterica aislados de muestras de pollos. [Lima Peru]: Universidad Mayor San Marcos; 2017.

19. Zaburlin D, Quiberoni A, Mercanti D. Changes in Environmental Conditions Modify Infection Kinetics of Dairy Phages. Food Environ Virol. 2017 Sep;9(3):270–6.

20. Bolger AM, Lohse M, Usadel B. Trimmomatic: a flexible trimmer for Illumina sequence data. Bioinformatics. 2014 Aug 1;30(15):2114–20.

21. Bankevich A, Nurk S, Antipov D, Gurevich AA, Dvorkin M, Kulikov AS, et al. SPAdes: A New Genome Assembly Algorithm and Its Applications to Single-Cell Sequencing. Journal of Computational Biology. 2012 Apr 16;19(5):455–77.

22. Langmead B, Trapnell C, Pop M, Salzberg SL. Ultrafast and memory-efficient alignment of short DNA sequences to the human genome. Genome Biology. 2009 Mar 4;10(3):R25.

23. Aziz RK, Bartels D, Best AA, DeJongh M, Disz T, Edwards RA, et al. The RAST Server: rapid annotations using subsystems technology. BMC Genomics. 2008 Feb 8;9:75.

24. Brettin T, Davis JJ, Disz T, Edwards RA, Gerdes S, Olsen GJ, et al. RASTtk: a modular and extensible implementation of the RAST algorithm for building custom annotation pipelines and annotating batches of genomes. Sci Rep. 2015 Feb 10;5:8365.

25. Overbeek R, Olson R, Pusch GD, Olsen GJ, Davis JJ, Disz T, et al. The SEED and the Rapid Annotation of microbial genomes using Subsystems Technology (RAST). Nucleic Acids Res. 2014 Jan;42(Database issue):D206-214.

26. Besemer J, Lomsadze A, Borodovsky M. GeneMarkS: a self-training method for prediction of gene starts in microbial genomes. Implications for finding sequence motifs in regulatory regions. Nucleic Acids Research. 2001 Jun 15;29(12):2607–18.

27. Delcher AL, Bratke KA, Powers EC, Salzberg SL. Identifying bacterial genes and endosymbiont DNA with Glimmer. Bioinformatics. 2007 Mar 15;23(6):673–9.

28. Salzberg SL, Delcher AL, Kasif S, White O. Microbial gene identification using interpolated Markov models. Nucleic Acids Res. 1998 Jan 15;26(2):544–8.

29. Altschul SF, Madden TL, Schäffer AA, Zhang J, Zhang Z, Miller W, et al. Gapped BLAST and PSI-BLAST: a new generation of protein database search programs. Nucleic Acids Res. 1997 Sep 1;25(17):3389–402.

30. Gabler F, Nam SZ, Till S, Mirdita M, Steinegger M, Söding J, et al. Protein Sequence Analysis Using the MPI Bioinformatics Toolkit. Current Protocols in Bioinformatics. 2020;72(1):e108.

31. Zimmermann L, Stephens A, Nam SZ, Rau D, Kübler J, Lozajic M, et al. A Completely Reimplemented MPI Bioinformatics Toolkit with a New HHpred Server at its Core. Journal of Molecular Biology. 2018 Jul 20;430(15):2237–43.

32. Meier A, Söding J. Automatic Prediction of Protein 3D Structures by Probabilistic Multi-template Homology Modeling. PLOS Computational Biology. 2015 Oct 23;11(10):e1004343.

33. Hildebrand A, Remmert M, Biegert A, Söding J. Fast and accurate automatic structure prediction with HHpred. Proteins: Structure, Function, and Bioinformatics. 2009;77(S9):128–32.

34. Potter SC, Luciani A, Eddy SR, Park Y, Lopez R, Finn RD. HMMER web server: 2018 update. Nucleic Acids Research. 2018 Jul 2;46(W1):W200–4.

35. Finn RD, Clements J, Arndt W, Miller BL, Wheeler TJ, Schreiber F, et al. HMMER web server: 2015 update. Nucleic Acids Research. 2015 Jul 1;43(W1):W30–8.

36. Hofmann P, Tashman Z. Hidden Markov Models and Their Application for Predicting Failure Events. In: Krzhizhanovskaya VV, Závodszky G, Lees MH, Dongarra JJ, Sloot PMA, Brissos S, et al., editors. Computational Science – ICCS 2020. Cham: Springer International Publishing; 2020. p. 464–77. (Lecture Notes in Computer Science).

37. Laslett D, Canback B. ARAGORN, a program to detect tRNA genes and tmRNA genes in nucleotide sequences. Nucleic Acids Res. 2004;32(1):11–6.

38. Darling ACE, Mau B, Blattner FR, Perna NT. Mauve: Multiple Alignment of Conserved Genomic Sequence With Rearrangements. Genome Res. 2004 Jan 7;14(7):1394–403.

39. Meier-Kolthoff JP, Göker M. VICTOR: genome-based phylogeny and classification of prokaryotic viruses. Bioinformatics. 2017 Nov 1;33(21):3396–404.

40. Lefort V, Desper R, Gascuel O. FastME 2.0: A Comprehensive, Accurate, and Fast Distance-Based Phylogeny Inference Program. Mol Biol Evol. 2015 Oct;32(10):2798–800.

41. Farris JS. Estimating Phylogenetic Trees from Distance Matrices. The American Naturalist. 1972;106(951):645–68.

42. Letunic I, Bork P. Interactive Tree Of Life (iTOL) v4: recent updates and new developments. Nucleic Acids Research. 2019 Jul 2;47(W1):W256–9.

43. Davis JJ, Wattam AR, Aziz RK, Brettin T, Butler R, Butler RM, et al. The PATRIC Bioinformatics Resource Center: expanding data and analysis capabilities. Nucleic Acids Res. 2020 Jan 8;48(D1):D606–12.

44. Tynecki P, Guziński A, Kazimierczak J, Jadczuk M, Dastych J, Onisko A. PhageAI - Bacteriophage Life Cycle Recognition with Machine Learning and Natural Language Processing. 2020 Jul 12 [cited 2021 Apr 11]; Available from: https://europepmc.org/article/ppr/ppr186662

45. R Core Team. R: A Language and Environment for Statistical Computing. Vienna, Austria: R Foundation for Statistical Computing; 2023.

46. Moraru C, Varsani A, Kropinski AM. VIRIDIC—A Novel Tool to Calculate the Intergenomic Similarities of Prokaryote-Infecting Viruses. Viruses. 2020 Nov;12(11):1268.

47. Moreno J. Antibiotic resistance profiles in Panama: Trends from 2007 to 2013. PPIJ [Internet]. 2018 Sep 7 [cited 2019 Jul 8];6(5). Available from: https://medcraveonline.com/PPIJ/PPIJ-06-00200.php

48. MINSA. 18400_2011_31657_INFORME RESISTENCIA ANTIBIOTICOS-USO RACIONAL - 036.pdf. Panama: ICGES; 2015 p. 48.

49. Abedon ST. Detection of Bacteriophages: Phage Plaques. In: Harper DR, Abedon ST, Burrowes BH, McConville ML, editors. Bacteriophages [Internet]. Cham: Springer International Publishing; 2018 [cited 2019 Jul 1]. p. 1–32. Available from: http://link.springer.com/10.1007/978-3-319-40598-8_16-1

50. Hyman P, Abedon ST. Bacteriophage: Overview. In: Reference Module in Biomedical Sciences [Internet]. Elsevier; 2019 [cited 2019 Jul 1]. p. B978012801238302506X. Available from: https://linkinghub.elsevier.com/retrieve/pii/B978012801238302506X

51. Phothichaisri W, Ounjai P, Phetruen T, Janvilisri T, Khunrae P, Singhakaew S, et al. Characterization of Bacteriophages Infecting Clinical Isolates of Clostridium difficile. Front Microbiol [Internet]. 2018 [cited 2019 Jul 14];9. Available from: https://www.frontiersin.org/articles/10.3389/fmicb.2018.01701/full#B51

52. Silva YJ, Costa L, Pereira C, Cunha Â, Calado R, Gomes NCM, et al. Influence of environmental variables in the efficiency of phage therapy in aquaculture. Microbial Biotechnology. 2014;7(5):401–13.

53. Litt PK, Jaroni D. International Journal of Microbiology. 2017 [cited 2019 Jul 9]. Isolation and Physiomorphological Characterization of Escherichia coli O157:H7-Infecting Bacteriophages Recovered from Beef Cattle Operations. Available from: https://www.hindawi.com/journals/ijmicro/2017/7013236/

54. Yamaki S, Kawai Y, Yamazaki K. Characterization of a novel bacteriophage, Phda1, infecting the histamine-producing Photobacterium damselae subsp. damselae. Journal of Applied Microbiology. 2015;118(6):1541–50.

55. Asif M, Naseem H, Alvi IA, Basit A, Rehman S ur. Characterization of a lytic EBP bacteriophage with large size genome against Enterobacter cloacae. APMIS. 2021;129(8):461–9.

56. Dąbrowska K. Phage therapy: What factors shape phage pharmacokinetics and bioavailability? Systematic and critical review. Med Res Rev. 2019 Sep;39(5):2000–25.

57. Khawaja KA, Abbas Z, Rehman S ur. Isolation and characterization of lytic phages TSE1-3 against Enterobacter cloacae. Open Life Sciences [Internet]. 2016 Jan 1 [cited 2019 Jul 5];11(1). Available from: https://www.degruyter.com/view/j/biol.2016.11.issue-1/biol-2016-0038/biol-2016-0038.xml

58. Finney AG, Perry JM, Evans DR, Westbrook KJ, McElheny CL, Iovleva A, et al. Isolation and Characterization of Lytic Bacteriophages Targeting Diverse Enterobacter spp. Clinical Isolates. PHAGE. 2022 Mar;3(1):50–8.

59. Soohey RL. Identifyng Genes Essential For Lysogeny in Cluster e Mycobacteriophage Ukulele. [Maine, Fl]: The University of Maine; 2017.

60. Ríos-Sandoval M. Caracterización Genómica, Morfológica y Replicativa del Bacteriófago ΦXaF18 de Xanthomonas vesicatoria. [Zapopan, Jalisco]: Centro De Investigación Y Asistencia En Tecnología Y Diseño Del Estado De Jalisco, A. C.; 2019.

61. Hatfull GF. Bacteriophage genomics. Current Opinion in Microbiology. 2008 Oct;11(5):447–53.

62. Turner D, Kropinski AM, Adriaenssens EM. A Roadmap for Genome-Based Phage Taxonomy. Viruses. 2021 Mar;13(3):506.

63. Adriaenssens EM, Sullivan MB, Knezevic P, van Zyl LJ, Sarkar BL, Dutilh BE, et al. Taxonomy of prokaryotic viruses: 2018-2019update from the ICTV Bacterial and Archaeal Viruses Subcommittee. Arch Virol. 2020 May 1;165(5):1253–60.

64. Adriaenssens E, Brister JR. How to Name and Classify Your Phage: An Informal Guide. Viruses. 2017 Apr 3;9(4):70.

65. Leskinen K, Blasdel BG, Lavigne R, Skurnik M. RNA-Sequencing Reveals the Progression of Phage-Host Interactions between φR1-37 and Yersinia enterocolitica. Viruses. 2016 Apr 22;8(4):111.

66. Jacquemot L, Bettarel Y, Monjol J, Corre E, Halary S, Desnues C, et al. Therapeutic Potential of a New Jumbo Phage That Infects Vibrio coralliilyticus, a Widespread Coral Pathogen. Front Microbiol [Internet]. 2018 [cited 2021 Jul 29];0. Available from: https://www.frontiersin.org/articles/10.3389/fmicb.2018.02501/full

67. Adams MJ, Lefkowitz EJ, King AMQ, Harrach B, Harrison RL, Knowles NJ, et al. 50 years of the International Committee on Taxonomy of Viruses: progress and prospects. Arch Virol. 2017 May;162(5):1441–6.

68. Turner D, Shkoporov AN, Lood C, Millard AD, Dutilh BE, Alfenas-Zerbini P, et al. Abolishment of morphology-based taxa and change to binomial species names: 2022 taxonomy update of the ICTV bacterial viruses subcommittee. Arch Virol. 2023 Jan 23;168(2):74.

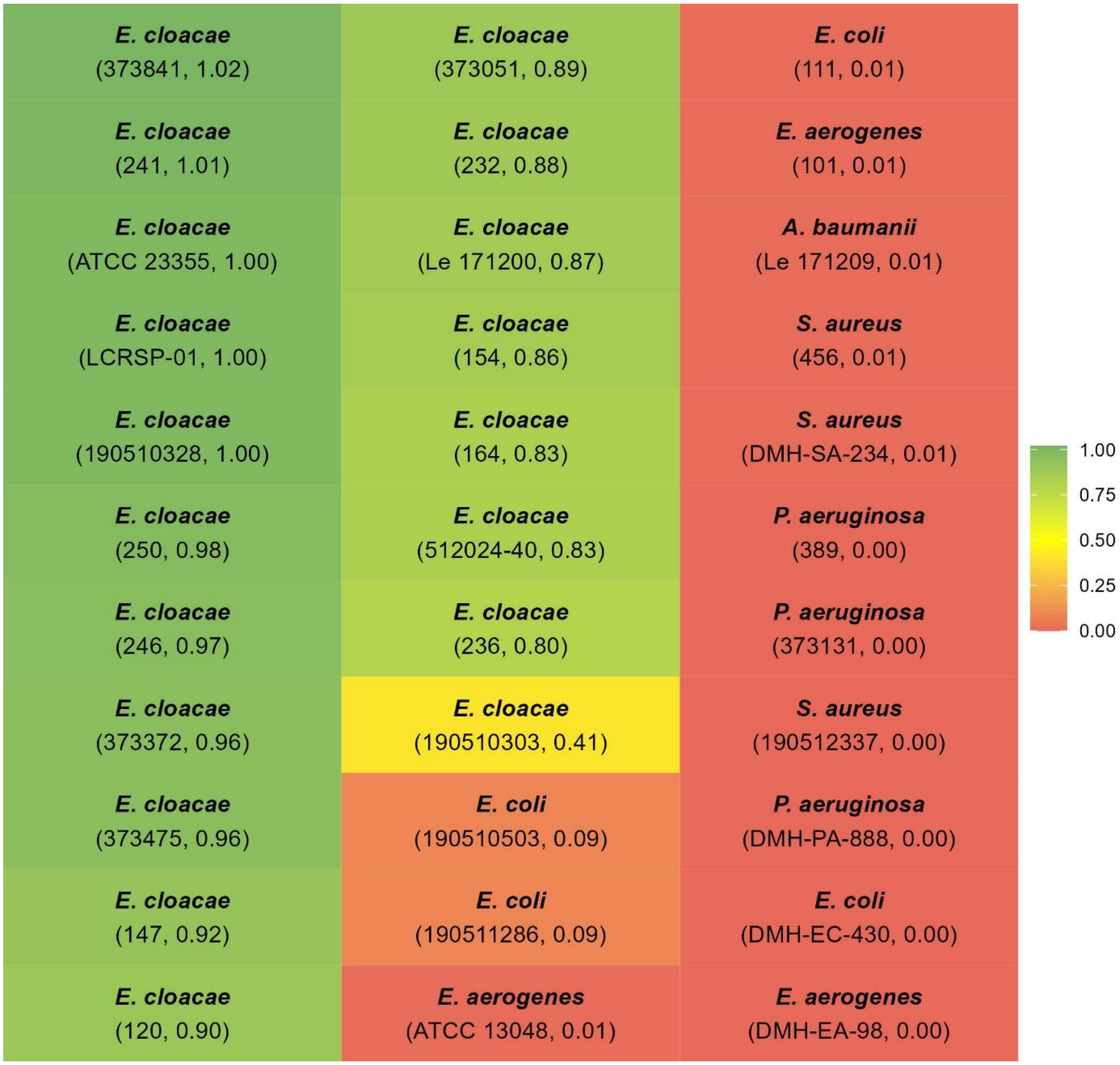

Figure 1. Host range specificity and efficiency of plating. The strain code followed by the efficiency value are shown in parentheses. Color range code associated with EOP is indicated on the right.

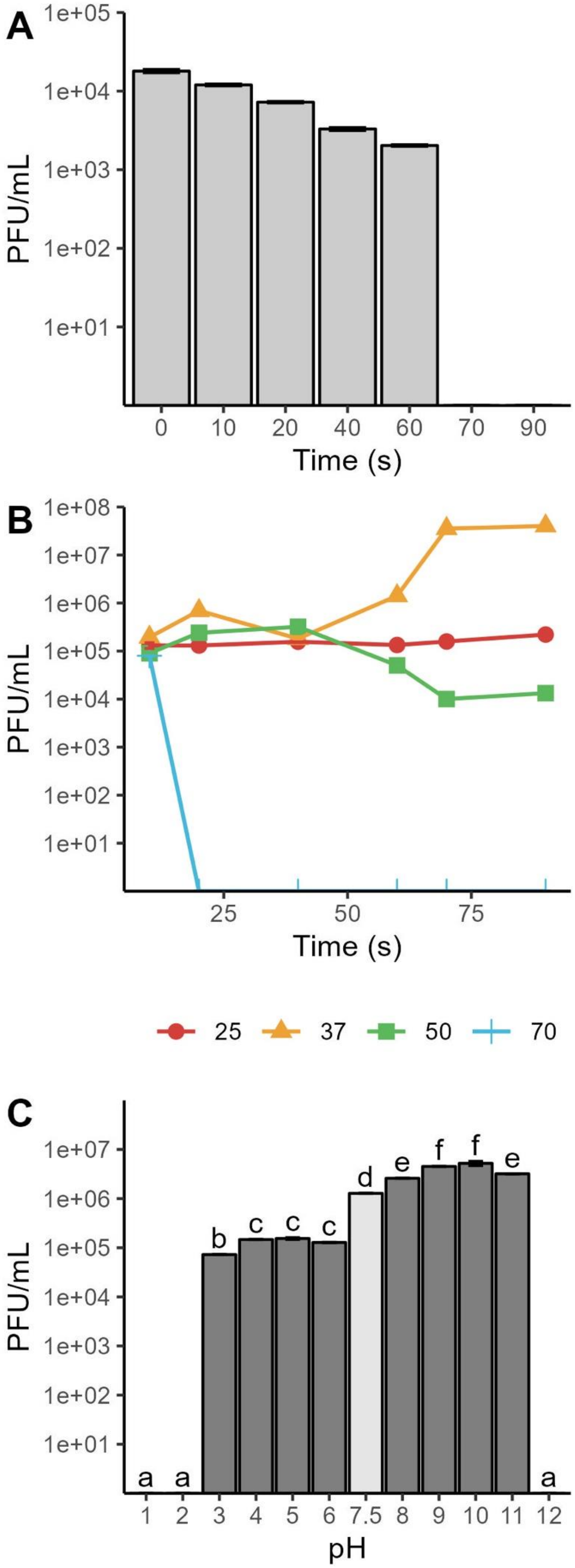

A
1e+05
1e+04
1e+03
1e+02
1e+01
PFU/mL
0
10
20
40
60
70
90
Time (s)
B
1e+08
1e+07
1e+06
1e+05
1e+04
1e+03
1e+02
1e+01
PFU/mL
25
50
75
Time (s)
25
37
50
70
C
1e+07
1e+06
1e+05
1e+04
1e+03
1e+02
1e+01
PFU/mL
a a b c c c d e f f e a
1 2 3 4 5 6 7.5 8 9 10 11 12
pH

507

Figure 2. Stability of vB_ECRAM-01 to (A) UV radiation, (B) temperature, and (C) pH. Data is expressed as mean ± standard error. Treatments with the same letters in panel (C) are not significantly different (Tukey's HSD test, p>0.05).

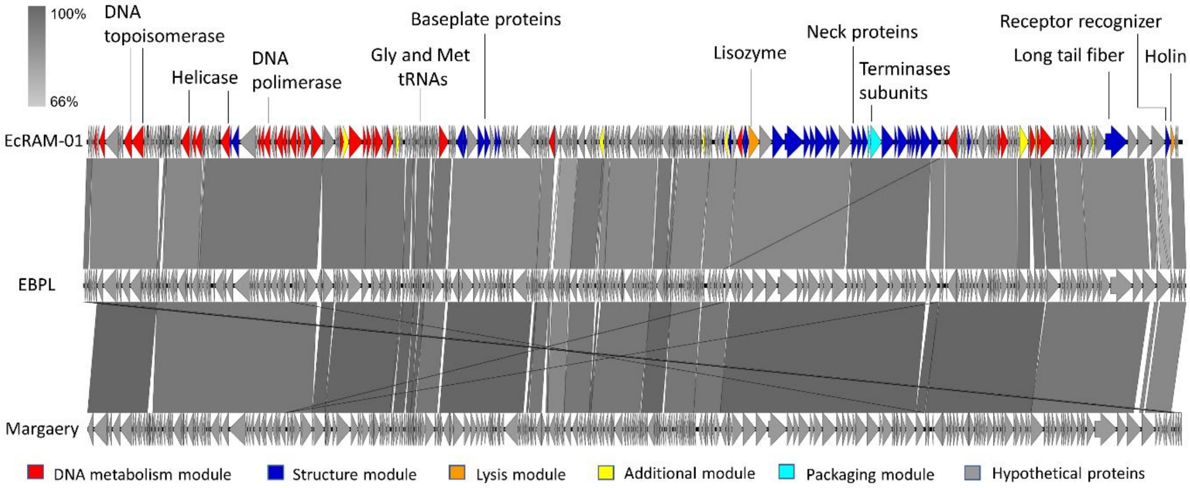

Figure 3. Comparison of Enterobacter phage vB_EcRAM-01 genome with close relatives. Color scales represent the percentage of nucleotide identity between regions obtained by blastn.

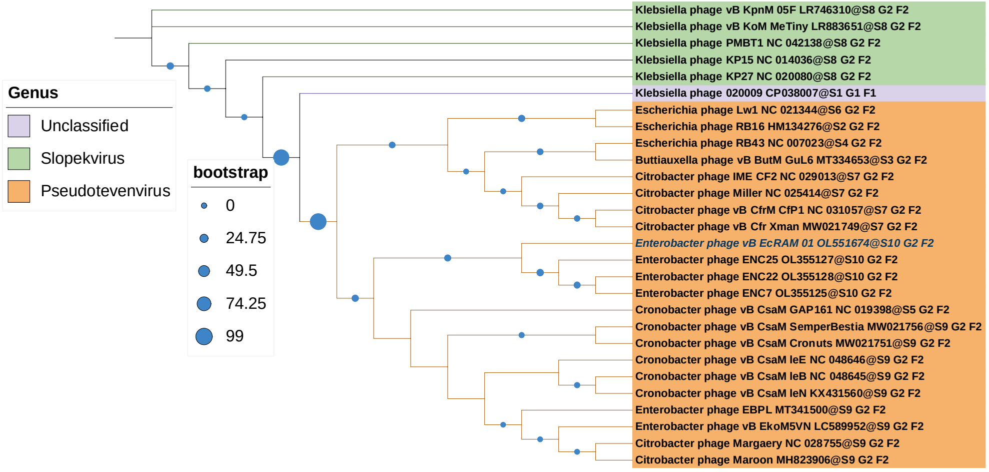

Figure 4. Phage vB_EcRAM-01 genomic and phylogenetic analyses. Genome Blast Distance Phylogeny (GBDP)-based phylogenetic tree of vB_EcRAM-01 and 28 related *Straboviridae* members

created by iTOL v6.0 platform. The Enterobacter phage vB_EcRAM-01 belongs to the genus *Pseudotevenvirus* and is a new phage species.

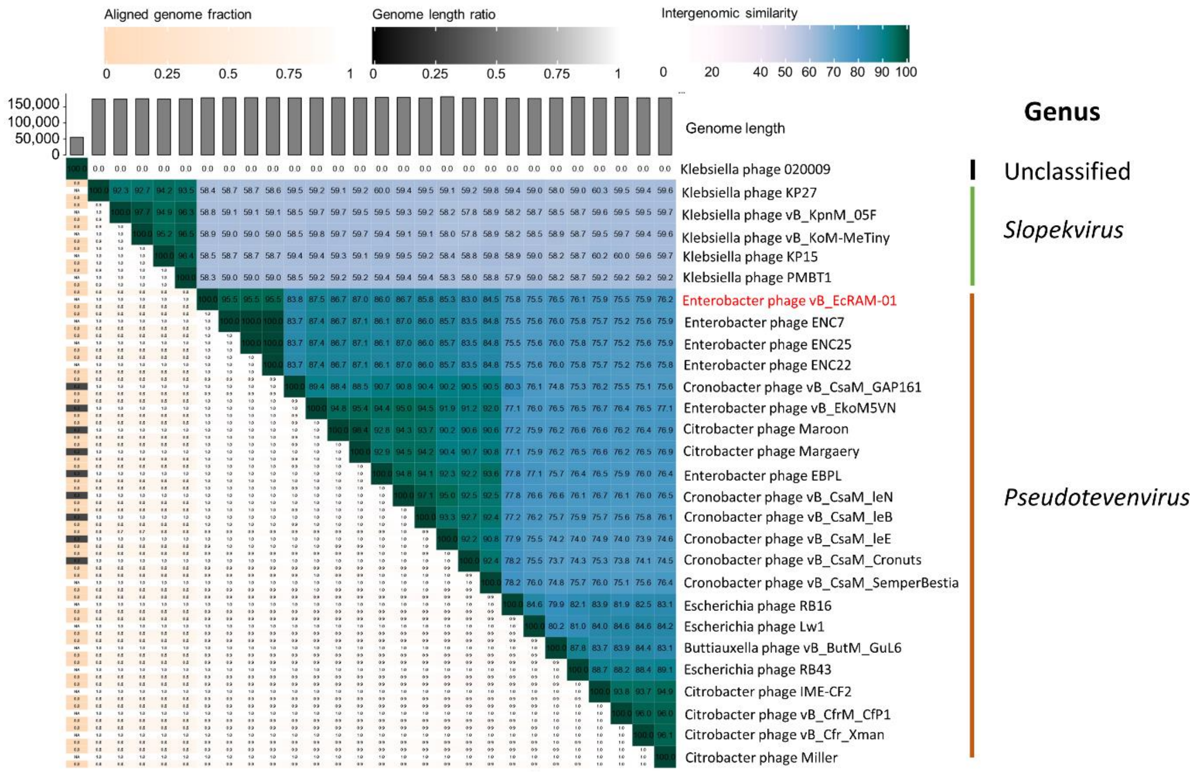

Figure 5. Taxonomic classification of Enterobacter phage vB_EcRAM-01. A heatmap created by VIRIDIC based on intergenomic similarities between viral genomes, with the proportion of similarity shown by the color scale.

Manuscript

Click here to access/download;Manuscript;Victoria-Blanco et al Revised Manuscript 220324.docx

Characterization of Enterobacter phage vB_EcRAM-01, a new *Pseudotevenvirus* against *Enterobacter cloacae*, isolated in an urban river in Panama.

Ednner E. Victoria-Blanco[1,2,7,¶], Jean Pierre González-Gómez[4¶], Juan Raúl Medina-Sánchez[2], Alexander A. Martínez[1,6,7], Nohelia Castro del Campo[4], Cristóbal Chaidez-Quiroz[4], Jordi Querol-Audi[1,2,5,7,*], Alex Omar Martínez-Torres[2,3,7,*]

[1] Programa de Maestría en Ciencias Biomédicas, Facultad de Medicina, Universidad de Panamá, Panamá.

[2] Laboratorio de Microbiología Experimental y Aplicada y Microbiología de Aguas (LAMEXA-LAMA), Universidad de Panamá, Panamá.

[3] Departamento de Microbiología y Parasitología, Facultad de Ciencias Naturales, Exactas y Tecnología, Universidad de Panamá, Panamá.

[4] Laboratorio Nacional para la Investigación en Inocuidad Alimentaria (LANIIA), Centro de Investigación en Alimentación y Desarrollo, A.C. (CIAD), Culiacán, Sinaloa, México.

[5] Departamento de Bioquímica y Nutrición, Facultad de Medicina, Universidad de Panamá, Panamá.

[6] Genomics and Proteomics Research Department, Gorgas Memorial Institute of Health Studies, Panamá, Panamá.

[7] Sistema Nacional de Investigación (SNI). Secretaría Nacional de Ciencia, Tecnología e Innovación (SENACYT), Panamá.

[*]Corresponding authors:

E-mail: alex.martinez@up.ac.pa (AOMT) and jordi.querol@up.ac.pa (JQA)

[¶]These authors contributed equally to this work and share first authorship.

Abstract

The *Enterobacter cloacae* complex, a prominent bacterium responsible worldwide for most bloodstream infections in the hospital environment, has shown broad-spectrum antibiotic resistance, including carbapenems. Therefore, bacteriophages have again attracted the attention of the science and medical community as an alternative to control Multidrug resistant bacteria. In this study, water samples from Río Abajo River, in Panama City, Panama, were collected, for phage isolation, purification, characterization and propagation against the *E. cloacae* complex. As result, a phage produced clear and round plaque-forming units indicating a lytic phage was isolated. Further analyses concluded that this phage is stable at temperatures between 25°C and 50°C, it remains infective in a pH range between 7 to 11, with high sensitivity to Ultraviolet light. Remarkedly, it exhibits a narrow host specificity only infecting *E. cloacae*. Whole genome sequencing revealed that is a myovirus with a genome size of 178,477 bp, a G-C content of 45.8%, and containing approximately 294 genes. Among them, protein-encoding genes involved in morphology, inactivation, adsorption to cells, DNA injection and lytic enzymes were identified. Additionally, the genome contained two tRNA sequences. Genes that encode holins and endolysins, typical of lytic bacteriophages, were also present. A whole-genome sequencing analysis indicated that, according to the genus demarcation criteria, this phage belongs to a novel species within the Family *Straboviridae*, called genus *Pseudotevenvirus*.

Introduction

Enterobacteriaceae are ubiquitous in nosocomial settings and can play important roles in the burden of disease in the urinary tract, lower respiratory tract, intra-abdominal organs, and skin infections. The most relevant members of this Gram-negative bacterial group are *Klebsiella pneumoniae, Escherichia coli*, and *Enterobacter* spp. The severity of the infections is intensified by the resistance level to most available antibiotics, including carbapenem and cephalosporins [1]. Special attention to *Enterobacter* is due to the intrinsic resistance to ampicillin and broad-spectrum cephalosporin which reduces the alternatives for antibiotic treatments. *Enterobacter* belongs to the ESKAPE group (acronym for *Enterococcus faecium*, *Staphylococcus aureus*, *Klebsiella pneumoniae, Acinetobacter baumannii*, *Pseudomonas aeruginosa*, and *Enterobacter* spp), related worldwide to severe nosocomial infections with a highly increasing multi-drug resistance. A problem of epidemiological importance is the presence of AmpC-producing strains together with BLEE-encoding plasmids and colistin co-resistance in *E. cloacae* complex (ECC) strains**[2–4]**. The continuous emergence of multi-drug resistant strains have hampered the effectiveness of antibiotic treatments. Therefore, one possible approach to address this problem is the use of bacteriophages. In itself, the

magnitude of the impact of phage predation on pathogenic bacteria has been demonstrated. In recent years, phage therapy has been successfully applied to *E. coli* O157:H7, causative agent of hemorrhagic colitis and hemolytic uremic syndrome **[5–7]**. The use of phages has also been studied in the treatment of peritoneal infections, otitis externa, in the eradication of *P. aeruginosa* in patients with cystic fibrosis [8,9], in the treatment of urinary infections or osteomyelitis caused by *S. aureus* [10]. The favorable effects of phage therapy may even exceed its antibacterial activity. A surprising number of data has demonstrated the *in vivo* activity of phages such as the adenovirus growth suppression mediated by bacteriophage T4 [11] or development inhibition of *P. aeruginosa* by phage Pf4 [12].

The principal advantage of employing phages in therapy and biological control lies in their specificity towards bacterial targets. Those that do not infect commensal bacteria can be selected, therefore posing no threat to humans and animals. Their widespread presence in nature, their versatility, and their inherent resilience have elevated their status from mere research organisms to potentially viable and indispensable tools in the battle against growing antimicrobial resistance. However, the transfer of resistance and virulence genes, and other genes associated with negative outcomes through bacteriophages, constituted to an entire research domain aimed at mitigating any potential adverse impacts [13,14]. For these reasons, this study is aimed to biologically and genetically isolate and characterize a phage against the *E. cloacae* complex.

Methods

Bacterial Strains and Growth Conditions.

The host strain ATCC 23355 of *E. cloacae* complex used for the isolation and proliferation of the phage was the same as previously reported [15]. In addition, *S. aureus, E. coli, E. aerogenes, E. cloacae, and P. aeruginosa*, provided by the Human Microbiology Department, Faculty of Medicine, Universidad de Panamá; Hospital Santo Tomas, and Complejo Hospitalario Dr. Arnulfo Arias Madrid, were used for host range determination. The *E. cloacae* host strain, along with twenty-seven clinical isolates of *E. cloacae*, were tested for antibiotic susceptibility using the Vitek II Automated Microbiological System (BioMérieux, Marcy l'Étoile, France) (results shown in Supplementary Table 1). The breakpoints were applied according to the Clinical and Laboratory Standards Institute (CLSI) guidelines [16].

Ethics Statement

The Bioethics Committee of the Universidad de Panamá with the endorsement of the Dirección General de Salud Pública of the Ministerio de Salud (Ministry of Health), approved this study (Sequential: 617).

Phage isolation.

The Enterobacter phage vB_EcRAM-01 was isolated from waters of Rio Abajo river in Panama City, Panamá (coordinates, 9°00′55.2″N 79°29′25.6″W), using *E. cloacae* strain ATCC 23355 as a propagation host, which was grown previously in tryptic soy broth at 37°C for 24 h. Phage isolation, propagation, and purification were performed using the soft-agar overlay method [17]. Briefly, **phage purification process involved serial dilution of filtrates exhibiting lytic activity against *E. cloacae*. Specifically, 100 µL of the phage dilution was mixed with an overnight culture of the host bacterium in 3 mL of soft agar (TSB with 0.4% agar), poured onto TSA plates, and incubated at 37 °C for 24 h. The phage plates, selected based on size and clarity, were transferred to microtubes containing 1 mL of nanopure water and the whole process was repeated at least three times to confirm that the isolated phages were descendants of a single virion.**

Host range of vB_EcRAM-01 against clinical *E. cloacae* isolates: Spot test and efficiency of plating (EOP).

Clinical isolates of *E. cloacae, S. aureus, E. coli, P. aeruginosa*, and *E. aerogenes*, along with *E. cloacae* and *E. aerogenes* ATCC reference strains, were cultured until reaching the exponential growth phase (OD_{600nm} 0.6). Subsequently, 200 - 500 µL of each culture were mixed with 3 mL of top agar containing 10 mM $CaCl_2$ and plated onto a 1.5% nutrient agar plate. After solidification, a single droplet (10 µL) from each phage dilution (10^{-1} to 10^{-8}) was applied onto the agar [18].The droplets were allowed to dry for 10 min and plates were incubated at 37°C for 24 h - 48 h. A phage titer in PFU/mL was calculated for each strain tested [19]. Phage virulence was assessed using the efficiency of the plating (EOP) method. The EOP was determined by dividing the phage titer at the final dilution on the test strain by the phage titer on its original isolation strain. Based on these calculations, phages were categorized into highly virulent ($0.1 < EOP > 1.00$), avirulent but still active ($EOP < 0.001$), or completely avirulent (no plaques were observed).

Morphological characterization

The isolated phage underwent examination using transmission electron microscopy (TEM). A formvar-coated grid (400 mesh copper grid) was utilized, onto which a drop of 10^9 PFU/mL phage stock was applied. Subsequently, negative

staining was performed with 2 % phosphotungstic acid (pH 7.2) for 5 minutes, and excess stain was eliminated with filter paper. Electron micrographs were captured at varying magnifications using a JEOL JEM-1011 transmission electron microscope.(20).

Effects of UV light, temperature and pH on the viability of phage vB_EcRAM-01

Purified vB_EcRAM-01 was exposed to different physicochemical conditions (UV light, temperature and pH) Suspensions were exposed to ultraviolet light at a distance of 50 cm for 10, 20, 40, 60, 70, and 90 s. To determine the effect of temperature, phage suspensions were placed at 25 ºC, 37 °C, 50 ºC and 70 ºC, respectively. pH stability was determined by mixing assay vB_EcRAM-01 phage samples resuspended in SM-gelatine buffer (composed of 5.8 g NaCl, 2 g MgSO4.7H20, 50 mL TrisHCl 1M (pH 7.5), and 5 mL gelatin 2% (w/v) with buffer solutions ranging from pH 2 to pH 14. Phage viability was determined by counting the number of lysis PFU using the double-layer agar technique and the experiments were carried out in triplicates [20,21].

Bacterial reduction assay

Bacteriolytic activity was evaluated using a spectrophotometer, targeting *E. cloacae* ATCC 23355. The bacterial strain's overnight culture, adjusted to 1.0 OD_{600nm}, served as the inoculum. A concentration of 8 log PFU/mL was tested with appropriate phage dilutions. Each tube, including negative and positive controls, contained 20 mL of TSB. Experimental tubes received an additional 200 µL of the phage dilution, followed by the addition of 1000 µL of bacterial culture to each tube (excluding the negative control). Incubation was carried out for 180 minutes at 37 °C, with measurements at 600 nm taken every 15 minutes. The experiment was conducted in triplicates (23).

Bacteriophage DNA isolation and genome sequencing

The QIAamp MinElute Virus Spin kit (QIAGEN, Valencia, CA) was used to extract DNA, according to the manufacturer´s instructions. The purity was assessed spectrophotometrically at 260 and 280 nm, using a BioSpec-nano Micro-volume UV-Vis Spectrophotometer (Shimadzu, Kyoto, Japan).

The DNA libraries were prepared using the Nextera XT DNA Library Preparation Kit (Illumina, San Diego, CA, USA) through fragmentation, PCR amplification, PCR cleanup, and library normalization. The genome was sequenced at the Genomics and Proteomics Research Department of ICGES using the MiSeq Illumina sequencer (2 x 250 bp paired-end reads) (ThermoFisher Scientific Waltham, Massachusetts USA). Raw reads (629,372 reads in total) were checked for quality using FastQC (www.bioinformatics.Babraham.ac.UK/projects/fastqc) and trimmed using Timmomatic v0.39 [22].

Genome annotations, comparative genomics and phylogenetic analyses.

The genome was assembled through SPAdes v3.15.1 [23], resulting in a single contig with 8.96-fold coverage [23]. We mapped the reads against the contig obtained using Bowtie v0.9.6 (http://bowtie-bio.sourceforge.net/index.shtml) to corroborate the sequence ends [24]. The phage genome annotation was performed using RASTk v2.0 **[25–27]**. Gene calling was performed through GeneMarkS v4.28 (http://exon.gatech.edu/GeneMark/genemarks.cgi) [28], and Glimmer v3.02 (http://ccb.jhu.edu/software/glimmer/index.shtml) [29,30].

Several methods were used to annotate the ORF functions, including the protein basic local alignment search tool (blastp) on the NCBI server (https://blast.ncbi.nlm.nih.gov/Blast.cgi) based on the search of the non-redundant protein sequence database [31], Hhpred using structural/domain database (https://toolkit.tuebingen.mpg.de/tools/hhpred) **[32–35]** and HMMER v2.41.1 through HMM database [36,37]. (https://toolkit.tuebingen.mpg.de/tools/hhpred). Using a non-redundant protein matrix, employing Markov hidden model profiles [38], the annotations were manually curated using the Geneious v9.1.8 platform employing default parameters. Identification of transfer RNAs was performed using tRNA scan-SE v2.07 (http://lowelab.ucsc.edu/tRNAscan-SE/) and ARAGORN v1.2.41 [39] software (http://www.ansikte.se/ARAGORN/). Comparative analysis between genomes was performed using Mauve progressive alignment v2.3.1 [40] (http://darlinglab.org/mauve/mauve.html) employing default parameters.

Pairwise comparison of nucleotide sequences was performed using the Genome-BLAST Distance Phylogeny (GBDP) method of the VICTOR (Virus Classification and Tree Building Online Resource) platform [41] (https://ggdc.dsmz.de/victor.php), in the recommended environments for prokaryotic viruses. The resulting intergenomic distances were used to infer a branch-supported balanced minimum evolution tree using FASTME, including SPR post-processing [42] using D6 formula. Branch support was inferred from 100 pseudo-bootstrap replicates each. Trees were rooted at the midpoint [43], visualized using FigTree; personalized with taxon boundaries at species, genus, and family scales; and

estimated with the iTOL program [44] (https://itol.embl.de/). The recommended clustering threshold is thirthy nine [41] and an F-value (fraction of links required for cluster fusion) of 0.5 [41].

Determination of taxonomy and viral replication cycle type was achieved using PATRIC v3.6.9 [45] (https://patricbrc.org/) and PhageAI (https://phage.ai/) server [46]. The genome comparison between Enterobacter phage vB_EcRAM-01 and close relatives was performed using EasyFig v2.2.2 [47]. At the end, the intergenomic similarities amongst complete viral genomes were calculated through the VIRIDIC web tool [48] with blastn default settings.

Statistical analysis.

Statistically significant differences were determined using ANOVA. Differences in group means were compared using Tukey's HSD test. A P-value below 0.05 was considered statistically significant. Statistical analysis was conducted using R version 4.3.1 [49].

Results

Bacteriophage isolation, host range analysis and morphology characterization

In order to evaluate the host specificity of vB_EcRAM-01, a comprehensive assessment was conducted involving a total of thirty-three strains: two ATCC strains alongside a diverse selection of, both, Gram-negative and Gram-positive clinical isolates (Fig 1).

Fig 1. Host range specificity and efficiency of plating. The strain code followed by the efficiency value are shown in parentheses. Color range code associated with EOP is indicated on the right.

Notably, vB_EcRAM-01 exhibited the capacity to infect all the *E. cloacae* isolates with varying degrees of efficacy while displaying negligible antimicrobial activity (efficiency < 0.10) against the other species tested, which included *E. coli*, *E. aerogenes*, *A. baumannii*, *S. aureus*, and *P. aeruginosa*. Upon infecting the reference strain, the clear plaque-forming units (PFUs), characterized by smooth perimeters, measured within the range of 3 mm to 7 mm in diameter, a hallmark indicative of its lytic phage attributes (S1 Fig). TEM micrographs of the phage showed a morphology typical of a myovirus, a phage with a tail, belonging to the class *Caudoviricetes*. The size measurements of its icosahedral head are about 104 x 76 nm, and its

contractile tail is approximately 110 nm (S2 Fig). Previously, phage morphology was sufficient to classify them taxonomically, but nowadays bioinformatics tools are used to perform similarity analysis and group them by evolutionary distances.

Environmental stress resistance: stability against UV light, temperature and pH

Exposure to UV light (365 nm) decreased the infectivity of phage vB_ECRAM-01 over time. Results showed an exponential decay resulting in complete inactivation when incubated for more than one minute (Fig 2A).

Fig 2**. Stability of vB_ECRAM-01 to (A) UV radiation, (B) temperature, and (C) pH. Data is expressed as mean ± standard error. Treatments with the same letters in panel (C) are not significantly different (Tukey's HSD test, p>0.05).**The phage exhibited lytic activity when subjected to temperatures ranging from 25 °C to 50 °C, even after being incubated for 90 minutes (Fig 2B). However, when exposed to 70 °C, the phage became completely inactivated after only 20 min of incubation and, at 80 °C, it lost its infectivity entirely.

The stability of vB_EcRAM-01 was determined at various pH values. It maintained its infectivity in a pH range between pH 7.5 and pH 11. The best conditions were found between pH 9 and 10 (Fig 2C). Incubation under acidic pH resulted in a significant reduction of viral proliferation. The phage was not able to replicate in a pH greater than 11 or less than 3. Overall, the best activity was found in alkaline media.

Bacteriolytic activity

The evaluated phage demonstrated a robust, complete bacteriolytic effect in the initial 105 minutes of the assay. Following this, the OD curve started to rise due to an increase in bacterial cell numbers. This trend persisted in subsequent measurements, although the rate of increase was lower than that observed in the positive control (Fig 3).

Fig 3. Effect of vB_EcRAM-01 on the growth of the E. cloacae complex. The light blue line indicates the bacterial concentration without being infected with the virus at 37°C, showing an absorbance of 1.6. On the other hand, the orange line shows the concentration of the bacteria being infected from the beginning, with a reduced absorbance of up to 0.77 (ANOVA $F = 613.62$. g.l = 1 and $p<0.0001$).

Comparative genomics and phylogenetic analysis

The genomic attributes of phage vB_EcRAM-01 were delineated as follows: the genome spans 178,477 base pairs with a 45.0 % of GC content, hosting a total of 293 genes. Among these, 86 genes were implicated in anticipated functions encompassing morphology, inactivation, cellular adsorption, DNA injection, and host lysis. Through a UniProt database blast search, we were able to identify to structural proteins, one sharing 97% homology with the Major capsid protein (ID: A0A1B1IX95) of Citrobacter phage vB_CfrM_CfP1, having a mass of 56.37 kDa [50] while the other protein exhibits 96.7% homology with the Baseplate wedge subunit protein (ID: M9V1F2) from Escherichia phage Lw1, with a mass of 38.01 kDa [51]. In fact, analysis by polyacrylamide gel electrophoresis revealed two prominent protein bands with molecular masses close to 50 and 37 kDa, respectively (S3 Fig). In bacteriophages possessing both capsid and tail structures, SDS-PAGE typically reveals multiple proteins, including capsid, tail, and baseplate proteins. Capsid proteins form the outer shell encasing the phage genome, while baseplate proteins are situated at the tail base, facilitating recognition and binding to specific receptors on host cell surfaces [52]. Hence, it is plausible that the observed proteins serve these functions; however, precise identification through LC-MS/MS analysis would provide a more definitive comparison [53].

Remarkably, the genome of vB_EcRAM-01 was lacking discernible genes associated with resistance, virulence, or allergenicity. The genomic blueprint encodes two transfer RNAs (tRNAs), one corresponding to methionine (cat) and the other to glycine (tcc). A pertinent insight is the substantial sequence identity—90.11%—shared with other phages (Fig 4) [15].

Fig 4. Comparison of Enterobacter phage vB_EcRAM-01 genome with close relatives. Color scales represent the percentage of nucleotide identity between regions obtained by blastn.

To assign a taxonomic classification to phage vB_EcRAM-01, a phylogenetic tree based on whole-genome was constructed, and intergenomic similarities were calculated between the closely related genomes reported in GenBank. The phylogenetic tree and intergenomic similarity heatmap revealed that phage vB_EcRAM-01 is a new member of the *Pseudotevenvirus* genus within the *Straboviridae* family.

The GBDP phylogenetic tree using the D6 formula with an average support of 31%, identifying ten and nine clusters, respectively, at the genus level. At the family level, there were five and six clusters, respectively (Fig 5).

Fig 5. Phage vB_EcRAM-01 genomic and phylogenetic analyses. Genome Blast Distance Phylogeny (GBDP)-based phylogenetic tree of vB_EcRAM-01 and 28 related Straboviridae members created by iTOL v6.0 platform. The Enterobacter phage vB_EcRAM-01 belongs to the genus Pseudotevenvirus and is a new phage species.

Nucleotide identity analysis (ANI) was done on the 28 genomes with the highest homology in the GenBank database using VIRIDIC. According to the results of the heat mapping, phage vB_EcRAM-01 shares less than 95% nucleotide identity with previously reported genomes, thereby establishing it as a novel species within the Family *Straboviridae*, genus *Pseudotevenvirus*, according to the genus demarcation criteria (>70% nucleotide identity over the entire genome length) recommended by the ICTV (Fig 6).

Fig 6. Taxonomic classification of Enterobacter phage vB_EcRAM-01. A heatmap created by VIRIDIC based on intergenomic similarities between viral genomes, with the proportion of similarity shown by the color scale.

Comparative BLASTn analysis of the complete genome of vB_EcRAM-01 against phages in the GenBank database revealed that vB_EcRAM-01 has a high degree of coverage and identity with the Enterobacter phages ENC22 (OL355128.1), ENC7 (OL355125.1), ENC25 (OL355127.1), EBPL (MT341500.1). In VIRIDIC analysis, the phages shared collinear gene blocks that are identical in length between the two genomes. In comparison, other blocks revealed a high degree of similarity between this phage and others, including Cronobacter phage vB_CsaM IeE (NC_048646.1), Cronobacter phage vB_CsaM IeN (KX431560.1), Citrobacter phage Margaery (KT381880.1), and myoviral members of the subfamily *Tevenvirinae*.

Discussion

In Panama, seven out of the eight bacterial pathogens responsible for 80% of the reported infections concerning the emergence of resistance in a hospital level (ESKAPE) has been identified: *S. aureus* (16%), *Enterococcus* spp (14.4%), *E. coli* (12%), coagulase-negative *Staphylococcus* (11%), *K. pneumoniae* and *K. oxytoca* (8%), *P. aeruginosa* (8%), and *Enterobacter* spp. (5%). Several microorganisms developed antibiotic resistance to the more common antibiotics to treat these bacterial infections like piperacillin-tazobactam, cephalosporins, nitrofurans, and carbapenems [54,55].

Therefore, in comparison to the usage of antibiotics, phage therapy is a viable option. While scientific publications on phage isolation have increased in recent years, only a small number of phages have been commercialized [56]. One of the

primary reasons is a lack of adequate characterization, which is required to detect and mitigate the hazards associated with phage therapy [57,56]. Moreover, the biological characteristics of a phage determines its stability and therefore, its optimum administration forms [57].

As a first step, vB_EcRAM-01was subjected to environmental stress tests, such as temperature, UV radiation and pH, factors that play a role in phage survival [58,59]. The results indicate that vB_EcRAM-01 is stable at temperatures ranging from 25°C to 50°C, although its replication rate is highest at 37°C. This aligns with findings observed in Myoviridae phages [60,61] exhibiting a comparable stability profile within a broad temperature range. However, it deviates from the EBP phage, known for its resilience across a wider temperature span [62]. Notably, inactivation under UV light transpires rapidly, underscoring the necessity of safeguarding possible clinical applications from sunlight exposure. Interestingly, vB_EcRAM-01 maintains its infectivity across expansive pH and temperature ranges, implying potential robustness within the human body. Enhancements through administration systems designed to shield the phage from gastrointestinal pH could potentially further increase its utility and stability in therapeutic applications [63].

The primary objective of this study was to investigate the host specificity (host range) of phage vB_EcRAM-01 concerning isolates within the *E. cloacae* complex. Accurate identification of the host bacteria's specificity for bacteriophage vB_EcRAM-01 is crucial for a comprehensive characterization of this bacteriophage in order to evaluate a potential therapeutic use. Our observations indicate that this bacteriophage displayed reactivity only with its host strain. These results are in line with findings reported for phages TSE1, TSE2, and TSE3 [64]. *E. coli* displayed some sensitivity but did not exhibit complete lysis. Exceptions to this pattern were observed with myophages *Enterobacter* phage ENC22, *Enterobacter* phage ENC7, and *Enterobacter* phage ENC25, which demonstrated the ability to infect diverse *Enterobacter* species [65].

Apart from meeting the requirements of being innocuous to normal microbiota, therapeutic use involves the employment of fully lytic phages that lack undesired virulence factor genes or antibiotic resistance genes [66]. The genomic assessment of the phage vB_EcRAM-01 confirmed its status as a strictly lytic phage according to the PhageAI platform. The genome contained genes coding for holins and endolysins, offering several advantages. These enzymes collaborate to facilitate bacterial lysis, enabling the release of newly assembled viral particles and the termination of the bacterial host [67]. Additionally, holin proteins play a crucial role in regulating the timing of lysis, ensuring that it occurs at the appropriate stage of the phage life cycle [68]. An important aspect to note is that resistance to endolysins has not been reported to date [69]. This suggests a higher probability of success when employing phages containing endolysins. Besides, it lacked genes associated with lysogeny, such as integrases, transposons, and excitons [70].

The evolutionary context designated it as a T4-type myophage spanning a genome length of 178,477 base pairs. Computational analysis further classified it as a viral entity within the Class *Caudoviricetes*, *Strabovviridae* family, and *Pseudotevenvirus* genus [70]. This type of virus is characterized by the presence of tRNA. Due to the compact nature of phage genomes, which constrains their capacity to utilize host resources fully, these genetic elements may serve as compensation for compositional differences between phages and hosts. They have the potential to supplement the translation machinery with their genetic information, thereby improving fitness and fortifying virulence [71] (X).

By scanning the GenBank database for homologous sequences, a 92% sequence homology with myophage EBPL was observed, a phage that also infects *E. cloacae* [62], and 99.16% sequence homology with myophages ENC7, ENC25, and ENC22 [65].

According to genotypic characteristics [72], vB_EcRAM-01, like an EBP phage, is a myovirus-like phage [62]. The vB_EcRAM-01 virus was termed a bacterial infecting virus isolated from Rio Abajo waters that suppressed *E. cloacae*, a myophage, according to the informal phage nomenclature criteria [73–75].

In this study, the genome-wide syntenic analysis indicated that both phages had comparable rearrangements but differed slightly in nucleotide sequence. Additionally, a phylogenetic study of whole-genome sequences showed that both phages diverged from a common ancestor but experienced many nucleotides substitution events since then [76]. Given that EBPL was previously described as a singleton phage in the myophage phylogenetic tree [77], phylogenetic and nucleotide identity analyses indicated that vB_EcRAM-01 might represent a previously unreported species.

Therefore, vB_EcRAM-01 is physiologically and molecularly stable under a wide temperature and pH range. However, it has a restricted host range. Although it had intense lytic activity against the *E. cloacae* complex, isolation was deemed necessary for more efficient eradication.

In Conclusion, this study identified a lytic bacteriophage. The characterization enhances comprehension of this novel phage, and through its phylogenetic relatives, it may exhibit a myovirus-like morphology, as demonstrated by insights from next-generation sequencing. This sequencing elucidates that vB_EcRAM-01 aligns with the Myoviridae family in accordance with the conventional taxonomic classification [78]. According to the phylogenetic analysis previously described, this virus is a new member of the family *Straboviridae* and the genus *Pseudotevenvirus* according to the new classification [79]. The vB_EcRAM-01 strain is also a viable candidate for many clinical applications, including phage therapy and biologic control or decontamination, due to its host specificity, the genetic link, and lack of lysogenic genes.

Data availability statement

The datasets presented in this study can be found in online repositories. The names of the repository/repositories and accession number(s) can be found at https://www.ncbi.nlm.nih.gov/genbank/ , OL551674, SAMN23426491, SRR18344380.

Author Contributions

EEVB and JPGG drafted the manuscript, while JRMS conducted the statistical analysis of the results. NCC and CCHQ, along with JQA, AAM and AOMT, contributed to data analysis and manuscript revisions. EEVB, JQA, AAM and AOMT secured project funding. All authors reviewed and approved the submitted manuscript.

Acknowledgments

Host bacterial strains were provided by José E. Moreno, MSc., of the Microbiology Division of Laboratorio Central de Referencia en Salud Pública, Instituto Conmemorativo Gorgas de Estudios de Salud (ICGES). We extend our gratitude to the staff of the Microbiology Department at the Clinical Laboratory of Hospital Santo Tomas for providing us with the clinical bacteria strains.

Additionally, we would like to thank Claudia González, MSc, Jessica Gondola, MSc, Ambar Moreno, MSc, and Oris Chavarria, MSc, of the Research Department of Genomics and Proteomics of ICGES for providing us with NGS Illumina sequencing. Furthermore, we would like to express our gratitude to Leandra Gómez, MSc, of the Department of Pharmacology at the Faculty of Medicine, Universidad de Panamá, for her invaluable contribution in reviewing the bioethical considerations within this research. Also, thank you to Bs. Tatiana A. Martínez González for her contribution to the English revision of the present work.

Bibliography

1. WHO. Global priority list of antibiotic-resistant bacteria to guide research, discovery, and development of new antibiotics. 2017.

2. Qian W, Fu Y, Liu M, Zhang J, Wang W, Li J, et al. Mechanisms of Action of Luteolin Against Single- and Dual-Species of Escherichia coli and Enterobacter cloacae and Its Antibiofilm Activities. Appl Biochem Biotechnol. 2021;193: 1397–1414. doi:10.1007/s12010-020-03330-w

3. Davin-Regli A, Lavigne J-P, Pagès J-M. Enterobacter spp.: Update on Taxonomy, Clinical Aspects, and Emerging Antimicrobial Resistance. Clin Microbiol Rev. 2019;32. doi:10.1128/CMR.00002-19

4. Mezzatesta ML, Gona F, Stefani S. *Enterobacter cloacae* complex: clinical impact and emerging antibiotic resistance. Future Microbiology. 2012;7: 887–902. doi:10.2217/fmb.12.61

5. Amarillas L, Rubí-Rangel L, Chaidez C, González-Robles A, Lightbourn-Rojas L, León-Félix J. Isolation and Characterization of phiLLS, a Novel Phage with Potential Biocontrol Agent against Multidrug-Resistant Escherichia coli. Frontiers in Microbiology. 2017;8. Available: https://www.frontiersin.org/articles/10.3389/fmicb.2017.01355

6. López-Cuevas O, Castro-Del Campo N, León-Félix J, Valdez-Torres B, Chaidez C. Evaluation of Bacteriophage Av-08 for Simultaneous Biocontrol of Salmonella Montevideo and Escherichia Coli O157:h7 in Experimentally Contaminated Chicken Skin. Journal of Food Safety. 2012;32: 305–310. doi:10.1111/j.1745-4565.2012.00381.x

7. Sharma M, Patel JR, Conway WS, Ferguson S, Sulakvelidze A. Effectiveness of Bacteriophages in Reducing Escherichia coli O157:H7 on Fresh-Cut Cantaloupes and Lettuce. Journal of Food Protection. 2009;72: 1481–1485. doi:10.4315/0362-028X-72.7.1481

8. Morello E, Saussereau E, Maura D, Huerre M, Touqui L, Debarbieux L. Pulmonary Bacteriophage Therapy on Pseudomonas aeruginosa Cystic Fibrosis Strains: First Steps Towards Treatment and Prevention. Aziz R, editor. PLoS ONE. 2011;6: e16963. doi:10.1371/journal.pone.0016963

9. Debarbieux L, Leduc D, Maura D, Morello E, Criscuolo A, Grossi O, et al. Bacteriophages Can Treat and Prevent *Pseudomonas aeruginosa* Lung Infections. J INFECT DIS. 2010;201: 1096–1104. doi:10.1086/651135

10. Abedon ST. Commentary: Phage Therapy of Staphylococcal Chronic Osteomyelitis in Experimental Animal Model. Front Microbiol. 2016;7. doi:10.3389/fmicb.2016.01251

11. Przybylski M, Dzieciątkowski T, Borysowski J, Międzybrodzki R, Górski A. Inhibitory Effects of Bacteriophage Preparations on Adenoviral Replication. INT. 2019;62: 37–44. doi:10.1159/000500540

12. Casto A, Hurwitz A, Kou K, Mansour G, Policke R, Schmidt A, et al. Bacteriophages: The Answer to Antibiotic Resistance? James Madison Undergraduate Research Journal. 20163: 36–41.

13. Hassan AY, Lin JT, Ricker N, Anany H. The Age of Phage: Friend or Foe in the New Dawn of Therapeutic and Biocontrol Applications? Pharmaceuticals. 2021;14: 199. doi:10.3390/ph14030199

14. Häusler T. Viruses vs. Superbugs. London: Palgrave Macmillan UK; 2006. doi:10.1007/978-0-230-55228-9

15. Victoria-Blanco EE, González-Gómez JP, Quiroz E, Martínez AA, González C, Castro del Campo N, et al. Complete Genome Sequence of Enterobacter Phage vB_EcRAM-01, a New Pseudotevenvirus against the Enterobacter cloacae Complex. Microbiology Resource Announcements. 10 May 2022: 1–3. doi:10.1128/mra.00045-22

16. CLSI. CLSI Performance Standards for Antimicrobial Susceptibility Testing, CLSI Guideline M100. 28th ed. Wayne, PA: Clinical and Laboratory Standards Institute; 2018.

17. Adams MH, Anderson ES, Kellenberger E. Bacteriophages. New York: Interscience Publishers; 1959.

18. Khan Mirzaei M, Nilsson AS. Isolation of Phages for Phage Therapy: A Comparison of Spot Tests and Efficiency of Plating Analyses for Determination of Host Range and Efficacy. Schuch R, editor. PLoS ONE. 2015;10: e0118557. doi:10.1371/journal.pone.0118557

19. Stephenson, Frank H. Calculations for Molecular Biology and Biotechnology. second edition. USA: Elsevier/AP, Academic Press is an imprint of Elsevier; 2010. Available: Vetbiotech.um.ac.ir

20. Flores V. Caracterización de bacteriófagos líticos de Salmonella enterica aislados de muestras de pollos. Universidad Mayor San Marcos. 2017.

21. Zaburlin D, Quiberoni A, Mercanti D. Changes in Environmental Conditions Modify Infection Kinetics of Dairy Phages. Food Environ Virol. 2017;9: 270–276. doi:10.1007/s12560-017-9296-2

22. Bolger AM, Lohse M, Usadel B. Trimmomatic: a flexible trimmer for Illumina sequence data. Bioinformatics. 2014;30: 2114–2120. doi:10.1093/bioinformatics/btu170

23. Bankevich A, Nurk S, Antipov D, Gurevich AA, Dvorkin M, Kulikov AS, et al. SPAdes: A New Genome Assembly Algorithm and Its Applications to Single-Cell Sequencing. Journal of Computational Biology. 2012;19: 455–477. doi:10.1089/cmb.2012.0021

24. Langmead B, Trapnell C, Pop M, Salzberg SL. Ultrafast and memory-efficient alignment of short DNA sequences to the human genome. Genome Biology. 2009;10: R25. doi:10.1186/gb-2009-10-3-r25

25. Aziz RK, Bartels D, Best AA, DeJongh M, Disz T, Edwards RA, et al. The RAST Server: rapid annotations using subsystems technology. BMC Genomics. 2008;9: 75. doi:10.1186/1471-2164-9-75

26. Brettin T, Davis JJ, Disz T, Edwards RA, Gerdes S, Olsen GJ, et al. RASTtk: a modular and extensible implementation of the RAST algorithm for building custom annotation pipelines and annotating batches of genomes. Sci Rep. 2015;5: 8365. doi:10.1038/srep08365

27. Overbeek R, Olson R, Pusch GD, Olsen GJ, Davis JJ, Disz T, et al. The SEED and the Rapid Annotation of microbial genomes using Subsystems Technology (RAST). Nucleic Acids Res. 2014;42: D206-214. doi:10.1093/nar/gkt1226

28. Besemer J, Lomsadze A, Borodovsky M. GeneMarkS: a self-training method for prediction of gene starts in microbial genomes. Implications for finding sequence motifs in regulatory regions. Nucleic Acids Research. 2001;29: 2607–2618. doi:10.1093/nar/29.12.2607

29. Delcher AL, Bratke KA, Powers EC, Salzberg SL. Identifying bacterial genes and endosymbiont DNA with Glimmer. Bioinformatics. 2007;23: 673–679. doi:10.1093/bioinformatics/btm009

30. Salzberg SL, Delcher AL, Kasif S, White O. Microbial gene identification using interpolated Markov models. Nucleic Acids Res. 1998;26: 544–548. doi:10.1093/nar/26.2.544

31. Altschul SF, Madden TL, Schäffer AA, Zhang J, Zhang Z, Miller W, et al. Gapped BLAST and PSI-BLAST: a new generation of protein database search programs. Nucleic Acids Res. 1997;25: 3389–3402.

32. Gabler F, Nam S-Z, Till S, Mirdita M, Steinegger M, Söding J, et al. Protein Sequence Analysis Using the MPI Bioinformatics Toolkit. Current Protocols in Bioinformatics. 2020;72: e108. doi:https://doi.org/10.1002/cpbi.108

33. Zimmermann L, Stephens A, Nam S-Z, Rau D, Kübler J, Lozajic M, et al. A Completely Reimplemented MPI Bioinformatics Toolkit with a New HHpred Server at its Core. Journal of Molecular Biology. 2018;430: 2237–2243. doi:10.1016/j.jmb.2017.12.007

34. Meier A, Söding J. Automatic Prediction of Protein 3D Structures by Probabilistic Multi-template Homology Modeling. PLOS Computational Biology. 2015;11: e1004343. doi:10.1371/journal.pcbi.1004343

35. Hildebrand A, Remmert M, Biegert A, Söding J. Fast and accurate automatic structure prediction with HHpred. Proteins: Structure, Function, and Bioinformatics. 2009;77: 128–132. doi:https://doi.org/10.1002/prot.22499

36. Potter SC, Luciani A, Eddy SR, Park Y, Lopez R, Finn RD. HMMER web server: 2018 update. Nucleic Acids Research. 2018;46: W200–W204. doi:10.1093/nar/gky448

37. Finn RD, Clements J, Arndt W, Miller BL, Wheeler TJ, Schreiber F, et al. HMMER web server: 2015 update. Nucleic Acids Research. 2015;43: W30–W38. doi:10.1093/nar/gkv397

38. Hofmann P, Tashman Z. Hidden Markov Models and Their Application for Predicting Failure Events. In: Krzhizhanovskaya VV, Závodszky G, Lees MH, Dongarra JJ, Sloot PMA, Brissos S, et al., editors. Computational Science – ICCS 2020. Cham: Springer International Publishing; 2020. pp. 464–477. doi:10.1007/978-3-030-50420-5_35

39. Laslett D, Canback B. ARAGORN, a program to detect tRNA genes and tmRNA genes in nucleotide sequences. Nucleic Acids Res. 2004;32: 11–16. doi:10.1093/nar/gkh152

40. Darling ACE, Mau B, Blattner FR, Perna NT. Mauve: Multiple Alignment of Conserved Genomic Sequence With Rearrangements. Genome Res. 2004;14: 1394–1403. doi:10.1101/gr.2289704

41. Meier-Kolthoff JP, Göker M. VICTOR: genome-based phylogeny and classification of prokaryotic viruses. Bioinformatics. 2017;33: 3396–3404. doi:10.1093/bioinformatics/btx440

42. Lefort V, Desper R, Gascuel O. FastME 2.0: A Comprehensive, Accurate, and Fast Distance-Based Phylogeny Inference Program. Mol Biol Evol. 2015;32: 2798–2800. doi:10.1093/molbev/msv150

43. Farris JS. Estimating Phylogenetic Trees from Distance Matrices. The American Naturalist. 1972;106: 645–668.

44. Letunic I, Bork P. Interactive Tree Of Life (iTOL) v4: recent updates and new developments. Nucleic Acids Research. 2019;47: W256–W259. doi:10.1093/nar/gkz239

45. Davis JJ, Wattam AR, Aziz RK, Brettin T, Butler R, Butler RM, et al. The PATRIC Bioinformatics Resource Center: expanding data and analysis capabilities. Nucleic Acids Res. 2020;48: D606–D612. doi:10.1093/nar/gkz943

46. Tynecki P, Guziński A, Kazimierczak J, Jadczuk M, Dastych J, Onisko A. PhageAI - Bacteriophage Life Cycle Recognition with Machine Learning and Natural Language Processing. 2020 [cited 11 Apr 2021]. doi:10.1101/2020.07.11.198606

47. Sullivan MJ, Petty NK, Beatson SA. Easyfig: a genome comparison visualizer. Bioinformatics. 2011;27: 1009–1010. doi:10.1093/bioinformatics/btr039

48. Moraru C, Varsani A, Kropinski AM. VIRIDIC—A Novel Tool to Calculate the Intergenomic Similarities of Prokaryote-Infecting Viruses. Viruses. 2020;12: 1268. doi:10.3390/v12111268

49. R Core Team. R: A Language and Environment for Statistical Computing. Vienna, Austria: R Foundation for Statistical Computing; 2023.

50. Oliveira H, Pinto G, Oliveira A, Oliveira C, Faustino MA, Briers Y, et al. Characterization and genome sequencing of a Citrobacter freundii phage CfP1 harboring a lysin active against multidrug-resistant isolates. Appl Microbiol Biotechnol. 2016;100: 10543–10553. doi:10.1007/s00253-016-7858-0

51. Kushkina AI, Tovkach FI, Comeau AM, Kostetskii IE, Lisovski I, Ostapchuk AM, et al. Complete Genome Sequence of Escherichia Phage Lw1, a New Member of the RB43 Group of Pseudo T-Even Bacteriophages. Genome Announcements. 2013;1: 10.1128/genomea.00743-13. doi:10.1128/genomea.00743-13

52. Kutter E, Sulakvelidze A, editors. Bacteriophages: biology and applications. Boca Raton, FL: CRC Press; 2005.

53. Al-Wrafy F, Brzozowska E, Górska S, Drab M, Strus M, Gamian A. Identification and characterization of phage protein and its activity against two strains of multidrug-resistant Pseudomonas aeruginosa. Sci Rep. 2019;9: 13487. doi:10.1038/s41598-019-50030-5

54. Moreno J. Antibiotic resistance profiles in Panama: Trends from 2007 to 2013. PPIJ. 2018;6. doi:10.15406/ppij.2018.06.00200

55. MINSA. 18400_2011_31657_INFORME RESISTENCIA ANTIBIOTICOS-USO RACIONAL -036.pdf. Panama: ICGES; 2015 p. 48.

56. Abedon ST. Detection of Bacteriophages: Phage Plaques. In: Harper DR, Abedon ST, Burrowes BH, McConville ML, editors. Bacteriophages. Cham: Springer International Publishing; 2018. pp. 1–32. doi:10.1007/978-3-319-40598-8_16-1

57. Hyman P, Abedon ST. Bacteriophage: Overview. Reference Module in Biomedical Sciences. Elsevier; 2019. p. B978012801238302506X. doi:10.1016/B978-0-12-801238-3.02506-X

58. Phothichaisri W, Ounjai P, Phetruen T, Janvilisri T, Khunrae P, Singhakaew S, et al. Characterization of Bacteriophages Infecting Clinical Isolates of Clostridium difficile. Front Microbiol. 2018;9. doi:10.3389/fmicb.2018.01701

59. Silva YJ, Costa L, Pereira C, Cunha Â, Calado R, Gomes NCM, et al. Influence of environmental variables in the efficiency of phage therapy in aquaculture. Microbial Biotechnology. 2014;7: 401–413. doi:10.1111/1751-7915.12090

60. Litt PK, Jaroni D. Isolation and Physiomorphological Characterization of Escherichia coli O157:H7-Infecting Bacteriophages Recovered from Beef Cattle Operations. In: International Journal of Microbiology [Internet]. 2017 [cited 9 Jul 2019]. doi:10.1155/2017/7013236

61. Yamaki S, Kawai Y, Yamazaki K. Characterization of a novel bacteriophage, Phda1, infecting the histamine-producing Photobacterium damselae subsp. damselae. Journal of Applied Microbiology. 2015;118: 1541–1550. doi:https://doi.org/10.1111/jam.12809

62. Asif M, Naseem H, Alvi IA, Basit A, Rehman S-. Characterization of a lytic EBP bacteriophage with large size genome against Enterobacter cloacae. APMIS. 2021;129: 461–469. doi:10.1111/apm.13138

63. Dąbrowska K. Phage therapy: What factors shape phage pharmacokinetics and bioavailability? Systematic and critical review. Med Res Rev. 2019;39: 2000–2025. doi:10.1002/med.21572

64. Khawaja KA, Abbas Z, Rehman S ur. Isolation and characterization of lytic phages TSE1-3 against Enterobacter cloacae. Open Life Sciences. 2016;11. doi:10.1515/biol-2016-0038

65. Finney AG, Perry JM, Evans DR, Westbrook KJ, McElheny CL, Iovleva A, et al. Isolation and Characterization of Lytic Bacteriophages Targeting Diverse Enterobacter spp. Clinical Isolates. PHAGE. 2022;3: 50–58. doi:10.1089/phage.2021.0011

66. Soohey RL. Identifyng Genes Essential For Lysogeny in Cluster e Mycobacteriophage Ukulele. The University of Maine. 2017.

67. Pollenz RS, Bland J, Pope WH. Bioinformatic characterization of endolysins and holin-like membrane proteins in the lysis cassette of phages that infect Gordonia rubripertincta. PLOS ONE. 2022;17: e0276603. doi:10.1371/journal.pone.0276603

68. Zhang M, Wang Y, Chen J, Hong X, Xu X, Wu Z, et al. Identification and Characterization of a New Type of Holin-Endolysin Lysis Cassette in Acidovorax oryzae Phage AP1. Viruses. 2022;14: 167. doi:10.3390/v14020167

69. Larpin Y, Oechslin F, Moreillon P, Resch G, Entenza JM, Mancini S. In vitro characterization of PlyE146, a novel phage lysin that targets Gram-negative bacteria. PLOS ONE. 2018;13: e0192507. doi:10.1371/journal.pone.0192507

70. Ríos-Sandoval M. Caracterización Genómica, Morfológica y Replicativa del Bacteriófago ΦXaF18 de Xanthomonas vesicatoria. Centro De Investigación Y Asistencia En Tecnología Y Diseño Del Estado De Jalisco, A. C. 2019.

71. Hatfull GF, Dedrick RM, Schooley RT. Phage Therapy for Antibiotic-Resistant Bacterial Infections. Annu Rev Med. 2022;73: 197–211. doi:10.1146/annurev-med-080219-122208

72. Hatfull GF. Bacteriophage genomics. Current Opinion in Microbiology. 2008;11: 447–453. doi:10.1016/j.mib.2008.09.004

73. Turner D, Kropinski AM, Adriaenssens EM. A Roadmap for Genome-Based Phage Taxonomy. Viruses. 2021;13: 506. doi:10.3390/v13030506

74. Adriaenssens EM, Sullivan MB, Knezevic P, van Zyl LJ, Sarkar BL, Dutilh BE, et al. Taxonomy of prokaryotic viruses: 2018-2019update from the ICTV Bacterial and Archaeal Viruses Subcommittee. Arch Virol. 2020;165: 1253–1260. doi:10.1007/s00705-020-04577-8

75. Adriaenssens E, Brister JR. How to Name and Classify Your Phage: An Informal Guide. Viruses. 2017;9: 70. doi:10.3390/v9040070

76. Leskinen K, Blasdel BG, Lavigne R, Skurnik M. RNA-Sequencing Reveals the Progression of Phage-Host Interactions between φR1-37 and Yersinia enterocolitica. Viruses. 2016;8: 111. doi:10.3390/v8040111

77. Jacquemot L, Bettarel Y, Monjol J, Corre E, Halary S, Desnues C, et al. Therapeutic Potential of a New Jumbo Phage That Infects Vibrio coralliilyticus, a Widespread Coral Pathogen. Front Microbiol. 2018;0. doi:10.3389/fmicb.2018.02501

78. Adams MJ, Lefkowitz EJ, King AMQ, Harrach B, Harrison RL, Knowles NJ, et al. 50 years of the International Committee on Taxonomy of Viruses: progress and prospects. Arch Virol. 2017;162: 1441–1446. doi:10.1007/s00705-016-3215-y

79. Turner D, Shkoporov AN, Lood C, Millard AD, Dutilh BE, Alfenas-Zerbini P, et al. Abolishment of morphology-based taxa and change to binomial species names: 2022 taxonomy update of the ICTV bacterial viruses subcommittee. Arch Virol. 2023;168: 74. doi:10.1007/s00705-022-05694-2

Supplementary figure legends

S1 Fig. Lytic effect of bacteriophage vB_EcRAM-01. According to the double agar plate method, the bacterial lawn of the *E. cloacae* complex is infected by bacteriophages. After purification, lysis PFU measuring approximately 9 mm (shown in the figure), rough-edged and completely clear, were obtained.

S2 Fig. Overall morphology of vB_EcRAM-01. A representative micrograph of a purified sample of the bacteriophage is shown (scale bar: 100nm).

S3 Fig. Electrophoretic profiles (SDS-PAGE) of phage EcRAM-01*.* M: Precision Plus protein ladder., S: phage sample. Ladder molecular weights are indicated on the left.

Figure 1 Click here to access/download;Figure;Fig 1.tif

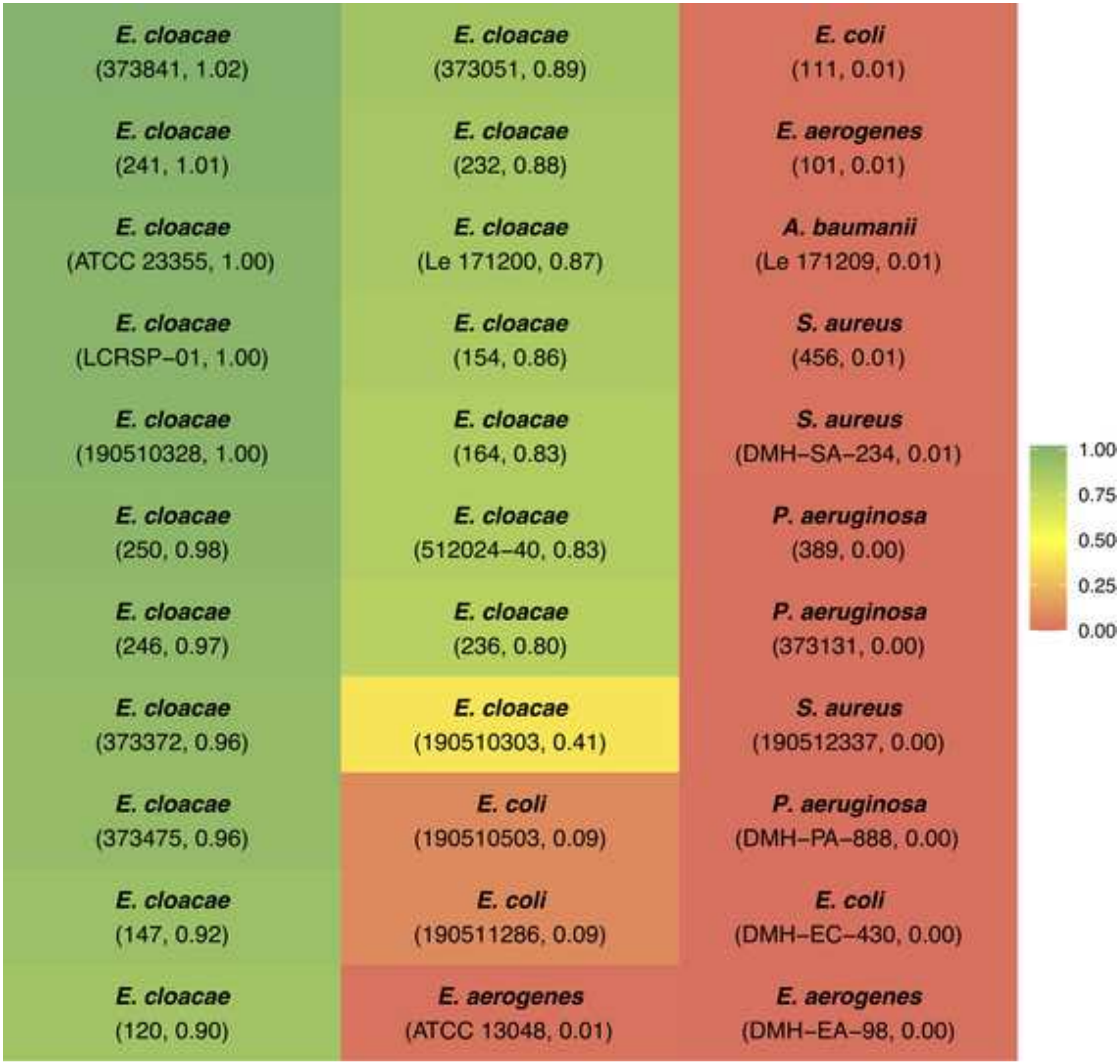

Figure 2

Click here to access/download;Figure;Fig 2.tiff

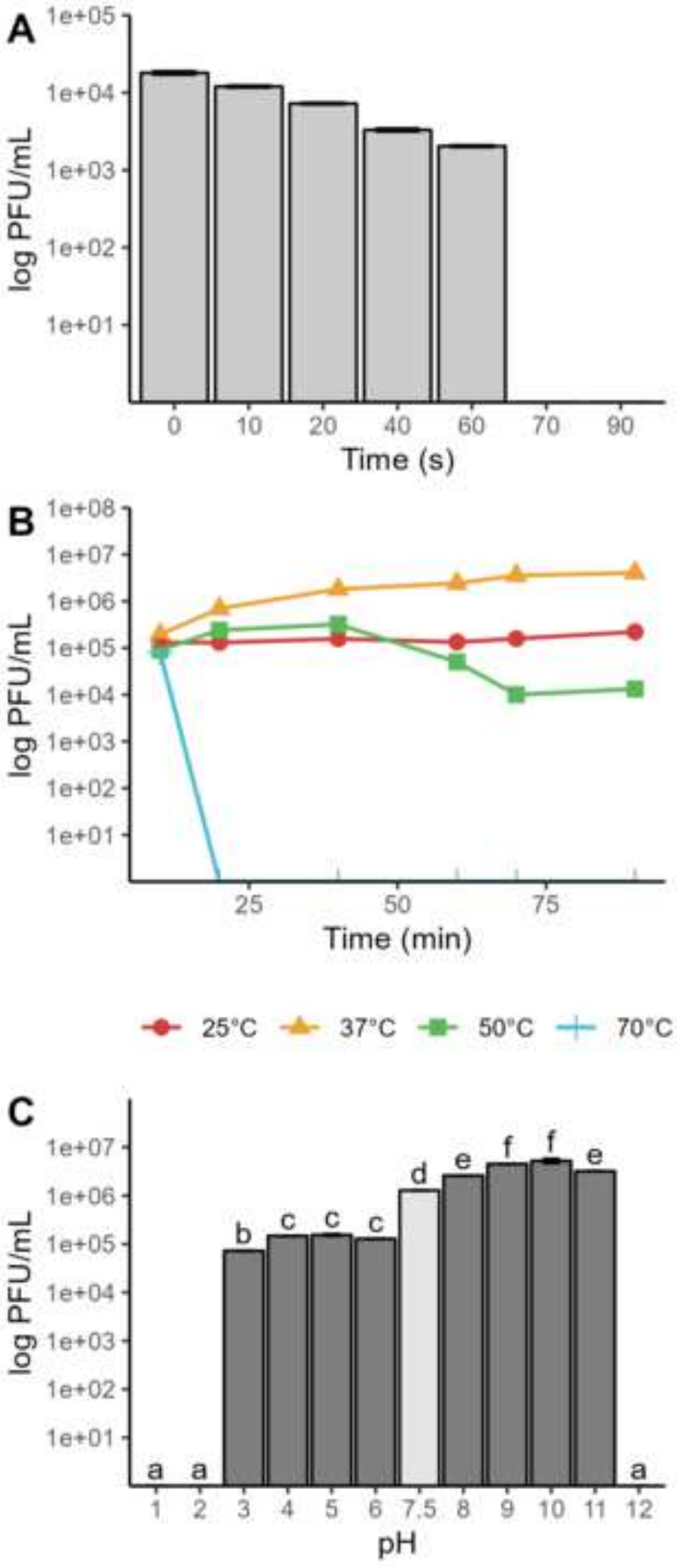

Figure 3

Click here to access/download;Figure;Fig 3.tif

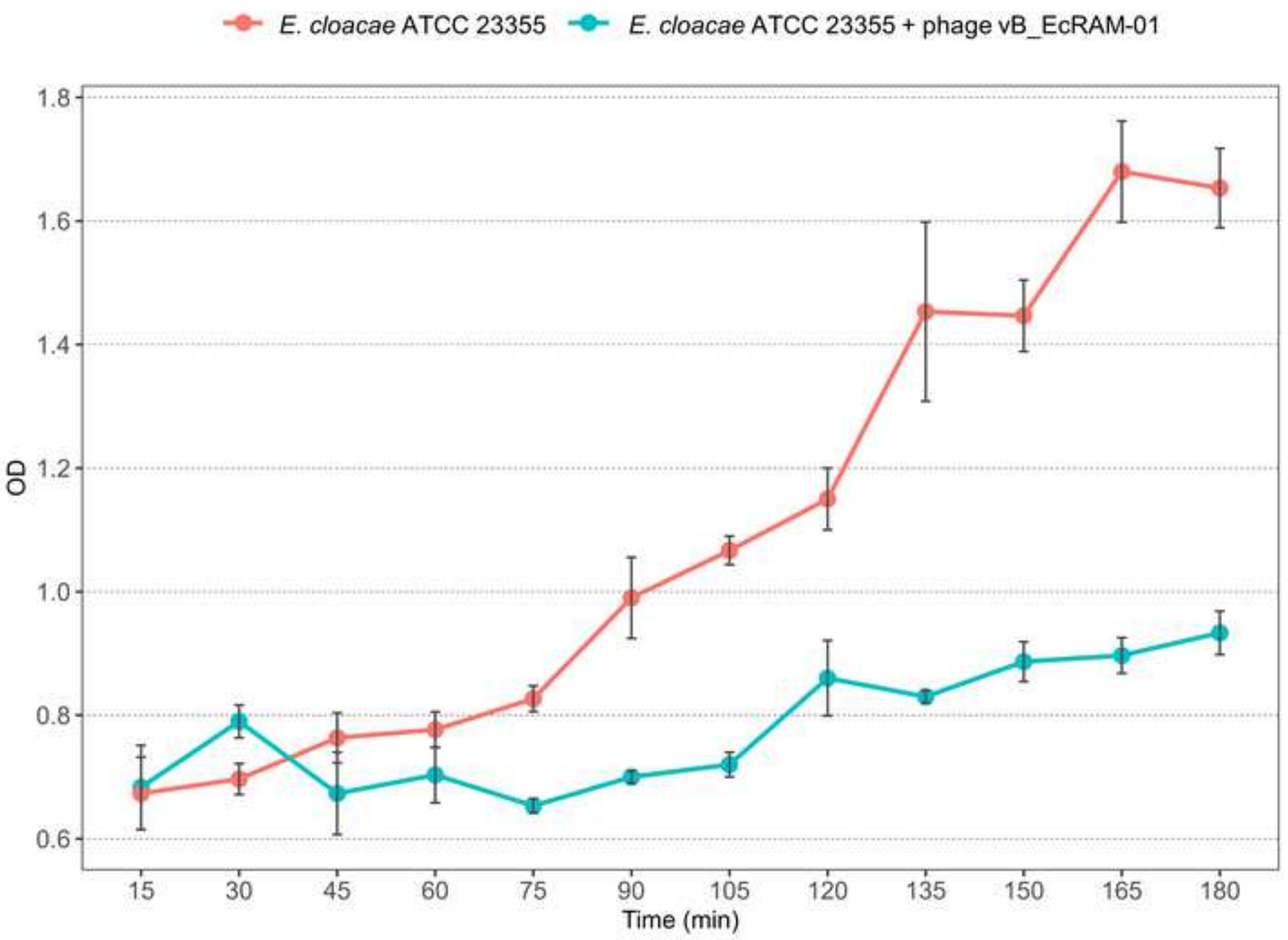

Figure 4

Click here to access/download;Figure;Figure4.eps

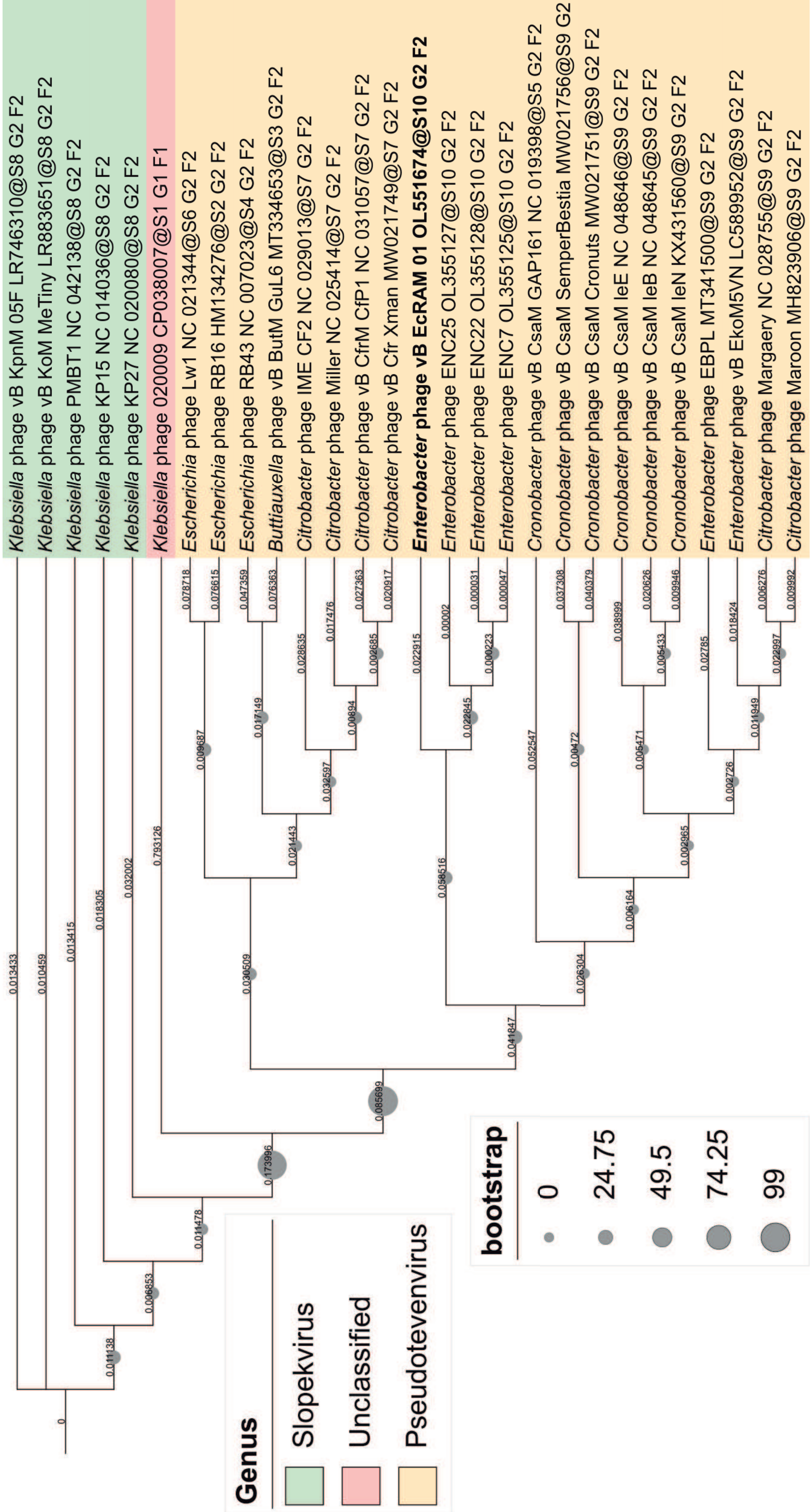

Figure 5

Click here to access/download;Figure;Fig 5.tif

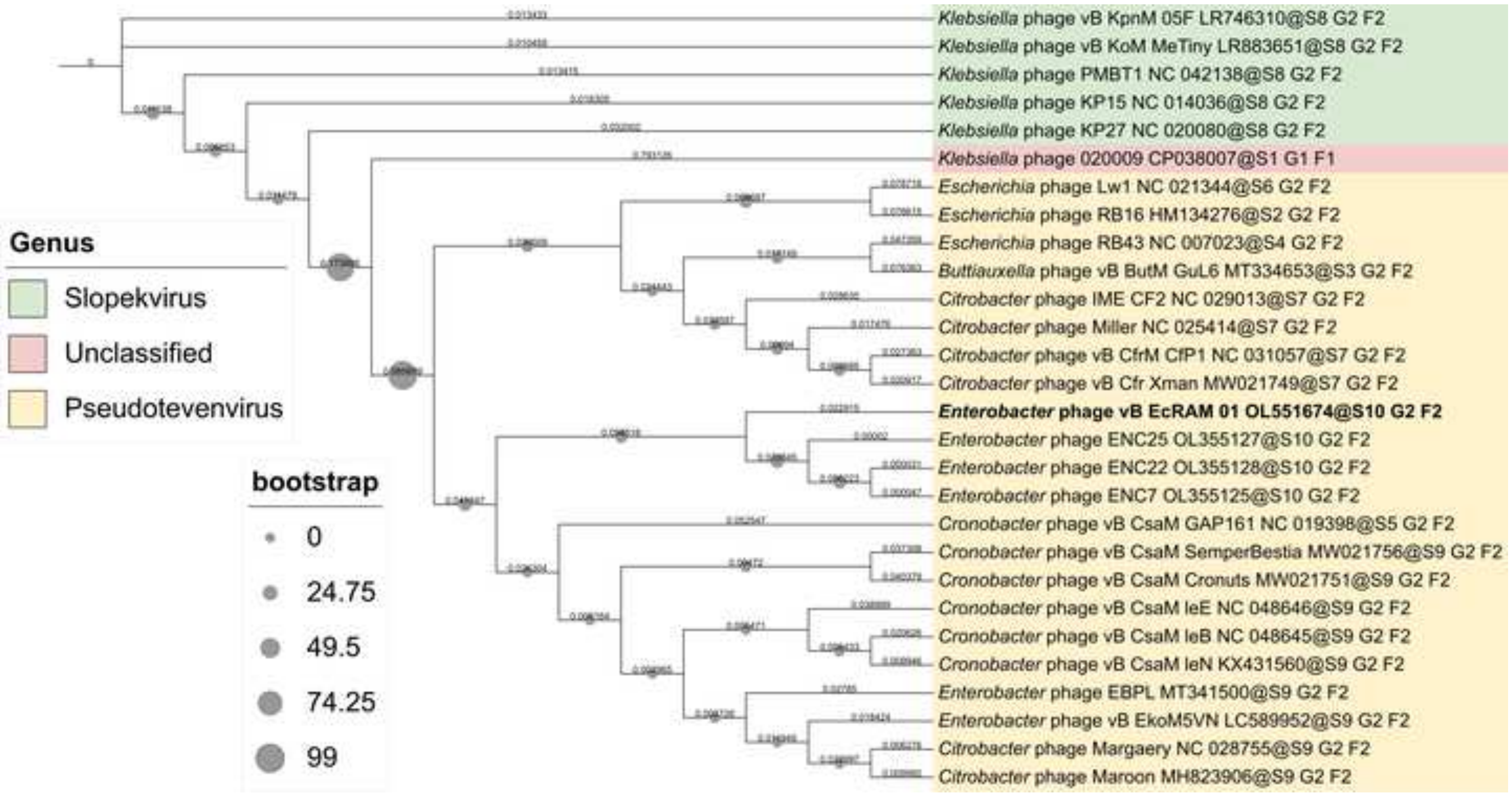

Figure 6

Click here to access/download;Figure;Fig 6.tif

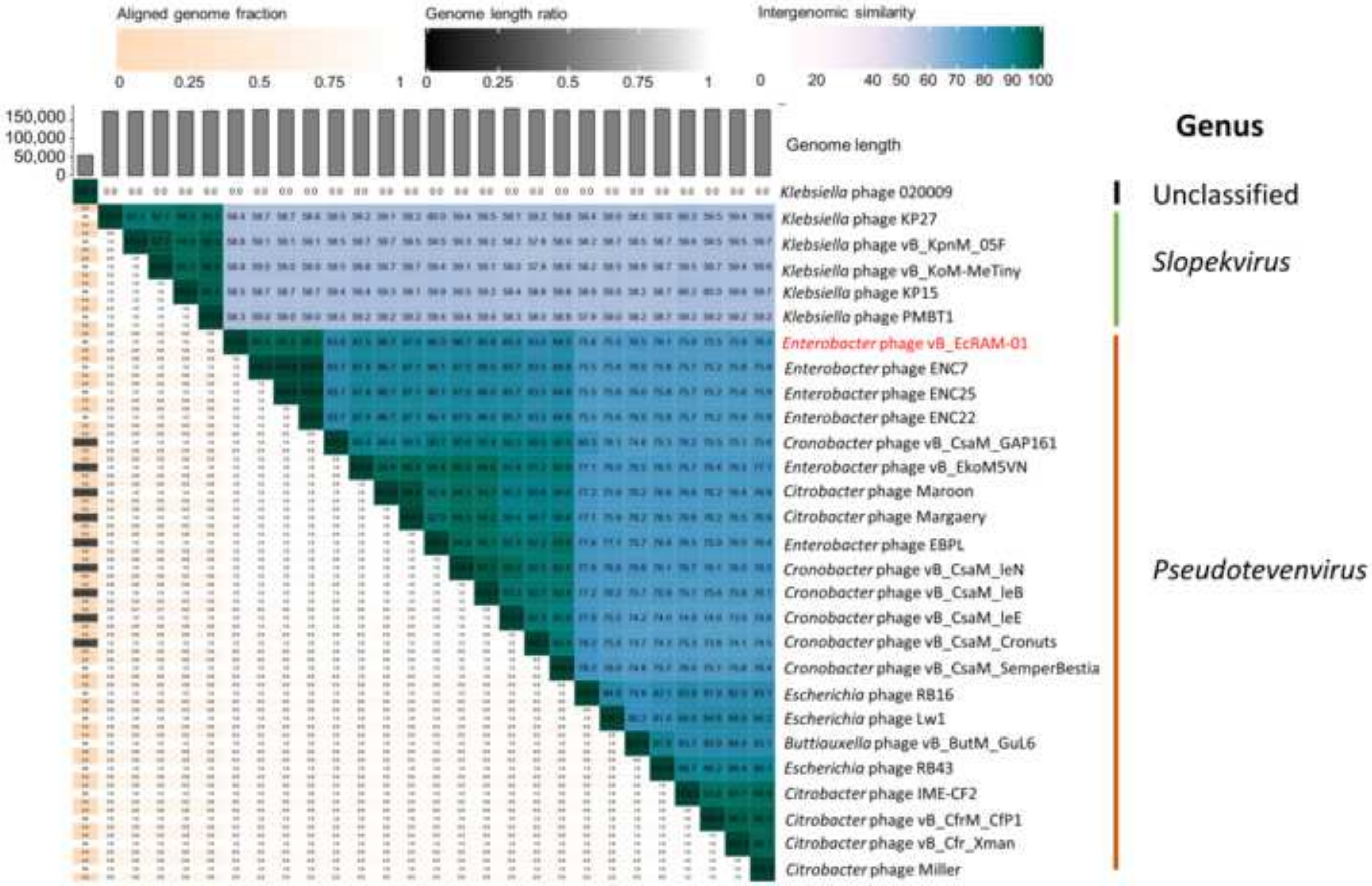

Manuscript

Click here to access/download
Supporting Information
Victoria-Blanco et. al. Supplementary material.docx

Click here to access/download
Supporting Information
Suppl. Information.docx

Characterization of Enterobacter phage vB_EcRAM-01, a new *Pseudotevenvirus* against *Enterobacter cloacae*, isolated in an urban river in Panama.

Ednner E. Victoria-Blanco[1,2,7,¶], Jean Pierre González-Gómez[4,¶], Juan Raúl Medina-Sánchez[2], Alexander A. Martínez[1,6,7], Nohelia Castro del Campo[4], Cristóbal Chaidez-Quiroz[4], Jordi Querol-Audi[1,2,5,7,*], Alex Omar Martínez-Torres[2,3,7,*]

[1] Programa de Maestría en Ciencias Biomédicas, Facultad de Medicina, Universidad de Panamá, Panamá.

[2] Laboratorio de Microbiología Experimental y Aplicada y Microbiología de Aguas (LAMEXA-LAMA), Universidad de Panamá, Panamá.

[3] Departamento de Microbiología y Parasitología, Facultad de Ciencias Naturales, Exactas y Tecnología, Universidad de Panamá, Panamá.

[4] Laboratorio Nacional para la Investigación en Inocuidad Alimentaria (LANIIA), Centro de Investigación en Alimentación y Desarrollo, A.C. (CIAD), Culiacán, Sinaloa, México.

[5] Departamento de Bioquímica y Nutrición, Facultad de Medicina, Universidad de Panamá, Panamá.

[6] Genomics and Proteomics Research Department, Gorgas Memorial Institute of Health Studies, Panamá, Panamá.

[7] Sistema Nacional de Investigación (SNI). Secretaría Nacional de Ciencia, Tecnología e Innovación (SENACYT), Panamá.

[*]Corresponding authors:

E-mail: alex.martinez@up.ac.pa (AOMT) and jordi.querol@up.ac.pa (JQA)

[¶]These authors contributed equally to this work and share first authorship.

Abstract

The *Enterobacter cloacae* complex, a prominent bacterium responsible worldwide for most bloodstream infections in the hospital environment, has shown broad-spectrum antibiotic resistance, including carbapenems. Therefore, bacteriophages have again attracted the attention of the science and medical community as an alternative to control Multidrug resistant bacteria. In this study, water samples from Río Abajo River, in Panama City, Panama, were collected, for phage isolation, purification, characterization and propagation against the *E. cloacae* complex. As result, a phage produced clear and round plaque-forming units indicating a lytic phage was isolated. Further analyses concluded that this phage is stable at temperatures between 25°C and 50°C, it remains infective in a pH range between 7 to 11, with high sensitivity to Ultraviolet light. Remarkedly, it exhibits a narrow host specificity only infecting *E. cloacae*. Whole genome sequencing revealed that is a myovirus with a genome size of 178,477 bp, a G-C content of 45.8%, and containing approximately 294 genes. Among them, protein-encoding genes involved in morphology, inactivation, adsorption to cells, DNA injection and lytic enzymes were identified. Additionally, the genome contained two tRNA sequences. Genes that encode holins and endolysins, typical of lytic bacteriophages, were also present. A whole-genome sequencing analysis indicated that, according to the genus demarcation criteria, this phage belongs to a novel species within the Family *Straboviridae*, called genus *Pseudotevenvirus*.

Formatted: Indent: First line: 0.5"

Introduction

Enterobacteriaceae are ubiquitous in nosocomial settings and can play important roles in the burden of disease in the urinary tract, lower respiratory tract, intra-abdominal organs, and skin infections. The most relevant members of this Gram-negative bacterial group are *Klebsiella pneumoniae*, *Escherichia coli*, and *Enterobacter* spp. The severity of the infections is intensified by the resistance level to most available antibiotics, including carbapenem and cephalosporins [1]. Special attention to *Enterobacter* is due to the intrinsic resistance to ampicillin and broad-spectrum cephalosporin which reduces the alternatives for antibiotic treatments. *Enterobacter* belongs to the ESKAPE group (acronym for *Enterococcus faecium*, *Staphylococcus aureus*, *Klebsiella pneumoniae*, *Acinetobacter baumannii*, *Pseudomonas aeruginosa*, and *Enterobacter* spp), related worldwide to severe nosocomial infections with a highly increasing multi-drug resistance. A problem of epidemiological importance is the presence of AmpC-producing strains together with BLEE-encoding plasmids and colistin co-resistance in *E. cloacae* complex (ECC) strains**[2–4]**. The continuous emergence of multi-drug resistant strains have hampered the effectiveness of antibiotic treatments. Therefore, one possible approach to address this problem is the use of bacteriophages. In itself, the

Formatted: Indent: First line: 0.5"

Formatted: English (United States)

magnitude of the impact of phage predation on pathogenic bacteria has been demonstrated. In recent years, phage therapy has been successfully applied to *E. coli* O157:H7, causative agent of hemorrhagic colitis and hemolytic uremic syndrome **[5–7]**. The use of phages has also been studied in the treatment of peritoneal infections, otitis externa, in the eradication of *P. aeruginosa* in patients with cystic fibrosis [8,9], in the treatment of urinary infections or osteomyelitis caused by *S. aureus* [10]. The favorable effects of phage therapy may even exceed its antibacterial activity. A surprising number of data has demonstrated the *in vivo* activity of phages such as the adenovirus growth suppression mediated by bacteriophage T4 [11] or development inhibition of *P. aeruginosa* by phage Pf4 [12].

Formatted: English (United States)

The principal advantage of employing phages in therapy and biological control lies in their specificity towards bacterial targets. Those that do not infect commensal bacteria can be selected, therefore posing no threat to humans and animals. Their widespread presence in nature, their versatility, and their inherent resilience have elevated their status from mere research organisms to potentially viable and indispensable tools in the battle against growing antimicrobial resistance. However, the transfer of resistance and virulence genes, and other genes associated with negative outcomes through bacteriophages, constituted to an entire research domain aimed at mitigating any potential adverse impacts [13,14]. For these reasons, this study is aimed to biologically and genetically isolate and characterize a phage against the *E. cloacae* complex.

Methods

Bacterial Strains and Growth Conditions.

The host strain ATCC 23355 of *E. cloacae* complex used for the isolation and proliferation of the phage was the same as previously reported [15]. In addition, *S. aureus, E. coli, E. aerogenes, E. cloacae, and P. aeruginosa*, provided by the Human Microbiology Department, Faculty of Medicine, Universidad de Panamá; Hospital Santo Tomas, and Complejo Hospitalario Dr. Arnulfo Arias Madrid, were used for host range determination. The *E. cloacae* host strain, along with twenty-seven clinical isolates of *E. cloacae*, were tested for antibiotic susceptibility using the Vitek II Automated Microbiological System (BioMérieux, Marcy l'Étoile, France) (results shown in Supplementary Table 1). The breakpoints were applied according to the Clinical and Laboratory Standards Institute (CLSI) guidelines [16].

Ethics Statement

The Bioethics Committee of the Universidad de Panamá with the endorsement of the Dirección General de Salud Pública of the Ministerio de Salud (Ministry of Health), approved this study (Sequential: 617).

Phage i~~I~~solation.

The Enterobacter phage vB_EcRAM-01 was isolated from waters of Rio Abajo river in Panama City, Panamá (coordinates, 9°00′55.2″N 79°29′25.6″W), using *E. cloacae* strain ATCC 23355 as a propagation host, which was grown previously in tryptic soy broth at 37°C for 24 h. Phage isolation, propagation, and purification were performed using the soft-agar overlay method [17]. Briefly, **phage purification process involved serial dilution of filtrates exhibiting lytic activity against *E. cloacae*. Specifically, 100 µL of the phage dilution was mixed with an overnight culture of the host bacterium in 3 mL of soft agar (TSB with 0.4% agar), poured onto TSA plates, and incubated at 37 °C for 24 h. The phage plates, selected based on size and clarity, were transferred to microtubes containing 1 mL of nanopure water and the whole process was repeated at least three times to confirm that the isolated phages were descendants of a single virion.**

Formatted: Highlight

Formatted: Font: 10 pt, Highlight

Formatted: Highlight

Formatted: Font: 10 pt, Highlight

Host r~~R~~ange of vB_EcRAM-01 against c~~C~~linical *E. cloacae* i~~I~~solates: Spot t~~T~~est and e~~E~~fficiency of p~~P~~lating (EOP).

Clinical isolates of *E. cloacae*, *S. aureus*, *E. coli*, *P. aeruginosa*, and *E. aerogenes*, along with *E. cloacae* and *E. aerogenes* ATCC reference strains, were cultured until reaching the exponential growth phase (OD_{600nm} 0.6). Subsequently, 200 - 500 µL of each culture were mixed with 3 mL of top agar containing 10 mM $CaCl_2$ and plated onto a 1.5% nutrient agar plate. After solidification, a single droplet (10 µL) from each phage dilution (10^{-1} to 10^{-8}) was applied onto the agar [18].The droplets were allowed to dry for 10 min and plates were incubated at 37°C for 24 h - 48 h. A phage titer in PFU/mL was calculated for each strain tested [19]. Phage virulence was assessed using the efficiency of the plating (EOP) method. The EOP was determined by dividing the phage titer at the final dilution on the test strain by the phage titer on its original isolation strain. Based on these calculations, phages were categorized into highly virulent (0.1 < EOP > 1.00), avirulent but still active (EOP < 0.001), or completely avirulent (no plaques were observed).

Morphological characterization

The isolated phage underwent examination using transmission electron microscopy (TEM). A formvar-coated grid (400 mesh copper grid) was utilized, onto which a drop of 10^9 PFU/mL phage stock was applied. Subsequently, negative

Formatted: Indent: First line: 0.5"

staining was performed with 2 % phosphotungstic acid (pH 7.2) for 5 minutes, and excess stain was eliminated with filter paper. Electron micrographs were captured at varying magnifications using a JEOL JEM-1011 transmission electron microscope. (20).

Effects of UV l~~L~~ight, t~~T~~emperature and pH on the v~~V~~iability of p~~P~~hage vB_EcRAM-01

Purified vB_EcRAM-01 was exposed to different physicochemical conditions (UV light, temperature and pH) Suspensions were exposed to ultraviolet light at a distance of 50 cm for 10, 20, 40, 60, 70, and 90 s. To determine the effect of temperature, phage suspensions were placed at 25 ºC, 37 °C, 50 ºC and 70 ºC, respectively. pH stability was determined by mixing assay vB_EcRAM-01 phage samples resuspended in SM-gelatine buffer (composed of 5.8 g NaCl, 2 g MgSO4.7H20, 50 mL TrisHCl 1M (pH 7.5), and 5 mL gelatin 2% (w/v) with buffer solutions ranging from pH 2 to pH 14. Phage viability was determined by counting the number of lysis PFU using the double-layer agar technique and the experiments were carried out in triplicates [20,21].

Formatted: Indent: First line: 0.5"

Bacterial reduction assay

Bacteriolytic activity was evaluated using a spectrophotometer, targeting *E. cloacae* ATCC 23355. The bacterial strain's overnight culture, adjusted to 1.0 OD_{600nm}, served as the inoculum. A concentration of 8 log PFU/mL was tested with appropriate phage dilutions. Each tube, including negative and positive controls, contained 20 mL of TSB. Experimental tubes received an additional 200 µL of the phage dilution, followed by the addition of 1000 µL of bacterial culture to each tube (excluding the negative control). Incubation was carried out for 180 minutes at 37 °C, with measurements at 600 nm taken every 15 minutes. The experiment was conducted in triplicates (23).

Formatted: Indent: First line: 0.5"

Bacteriophage DNA i~~I~~solation and g~~G~~enome s~~S~~equencing

The QIAamp MinElute Virus Spin kit (QIAGEN, Valencia, CA) was used to extract DNA, according to the manufacturer´s instructions. The purity was assessed spectrophotometrically at 260 and 280 nm, using a BioSpec-nano Micro-volume UV-Vis Spectrophotometer (Shimadzu, Kyoto, Japan).

Formatted: Indent: First line: 0.5"

The DNA libraries were prepared using the Nextera XT DNA Library Preparation Kit (Illumina, San Diego, CA, USA) through fragmentation, PCR amplification, PCR cleanup, and library normalization. The genome was sequenced at the Genomics and Proteomics Research Department of ICGES using the MiSeq Illumina sequencer (2 x 250 bp paired-end reads) (ThermoFisher Scientific Waltham, Massachusetts USA). Raw reads (629,372 reads in total) were checked for quality using FastQC (www.bioinformatics.Babraham.ac.UK/projects/fastqc) and trimmed using Timmomatic v0.39~~–~~ [22].

~~The genome was sequenced at the Genomics and Proteomics Research Department of ICGES using the MiSeq Illumina sequencer (2 x 250 bp paired-end reads) (ThermoFisher Scientific Waltham, Massachusetts USA).~~

Sodium dodecyl sulphate polyacrylamide gel electrophoresis (SDS-PAGE).

SDS-PAGE was carried out in 10% resolving gel in standard Tris–glycine chamber buffer at a constant current of 20 mA. A molecular weight standard (Precision Plus Protein Dual Xtra - Bio-Rad) was included. Gels were stained with Coomassie blue (Sigma, Ge) and images were recorded in a benchtop visible-UV transilluminator (BioDoc 220 imaging system).

Genome a~~A~~nnotations, c~~C~~omparative g~~G~~enomics and p~~P~~hylogenetic a~~A~~nalyses.

The genome was assembled through SPAdes v3.15.1 [23], resulting in a single contig with 8.96-fold coverage [23]. We mapped the reads against the contig obtained using Bowtie v0.9.6 (http://bowtie-bio.sourceforge.net/index.shtml) to corroborate the sequence ends [24]. The phage genome annotation was performed using RASTk v2.0 **[25–27]**. Gene calling was performed through GeneMarkS v4.28 (http://exon.gatech.edu/GeneMark/genemarks.cgi) [28], and Glimmer v3.02 (http://ccb.jhu.edu/software/glimmer/index.shtml) [29,30].

Several methods were used to annotate the ORF functions, including the protein basic local alignment search tool (blastp) on the NCBI server (https://blast.ncbi.nlm.nih.gov/Blast.cgi) based on the search of the non-redundant protein sequence database [31], Hhpred using structural/domain database (https://toolkit.tuebingen.mpg.de/tools/hhpred) **[32–35]** and HMMER v2.41.1 through HMM database [36,37]. (https://toolkit.tuebingen.mpg.de/tools/hhpred). Using a non-redundant protein matrix, employing Markov hidden model profiles [38], the annotations were manually curated using the Geneious v9.1.8

Formatted: Font: (Default) Times New Roman, Font color: Auto, Not Expanded by / Condensed by , Highlight

Formatted: Justified

Formatted: Highlight

Formatted: Font: (Default) Times New Roman, Bold, Font color: Auto, Not Expanded by / Condensed by , Highlight

Formatted: Highlight

Formatted: p, Indent: First line: 0.5", Space Before: 20 pt, After: 20 pt, Line spacing: Double

Formatted: Highlight

Formatted: Font: Times New Roman, 10 pt, Font color: Auto, Highlight

Formatted: Font: Times New Roman, 10 pt, Font color: Auto, Highlight

Formatted: Highlight

Formatted: Font: Times New Roman, 10 pt, Font color: Auto, Highlight

Formatted: Highlight

Formatted: Font: Times New Roman, 10 pt, Font color: Auto, Highlight

Formatted: Highlight

Formatted: Font: Times New Roman, 10 pt, Font color: Auto, Highlight

Formatted: Font: Bold

Formatted: Indent: First line: 0.5"

Formatted: English (United States)

platform employing default parameters. Identification of transfer RNAs was performed using tRNA scan-SE v2.07 (http://lowelab.ucsc.edu/tRNAscan-SE/) and ARAGORN v1.2.41 [39] software (http://www.ansikte.se/ARAGORN/). Comparative analysis between genomes was performed using Mauve progressive alignment v2.3.1 [40] (http://darlinglab.org/mauve/mauve.html) employing default parameters.

Pairwise comparison of nucleotide sequences was performed using the Genome-BLAST Distance Phylogeny (GBDP) method of the VICTOR (Virus Classification and Tree Building Online Resource) platform [41] (https://ggdc.dsmz.de/victor.php), in the recommended environments for prokaryotic viruses. The resulting intergenomic distances were used to infer a branch-supported balanced minimum evolution tree using FASTME, including SPR post-processing [42] using D6 formula. Branch support was inferred from 100 pseudo-bootstrap replicates each. Trees were rooted at the midpoint [43], visualized using FigTree; personalized with taxon boundaries at species, genus, and family scales; and estimated with the iTOL program [44] (https://itol.embl.de/). The recommended clustering threshold is thirthy nine [41] and an F-value (fraction of links required for cluster fusion) of 0.5 [41].

Determination of taxonomy and viral replication cycle type was achieved using PATRIC v3.6.9 [45] (https://patricbrc.org/) and PhageAI (https://phage.ai/) server [46]. The genome comparison between Enterobacter phage vB_EcRAM-01 and close relatives was performed using EasyFig v2.2.2 [47]. At the end, the intergenomic similarities amongst complete viral genomes were calculated through the VIRIDIC web tool [48] with blastn default settings.

Statistical aAnalysis.

Statistically significant differences were determined using ANOVA. Differences in group means were compared using Tukey's HSD test. A P-value below 0.05 was considered statistically significant. Statistical analysis was conducted using R version 4.3.1 [49].

Formatted: Indent: First line: 0.5"

Results

Bacteriophage iIsolation, hHost rRange aAnalysis and mMorphology cCharacterization

In order to evaluate the host specificity of vB_EcRAM-01, a comprehensive assessment was conducted involving a total of thirty-three strains: two ATCC strains alongside a diverse selection of, both, Gram-negative and Gram-positive clinical isolates (Fig 1).

Fig 1. Host range specificity and efficiency of plating. The strain code followed by the efficiency value are shown in parentheses. Color range code associated with EOP is indicated on the right.

Notably, vB_EcRAM-01 exhibited the capacity to infect all the *E. cloacae* isolates with varying degrees of efficacy while displaying negligible antimicrobial activity (efficiency < 0.10) against the other species tested, which included *E. coli*, *E. aerogenes, A. baumannii, S. aureus*, and *P. aeruginosa*. Upon infecting the reference strain, the clear plaque-forming units (PFUs), characterized by smooth perimeters, measured within the range of 3 mm to 7 mm in diameter, a hallmark indicative of its lytic phage attributes (S1 Fig). TEM micrographs of the phage showed a morphology typical of a myovirus, a phage with a tail, belonging to the class *Caudoviricetes*. The size measurements of its icosahedral head are about 104 x 76 nm, and its contractile tail is approximately 110 nm (S2 Fig). Previously, phage morphology was sufficient to classify them taxonomically, but nowadays bioinformatics tools are used to perform similarity analysis and group them by evolutionary distances.

Environmental stress resistance: stability against UV light, temperature and pH

Exposure to UV light (365 nm) decreased the infectivity of phage vB_ECRAM-01 over time. Results showed an exponential decay resulting in complete inactivation when incubated for more than one minute (Fig 2A).

Fig 2. Stability of vB_ECRAM-01 to (A) UV radiation, (B) temperature, and (C) pH. Data is expressed as mean ± standard error. Treatments with the same letters in panel (C) are not significantly different (Tukey's HSD test, p>0.05).

The phage exhibited lytic activity when subjected to temperatures ranging from 25 °C to 50 °C, even after being incubated for 90 minutes (Fig 2B). However, when exposed to 70 °C, the phage became completely inactivated after only 20 min of incubation and, at 80 °C, it lost its infectivity entirely (data not shown for clarity).

The stability of vB_EcRAM-01 was determined at various pH values. It maintained its infectivity in a pH range between pH 7.5 and pH 11. The best conditions were found between pH 9 and 10 (Fig 2C). Incubation under acidic pH resulted in a

Formatted: Highlight

Formatted: Indent: First line: 0"

Formatted: Font: Bold, Highlight

Formatted: Normal, Left, Space After: 0 pt

Formatted: Font: (Default) Times New Roman, 10 pt, Font color: Auto, Highlight

Formatted: Font: (Default) Times New Roman, 10 pt, Font color: Auto

Formatted: Indent: First line: 0.5"

significant reduction of viral proliferation. The phage was not able to replicate in a pH greater than 11 or less than 3. Overall, the best activity was found in alkaline media.

Bacteriolytic activity

The evaluated phage demonstrated a robust, complete bacteriolytic effect in the initial 105 minutes of the assay. Following this, the OD curve started to rise due to an increase in bacterial cell numbers. This trend persisted in subsequent measurements, although the rate of increase was lower than that observed in the positive control (Fig 3).

Fig 3. Effect of vB_EcRAM-01 on the growth of the E. cloacae complex. The light blue line indicates the bacterial concentration without being infected with the virus at 37°C, showing an absorbance of 1.6. On the other hand, the orange line shows the concentration of the bacteria being infected from the beginning, with a reduced absorbance of up to 0.77 (ANOVA F = 613.62. g.l = 1 and p<0.0001).

Comparative genomics and phylogenetic analysis

The genomic attributes of phage vB_EcRAM-01 were delineated as follows: the genome spans 178,477 base pairs with a 45.0 % of GC content, hosting a total of 293 genes. Among these, 86 genes were implicated in anticipated functions encompassing morphology, inactivation, cellular adsorption, DNA injection, and host lysis. Through a UniProt database blast search, we were able to identify to structural proteins, one sharing 97% homology with the Major capsid protein (ID: A0A1B1IX95) of Citrobacter phage vB_CfrM_CfP1, having a mass of 56.37 kDa [50] while the other protein exhibits 96.7% homology with the Baseplate wedge subunit protein (ID: M9V1F2) from Escherichia phage Lw1, with a mass of 38.01 kDa [51]. In fact, analysis by polyacrylamide gel electrophoresis revealed two prominent protein bands with molecular masses close to 50 and 37 kDa, respectively (S3 Fig). In bacteriophages possessing both capsid and tail structures, SDS-PAGE typically reveals multiple proteins, including capsid, tail, and baseplate proteins. Capsid proteins form the outer shell encasing the phage genome, while baseplate proteins are situated at the tail base, facilitating recognition and binding to specific receptors on host cell surfaces [52]. Hence, it is plausible that the observed proteins serve these functions; however, precise identification through LC-MS/MS analysis would provide a more definitive comparison [53].

Formatted: Font: (Default) Times New Roman, Bold
Formatted: Font: (Default) Times New Roman, 10 pt, Highlight
Formatted: Normal, Space After: 0 pt, Line spacing: Double
Formatted: Font: (Default) Times New Roman, 10 pt, Bold, Highlight
Formatted: Font: Bold, Highlight
Formatted: Font: (Default) Times New Roman
Formatted: Indent: First line: 0.5", Line spacing: Double
Formatted: Font: (Default) Times New Roman, Not Highlight
Formatted: Font: (Default) Times New Roman
Formatted: Highlight
Formatted: Highlight
Formatted: Highlight
Formatted: Highlight
Formatted: Highlight
Formatted: Font: (Default) +Body (Calibri), 10 pt, Font color: Auto, Pattern: Clear, Highlight
Formatted: Highlight
Formatted: Font: (Default) +Body (Calibri), 10 pt, Font color: Auto, Pattern: Clear, Highlight
Formatted: Highlight
Formatted: Highlight
Formatted: Highlight
Formatted: Highlight
Formatted: Highlight
Formatted: Highlight

Remarkably, the genome of vB_EcRAM-01 was lacking discernible genes associated with resistance, virulence, or allergenicity. The genomic blueprint encodes two transfer RNAs (tRNAs), one corresponding to methionine (cat) and the other to glycine (tcc). A pertinent insight is the substantial sequence identity—90.11%—shared with other phages (Fig~~.~~ 4) [15].

Fig 4. Comparison of Enterobacter phage vB_EcRAM-01 genome with close relatives. Color scales represent the percentage of nucleotide identity between regions obtained by blastn.

To assign a taxonomic classification to phage vB_EcRAM-01, a phylogenetic tree based on whole-genome was constructed, and intergenomic similarities were calculated between the closely related genomes reported in GenBank. The phylogenetic tree and intergenomic similarity heatmap revealed that phage vB_EcRAM-01 is a new member of the *Pseudotevenvirus* genus within the *Straboviridae* family.

The GBDP phylogenetic tree using the D6 formula with an average support of 31%, identifying ten and nine clusters, respectively, at the genus level. At the family level, there were five and six clusters, respectively (Fig~~.~~ 5).

Fig 5. Phage vB_EcRAM-01 genomic and phylogenetic analyses. Genome Blast Distance Phylogeny (GBDP)-based phylogenetic tree of vB_EcRAM-01 and 28 related Straboviridae members created by iTOL v6.0 platform. The Enterobacter phage vB_EcRAM-01 belongs to the genus Pseudotevenvirus and is a new phage species.

Nucleotide identity analysis (ANI) was done on the 28 genomes with the highest homology in the GenBank database using VIRIDIC. According to the results of the heat mapping, phage vB_EcRAM-01 shares less than 95% nucleotide identity with previously reported genomes, thereby establishing it as a novel species within the Family *Straboviridae*, genus *Pseudotevenvirus*, according to the genus demarcation criteria (>70% nucleotide identity over the entire genome length) recommended by the ICTV (Fig~~.~~ 6~~5~~).

Fig 6. Taxonomic classification of Enterobacter phage vB_EcRAM-01. A heatmap created by VIRIDIC based on intergenomic similarities between viral genomes, with the proportion of similarity shown by the color scale.

Comparative BLASTn analysis of the complete genome of vB_EcRAM-01 against phages in the GenBank database revealed that vB_EcRAM-01 has a high degree of coverage and identity with the Enterobacter phages ENC22 (OL355128.1), ENC7 (OL355125.1), ENC25 (OL355127.1), EBPL (MT341500.1). In VIRIDIC analysis, the phages shared collinear gene blocks that are identical in length between the two genomes. In comparison, other blocks revealed a high degree of similarity

Formatted: Font: (Default) Times New Roman

Formatted: Font: (Default) Times New Roman

Formatted: Font: (Default) Times New Roman, Not Bold

Formatted: Font: (Default) Times New Roman

Formatted: Font: (Default) Times New Roman, 10 pt, Bold, Font color: Auto, Pattern: Clear, Highlight

Formatted: Normal, Space After: 0 pt, Line spacing: Double

Formatted: Font: (Default) Times New Roman, Bold, Highlight

Formatted: Font: (Default) Times New Roman, 10 pt, Bold, Font color: Auto, Pattern: Clear, Highlight

Formatted: Indent: First line: 0.5"

Formatted: Font: (Default) Times New Roman, 10 pt, Font color: Auto, Highlight

Formatted: Font: Bold, Highlight

Formatted: Font: (Default) Times New Roman, 10 pt, Bold, Font color: Auto, Highlight

Formatted: Font: (Default) Times New Roman, 10 pt, Font color: Auto

Formatted: Font: (Default) Times New Roman, 10 pt, Bold, Font color: Auto, Highlight

Formatted: Normal, Space After: 0 pt, Line spacing: Double

Formatted: Indent: First line: 0.5"

between this phage and others, including Cronobacter phage vB_CsaM IeE (NC_048646.1), Cronobacter phage vB_CsaM IeN (KX431560.1), Citrobacter phage Margaery (KT381880.1), and myoviral members of the subfamily *Tevenvirinae*.

Discussion

In Panama, seven out of the eight bacterial pathogens responsible for 80% of the reported infections concerning the emergence of resistance in a hospital level (ESKAPE) has been identified: *S. aureus* (16%), *Enterococcus* spp (14.4%), *E. coli* (12%), coagulase-negative *Staphylococcus* (11%), *K. pneumoniae* and *K. oxytoca* (8%), *P. aeruginosa* (8%), and *Enterobacter* spp. (5%). Several microorganisms developed antibiotic resistance to the more common antibiotics to treat these bacterial infections like piperacillin-tazobactam, cephalosporins, nitrofurans, and carbapenems [54,55].

Formatted: Indent: First line: 0.5"

Therefore, in comparison to the usage of antibiotics, phage therapy is a viable option. While scientific publications on phage isolation have increased in recent years, only a small number of phages have been commercialized [56]. One of the primary reasons is a lack of adequate characterization, which is required to detect and mitigate the hazards associated with phage therapy [57,56]. Moreover, the biological characteristics of a phage determines its stability and therefore, its optimum administration forms [57].

As a first step, vB_EcRAM-01was subjected to environmental stress tests, such as temperature, UV radiation and pH, factors that play a role in phage survival [58,59]. The results indicate that vB_EcRAM-01 is stable at temperatures ranging from 25°C to 50°C, although its replication rate is highest at 37°C. This aligns with findings observed in Myoviridae phages [60,61] exhibiting a comparable stability profile within a broad temperature range. However, it deviates from the EBP phage, known for its resilience across a wider temperature span [62]. Notably, inactivation under UV light transpires rapidly, underscoring the necessity of safeguarding possible clinical applications from sunlight exposure. Interestingly, vB_EcRAM-01 maintains its infectivity across expansive pH and temperature ranges, implying potential robustness within the human body. Enhancements through administration systems designed to shield the phage from gastrointestinal pH could potentially further increase its utility and stability in therapeutic applications [63].

The primary objective of this study was to investigate the host specificity (host range) of phage vB_EcRAM-01 concerning isolates within the *E. cloacae* complex. Accurate identification of the host bacteria's specificity for bacteriophage vB_EcRAM-01 is crucial for a comprehensive characterization of this bacteriophage in order to evaluate a potential therapeutic

use. Our observations indicate that this bacteriophage displayed reactivity ~~mainly~~only with its host strain. These results are in line with findings reported for phages TSE1, TSE2, and TSE3 [64]. *E. coli* displayed some sensitivity but did not exhibit complete lysis. Exceptions to this pattern were observed with myophages *Enterobacter* phage ENC22, *Enterobacter* phage ENC7, and *Enterobacter* phage ENC25, which demonstrated the ability to infect diverse *Enterobacter* species [65]. ~~Our observations indicate that this bacteriophage displayed reactivity mainly with its host strain. These results are in line with findings reported for phages TSE1, TSE2, and TSE3 (60). *E. coli* displayed some sensitivity but did not exhibit complete lysis. Exceptions to this pattern were observed with myophages *Enterobacter* phage ENC22, *Enterobacter* phage ENC7, and *Enterobacter* phage ENC25, which demonstrated the ability to infect diverse *Enterobacter* species (61).~~

Apart from meeting the requirements of being innocuous to normal microbiota, therapeutic use involves the employment of fully lytic phages that lack undesired virulence factor genes or antibiotic resistance genes [66]. The genomic assessment of the phage vB_EcRAM-01 confirmed its status as a strictly lytic phage according to the PhageAI platform. The genome contained genes coding for holins and endolysins, offering several advantages. These enzymes collaborate to facilitate bacterial lysis, enabling the release of newly assembled viral particles and the termination of the bacterial host [67]. Additionally, holin proteins play a crucial role in regulating the timing of lysis, ensuring that it occurs at the appropriate stage of the phage life cycle [68]. An important aspect to note is that resistance to endolysins has not been reported to date [69]. This suggests a higher probability of success when employing phages containing endolysins. Besides, it lacked genes associated with lysogeny, such as integrases, transposons, and excitons [70].

The evolutionary context designated it as a T4-type myophage spanning a genome length of 178,477 base pairs. Computational analysis further classified it as a viral entity within the Class *Caudoviricetes*, *Strabovviridae* family, and *Pseudotevenvirus* genus [70]. This type of virus is characterized by the presence of tRNA. Due to the compact nature of phage genomes, which constrains their capacity to utilize host resources fully, these genetic elements may serve as compensation for compositional differences between phages and hosts. They have the potential to supplement the translation machinery with their genetic information, thereby improving fitness and fortifying virulence [71].

By scanning the GenBank database for homologous sequences, a 92% sequence homology with myophage EBPL was observed, a phage that also infects *E. cloacae* [62], and 99.16% sequence homology with myophages ENC7, ENC25, and ENC22 [65].

Formatted: Highlight

Formatted: Highlight

Formatted: Highlight

Formatted: Highlight

Formatted: Highlight

Formatted: Highlight

Formatted: Highlight

Formatted: Highlight

Formatted: Indent: First line: 0.5"

According to genotypic characteristics [72], vB_EcRAM-01, like an EBP phage, is a myovirus-like phage [62]. The vB_EcRAM-01 virus was termed a bacterial infecting virus isolated from Rio Abajo waters that suppressed *E. cloacae*, a myophage, according to the informal phage nomenclature criteria [73–75].

Formatted: Font: Not Bold, English (United States)

In this study, the genome-wide syntenic analysis indicated that both phages had comparable rearrangements but differed slightly in nucleotide sequence. Additionally, a phylogenetic study of whole-genome sequences showed that both phages diverged from a common ancestor but experienced many nucleotides substitution events since then [76]. Given that EBPL was previously described as a singleton phage in the myophage phylogenetic tree [77], phylogenetic and nucleotide identity analyses indicated that vB_EcRAM-01 might represent a previously unreported species.

Therefore, vB_EcRAM-01 is physiologically and molecularly stable under a wide temperature and pH range. However, it has a restricted host range. Although it had intense lytic activity against the *E. cloacae* complex, isolation was deemed necessary for more efficient eradication.

In Conclusion, this study identified a lytic bacteriophage. The characterization enhances comprehension of this novel phage, and through its phylogenetic relatives, it may exhibit a myovirus-like morphology, as demonstrated by insights from next-generation sequencing. This sequencing elucidates that vB_EcRAM-01 aligns with the Myoviridae family in accordance with the conventional taxonomic classification [78]. According to the phylogenetic analysis previously described, this virus is a new member of the family *Straboviridae* and the genus *Pseudotevenvirus* according to the new classification [79]. The vB_EcRAM-01 strain is also a viable candidate for many clinical applications, including phage therapy and biologic control or decontamination, due to its host specificity, the genetic link, and lack of lysogenic genes.

Formatted: Indent: First line: 0.5"

Data availability statement

The datasets presented in this study can be found in online repositories. The names of the repository/repositories and accession number(s) can be found at https://www.ncbi.nlm.nih.gov/genbank/ , OL551674, SAMN23426491, SRR18344380.

Author Contributions

EEVB and JPGG drafted the manuscript, while JRMS conducted the statistical analysis of the results. NCC and CCHQ, along with JQA, AAM and AOMT, contributed to data analysis and manuscript revisions. EEVB, JQA, AAM and AOMT secured project funding. All authors reviewed and approved the submitted manuscript.

Acknowledgments

~~This work was funded in part by the Sistema Nacional de Investigación (SNI) of the Secretaría Nacional de Ciencia, Tecnología e Innovación (SENACYT, Panama), through grants 87-2019, 81-2019 and FID18-044, and also from the Water, Protected Areas and Wildlife Trust of the Ministry of Environment of Panama through grant 026-45-2019 and grant 111130406 from the Ministry of Economy and Finance of Panama.~~ Host bacterial strains were provided by José E. Moreno, MSc., of the Microbiology Division of Laboratorio Central de Referencia en Salud Pública, Instituto Conmemorativo Gorgas de Estudios de Salud (ICGES). We extend our gratitude to the staff of the Microbiology Department at the Clinical Laboratory of Hospital Santo Tomas for providing us with the clinical bacteria strains.

Additionally, we would like to thank Claudia González, MSc, Jessica Gondola, MSc, Ambar Moreno, MSc, and Oris Chavarria, MSc, of the Research Department of Genomics and Proteomics of ICGES for providing us with NGS Illumina sequencing. Furthermore, we would like to express our gratitude to Leandra Gómez, MSc, of the Department of Pharmacology at the Faculty of Medicine, Universidad de Panamá, for her invaluable contribution in reviewing the bioethical considerations within this research. Also, thank you to Bs. Tatiana A. Martínez González for her contribution to the English revision of the present work.

Bibliography

1. WHO. Global priority list of antibiotic-resistant bacteria to guide research, discovery, and development of new antibiotics. 2017.

2. Qian W, Fu Y, Liu M, Zhang J, Wang W, Li J, et al. Mechanisms of Action of Luteolin Against Single- and Dual-Species of Escherichia coli and Enterobacter cloacae and Its Antibiofilm Activities. Appl Biochem Biotechnol. 2021;193: 1397–1414. doi:10.1007/s12010-020-03330-w

3. Davin-Regli A, Lavigne J-P, Pagès J-M. Enterobacter spp.: Update on Taxonomy, Clinical Aspects, and Emerging Antimicrobial Resistance. Clin Microbiol Rev. 2019;32. doi:10.1128/CMR.00002-19

Formatted: English (United States)

4. Mezzatesta ML, Gona F, Stefani S. *Enterobacter cloacae* complex: clinical impact and emerging antibiotic resistance. Future Microbiology. 2012;7: 887–902. doi:10.2217/fmb.12.61

5. Amarillas L, Rubí-Rangel L, Chaidez C, González-Robles A, Lightbourn-Rojas L, León-Félix J. Isolation and Characterization of phiLLS, a Novel Phage with Potential Biocontrol Agent against Multidrug-Resistant Escherichia coli. Frontiers in Microbiology. 2017;8. Available: https://www.frontiersin.org/articles/10.3389/fmicb.2017.01355

6. López-Cuevas O, Castro-Del Campo N, León-Félix J, Valdez-Torres B, Chaidez C. Evaluation of Bacteriophage Av-08 for Simultaneous Biocontrol of Salmonella Montevideo and Escherichia Coli O157:h7 in Experimentally Contaminated Chicken Skin. Journal of Food Safety. 2012;32: 305–310. doi:10.1111/j.1745-4565.2012.00381.x

7. Sharma M, Patel JR, Conway WS, Ferguson S, Sulakvelidze A. Effectiveness of Bacteriophages in Reducing Escherichia coli O157:H7 on Fresh-Cut Cantaloupes and Lettuce. Journal of Food Protection. 2009;72: 1481–1485. doi:10.4315/0362-028X-72.7.1481

8. Morello E, Saussereau E, Maura D, Huerre M, Touqui L, Debarbieux L. Pulmonary Bacteriophage Therapy on Pseudomonas aeruginosa Cystic Fibrosis Strains: First Steps Towards Treatment and Prevention. Aziz R, editor. PLoS ONE. 2011;6: e16963. doi:10.1371/journal.pone.0016963

9. Debarbieux L, Leduc D, Maura D, Morello E, Criscuolo A, Grossi O, et al. Bacteriophages Can Treat and Prevent *Pseudomonas aeruginosa* Lung Infections. J INFECT DIS. 2010;201: 1096–1104. doi:10.1086/651135

Formatted: English (United States)

10. Abedon ST. Commentary: Phage Therapy of Staphylococcal Chronic Osteomyelitis in Experimental Animal Model. Front Microbiol. 2016;7. doi:10.3389/fmicb.2016.01251

11. Przybylski M, Dzieciątkowski T, Borysowski J, Międzybrodzki R, Górski A. Inhibitory Effects of Bacteriophage Preparations on Adenoviral Replication. INT. 2019;62: 37–44. doi:10.1159/000500540

12. Casto A, Hurwitz A, Kou K, Mansour G, Policke R, Schmidt A, et al. Bacteriophages: The Answer to Antibiotic Resistance? James Madison Undergraduate Research Journal. 20163: 36–41.

13. Hassan AY, Lin JT, Ricker N, Anany H. The Age of Phage: Friend or Foe in the New Dawn of Therapeutic and Biocontrol Applications? Pharmaceuticals. 2021;14: 199. doi:10.3390/ph14030199

14. Häusler T. Viruses vs. Superbugs. London: Palgrave Macmillan UK; 2006. doi:10.1007/978-0-230-55228-9

15. Victoria-Blanco EE, González-Gómez JP, Quiroz E, Martínez AA, González C, Castro del Campo N, et al. Complete Genome Sequence of Enterobacter Phage vB_EcRAM-01, a New Pseudotevenvirus against the Enterobacter cloacae Complex. Microbiology Resource Announcements. 10 May 2022: 1–3. doi:10.1128/mra.00045-22

Formatted: English (United States)

16. CLSI. CLSI Performance Standards for Antimicrobial Susceptibility Testing, CLSI Guideline M100. 28th ed. Wayne, PA: Clinical and Laboratory Standards Institute; 2018.

17. Adams MH, Anderson ES, Kellenberger E. Bacteriophages. New York: Interscience Publishers; 1959.

18. Khan Mirzaei M, Nilsson AS. Isolation of Phages for Phage Therapy: A Comparison of Spot Tests and Efficiency of Plating Analyses for Determination of Host Range and Efficacy. Schuch R, editor. PLoS ONE. 2015;10: e0118557. doi:10.1371/journal.pone.0118557

19. Stephenson, Frank H. Calculations for Molecular Biology and Biotechnology. second edition. USA: Elsevier/AP, Academic Press is an imprint of Elsevier; 2010. Available: Vetbiotech.um.ac.ir

20. Flores V. Caracterización de bacteriófagos líticos de Salmonella enterica aislados de muestras de pollos. Universidad Mayor San Marcos. 2017.

Formatted: English (United States)

21. Zaburlin D, Quiberoni A, Mercanti D. Changes in Environmental Conditions Modify Infection Kinetics of Dairy Phages. Food Environ Virol. 2017;9: 270–276. doi:10.1007/s12560-017-9296-2

22. Bolger AM, Lohse M, Usadel B. Trimmomatic: a flexible trimmer for Illumina sequence data. Bioinformatics. 2014;30: 2114–2120. doi:10.1093/bioinformatics/btu170

23. Bankevich A, Nurk S, Antipov D, Gurevich AA, Dvorkin M, Kulikov AS, et al. SPAdes: A New Genome Assembly Algorithm and Its Applications to Single-Cell Sequencing. Journal of Computational Biology. 2012;19: 455–477. doi:10.1089/cmb.2012.0021

24. Langmead B, Trapnell C, Pop M, Salzberg SL. Ultrafast and memory-efficient alignment of short DNA sequences to the human genome. Genome Biology. 2009;10: R25. doi:10.1186/gb-2009-10-3-r25

25. Aziz RK, Bartels D, Best AA, DeJongh M, Disz T, Edwards RA, et al. The RAST Server: rapid annotations using subsystems technology. BMC Genomics. 2008;9: 75. doi:10.1186/1471-2164-9-75

26. Brettin T, Davis JJ, Disz T, Edwards RA, Gerdes S, Olsen GJ, et al. RASTtk: a modular and extensible implementation of the RAST algorithm for building custom annotation pipelines and annotating batches of genomes. Sci Rep. 2015;5: 8365. doi:10.1038/srep08365

27. Overbeek R, Olson R, Pusch GD, Olsen GJ, Davis JJ, Disz T, et al. The SEED and the Rapid Annotation of microbial genomes using Subsystems Technology (RAST). Nucleic Acids Res. 2014;42: D206-214. doi:10.1093/nar/gkt1226

28. Besemer J, Lomsadze A, Borodovsky M. GeneMarkS: a self-training method for prediction of gene starts in microbial genomes. Implications for finding sequence motifs in regulatory regions. Nucleic Acids Research. 2001;29: 2607–2618. doi:10.1093/nar/29.12.2607

29. Delcher AL, Bratke KA, Powers EC, Salzberg SL. Identifying bacterial genes and endosymbiont DNA with Glimmer. Bioinformatics. 2007;23: 673–679. doi:10.1093/bioinformatics/btm009

30. Salzberg SL, Delcher AL, Kasif S, White O. Microbial gene identification using interpolated Markov models. Nucleic Acids Res. 1998;26: 544–548. doi:10.1093/nar/26.2.544

31. Altschul SF, Madden TL, Schäffer AA, Zhang J, Zhang Z, Miller W, et al. Gapped BLAST and PSI-BLAST: a new generation of protein database search programs. Nucleic Acids Res. 1997;25: 3389–3402.

32. Gabler F, Nam S-Z, Till S, Mirdita M, Steinegger M, Söding J, et al. Protein Sequence Analysis Using the MPI Bioinformatics Toolkit. Current Protocols in Bioinformatics. 2020;72: e108. doi:https://doi.org/10.1002/cpbi.108

33. Zimmermann L, Stephens A, Nam S-Z, Rau D, Kübler J, Lozajic M, et al. A Completely Reimplemented MPI Bioinformatics Toolkit with a New HHpred Server at its Core. Journal of Molecular Biology. 2018;430: 2237–2243. doi:10.1016/j.jmb.2017.12.007

34. Meier A, Söding J. Automatic Prediction of Protein 3D Structures by Probabilistic Multi-template Homology Modeling. PLOS Computational Biology. 2015;11: e1004343. doi:10.1371/journal.pcbi.1004343

35. Hildebrand A, Remmert M, Biegert A, Söding J. Fast and accurate automatic structure prediction with HHpred. Proteins: Structure, Function, and Bioinformatics. 2009;77: 128–132. doi:https://doi.org/10.1002/prot.22499

36. Potter SC, Luciani A, Eddy SR, Park Y, Lopez R, Finn RD. HMMER web server: 2018 update. Nucleic Acids Research. 2018;46: W200–W204. doi:10.1093/nar/gky448

37. Finn RD, Clements J, Arndt W, Miller BL, Wheeler TJ, Schreiber F, et al. HMMER web server: 2015 update. Nucleic Acids Research. 2015;43: W30–W38. doi:10.1093/nar/gkv397

38. Hofmann P, Tashman Z. Hidden Markov Models and Their Application for Predicting Failure Events. In: Krzhizhanovskaya VV, Závodszky G, Lees MH, Dongarra JJ, Sloot PMA, Brissos S, et al., editors. Computational Science – ICCS 2020. Cham: Springer International Publishing; 2020. pp. 464–477. doi:10.1007/978-3-030-50420-5_35

39. Laslett D, Canback B. ARAGORN, a program to detect tRNA genes and tmRNA genes in nucleotide sequences. Nucleic Acids Res. 2004;32: 11–16. doi:10.1093/nar/gkh152

40. Darling ACE, Mau B, Blattner FR, Perna NT. Mauve: Multiple Alignment of Conserved Genomic Sequence With Rearrangements. Genome Res. 2004;14: 1394–1403. doi:10.1101/gr.2289704

41. Meier-Kolthoff JP, Göker M. VICTOR: genome-based phylogeny and classification of prokaryotic viruses. Bioinformatics. 2017;33: 3396–3404. doi:10.1093/bioinformatics/btx440

42. Lefort V, Desper R, Gascuel O. FastME 2.0: A Comprehensive, Accurate, and Fast Distance-Based Phylogeny Inference Program. Mol Biol Evol. 2015;32: 2798–2800. doi:10.1093/molbev/msv150

43. Farris JS. Estimating Phylogenetic Trees from Distance Matrices. The American Naturalist. 1972;106: 645–668.

44. Letunic I, Bork P. Interactive Tree Of Life (iTOL) v4: recent updates and new developments. Nucleic Acids Research. 2019;47: W256–W259. doi:10.1093/nar/gkz239

45. Davis JJ, Wattam AR, Aziz RK, Brettin T, Butler R, Butler RM, et al. The PATRIC Bioinformatics Resource Center: expanding data and analysis capabilities. Nucleic Acids Res. 2020;48: D606–D612. doi:10.1093/nar/gkz943

46. Tynecki P, Guziński A, Kazimierczak J, Jadczuk M, Dastych J, Onisko A. PhageAI - Bacteriophage Life Cycle Recognition with Machine Learning and Natural Language Processing. 2020 [cited 11 Apr 2021]. doi:10.1101/2020.07.11.198606

47. Sullivan MJ, Petty NK, Beatson SA. Easyfig: a genome comparison visualizer. Bioinformatics. 2011;27: 1009–1010. doi:10.1093/bioinformatics/btr039

48. Moraru C, Varsani A, Kropinski AM. VIRIDIC—A Novel Tool to Calculate the Intergenomic Similarities of Prokaryote-Infecting Viruses. Viruses. 2020;12: 1268. doi:10.3390/v12111268

49. R Core Team. R: A Language and Environment for Statistical Computing. Vienna, Austria: R Foundation for Statistical Computing; 2023.

50. Oliveira H, Pinto G, Oliveira A, Oliveira C, Faustino MA, Briers Y, et al. Characterization and genome sequencing of a Citrobacter freundii phage CfP1 harboring a lysin active against multidrug-resistant isolates. Appl Microbiol Biotechnol. 2016;100: 10543–10553. doi:10.1007/s00253-016-7858-0

51. Kushkina AI, Tovkach FI, Comeau AM, Kostetskii IE, Lisovski I, Ostapchuk AM, et al. Complete Genome Sequence of Escherichia Phage Lw1, a New Member of the RB43 Group of Pseudo T-Even Bacteriophages. Genome Announcements. 2013;1: 10.1128/genomea.00743-13. doi:10.1128/genomea.00743-13

52. Kutter E, Sulakvelidze A, editors. Bacteriophages: biology and applications. Boca Raton, FL: CRC Press; 2005.

53. Al-Wrafy F, Brzozowska E, Górska S, Drab M, Strus M, Gamian A. Identification and characterization of phage protein and its activity against two strains of multidrug-resistant Pseudomonas aeruginosa. Sci Rep. 2019;9: 13487. doi:10.1038/s41598-019-50030-5

54. Moreno J. Antibiotic resistance profiles in Panama: Trends from 2007 to 2013. PPIJ. 2018;6. doi:10.15406/ppij.2018.06.00200

55. MINSA. 18400_2011_31657_INFORME RESISTENCIA ANTIBIOTICOS-USO RACIONAL -036.pdf. Panama: ICGES; 2015 p. 48.

56. Abedon ST. Detection of Bacteriophages: Phage Plaques. In: Harper DR, Abedon ST, Burrowes BH, McConville ML, editors. Bacteriophages. Cham: Springer International Publishing; 2018. pp. 1–32. doi:10.1007/978-3-319-40598-8_16-1

57. Hyman P, Abedon ST. Bacteriophage: Overview. Reference Module in Biomedical Sciences. Elsevier; 2019. p. B978012801238302506X. doi:10.1016/B978-0-12-801238-3.02506-X

58. Phothichaisri W, Ounjai P, Phetruen T, Janvilisri T, Khunrae P, Singhakaew S, et al. Characterization of Bacteriophages Infecting Clinical Isolates of Clostridium difficile. Front Microbiol. 2018;9. doi:10.3389/fmicb.2018.01701

59. Silva YJ, Costa L, Pereira C, Cunha Â, Calado R, Gomes NCM, et al. Influence of environmental variables in the efficiency of phage therapy in aquaculture. Microbial Biotechnology. 2014;7: 401–413. doi:10.1111/1751-7915.12090

60. Litt PK, Jaroni D. Isolation and Physiomorphological Characterization of Escherichia coli O157:H7-Infecting Bacteriophages Recovered from Beef Cattle Operations. In: International Journal of Microbiology [Internet]. 2017 [cited 9 Jul 2019]. doi:10.1155/2017/7013236

61. Yamaki S, Kawai Y, Yamazaki K. Characterization of a novel bacteriophage, Phda1, infecting the histamine-producing Photobacterium damselae subsp. damselae. Journal of Applied Microbiology. 2015;118: 1541–1550. doi:https://doi.org/10.1111/jam.12809

62. Asif M, Naseem H, Alvi IA, Basit A, Rehman S-. Characterization of a lytic EBP bacteriophage with large size genome against Enterobacter cloacae. APMIS. 2021;129: 461–469. doi:10.1111/apm.13138

63. Dąbrowska K. Phage therapy: What factors shape phage pharmacokinetics and bioavailability? Systematic and critical review. Med Res Rev. 2019;39: 2000–2025. doi:10.1002/med.21572

64. Khawaja KA, Abbas Z, Rehman S ur. Isolation and characterization of lytic phages TSE1-3 against Enterobacter cloacae. Open Life Sciences. 2016;11. doi:10.1515/biol-2016-0038

65. Finney AG, Perry JM, Evans DR, Westbrook KJ, McElheny CL, Iovleva A, et al. Isolation and Characterization of Lytic Bacteriophages Targeting Diverse Enterobacter spp. Clinical Isolates. PHAGE. 2022;3: 50–58. doi:10.1089/phage.2021.0011

66. Soohey RL. Identifyng Genes Essential For Lysogeny in Cluster e Mycobacteriophage Ukulele. The University of Maine. 2017.

67. Pollenz RS, Bland J, Pope WH. Bioinformatic characterization of endolysins and holin-like membrane proteins in the lysis cassette of phages that infect Gordonia rubripertincta. PLOS ONE. 2022;17: e0276603. doi:10.1371/journal.pone.0276603

68. Zhang M, Wang Y, Chen J, Hong X, Xu X, Wu Z, et al. Identification and Characterization of a New Type of Holin-Endolysin Lysis Cassette in Acidovorax oryzae Phage AP1. Viruses. 2022;14: 167. doi:10.3390/v14020167

69. Larpin Y, Oechslin F, Moreillon P, Resch G, Entenza JM, Mancini S. In vitro characterization of PlyE146, a novel phage lysin that targets Gram-negative bacteria. PLOS ONE. 2018;13: e0192507. doi:10.1371/journal.pone.0192507

70. Ríos-Sandoval M. Caracterización Genómica, Morfológica y Replicativa del Bacteriófago ΦXaF18 de Xanthomonas vesicatoria. Centro De Investigación Y Asistencia En Tecnología Y Diseño Del Estado De Jalisco, A. C. 2019.

71. Hatfull GF, Dedrick RM, Schooley RT. Phage Therapy for Antibiotic-Resistant Bacterial Infections. Annu Rev Med. 2022;73: 197–211. doi:10.1146/annurev-med-080219-122208

72. Hatfull GF. Bacteriophage genomics. Current Opinion in Microbiology. 2008;11: 447–453. doi:10.1016/j.mib.2008.09.004

73. Turner D, Kropinski AM, Adriaenssens EM. A Roadmap for Genome-Based Phage Taxonomy. Viruses. 2021;13: 506. doi:10.3390/v13030506

74. Adriaenssens EM, Sullivan MB, Knezevic P, van Zyl LJ, Sarkar BL, Dutilh BE, et al. Taxonomy of prokaryotic viruses: 2018-2019update from the ICTV Bacterial and Archaeal Viruses Subcommittee. Arch Virol. 2020;165: 1253–1260. doi:10.1007/s00705-020-04577-8

75. Adriaenssens E, Brister JR. How to Name and Classify Your Phage: An Informal Guide. Viruses. 2017;9: 70. doi:10.3390/v9040070

76. Leskinen K, Blasdel BG, Lavigne R, Skurnik M. RNA-Sequencing Reveals the Progression of Phage-Host Interactions between φR1-37 and Yersinia enterocolitica. Viruses. 2016;8: 111. doi:10.3390/v8040111

77. Jacquemot L, Bettarel Y, Monjol J, Corre E, Halary S, Desnues C, et al. Therapeutic Potential of a New Jumbo Phage That Infects Vibrio coralliilyticus, a Widespread Coral Pathogen. Front Microbiol. 2018;0. doi:10.3389/fmicb.2018.02501

78. Adams MJ, Lefkowitz EJ, King AMQ, Harrach B, Harrison RL, Knowles NJ, et al. 50 years of the International Committee on Taxonomy of Viruses: progress and prospects. Arch Virol. 2017;162: 1441–1446. doi:10.1007/s00705-016-3215-y

79. Turner D, Shkoporov AN, Lood C, Millard AD, Dutilh BE, Alfenas-Zerbini P, et al. Abolishment of morphology-based taxa and change to binomial species names: 2022 taxonomy update of the ICTV bacterial viruses subcommittee. Arch Virol. 2023;168: 74. doi:10.1007/s00705-022-05694-2

Supplementary figure legends

S1 Fig. Lytic effect of bacteriophage vB_EcRAM-01. According to the double agar plate method, the bacterial lawn of the *E. cloacae* complex is infected by bacteriophages. After purification, lysis PFU measuring approximately 9 mm (shown in the figure), rough-edged and completely clear, were obtained.

S2 Fig. Overall morphology of vB_EcRAM-01. A representative micrograph of a purified sample of the bacteriophage is shown (scale bar: 100nm).

S3 Fig. Electrophoretic profiles (SDS-PAGE) of phage EcRAM-01. M: Precision Plus protein ladder., S: phage sample. Ladder molecular weights are indicated on the left.

Formatted: English (United States)

Formatted: Font: 10 pt, Bold, Font color: Black

Formatted: Font: 10 pt, Font color: Black

Formatted: Font: 10 pt, Not Bold, Font color: Black

Formatted: Font: 10 pt, Font color: Black

Formatted: Font: 10 pt, Not Bold, Font color: Black

Formatted: Font: 10 pt, Font color: Black

Formatted: Font: 10 pt, Bold, Font color: Black

Formatted: Font: +Body (Calibri), 10 pt, Bold, Font color: Black, Pattern: Clear

Formatted: Font: 10 pt, Font color: Black
