# Supplementary material for: Characterization of Enterobacter phage vB_EcRAM-01, a new Pseudotevenvirus against Enterobacter cloacae, isolated in an urban river in Panama
Source: PLoS One. 2024 Dec 31;19(12):e0310824. doi: 10.1371/journal.pone.0310824 (PMC11687723; doi:10.1371/journal.pone.0310824)
Supplement: Attachment — Submitted filename: PONE-D-23-39735_R2_reviewer1.pdf [file pone.0310824.s008.pdf]

PLOS ONE

Characterization of Enterobacter phage vB_EcRAM-01, a new Pseudotevenvirus against Enterobacter cloacae , isolated in an urban river in Panama.

--Manuscript Draft--

**Manuscript Number:**	PONE-D-23-39735R2
**Article Type:**	Research Article
**Full Title:**	Characterization of Enterobacter phage vB_EcRAM-01, a new Pseudotevenvirus against Enterobacter cloacae , isolated in an urban river in Panama.
**Short Title:**	Characterization a new Pseudotevenvirus against Enterobacter cloacae
**Corresponding Author:**	Álex Omar Matínez-Torres, Doctor<br>Universidad de Panamá: Universidad de Panama<br>Panama, Panamá PANAMA
**Keywords:**	Bacteriophage, E. cloacae complex, phage therapy, molecular characterization, whole genome sequencing, "de novo" assembly
**Abstract:**	The Enterobacter cloacae complex, a prominent bacterium responsible worldwide for most bloodstream infections in the hospital environment, has shown broad-spectrum antibiotic resistance, including carbapenems. Therefore, bacteriophages have again attracted the attention of the science and medical community as an alternative to control MDR bacteria. In this study, water samples from Río Abajo River, in Panama City, Panama, were collected, for phage isolation, purification, characterization and propagation against the E. cloacae complex. As result, a phage produced clear and round plaque-forming units indicating a lytic phage was isolated. Further analyses concluded that this phage is stable at temperatures between 25°C and 50°C, it remains infective in a pH range between 7 to 11, with high sensitivity to UV light. Remarkedly, it exhibits a narrow host specificity only infecting E. cloacae. Whole genome sequencing revealed that is a myovirus with a genome size of 178,477 bp, a G-C content of 45.8%, and containing approximately 294 genes. Among them, protein-encoding genes involved in morphology, inactivation, adsorption to cells, DNA injection and lytic enzymes were identified. Additionally, the genome contained two tRNA sequences. Genes that encode holins and endolysins, typical of lytic bacteriophages, were also present. A whole-genome sequencing analysis indicated that, according to the genus demarcation criteria, this phage belongs to a novel species within the Family Straboviridae, called genusPseudotevenvirus.
**Order of Authors:**	Jordi Querol-Audi, Ph.D.
	Álex Omar Matínez-Torres, Doctor
	Ednner Victoria-Blanco, Magister
	Jean Pierre González-Gómez
	Juan Raul Medina-Sánchez
	Alexander Martínez
	Nohelia Castro del Campo
	Cristóbal Cháidez-Quirós
**Response to Reviewers:**	Response to the reviewers:<br>We appreciate your valuable feedback on our manuscript entitled Characterization of Enterobacter phage vB_EcRAM-01, a new Pseudotevenvirus against Enterobacter cloacae, isolated in an urban river in Panama. We are grateful for the opportunity to

Powered by Editorial Manager® and ProduXion Manager® from Aries Systems Corporation

	address your comments.<br><br>Reviewer #1: 1. In introduction section; the authors do not go into E. cloacae complex-infecting phages quite enough for this paper. Write more reports about phages specific for E. cloacae complex.<br>We thank the reviewer for pointing out this missing piece of information. A full paragraph has been included mentioning recent studies about phages specific for E. cloacae complex in the current version (lines 56-65)<br><br>2. Have you done bacterial reduction assay at different MOI?<br>We acknowledge the importance of investigating the impact of varying MOIs on phage-mediated bacterial reduction. Here we present an exploratory experiment and we have already begun planning subsequent experiments to analyze the dose-response relationship between MOI and bacterial clearance, particularly in combination with different antibiotics that we intend to address in a future paper.<br><br>3. In Methods: state the procedure of phage concentration.<br>The reviewer is right. A section describing the phage concentration procedure has been included in the Methods section in this new version (lines 94-101)<br>.<br>4. In Methods: Please add SDS-PAGE analysis protocol.<br>A section describing the SDS-PAGE analysis protocol has been included in the Methods section in this new version (lines 119-124).<br><br>5. In result section: you did not write about phage isolation.<br>Thank you for pointing this out. A paragraph describing phage isolation has been included in the results section (lines 184-188).<br>.<br>6. S2 Fig and S3 Fig are not clear. Please provide clear images with correct resolution.<br>We acknowledge that the resolution of the TEM image may not meet modern standards due to the limitations of the equipment used. However, we would like to emphasize that nowadays, the classification and identification of bacteriophages are increasingly reliant on genomic analysis rather than on morphological characteristics. Recent studies have demonstrated the importance of complete genome sequencing in the accurate classification of bacteriophages within specific genera. This approach has been widely adopted in the field and has led to a more precise and systematic classification of bacteriophages, overcoming the limitations associated with morphological variability and resolution in TEM images.<br>Regarding S3 Fig, we agree with the reviewer that the protein bands are quite faint. We know that the precipitation method used in this study doesn't allow to obtain a more concentrated phage sample. Unfortunately, we do not have access to an ultracentrifuge in Panama that would allow us to concentrate the viruses in order to get a better SDS-PAGE sample.<br><br>Reviewer #2: Please revise the representation of the values of the ordinate in Fig.2. And check carefully through out the manuscript.<br>The values of the ordinates in Figure 2 have been modified.<br>Once again, we appreciate your constructive feedback, which has strengthened the scientific rigor of our work. We are committed to addressing all concerns raised by the reviewers and ensuring the clarity and completeness of our manuscript.<br><br>Thank you for your consideration.
**Additional Information:**	
**Question**	**Response**
**Financial Disclosure**<br><br>Enter a financial disclosure statement that describes the sources of funding for the work included in this submission. Review the submission guidelines for detailed requirements. View published research	Yes

articles from *PLOS ONE* for specific examples.<br><br>This statement is required for submission and **will appear in the published article** if the submission is accepted. Please make sure it is accurate.<br><br>**Funded studies**<br>Enter a statement with the following details:<br>• Initials of the authors who received each award<br>• Grant numbers awarded to each author<br>• The full name of each funder<br>• URL of each funder website<br>• Did the sponsors or funders play any role in the study design, data collection and analysis, decision to publish, or preparation of the manuscript?<br><br>Did you receive funding for this work?	
Please add funding details.<br>as follow-up to "**Financial Disclosure**<br><br>Enter a financial disclosure statement that describes the sources of funding for the work included in this submission. Review the submission guidelines for detailed requirements. View published research articles from *PLOS ONE* for specific examples.<br><br>This statement is required for submission and **will appear in the published article** if the submission is accepted. Please make sure it is accurate.	This work was funded in part by the Sistema Nacional de Investigación (SNI) of the Secretaría Nacional de Ciencia, Tecnología e Innovación (SENACYT, Panama), through grants 87-2019, 81-2019 and FID18-044, and also from the Water, Protected Areas and Wildlife Trust of the Ministry of Environment of Panama through grant 026-45-2019 and grant 111130406 from the Ministry of Economy and Finance of Panama. The funders had no role in study design, data collection and analysis, decision to publish, or preparation of the manuscript.

**Funded studies**<br>Enter a statement with the following details:<br>• Initials of the authors who received each award<br>• Grant numbers awarded to each author<br>• The full name of each funder<br>• URL of each funder website<br>• Did the sponsors or funders play any role in the study design, data collection and analysis, decision to publish, or preparation of the manuscript?<br><br>Did you receive funding for this work?"	
Please select the country of your main research funder (please select carefully as in some cases this is used in fee calculation).<br>as follow-up to "**Financial Disclosure**<br><br>Enter a financial disclosure statement that describes the sources of funding for the work included in this submission. Review the submission guidelines for detailed requirements. View published research articles from *PLOS ONE* for specific examples.<br><br>This statement is required for submission and **will appear in the published article** if the submission is accepted. Please make sure it is accurate.<br><br>**Funded studies**<br>Enter a statement with the following details:<br>• Initials of the authors who received each award<br>• Grant numbers awarded to each author<br>• The full name of each funder<br>• URL of each funder website<br>• Did the sponsors or funders play any role in the study design, data collection and analysis, decision to publish, or preparation of the manuscript?<br><br>Did you receive funding for this work?"	PANAMA - PA
**Competing Interests**	The authors have declared that no competing interests exist.

Use the instructions below to enter a competing interest statement for this submission. On behalf of all authors, disclose any competing interests that could be perceived to bias this work—acknowledging all financial support and any other relevant financial or non-financial competing interests.<br><br>This statement is required for submission and **will appear in the published article** if the submission is accepted. Please make sure it is accurate and that any funding sources listed in your Funding Information later in the submission form are also declared in your Financial Disclosure statement.<br><br>View published research articles from *PLOS ONE* for specific examples.<br><br>**NO authors have competing interests**<br><br>Enter: *The authors have declared that no competing interests exist.*<br><br>**Authors with competing interests**<br><br>Enter competing interest details beginning with this statement:<br><br>*I have read the journal's policy and the authors of this manuscript have the following competing interests: [insert competing interests here]*<br><br>* typeset	
**Ethics Statement**<br><br>Enter an ethics statement for this submission. This statement is required if the study involved:<br><br>• Human participants<br>• Human specimens or tissue<br>• Vertebrate animals or cephalopods<br>• Vertebrate embryos or tissues	The Bioethics Committee of the Universidad de Panamá with the endorsement of the Dirección General de Salud Pública of the Ministerio de Salud (Ministry of Health), approved this study (Sequential: 617). The samples were analyzed anonymously.

• Field research<br><br>Write "N/A" if the submission does not require an ethics statement.<br><br>General guidance is provided below. Consult the submission guidelines for detailed instructions. **Make sure that all information entered here is included in the Methods section of the manuscript.**	

Powered by Editorial Manager® and ProduXion Manager® from Aries Systems Corporation

**Format for specific study types**<br><br>**Human Subject Research (involving human participants and/or tissue)**<br>• Give the name of the institutional review board or ethics committee that approved the study<br>• Include the approval number and/or a statement indicating approval of this research<br>• Indicate the form of consent obtained (written/oral) or the reason that consent was not obtained (e.g. the data were analyzed anonymously)<br><br>**Animal Research (involving vertebrate animals, embryos or tissues)**<br>• Provide the name of the Institutional Animal Care and Use Committee (IACUC) or other relevant ethics board that reviewed the study protocol, and indicate whether they approved this research or granted a formal waiver of ethical approval<br>• Include an approval number if one was obtained<br>• If the study involved *non-human primates*, add *additional details* about animal welfare and steps taken to ameliorate suffering<br>• If anesthesia, euthanasia, or any kind of animal sacrifice is part of the study, include briefly which substances and/or methods were applied<br><br>**Field Research**<br><br>Include the following details if this study involves the collection of plant, animal, or other materials from a natural setting:<br>• Field permit number<br>• Name of the institution or relevant body that granted permission	
**Data Availability**<br><br>Authors are required to make all data underlying the findings described fully available, without restriction, and from the time of publication. PLOS allows rare exceptions to address legal and ethical concerns. See the PLOS Data Policy and FAQ for detailed information.	Yes - all data are fully available without restriction

A Data Availability Statement describing where the data can be found is required at submission. Your answers to this question constitute the Data Availability Statement and **will be published in the article**, if accepted.<br><br>**Important:** Stating 'data available on request from the author' is not sufficient. If your data are only available upon request, select 'No' for the first question and explain your exceptional situation in the text box.<br><br>Do the authors confirm that all data underlying the findings described in their manuscript are fully available without restriction?	
**Describe where the data may be found in full sentences. If you are copying our sample text, replace any instances of XXX with the appropriate details.**<br><br>• If the data are **held or will be held in a public repository**, include URLs, accession numbers or DOIs. If this information will only be available after acceptance, indicate this by ticking the box below. For example: *All XXX files are available from the XXX database (accession number(s) XXX, XXX.*).<br>• If the data are all contained **within the manuscript and/or Supporting Information files**, enter the following: *All relevant data are within the manuscript and its Supporting Information files.*<br>• If neither of these applies but you are able to provide **details of access elsewhere**, with or without limitations, please do so. For example:<br><br>*Data cannot be shared publicly because of [XXX]. Data are available from the XXX Institutional Data Access / Ethics Committee (contact via XXX) for researchers who meet the criteria for access to confidential data.*<br><br>*The data underlying the results presented in the study are available from (include the name of the third party*	The datasets presented in this study can be found in online repositories. The names of the repository/repositories and accession number(s) can be found at https://www.ncbi.nlm.nih.gov/genbank/ , OL551674, SAMN23426491, SRR18344380.

and contact information or URL).<br>• This text is appropriate if the data are owned by a third party and authors do not have permission to share the data.<br><br>* typeset	
Additional data availability information:	Tick here if the URLs/accession numbers/DOIs will be available only after acceptance of the manuscript for publication so that we can ensure their inclusion before publication.

Powered by Editorial Manager® and ProduXion Manager® from Aries Systems Corporation

Manuscript

Click here to access/download;Manuscript;Victoria-Blanco et al Revised Manuscript 220525.docx

Characterization of Enterobacter phage vB_EcRAM-01, a new *Pseudotevenvirus* against *Enterobacter cloacae*, isolated in an urban river in Panama.

Ednner E. Victoria-Blanco[1,2,7,¶], Jean Pierre González-Gómez[4¶], Juan Raúl Medina-Sánchez[2], Alexander A. Martínez[1,6,7], Nohelia Castro del Campo[4], Cristóbal Chaidez-Quiroz[4], Jordi Querol-Audi[1,2,5,7,*], Alex Omar Martínez-Torres[2,3,7,*]

[1] Programa de Maestría en Ciencias Biomédicas, Facultad de Medicina, Universidad de Panamá, Panamá.

[2] Laboratorio de Microbiología Experimental y Aplicada y Microbiología de Aguas (LAMEXA-LAMA), Universidad de Panamá, Panamá.

[3] Departamento de Microbiología y Parasitología, Facultad de Ciencias Naturales, Exactas y Tecnología, Universidad de Panamá, Panamá.

[4] Laboratorio Nacional para la Investigación en Inocuidad Alimentaria (LANIIA), Centro de Investigación en Alimentación y Desarrollo, A.C. (CIAD), Culiacán, Sinaloa, México.

[5] Departamento de Bioquímica y Nutrición, Facultad de Medicina, Universidad de Panamá, Panamá.

[6] Genomics and Proteomics Research Department, Gorgas Memorial Institute of Health Studies, Panamá, Panamá.

[7] Sistema Nacional de Investigación (SNI). Secretaría Nacional de Ciencia, Tecnología e Innovación (SENACYT), Panamá.

[*]Corresponding authors:

E-mail: alex.martinez@up.ac.pa (AOMT) and jordi.querol@up.ac.pa (JQA)

[¶]These authors contributed equally to this work and share first authorship.

Abstract

The *Enterobacter cloacae* complex, a prominent bacterium responsible worldwide for most bloodstream infections in the hospital environment, has shown broad-spectrum antibiotic resistance, including carbapenems. Therefore, bacteriophages have again attracted the attention of the science and medical community as an alternative to control Multidrug resistant bacteria. In this study, water samples from Río Abajo River, in Panama City, Panama, were collected, for phage isolation, purification, characterization and propagation against the *E. cloacae* complex. As result, a phage produced clear and round plaque-forming units indicating a lytic phage was isolated. Further analyses concluded that this phage is stable at temperatures between 25°C and 50°C, it remains infective in a pH range between 7 to 11, with high sensitivity to Ultraviolet light. Remarkedly, it exhibits a narrow host specificity only infecting *E. cloacae*. Whole genome sequencing revealed that is a myovirus with a genome size of 178,477 bp, a G-C content of 45.8%, and containing approximately 294 genes. Among them, protein-encoding genes involved in morphology, inactivation, adsorption to cells, DNA injection and lytic enzymes were identified. Additionally, the genome contained two tRNA sequences. Genes that encode holins and endolysins, typical of lytic bacteriophages, were also present. A whole-genome sequencing analysis indicated that, according to the genus demarcation criteria, this phage belongs to a novel species within the Family *Straboviridae*, called genus *Pseudotevenvirus*.

Introduction

Enterobacteriaceae are ubiquitous in nosocomial settings and can play important roles in the burden of disease in the urinary tract, lower respiratory tract, intra-abdominal organs, and skin infections. The most relevant members of this Gram-negative bacterial group are *Klebsiella pneumoniae*, *Escherichia coli*, and *Enterobacter* spp. The severity of the infections is intensified by the resistance level to most available antibiotics, including carbapenem and cephalosporins [1]. Special attention to *Enterobacter* is due to the intrinsic resistance to ampicillin and broad-spectrum cephalosporin which reduces the alternatives for antibiotic treatments. *Enterobacter* belongs to the ESKAPE group (acronym for *Enterococcus faecium*, *Staphylococcus aureus*, *Klebsiella pneumoniae*, *Acinetobacter baumannii*, *Pseudomonas aeruginosa*, and *Enterobacter* spp), related worldwide to severe nosocomial infections with a highly increasing multi-drug resistance. A problem of epidemiological importance is the presence of AmpC-producing strains together with BLEE-encoding plasmids and colistin co-resistance in *E. cloacae* complex (ECC) strains **[2–4]**. The continuous emergence of multi-drug resistant strains have hampered the effectiveness of antibiotic treatments. Therefore, one possible approach to address this problem is the use of bacteriophages. In itself, the

magnitude of the impact of phage predation on pathogenic bacteria has been demonstrated. In recent years, phage therapy has been successfully applied to *E. coli* O157:H7, causative agent of hemorrhagic colitis and hemolytic uremic syndrome **[5–7]**. The use of phages has also been studied in the treatment of peritoneal infections, otitis externa, in the eradication of *P. aeruginosa* in patients with cystic fibrosis [8,9], in the treatment of urinary infections or osteomyelitis caused by *S. aureus* [10]. The favorable effects of phage therapy may even exceed its antibacterial activity. A surprising number of data has demonstrated the *in vivo* activity of phages such as the adenovirus growth suppression mediated by bacteriophage T4 [11] or development inhibition of *P. aeruginosa* by phage Pf4 [12].

In recent years, several studies have been published on the use of bacteriophages against *E. cloacae*. Isolation and Characterization of Lytic Phages: A recent study focused on the isolation of the phage ECLFM1 from sewage water using a carbapenem-resistant clinical strain as the host. ECLFM1 exhibited rapid adsorption and a 15-minute latent period, with a burst size of approximately 75 PFU/infected cell. This phage belongs to the Karamvirus family and demonstrated high effectiveness in therapeutic applications, increasing survival rates in zebrafish infected with E. cloacae [13]. Another study identified and characterized the phage vB_EclM_Q7622, isolated from sewage in a poultry farm. This phage showed remarkable capability to infect and destroy multi-drug-resistant strains of *E. cloacae*, highlighting its potential as a therapeutic agent in clinical infections [14]. Phages PϕEn-CL and PϕEn-HO demonstrated the ability to eliminate multi-drug-resistant *E. cloacae* strains. The study emphasized the specificity of these phages and their efficacy in destroying bacterial biofilms, which is crucial for treating persistent infections in hospital settings [15].

The principal advantage of employing phages in therapy and biological control lies in their specificity towards bacterial targets. Those that do not infect commensal bacteria can be selected, therefore posing no threat to humans and animals. Their widespread presence in nature, their versatility, and their inherent resilience have elevated their status from mere research organisms to potentially viable and indispensable tools in the battle against growing antimicrobial resistance. However, the transfer of resistance and virulence genes, and other genes associated with negative outcomes through bacteriophages, constituted to an entire research domain aimed at mitigating any potential adverse impacts [16,17]. For these reasons, this study is aimed to isolate and biologically and genetically characterize a phage against the *E. cloacae* complex.

Methods

Bacterial Strains and Growth Conditions.

The host strain ATCC 23355 of *E. cloacae* complex used for the isolation and proliferation of the phage was the same as previously reported [18]. In addition, *S. aureus, E. coli, E. aerogenes, E. cloacae, and P. aeruginosa*, provided by the Human Microbiology Department, Faculty of Medicine, Universidad de Panamá; Hospital Santo Tomas, and Complejo Hospitalario Dr. Arnulfo Arias Madrid, were used for host range determination. The *E. cloacae* host strain, along with twenty-seven clinical isolates of *E. cloacae*, were tested for antibiotic susceptibility using the Vitek II Automated Microbiological System (BioMérieux, Marcy l'Étoile, France) (results shown in Supplementary Table 1). The breakpoints were applied according to the Clinical and Laboratory Standards Institute (CLSI) guidelines [19].

Ethics Statement.

The Bioethics Committee of the Universidad de Panamá with the endorsement of the Dirección General de Salud Pública of the Ministerio de Salud (Ministry of Health), approved this study (Sequential: 617).

Phage isolation.

The Enterobacter phage vB_EcRAM-01 was isolated from waters of Rio Abajo river in Panama City, Panamá (coordinates, 9°00′55.2″N 79°29′25.6″W), using *E. cloacae* strain ATCC 23355 as a propagation host, which was grown previously in tryptic soy broth at 37°C for 24 h. Phage isolation, propagation, and purification were performed using the soft-agar overlay method [20]. Briefly, phage purification process involved serial dilution of filtrates exhibiting lytic activity against *E. cloacae*. Specifically, 100 µL of the phage dilution was mixed with an overnight culture of the host bacterium in 3 mL of soft agar (TSB with 0.4% agar), poured onto TSA plates, and incubated at 37 °C for 24 h. The phage plates, selected based on size and clarity, were transferred to microtubes containing 1 mL of nanopure water and the whole process was repeated at least three times to confirm that the isolated phages were descendants of a single virion.

Phage concentration.

The soft-agar overlay method was used to propagate the isolated phage. 5 mL of phage buffer (40 mM Tris-Cl, 150 mM NaCl, 10 mM $MgSO_4$) were added to each Petri dish and left for 24 hours at 4°C. Then the buffer was recovered with a serological pipette and subsequently, the top agar was scraped. Sample was centrifuged at 4,000 rpm for 15 minutes and the supernatant was recovered and centrifugated again was. The supernatant was recovered and filtered using a 0.22 µm filter. The phage sample was then incubated with PEG8000 (8%w/v final concentration) in an automatic tube mixer O/N at 4°C. Sample was

centrifuged at 6,000 rpm for 15-30 minutes, the supernatant was discarded, and the pellet was gently resuspended with 1 mL of sterile PBS buffer.

Host range of vB_EcRAM-01 against clinical *E. cloacae* isolates: Spot test and efficiency of plating (EOP).

Clinical isolates of *E. cloacae, S. aureus, E. coli, P. aeruginosa*, and *E. aerogenes*, along with *E. cloacae* and *E. aerogenes* ATCC reference strains, were cultured until reaching the exponential growth phase (OD_{600nm} 0.6). Subsequently, 200 - 500 µL of each culture were mixed with 3 mL of top agar containing 10 mM $CaCl_2$ and plated onto a 1.5% nutrient agar plate. After solidification, a single droplet (10 µL) from each phage dilution (10^{-1} to 10^{-8}) was applied onto the agar [21].The droplets were allowed to dry for 10 min and plates were incubated at 37°C for 24 h - 48 h. A phage titer in PFU/mL was calculated for each strain tested [22]. Phage virulence was assessed using the efficiency of the plating (EOP) method. The EOP was determined by dividing the phage titer at the final dilution on the test strain by the phage titer on its original isolation strain. Based on these calculations, phages were categorized into highly virulent (0.1 < EOP > 1.00), avirulent but still active (EOP < 0.001), or completely avirulent (no plaques were observed).

Morphological characterization.

The isolated phage underwent examination using transmission electron microscopy (TEM). A formvar-coated grid (400 mesh copper grid) was utilized, onto which a drop of 10^9 PFU/mL phage stock was applied. Subsequently, negative staining was performed with 2 % phosphotungstic acid (pH 7.2) for 5 minutes, and excess stain was eliminated with filter paper. Electron micrographs were captured at varying magnifications using a JEOL JEM-1011 transmission electron microscope (20).

Sodium dodecyl sulphate polyacrylamide gel electrophoresis (SDS-PAGE).

SDS-PAGE electrophoresis was performed following the Laemmli method [23]. Separation was carried out in 10% resolving gel in standard Tris–glycine buffer at a constant current of 20 mA. A molecular weight standard (Precision Plus protein ladder, BioRad) was used in the electrophoretic separation, after which the gels were stained with Coomassie blue

(Sigma, Ge). After incubation with a solution containing 20% ethanol and 7% acetic acid to remove the excess of stain, images were recorded using a BioDoc-it Imaging System (UVP).

Effects of UV light, temperature and pH on the viability of phage vB_EcRAM-01.

Purified vB_EcRAM-01 was exposed to different physicochemical conditions (UV light, temperature and pH) Suspensions were exposed to ultraviolet light at a distance of 50 cm for 10, 20, 40, 60, 70, and 90 s. To determine the effect of temperature, phage suspensions were placed at 25 ºC, 37 °C, 50 ºC and 70 ºC, respectively. pH stability was determined by mixing assay vB_EcRAM-01 phage samples resuspended in SM-gelatine buffer (50 mM Tris–HCl pH 7.5, 100 mM NaCl, 8 mM MgSO4 and gelatin 2% (w/v)) with a pH ranging from 2 to 14. Phage viability was determined by counting the number of lysis PFU using the double-layer agar technique and the experiments were carried out in triplicates [24,25].

Bacterial reduction assay.

Bacteriolytic activity was evaluated using a spectrophotometer, targeting *E. cloacae* ATCC 23355. The bacterial strain's overnight culture, adjusted to 1.0 OD_{600nm}, served as the inoculum. A concentration of 8 log PFU/mL was tested with appropriate phage dilutions. Each tube, including negative and positive controls, contained 20 mL of TSB. Experimental tubes received an additional 200 µL of the phage dilution, followed by the addition of 1000 µL of bacterial culture to each tube (excluding the negative control). Incubation was carried out for 180 minutes at 37 °C, with measurements at 600 nm taken every 15 minutes. The experiment was conducted in triplicates.

Bacteriophage DNA isolation and genome sequencing.

The QIAamp MinElute Virus Spin kit (QIAGEN, Valencia, CA) was used to extract DNA, according to the manufacturer´s instructions. The purity was assessed spectrophotometrically at 260 and 280 nm, using a BioSpec-nano Micro-volume UV-Vis Spectrophotometer (Shimadzu, Kyoto, Japan).

The DNA libraries were prepared using the Nextera XT DNA Library Preparation Kit (Illumina, San Diego, CA, USA) through fragmentation, PCR amplification, PCR cleanup, and library normalization. The genome was sequenced at the Genomics and Proteomics Research Department of ICGES using the MiSeq Illumina sequencer (2 x 250 bp paired-end reads) (ThermoFisher

Scientific Waltham, Massachusetts USA). Raw reads (629,372 reads in total) were checked for quality using FastQC (www.bioinformatics.Babraham.ac.UK/projects/fastqc) and trimmed using Timmomatic v0.39 [26].

Genome annotations, comparative genomics and phylogenetic analyses.

The genome was assembled through SPAdes v3.15.1 [27], resulting in a single contig with 8.96-fold coverage [27]. We mapped the reads against the contig obtained using Bowtie v0.9.6 (http://bowtie-bio.sourceforge.net/index.shtml) to corroborate the sequence ends [28]. The phage genome annotation was performed using RASTk v2.0 [29–31]. Gene calling was performed through GeneMarkS v4.28 (http://exon.gatech.edu/GeneMark/genemarks.cgi) [32], and Glimmer v3.02 (http://ccb.jhu.edu/software/glimmer/index.shtml) [33,34].

Several methods were used to annotate the ORF functions, including the protein basic local alignment search tool (blastp) on the NCBI server (https://blast.ncbi.nlm.nih.gov/Blast.cgi) based on the search of the non-redundant protein sequence database [35], Hhpred using structural/domain database (https://toolkit.tuebingen.mpg.de/tools/hhpred) [36–39] and HMMER v2.41.1 through HMM database [40,41]. (https://toolkit.tuebingen.mpg.de/tools/hhpred). Using a non-redundant protein matrix, employing Markov hidden model profiles [42], the annotations were manually curated using the Geneious v9.1.8 platform employing default parameters. Identification of transfer RNAs was performed using tRNA scan-SE v2.07 (http://lowelab.ucsc.edu/tRNAscan-SE/) and ARAGORN v1.2.41 [43] software (http://www.ansikte.se/ARAGORN/). Comparative analysis between genomes was performed using Mauve progressive alignment v2.3.1 [44] (http://darlinglab.org/mauve/mauve.html) employing default parameters.

Pairwise comparison of nucleotide sequences was performed using the Genome-BLAST Distance Phylogeny (GBDP) method of the VICTOR (Virus Classification and Tree Building Online Resource) platform [45] (https://ggdc.dsmz.de/victor.php), in the recommended environments for prokaryotic viruses. The resulting intergenomic distances were used to infer a branch-supported balanced minimum evolution tree using FASTME, including SPR post-processing [46] using D6 formula. Branch support was inferred from 100 pseudo-bootstrap replicates each. Trees were rooted at the midpoint [47], visualized using FigTree; personalized with taxon boundaries at species, genus, and family scales; and estimated with the iTOL program [48] (https://itol.embl.de/). The recommended clustering threshold is thirthy nine [45] and an F-value (fraction of links required for cluster fusion) of 0.5 [45].

Determination of taxonomy and viral replication cycle type was achieved using PATRIC v3.6.9 [49] (https://patricbrc.org/) and PhageAI (https://phage.ai/) server [50]. The genome comparison between Enterobacter phage

vB_EcRAM-01 and close relatives was performed using EasyFig v2.2.2 [51]. At the end, the intergenomic similarities amongst complete viral genomes were calculated through the VIRIDIC web tool [52] with blastn default settings.

Statistical analysis.

Statistically significant differences were determined using ANOVA. Differences in group means were compared using Tukey's HSD test. A P-value below 0.05 was considered statistically significant. Statistical analysis was conducted using R version 4.3.1 [53].

Results

Bacteriophage isolation, host range analysis and morphology characterization

In recent years, several studies have been published on the use of bacteriophages against E. *cloacae,* an opportunistic pathogen posing a clinical challenge due to its resistance to multiple antibiotics[13–15]. In this study, the Enterobacter phage vB_EcRAM-01 was isolated from the waters of the Rio Abajo river in Panama City, Panama. Using *E. cloacae* strain ATCC 23355 as the host, the phage was isolated and purified. The isolation involved a series of dilutions and the use of the soft-agar overlay technique to propagate the phage. Clear plaques indicating phage lytic activity were selected and purified to ensure that the isolated phages originated from a single virion.

In order to evaluate the host specificity of vB_EcRAM-01, a comprehensive assessment was conducted involving a total of thirty-three strains: two ATCC strains alongside a diverse selection of, both, Gram-negative and Gram-positive clinical isolates (Fig 1).

Fig 1. Host range specificity and efficiency of plating. The strain code followed by the efficiency value are shown in parentheses. Color range code associated with EOP is indicated on the right.

Notably, vB_EcRAM-01 exhibited the capacity to infect all the *E. cloacae* isolates with varying degrees of efficacy while displaying negligible antimicrobial activity (efficiency < 0.10) against the other species tested, which included *E. coli, E. aerogenes, A. baumannii, S. aureus*, and *P. aeruginosa*. Upon infecting the reference strain, the clear plaque-forming units (PFUs), characterized by smooth perimeters, measured within the range of 3 mm to 7 mm in diameter, a hallmark indicative of its lytic phage attributes (S1 Fig). TEM micrographs of the phage showed a morphology typical of a myovirus, a phage with a

tail, belonging to the class *Caudoviricetes*. The size measurements of its icosahedral head are about 104 x 76 nm, and its contractile tail is approximately 110 nm (S2 Fig). Previously, phage morphology was sufficient to classify them taxonomically, but nowadays bioinformatics tools are used to perform similarity analysis and group them by evolutionary distances.

Environmental stress resistance: stability against UV light, temperature and pH

Exposure to UV light (365 nm) decreased the infectivity of phage vB_ECRAM-01 over time. Results showed an exponential decay resulting in complete inactivation when incubated for more than one minute (Fig 2A).

Fig 2. Stability of vB_ECRAM-01 to (A) UV radiation, (B) temperature, and (C) pH. Data is expressed as mean ± standard error. Treatments with the same letters in panel (C) are not significantly different (Tukey's HSD test, $p>0.05$).

The phage exhibited lytic activity when subjected to temperatures ranging from 25 °C to 50 °C, even after being incubated for 90 minutes (Fig 2B). However, when exposed to 70 °C, the phage became completely inactivated after only 20 min of incubation and, at 80 °C, it lost its infectivity entirely (data not shown for clarity).

The stability of vB_EcRAM-01 was determined at various pH values. It maintained its infectivity in a pH range between pH 7.5 and pH 11. The best conditions were found between pH 9 and 10 (Fig 2C). Incubation under acidic pH resulted in a significant reduction of viral proliferation. The phage was not able to replicate in a pH greater than 11 or less than 3. Overall, the best activity was found in alkaline media.

Bacteriolytic activity

The evaluated phage demonstrated a robust, complete bacteriolytic effect in the initial 105 minutes of the assay. Following this, the OD curve started to rise due to an increase in bacterial cell numbers. This trend persisted in subsequent measurements, although the rate of increase was lower than that observed in the positive control (Fig 3).

Fig 3. Effect of vB_EcRAM-01 on the growth of the E. cloacae complex. The light blue line indicates the bacterial concentration without being infected with the virus at 37°C, showing an absorbance of 1.6. On the other hand, the orange line shows the concentration of the bacteria being infected from the beginning, with a reduced absorbance of up to 0.77 (ANOVA $F = 613.62$. g.l = 1 and $p<0.0001$).

Comparative genomics and phylogenetic analysis

The genomic attributes of phage vB_EcRAM-01 were delineated as follows: the genome spans 178,477 base pairs with a 45.0 % of GC content, hosting a total of 293 genes. Among these, 86 genes were implicated in anticipated functions encompassing morphology, inactivation, cellular adsorption, DNA injection, and host lysis. Through a UniProt database blast search, we were able to identify to structural proteins, one sharing 97% homology with the Major capsid protein (ID: A0A1B1IX95) of Citrobacter phage vB_CfrM_CfP1, having a mass of 56.37 kDa [54] while the other protein exhibits 96.7% homology with the Baseplate wedge subunit protein (ID: M9V1F2) from Escherichia phage Lw1, with a mass of 38.01 kDa [55]. In fact, analysis by polyacrylamide gel electrophoresis revealed two prominent protein bands with molecular masses close to 50 and 37 kDa, respectively (S3 Fig). In bacteriophages possessing both capsid and tail structures, SDS-PAGE typically reveals multiple proteins, including capsid, tail, and baseplate proteins. Capsid proteins form the outer shell encasing the phage genome, while baseplate proteins are situated at the tail base, facilitating recognition and binding to specific receptors on host cell surfaces [56]. Hence, it is plausible that the observed proteins serve these functions; however, precise identification through LC-MS/MS analysis would provide a more definitive comparison [57].

Remarkably, the genome of vB_EcRAM-01 was lacking discernible genes associated with resistance, virulence, or allergenicity. The genomic blueprint encodes two transfer RNAs (tRNAs), one corresponding to methionine (cat) and the other to glycine (tcc). A pertinent insight is the substantial sequence identity—90.11%—shared with other phages (Fig 4) [18].

Fig 4. Comparison of Enterobacter phage vB_EcRAM-01 genome with close relatives. Color scales represent the percentage of nucleotide identity between regions obtained by blastn.

To assign a taxonomic classification to phage vB_EcRAM-01, a phylogenetic tree based on whole-genome was constructed, and intergenomic similarities were calculated between the closely related genomes reported in GenBank. The phylogenetic tree and intergenomic similarity heatmap revealed that phage vB_EcRAM-01 is a new member of the *Pseudotevenvirus* genus within the *Straboviridae* family.

The GBDP phylogenetic tree using the D6 formula with an average support of 31%, identifying ten and nine clusters, respectively, at the genus level. At the family level, there were five and six clusters, respectively (Fig 5).

Fig 5. Phage vB_EcRAM-01 genomic and phylogenetic analyses. Genome Blast Distance Phylogeny (GBDP)-based phylogenetic tree of vB_EcRAM-01 and 28 related Straboviridae members created by iTOL v6.0 platform. The Enterobacter phage vB_EcRAM-01 belongs to the genus Pseudotevenvirus and is a new phage species.

Nucleotide identity analysis (ANI) was done on the 28 genomes with the highest homology in the GenBank database using VIRIDIC. According to the results of the heat mapping, phage vB_EcRAM-01 shares less than 95% nucleotide identity with previously reported genomes, thereby establishing it as a novel species within the Family *Straboviridae*, genus *Pseudotevenvirus*, according to the genus demarcation criteria (>70% nucleotide identity over the entire genome length) recommended by the ICTV (Fig 6).

Fig 6. Taxonomic classification of Enterobacter phage vB_EcRAM-01. A heatmap created by VIRIDIC based on intergenomic similarities between viral genomes, with the proportion of similarity shown by the color scale.

Comparative BLASTn analysis of the complete genome of vB_EcRAM-01 against phages in the GenBank database revealed that vB_EcRAM-01 has a high degree of coverage and identity with the Enterobacter phages ENC22 (OL355128.1), ENC7 (OL355125.1), ENC25 (OL355127.1), EBPL (MT341500.1). In VIRIDIC analysis, the phages shared collinear gene blocks that are identical in length between the two genomes. In comparison, other blocks revealed a high degree of similarity between this phage and others, including Cronobacter phage vB_CsaM IeE (NC_048646.1), Cronobacter phage vB_CsaM IeN (KX431560.1), Citrobacter phage Margaery (KT381880.1), and myoviral members of the subfamily *Tevenvirinae*.

Discussion

In Panama, seven out of the eight bacterial pathogens responsible for 80% of the reported infections concerning the emergence of resistance in a hospital level (ESKAPE) has been identified: *S. aureus* (16%), *Enterococcus* spp (14.4%), *E. coli* (12%), coagulase-negative *Staphylococcus* (11%), *K. pneumoniae* and *K. oxytoca* (8%), *P. aeruginosa* (8%), and *Enterobacter* spp. (5%). Several microorganisms developed antibiotic resistance to the more common antibiotics to treat these bacterial infections like piperacillin-tazobactam, cephalosporins, nitrofurans, and carbapenems [58,59].

Therefore, in comparison to the usage of antibiotics, phage therapy is a viable option. While scientific publications on phage isolation have increased in recent years, only a small number of phages have been commercialized [60]. One of the

primary reasons is a lack of adequate characterization, which is required to detect and mitigate the hazards associated with phage therapy [61,60]. Moreover, the biological characteristics of a phage determines its stability and therefore, its optimum administration forms [61].

As a first step, vB_EcRAM-01was subjected to environmental stress tests, such as temperature, UV radiation and pH, factors that play a role in phage survival [62,63]. The results indicate that vB_EcRAM-01 is stable at temperatures ranging from 25°C to 50°C, although its replication rate is highest at 37°C. This aligns with findings observed in Myoviridae phages [64,65] exhibiting a comparable stability profile within a broad temperature range. However, it deviates from the EBP phage, known for its resilience across a wider temperature span [66]. Notably, inactivation under UV light transpires rapidly, underscoring the necessity of safeguarding possible clinical applications from sunlight exposure. Interestingly, vB_EcRAM-01 maintains its infectivity across expansive pH and temperature ranges, implying potential robustness within the human body. Enhancements through administration systems designed to shield the phage from gastrointestinal pH could potentially further increase its utility and stability in therapeutic applications [67].

The primary objective of this study was to investigate the host specificity (host range) of phage vB_EcRAM-01 concerning isolates within the *E. cloacae* complex. Accurate identification of the host bacteria's specificity for bacteriophage vB_EcRAM-01 is crucial for a comprehensive characterization of this bacteriophage in order to evaluate a potential therapeutic use. Our observations indicate that this bacteriophage displayed reactivity only with its host strain. These results are in line with findings reported for phages TSE1, TSE2, and TSE3 [68]. *E. coli* displayed some sensitivity but did not exhibit complete lysis. Exceptions to this pattern were observed with myophages *Enterobacter* phage ENC22, *Enterobacter* phage ENC7, and *Enterobacter* phage ENC25, which demonstrated the ability to infect diverse *Enterobacter* species [69].

Apart from meeting the requirements of being innocuous to normal microbiota, therapeutic use involves the employment of fully lytic phages that lack undesired virulence factor genes or antibiotic resistance genes [70]. The genomic assessment of the phage vB_EcRAM-01 confirmed its status as a strictly lytic phage according to the PhageAI platform. The genome contained genes coding for holins and endolysins, offering several advantages. These enzymes collaborate to facilitate bacterial lysis, enabling the release of newly assembled viral particles and the termination of the bacterial host [71]. Additionally, holin proteins play a crucial role in regulating the timing of lysis, ensuring that it occurs at the appropriate stage of the phage life cycle [72]. An important aspect to note is that resistance to endolysins has not been reported to date [73]. This suggests a higher probability of success when employing phages containing endolysins. Besides, it lacked genes associated with lysogeny, such as integrases, transposons, and excitons [74].

The evolutionary context designated it as a T4-type myophage spanning a genome length of 178,477 base pairs. Computational analysis further classified it as a viral entity within the Class *Caudoviricetes*, *Strabovviridae* family, and *Pseudotevenvirus* genus [74]. This type of virus is characterized by the presence of tRNA. Due to the compact nature of phage genomes, which constrains their capacity to utilize host resources fully, these genetic elements may serve as compensation for compositional differences between phages and hosts. They have the potential to supplement the translation machinery with their genetic information, thereby improving fitness and fortifying virulence [75].

By scanning the GenBank database for homologous sequences, a 92% sequence homology with myophage EBPL was observed, a phage that also infects *E. cloacae* [66], and 99.16% sequence homology with myophages ENC7, ENC25, and ENC22 [69].

According to genotypic characteristics [76], vB_EcRAM-01, like an EBP phage, is a myovirus-like phage [66]. The vB_EcRAM-01 virus was termed a bacterial infecting virus isolated from Rio Abajo waters that suppressed *E. cloacae*, a myophage, according to the informal phage nomenclature criteria [77–79].

In this study, the genome-wide syntenic analysis indicated that both phages had comparable rearrangements but differed slightly in nucleotide sequence. Additionally, a phylogenetic study of whole-genome sequences showed that both phages diverged from a common ancestor but experienced many nucleotides substitution events since then [80]. Given that EBPL was previously described as a singleton phage in the myophage phylogenetic tree [81], phylogenetic and nucleotide identity analyses indicated that vB_EcRAM-01 might represent a previously unreported species.

Therefore, vB_EcRAM-01 is physiologically and molecularly stable under a wide temperature and pH range. However, it has a restricted host range. Although it had intense lytic activity against the *E. cloacae* complex, isolation was deemed necessary for more efficient eradication.

In Conclusion, this study identified a lytic bacteriophage. The characterization enhances comprehension of this novel phage, and through its phylogenetic relatives, it may exhibit a myovirus-like morphology, as demonstrated by insights from next-generation sequencing. This sequencing elucidates that vB_EcRAM-01 aligns with the Myoviridae family in accordance with the conventional taxonomic classification [82]. According to the phylogenetic analysis previously described, this virus is a new member of the family *Straboviridae* and the genus *Pseudotevenvirus* according to the new classification [83]. The vB_EcRAM-01 strain is also a viable candidate for many clinical applications, including phage therapy and biologic control or decontamination, due to its host specificity, the genetic link, and lack of lysogenic genes.

Data availability statement

The datasets presented in this study can be found in online repositories. The names of the repository/repositories and accession number(s) can be found at https://www.ncbi.nlm.nih.gov/genbank/ , OL551674, SAMN23426491, SRR18344380.

Author Contributions

EEVB and JPGG drafted the manuscript, while JRMS conducted the statistical analysis of the results. NCC and CCHQ, along with JQA, AAM and AOMT, contributed to data analysis and manuscript revisions. EEVB, JQA, AAM and AOMT secured project funding. All authors reviewed and approved the submitted manuscript.

Acknowledgments

Host bacterial strains were provided by José E. Moreno, MSc., of the Microbiology Division of Laboratorio Central de Referencia en Salud Pública, Instituto Conmemorativo Gorgas de Estudios de Salud (ICGES). We extend our gratitude to the staff of the Microbiology Department at the Clinical Laboratory of Hospital Santo Tomas for providing us with the clinical bacteria strains.

Additionally, we would like to thank Claudia González, MSc, Jessica Gondola, MSc, Ambar Moreno, MSc, and Oris Chavarria, MSc, of the Research Department of Genomics and Proteomics of ICGES for providing us with NGS Illumina sequencing. Furthermore, we would like to express our gratitude to Leandra Gómez, MSc, of the Department of Pharmacology at the Faculty of Medicine, Universidad de Panamá, for her invaluable contribution in reviewing the bioethical considerations within this research. Also, thank you to Bs. Tatiana A. Martínez González for her contribution to the English revision of the present work.

Bibliography

1. WHO. Global priority list of antibiotic-resistant bacteria to guide research, discovery, and development of new antibiotics. 2017.

2. Qian W, Fu Y, Liu M, Zhang J, Wang W, Li J, et al. Mechanisms of Action of Luteolin Against Single- and Dual-Species of Escherichia coli and Enterobacter cloacae and Its Antibiofilm Activities. Appl Biochem Biotechnol. 2021;193: 1397–1414. doi:10.1007/s12010-020-03330-w

3. Davin-Regli A, Lavigne J-P, Pagès J-M. Enterobacter spp.: Update on Taxonomy, Clinical Aspects, and Emerging Antimicrobial Resistance. Clin Microbiol Rev. 2019;32. doi:10.1128/CMR.00002-19

4. Mezzatesta ML, Gona F, Stefani S. *Enterobacter cloacae* complex: clinical impact and emerging antibiotic resistance. Future Microbiology. 2012;7: 887–902. doi:10.2217/fmb.12.61

5. Amarillas L, Rubí-Rangel L, Chaidez C, González-Robles A, Lightbourn-Rojas L, León-Félix J. Isolation and Characterization of phiLLS, a Novel Phage with Potential Biocontrol Agent against Multidrug-Resistant Escherichia coli. Frontiers in Microbiology. 2017;8. Available: https://www.frontiersin.org/articles/10.3389/fmicb.2017.01355

6. López-Cuevas O, Castro-Del Campo N, León-Félix J, Valdez-Torres B, Chaidez C. Evaluation of Bacteriophage Av-08 for Simultaneous Biocontrol of Salmonella Montevideo and Escherichia Coli O157:h7 in Experimentally Contaminated Chicken Skin. Journal of Food Safety. 2012;32: 305–310. doi:10.1111/j.1745-4565.2012.00381.x

7. Sharma M, Patel JR, Conway WS, Ferguson S, Sulakvelidze A. Effectiveness of Bacteriophages in Reducing Escherichia coli O157:H7 on Fresh-Cut Cantaloupes and Lettuce. Journal of Food Protection. 2009;72: 1481–1485. doi:10.4315/0362-028X-72.7.1481

8. Morello E, Saussereau E, Maura D, Huerre M, Touqui L, Debarbieux L. Pulmonary Bacteriophage Therapy on Pseudomonas aeruginosa Cystic Fibrosis Strains: First Steps Towards Treatment and Prevention. Aziz R, editor. PLoS ONE. 2011;6: e16963. doi:10.1371/journal.pone.0016963

9. Debarbieux L, Leduc D, Maura D, Morello E, Criscuolo A, Grossi O, et al. Bacteriophages Can Treat and Prevent *Pseudomonas aeruginosa* Lung Infections. J INFECT DIS. 2010;201: 1096–1104. doi:10.1086/651135

10. Abedon ST. Commentary: Phage Therapy of Staphylococcal Chronic Osteomyelitis in Experimental Animal Model. Front Microbiol. 2016;7. doi:10.3389/fmicb.2016.01251

11. Przybylski M, Dzieciątkowski T, Borysowski J, Międzybrodzki R, Górski A. Inhibitory Effects of Bacteriophage Preparations on Adenoviral Replication. INT. 2019;62: 37–44. doi:10.1159/000500540

12. Casto A, Hurwitz A, Kou K, Mansour G, Policke R, Schmidt A, et al. Bacteriophages: The Answer to Antibiotic Resistance? James Madison Undergraduate Research Journal. 20163: 36–41.

13. Ali SF, Teh S-H, Yang H-H, Tsai Y-C, Chao H-J, Peng S-S, et al. Therapeutic Potential of a Novel Lytic Phage, vB_EclM_ECLFM1, against Carbapenem-Resistant Enterobacter cloacae. Int J Mol Sci. 2024;25: 854. doi:10.3390/ijms25020854

14. Nair A, Vyawahare R, Khairnar K. Characterization of a novel, biofilm dispersing, lytic bacteriophage against drug-resistant Enterobacter cloacae. J Appl Microbiol. 2022;132: 2721–2732. doi:10.1111/jam.15420

15. Rahimzadeh Torabi L, Doudi M, Naghavi NS, Monajemi R. Bacteriophages PϕEn-CL and PϕEn-HO can

eliminate MDR *Enterobacter cloacae* and *Enterobacter hormaechei* isolated from burn wound infections without toxicity for human skin cells. FEMS Microbiology Letters. 2021;368: fnab143. doi:10.1093/femsle/fnab143

16. Hassan AY, Lin JT, Ricker N, Anany H. The Age of Phage: Friend or Foe in the New Dawn of Therapeutic and Biocontrol Applications? Pharmaceuticals. 2021;14: 199. doi:10.3390/ph14030199

17. Häusler T. Viruses vs. Superbugs. London: Palgrave Macmillan UK; 2006. doi:10.1007/978-0-230-55228-9

18. Victoria-Blanco EE, González-Gómez JP, Quiroz E, Martínez AA, González C, Castro del Campo N, et al. Complete Genome Sequence of Enterobacter Phage vB_EcRAM-01, a New Pseudotevenvirus against the Enterobacter cloacae Complex. Microbiology Resource Announcements. 10 May 2022: 1–3. doi:10.1128/mra.00045-22

19. CLSI. CLSI Performance Standards for Antimicrobial Susceptibility Testing, CLSI Guideline M100. 28th ed. Wayne, PA: Clinical and Laboratory Standards Institute; 2018.

20. Adams MH, Anderson ES, Kellenberger E. Bacteriophages. New York: Interscience Publishers; 1959.

21. Khan Mirzaei M, Nilsson AS. Isolation of Phages for Phage Therapy: A Comparison of Spot Tests and Efficiency of Plating Analyses for Determination of Host Range and Efficacy. Schuch R, editor. PLoS ONE. 2015;10: e0118557. doi:10.1371/journal.pone.0118557

22. Stephenson, Frank H. Calculations for Molecular Biology and Biotechnology. second edition. USA: Elsevier/AP, Academic Press is an imprint of Elsevier; 2010. Available: Vetbiotech.um.ac.ir

23. Laemmli UK. Cleavage of structural proteins during the assembly of the head of bacteriophage T4. Nature. 1970;227: 680–685. doi:10.1038/227680a0

24. Flores V. Caracterización de bacteriófagos líticos de Salmonella enterica aislados de muestras de pollos. Universidad Mayor San Marcos. 2017.

25. Zaburlin D, Quiberoni A, Mercanti D. Changes in Environmental Conditions Modify Infection Kinetics of Dairy Phages. Food Environ Virol. 2017;9: 270–276. doi:10.1007/s12560-017-9296-2

26. Bolger AM, Lohse M, Usadel B. Trimmomatic: a flexible trimmer for Illumina sequence data. Bioinformatics. 2014;30: 2114–2120. doi:10.1093/bioinformatics/btu170

27. Bankevich A, Nurk S, Antipov D, Gurevich AA, Dvorkin M, Kulikov AS, et al. SPAdes: A New Genome Assembly Algorithm and Its Applications to Single-Cell Sequencing. Journal of Computational Biology. 2012;19: 455–477. doi:10.1089/cmb.2012.0021

28. Langmead B, Trapnell C, Pop M, Salzberg SL. Ultrafast and memory-efficient alignment of short DNA sequences to the human genome. Genome Biology. 2009;10: R25. doi:10.1186/gb-2009-10-3-r25

29. Aziz RK, Bartels D, Best AA, DeJongh M, Disz T, Edwards RA, et al. The RAST Server: rapid annotations using subsystems technology. BMC Genomics. 2008;9: 75. doi:10.1186/1471-2164-9-75

30. Brettin T, Davis JJ, Disz T, Edwards RA, Gerdes S, Olsen GJ, et al. RASTtk: a modular and extensible implementation of the RAST algorithm for building custom annotation pipelines and annotating batches of genomes. Sci Rep. 2015;5: 8365. doi:10.1038/srep08365

31. Overbeek R, Olson R, Pusch GD, Olsen GJ, Davis JJ, Disz T, et al. The SEED and the Rapid Annotation of microbial genomes using Subsystems Technology (RAST). Nucleic Acids Res. 2014;42: D206-214.

doi:10.1093/nar/gkt1226

32. Besemer J, Lomsadze A, Borodovsky M. GeneMarkS: a self-training method for prediction of gene starts in microbial genomes. Implications for finding sequence motifs in regulatory regions. Nucleic Acids Research. 2001;29: 2607–2618. doi:10.1093/nar/29.12.2607

33. Delcher AL, Bratke KA, Powers EC, Salzberg SL. Identifying bacterial genes and endosymbiont DNA with Glimmer. Bioinformatics. 2007;23: 673–679. doi:10.1093/bioinformatics/btm009

34. Salzberg SL, Delcher AL, Kasif S, White O. Microbial gene identification using interpolated Markov models. Nucleic Acids Res. 1998;26: 544–548. doi:10.1093/nar/26.2.544

35. Altschul SF, Madden TL, Schäffer AA, Zhang J, Zhang Z, Miller W, et al. Gapped BLAST and PSI-BLAST: a new generation of protein database search programs. Nucleic Acids Res. 1997;25: 3389–3402.

36. Gabler F, Nam S-Z, Till S, Mirdita M, Steinegger M, Söding J, et al. Protein Sequence Analysis Using the MPI Bioinformatics Toolkit. Current Protocols in Bioinformatics. 2020;72: e108. doi:https://doi.org/10.1002/cpbi.108

37. Zimmermann L, Stephens A, Nam S-Z, Rau D, Kübler J, Lozajic M, et al. A Completely Reimplemented MPI Bioinformatics Toolkit with a New HHpred Server at its Core. Journal of Molecular Biology. 2018;430: 2237–2243. doi:10.1016/j.jmb.2017.12.007

38. Meier A, Söding J. Automatic Prediction of Protein 3D Structures by Probabilistic Multi-template Homology Modeling. PLOS Computational Biology. 2015;11: e1004343. doi:10.1371/journal.pcbi.1004343

39. Hildebrand A, Remmert M, Biegert A, Söding J. Fast and accurate automatic structure prediction with HHpred. Proteins: Structure, Function, and Bioinformatics. 2009;77: 128–132. doi:https://doi.org/10.1002/prot.22499

40. Potter SC, Luciani A, Eddy SR, Park Y, Lopez R, Finn RD. HMMER web server: 2018 update. Nucleic Acids Research. 2018;46: W200–W204. doi:10.1093/nar/gky448

41. Finn RD, Clements J, Arndt W, Miller BL, Wheeler TJ, Schreiber F, et al. HMMER web server: 2015 update. Nucleic Acids Research. 2015;43: W30–W38. doi:10.1093/nar/gkv397

42. Hofmann P, Tashman Z. Hidden Markov Models and Their Application for Predicting Failure Events. In: Krzhizhanovskaya VV, Závodszky G, Lees MH, Dongarra JJ, Sloot PMA, Brissos S, et al., editors. Computational Science – ICCS 2020. Cham: Springer International Publishing; 2020. pp. 464–477. doi:10.1007/978-3-030-50420-5_35

43. Laslett D, Canback B. ARAGORN, a program to detect tRNA genes and tmRNA genes in nucleotide sequences. Nucleic Acids Res. 2004;32: 11–16. doi:10.1093/nar/gkh152

44. Darling ACE, Mau B, Blattner FR, Perna NT. Mauve: Multiple Alignment of Conserved Genomic Sequence With Rearrangements. Genome Res. 2004;14: 1394–1403. doi:10.1101/gr.2289704

45. Meier-Kolthoff JP, Göker M. VICTOR: genome-based phylogeny and classification of prokaryotic viruses. Bioinformatics. 2017;33: 3396–3404. doi:10.1093/bioinformatics/btx440

46. Lefort V, Desper R, Gascuel O. FastME 2.0: A Comprehensive, Accurate, and Fast Distance-Based Phylogeny Inference Program. Mol Biol Evol. 2015;32: 2798–2800. doi:10.1093/molbev/msv150

47. Farris JS. Estimating Phylogenetic Trees from Distance Matrices. The American Naturalist. 1972;106:

645–668.

48. Letunic I, Bork P. Interactive Tree Of Life (iTOL) v4: recent updates and new developments. Nucleic Acids Research. 2019;47: W256–W259. doi:10.1093/nar/gkz239

49. Davis JJ, Wattam AR, Aziz RK, Brettin T, Butler R, Butler RM, et al. The PATRIC Bioinformatics Resource Center: expanding data and analysis capabilities. Nucleic Acids Res. 2020;48: D606–D612. doi:10.1093/nar/gkz943

50. Tynecki P, Guziński A, Kazimierczak J, Jadczuk M, Dastych J, Onisko A. PhageAI - Bacteriophage Life Cycle Recognition with Machine Learning and Natural Language Processing. 2020 [cited 11 Apr 2021]. doi:10.1101/2020.07.11.198606

51. Sullivan MJ, Petty NK, Beatson SA. Easyfig: a genome comparison visualizer. Bioinformatics. 2011;27: 1009–1010. doi:10.1093/bioinformatics/btr039

52. Moraru C, Varsani A, Kropinski AM. VIRIDIC—A Novel Tool to Calculate the Intergenomic Similarities of Prokaryote-Infecting Viruses. Viruses. 2020;12: 1268. doi:10.3390/v12111268

53. R Core Team. R: A Language and Environment for Statistical Computing. Vienna, Austria: R Foundation for Statistical Computing; 2023.

54. Oliveira H, Pinto G, Oliveira A, Oliveira C, Faustino MA, Briers Y, et al. Characterization and genome sequencing of a Citrobacter freundii phage CfP1 harboring a lysin active against multidrug-resistant isolates. Appl Microbiol Biotechnol. 2016;100: 10543–10553. doi:10.1007/s00253-016-7858-0

55. Kushkina AI, Tovkach FI, Comeau AM, Kostetskii IE, Lisovski I, Ostapchuk AM, et al. Complete Genome Sequence of Escherichia Phage Lw1, a New Member of the RB43 Group of Pseudo T-Even Bacteriophages. Genome Announcements. 2013;1: 10.1128/genomea.00743-13. doi:10.1128/genomea.00743-13

56. Kutter E, Sulakvelidze A, editors. Bacteriophages: biology and applications. Boca Raton, FL: CRC Press; 2005.

57. Al-Wrafy F, Brzozowska E, Górska S, Drab M, Strus M, Gamian A. Identification and characterization of phage protein and its activity against two strains of multidrug-resistant Pseudomonas aeruginosa. Sci Rep. 2019;9: 13487. doi:10.1038/s41598-019-50030-5

58. Moreno J. Antibiotic resistance profiles in Panama: Trends from 2007 to 2013. PPIJ. 2018;6. doi:10.15406/ppij.2018.06.00200

59. MINSA. 18400_2011_31657_INFORME RESISTENCIA ANTIBIOTICOS-USO RACIONAL -036.pdf. Panama: ICGES; 2015 p. 48.

60. Abedon ST. Detection of Bacteriophages: Phage Plaques. In: Harper DR, Abedon ST, Burrowes BH, McConville ML, editors. Bacteriophages. Cham: Springer International Publishing; 2018. pp. 1–32. doi:10.1007/978-3-319-40598-8_16-1

61. Hyman P, Abedon ST. Bacteriophage: Overview. Reference Module in Biomedical Sciences. Elsevier; 2019. p. B978012801238302506X. doi:10.1016/B978-0-12-801238-3.02506-X

62. Phothichaisri W, Ounjai P, Phetruen T, Janvilisri T, Khunrae P, Singhakaew S, et al. Characterization of Bacteriophages Infecting Clinical Isolates of Clostridium difficile. Front Microbiol. 2018;9. doi:10.3389/fmicb.2018.01701

63. Silva YJ, Costa L, Pereira C, Cunha Â, Calado R, Gomes NCM, et al. Influence of environmental variables in the efficiency of phage therapy in aquaculture. Microbial Biotechnology. 2014;7: 401–413. doi:10.1111/1751-7915.12090

64. Litt PK, Jaroni D. Isolation and Physiomorphological Characterization of Escherichia coli O157:H7-Infecting Bacteriophages Recovered from Beef Cattle Operations. In: International Journal of Microbiology [Internet]. 2017 [cited 9 Jul 2019]. doi:10.1155/2017/7013236

65. Yamaki S, Kawai Y, Yamazaki K. Characterization of a novel bacteriophage, Phda1, infecting the histamine-producing Photobacterium damselae subsp. damselae. Journal of Applied Microbiology. 2015;118: 1541–1550. doi:https://doi.org/10.1111/jam.12809

66. Asif M, Naseem H, Alvi IA, Basit A, Rehman S-. Characterization of a lytic EBP bacteriophage with large size genome against Enterobacter cloacae. APMIS. 2021;129: 461–469. doi:10.1111/apm.13138

67. Dąbrowska K. Phage therapy: What factors shape phage pharmacokinetics and bioavailability? Systematic and critical review. Med Res Rev. 2019;39: 2000–2025. doi:10.1002/med.21572

68. Khawaja KA, Abbas Z, Rehman S ur. Isolation and characterization of lytic phages TSE1-3 against Enterobacter cloacae. Open Life Sciences. 2016;11. doi:10.1515/biol-2016-0038

69. Finney AG, Perry JM, Evans DR, Westbrook KJ, McElheny CL, Iovleva A, et al. Isolation and Characterization of Lytic Bacteriophages Targeting Diverse Enterobacter spp. Clinical Isolates. PHAGE. 2022;3: 50–58. doi:10.1089/phage.2021.0011

70. Soohey RL. Identifyng Genes Essential For Lysogeny in Cluster e Mycobacteriophage Ukulele. The University of Maine. 2017.

71. Pollenz RS, Bland J, Pope WH. Bioinformatic characterization of endolysins and holin-like membrane proteins in the lysis cassette of phages that infect Gordonia rubripertincta. PLOS ONE. 2022;17: e0276603. doi:10.1371/journal.pone.0276603

72. Zhang M, Wang Y, Chen J, Hong X, Xu X, Wu Z, et al. Identification and Characterization of a New Type of Holin-Endolysin Lysis Cassette in Acidovorax oryzae Phage AP1. Viruses. 2022;14: 167. doi:10.3390/v14020167

73. Larpin Y, Oechslin F, Moreillon P, Resch G, Entenza JM, Mancini S. In vitro characterization of PlyE146, a novel phage lysin that targets Gram-negative bacteria. PLOS ONE. 2018;13: e0192507. doi:10.1371/journal.pone.0192507

74. Ríos-Sandoval M. Caracterización Genómica, Morfológica y Replicativa del Bacteriófago ΦXaF18 de Xanthomonas vesicatoria. Centro De Investigación Y Asistencia En Tecnología Y Diseño Del Estado De Jalisco, A. C. 2019.

75. Hatfull GF, Dedrick RM, Schooley RT. Phage Therapy for Antibiotic-Resistant Bacterial Infections. Annu Rev Med. 2022;73: 197–211. doi:10.1146/annurev-med-080219-122208

76. Hatfull GF. Bacteriophage genomics. Current Opinion in Microbiology. 2008;11: 447–453. doi:10.1016/j.mib.2008.09.004

77. Turner D, Kropinski AM, Adriaenssens EM. A Roadmap for Genome-Based Phage Taxonomy. Viruses. 2021;13: 506. doi:10.3390/v13030506

78. Adriaenssens EM, Sullivan MB, Knezevic P, van Zyl LJ, Sarkar BL, Dutilh BE, et al. Taxonomy of prokaryotic viruses: 2018-2019update from the ICTV Bacterial and Archaeal Viruses Subcommittee. Arch Virol. 2020;165: 1253–1260. doi:10.1007/s00705-020-04577-8

79. Adriaenssens E, Brister JR. How to Name and Classify Your Phage: An Informal Guide. Viruses. 2017;9: 70. doi:10.3390/v9040070

80. Leskinen K, Blasdel BG, Lavigne R, Skurnik M. RNA-Sequencing Reveals the Progression of Phage-Host Interactions between φR1-37 and Yersinia enterocolitica. Viruses. 2016;8: 111. doi:10.3390/v8040111

81. Jacquemot L, Bettarel Y, Monjol J, Corre E, Halary S, Desnues C, et al. Therapeutic Potential of a New Jumbo Phage That Infects Vibrio coralliilyticus, a Widespread Coral Pathogen. Front Microbiol. 2018;0. doi:10.3389/fmicb.2018.02501

82. Adams MJ, Lefkowitz EJ, King AMQ, Harrach B, Harrison RL, Knowles NJ, et al. 50 years of the International Committee on Taxonomy of Viruses: progress and prospects. Arch Virol. 2017;162: 1441–1446. doi:10.1007/s00705-016-3215-y

83. Turner D, Shkoporov AN, Lood C, Millard AD, Dutilh BE, Alfenas-Zerbini P, et al. Abolishment of morphology-based taxa and change to binomial species names: 2022 taxonomy update of the ICTV bacterial viruses subcommittee. Arch Virol. 2023;168: 74. doi:10.1007/s00705-022-05694-2

Supplementary figure legends

S1 Fig. Lytic effect of bacteriophage vB_EcRAM-01. According to the double agar plate method, the bacterial lawn of the *E. cloacae* complex is infected by bacteriophages. After purification, lysis PFU measuring approximately 9 mm (shown in the figure), rough-edged and completely clear, were obtained.

S2 Fig. Overall morphology of vB_EcRAM-01. A representative micrograph of a purified sample of the bacteriophage is shown (scale bar: 100nm).

S3 Fig. Electrophoretic profiles (SDS-PAGE) of phage EcRAM-01. M: Precision Plus protein ladder., S: phage sample. Ladder molecular weights are indicated on the left.

Figure 1

Click here to access/download;Figure;Fig 1.tif

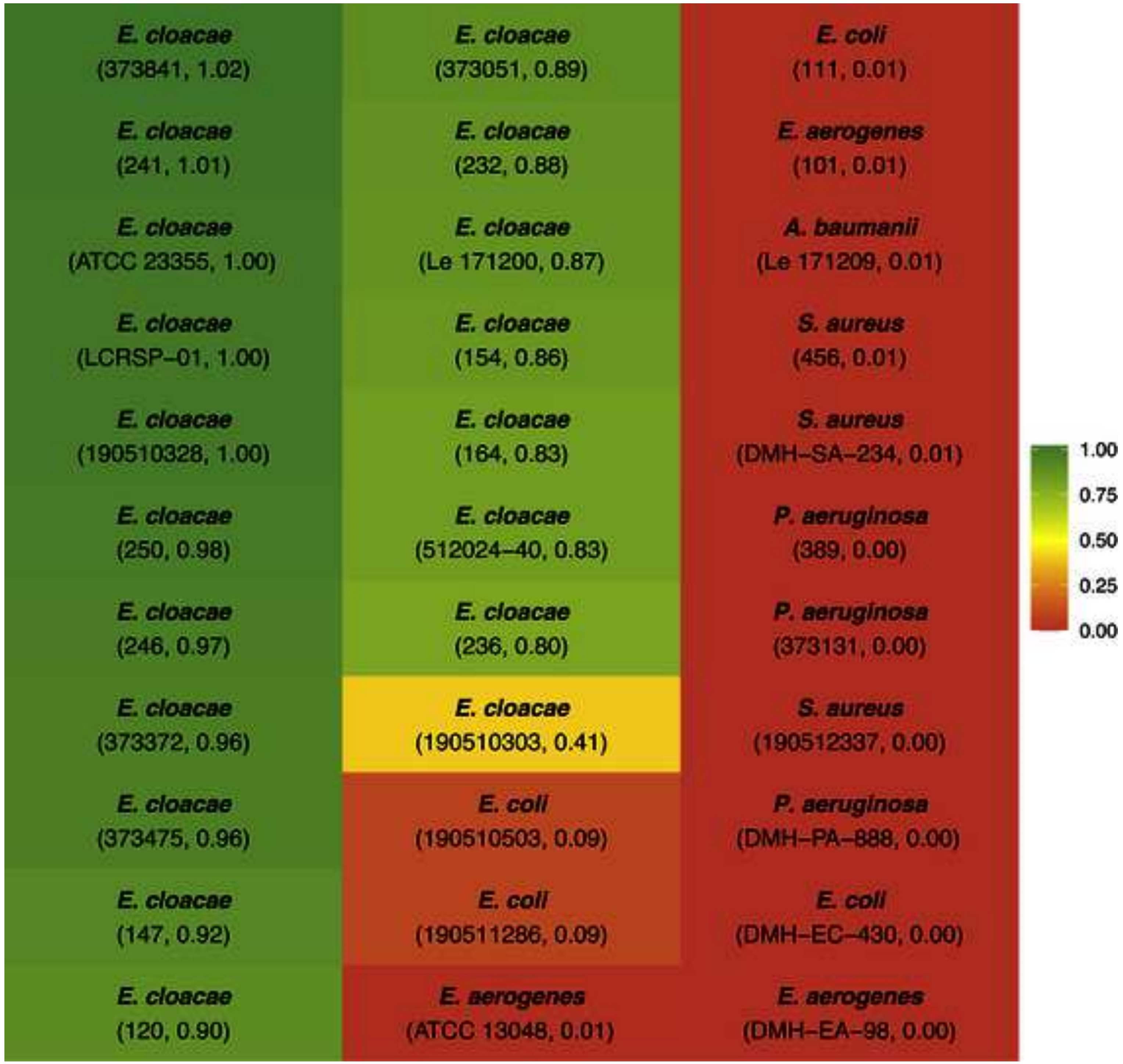

Figure 3

Click here to access/download;Figure;Fig 3.tif

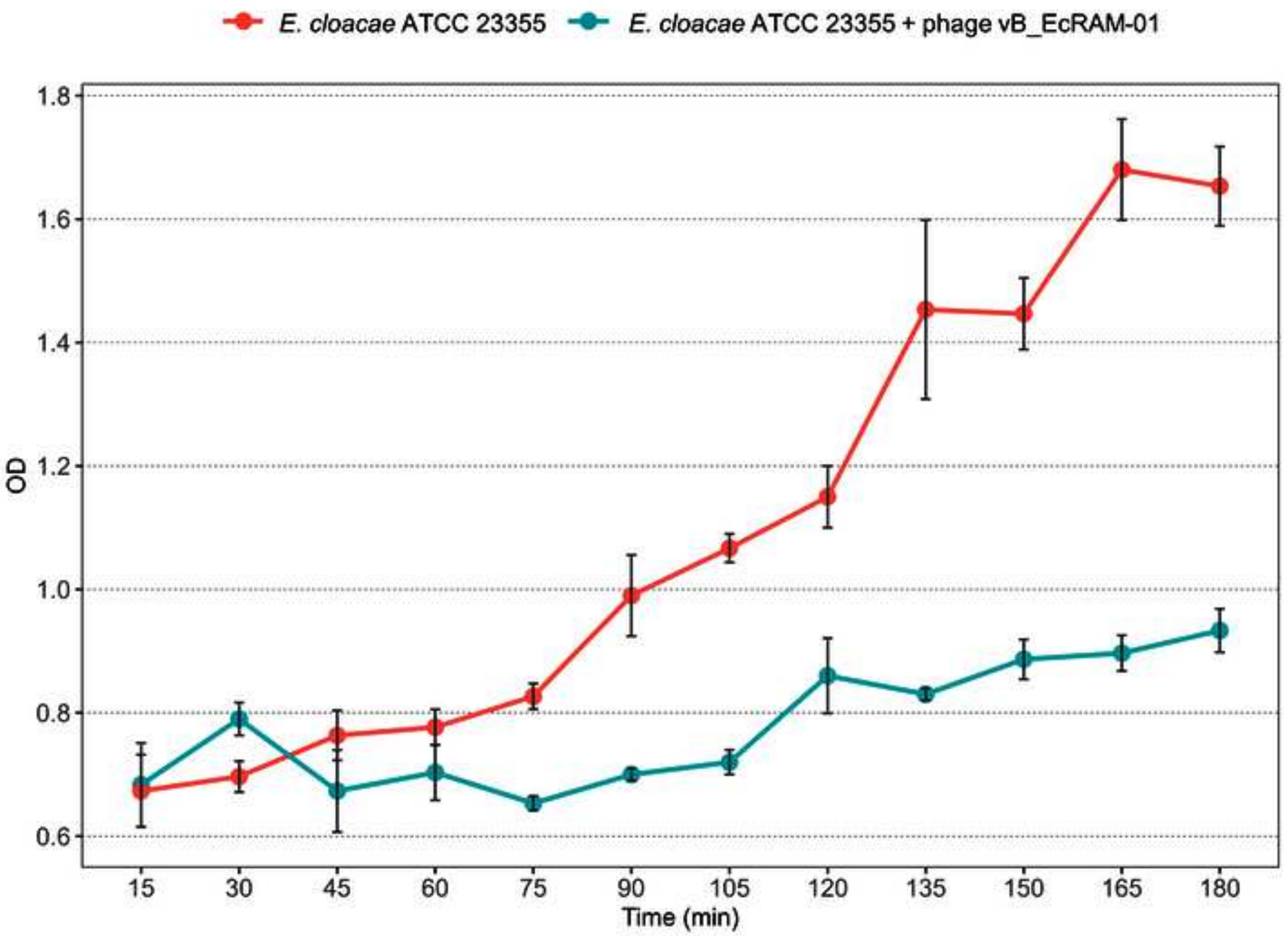

Figure 4

Click here to access/download;Figure;Figure4.eps

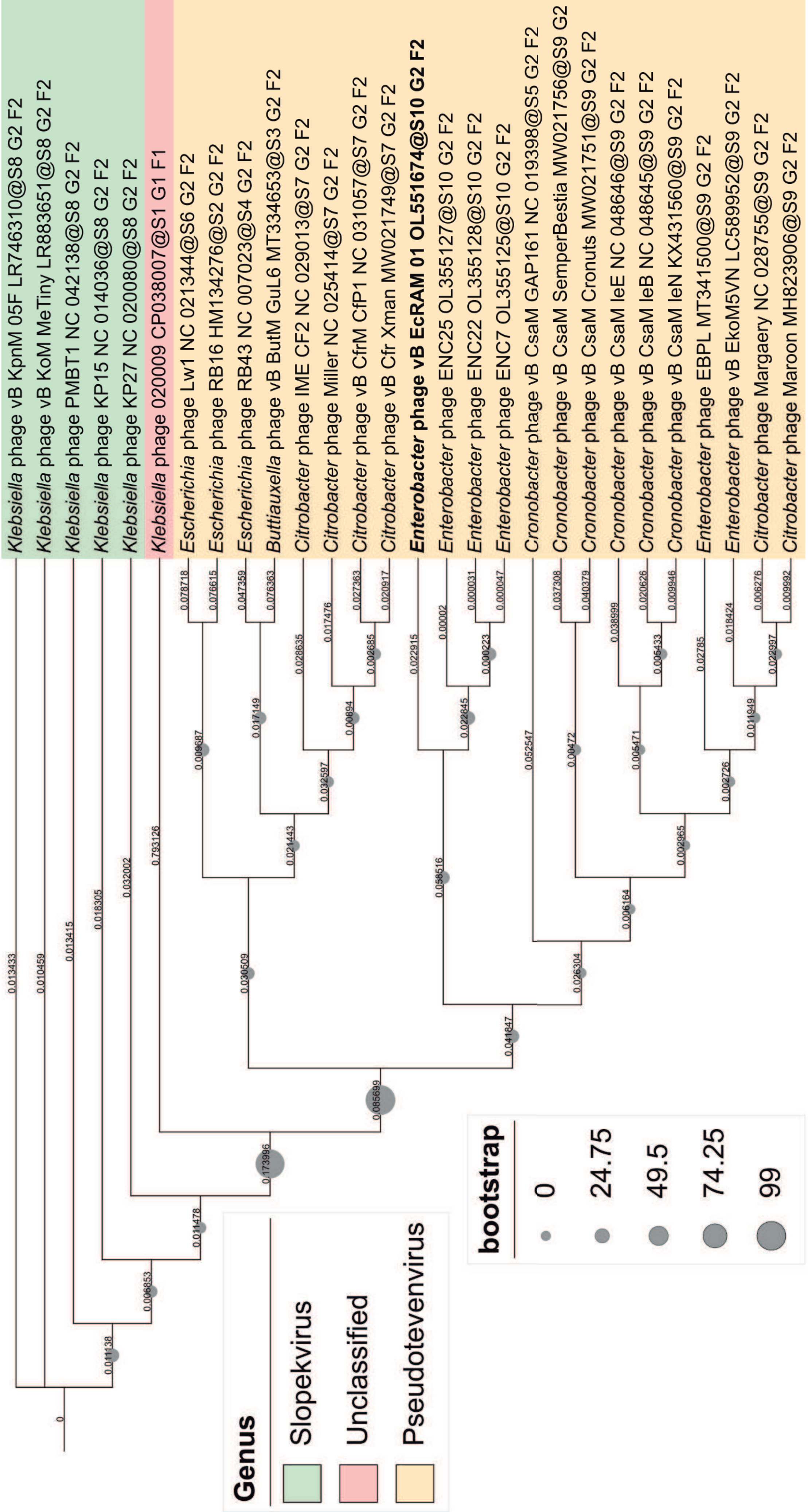

Figure 5

Click here to access/download;Figure;Fig 5.tif

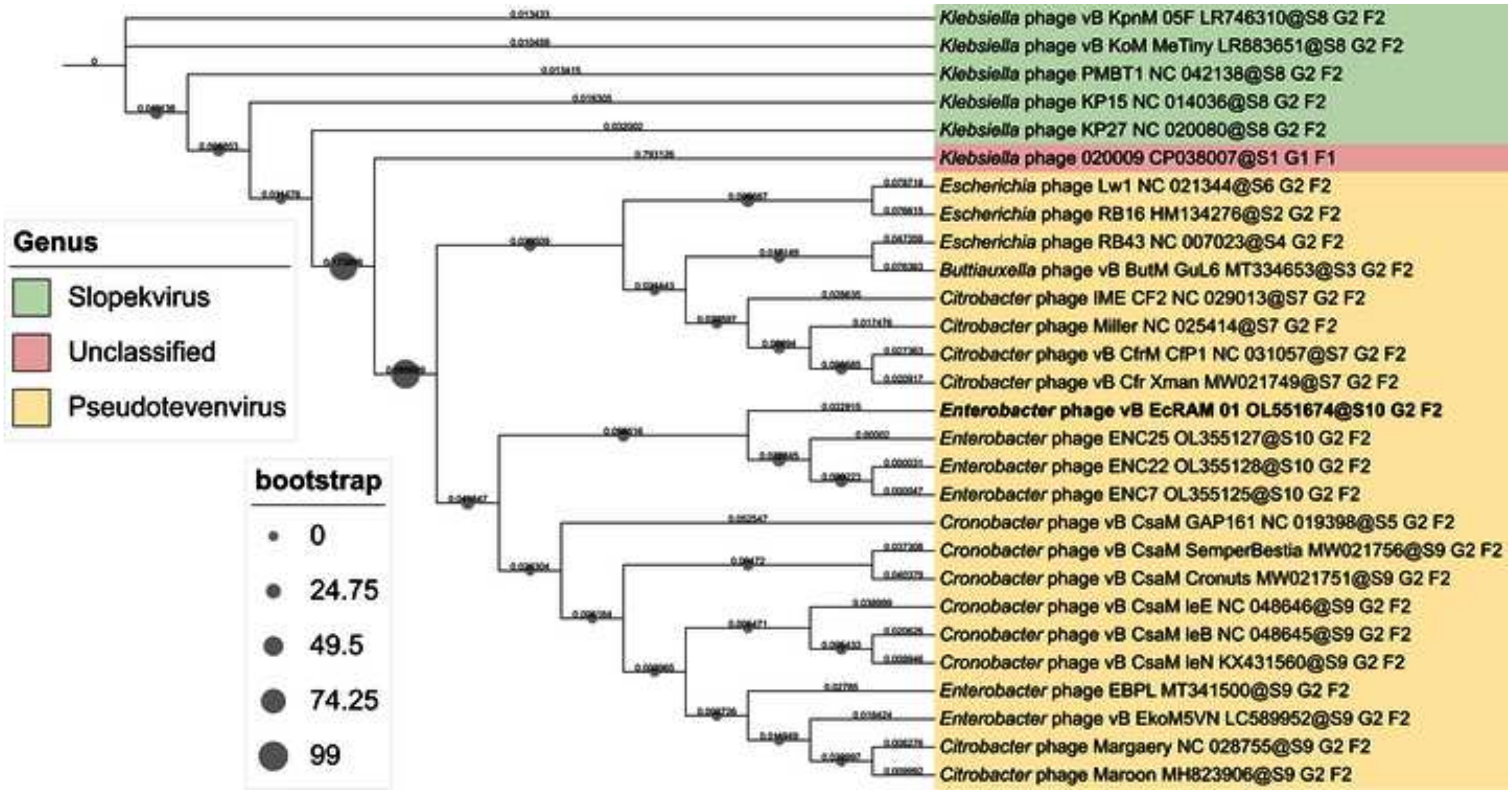

Figure 6

Click here to access/download;Figure;Fig 6.tif

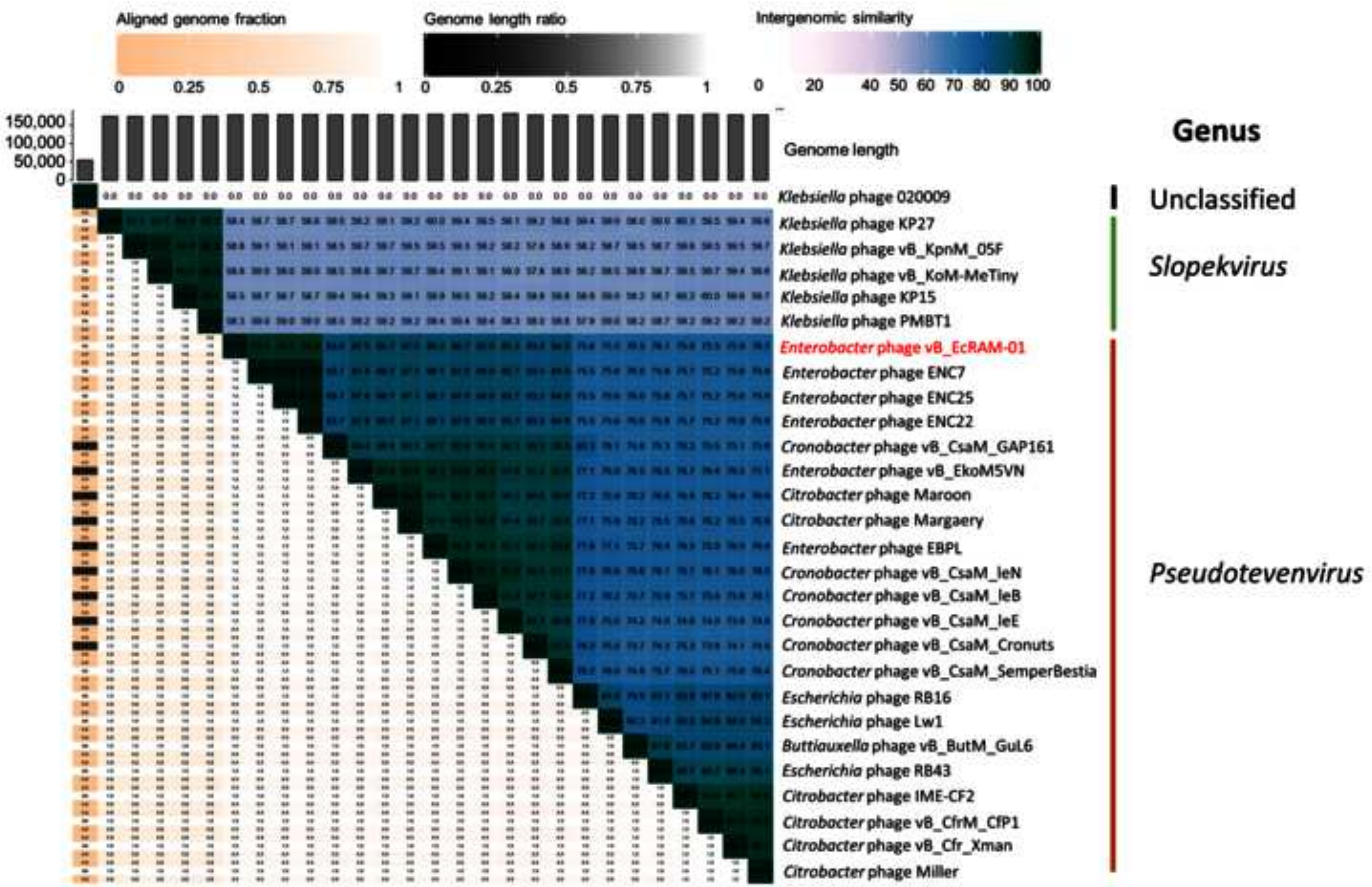

Figure 2

Click here to access/download;Figure;Fig2.tif

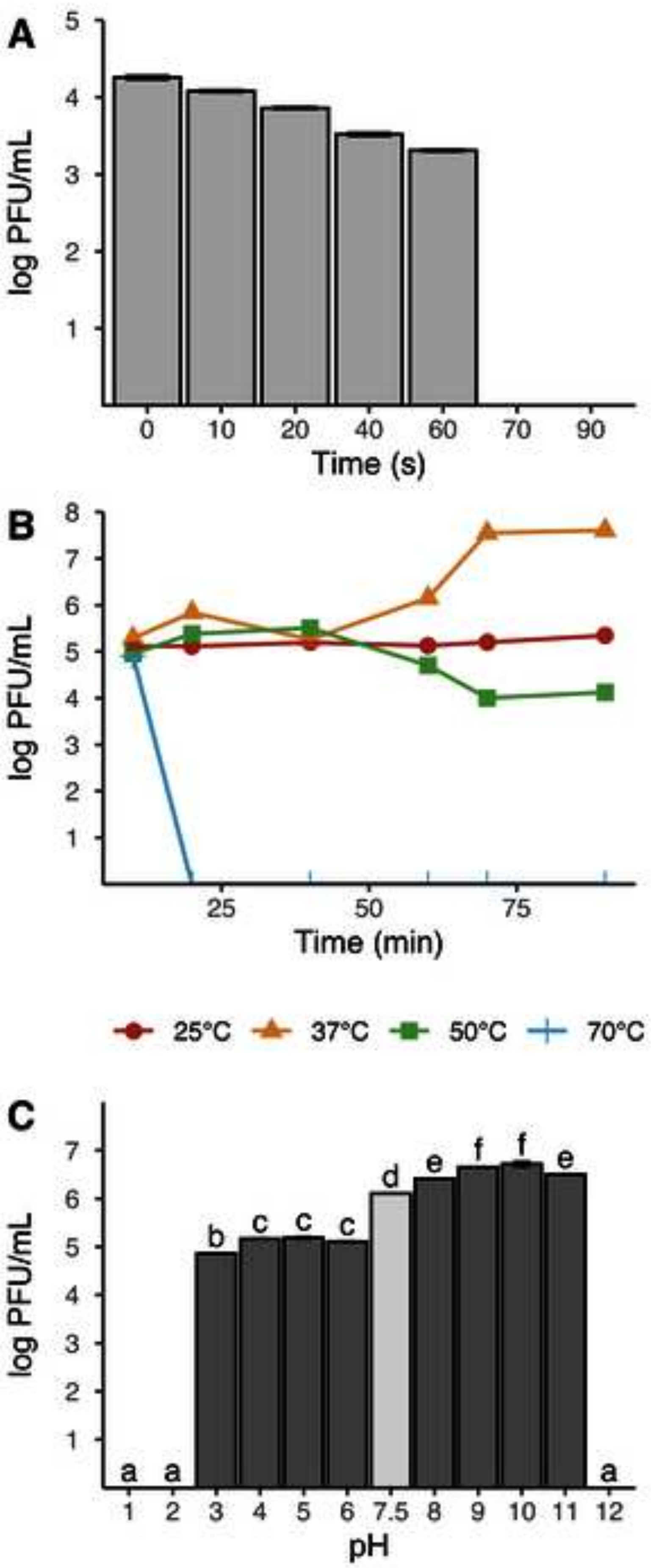

Click here to access/download
Supporting Information
Suppl. Information.docx

Characterization of Enterobacter phage vB_EcRAM-01, a new *Pseudotevenvirus* against *Enterobacter cloacae*, isolated in an urban river in Panama.

Ednner E. Victoria-Blanco[1,2,7,¶], Jean Pierre González-Gómez[4¶], Juan Raúl Medina-Sánchez[2], Alexander A. Martínez[1,6,7], Nohelia Castro del Campo[4], Cristóbal Chaidez-Quiroz[4], Jordi Querol-Audi[1,2,5,7,*], Alex Omar Martínez-Torres[2,3,7,*]

[1] Programa de Maestría en Ciencias Biomédicas, Facultad de Medicina, Universidad de Panamá, Panamá.

[2] Laboratorio de Microbiología Experimental y Aplicada y Microbiología de Aguas (LAMEXA-LAMA), Universidad de Panamá, Panamá.

[3] Departamento de Microbiología y Parasitología, Facultad de Ciencias Naturales, Exactas y Tecnología, Universidad de Panamá, Panamá.

[4] Laboratorio Nacional para la Investigación en Inocuidad Alimentaria (LANIIA), Centro de Investigación en Alimentación y Desarrollo, A.C. (CIAD), Culiacán, Sinaloa, México.

[5] Departamento de Bioquímica y Nutrición, Facultad de Medicina, Universidad de Panamá, Panamá.

[6] Genomics and Proteomics Research Department, Gorgas Memorial Institute of Health Studies, Panamá, Panamá.

[7] Sistema Nacional de Investigación (SNI). Secretaría Nacional de Ciencia, Tecnología e Innovación (SENACYT), Panamá.

[*]Corresponding authors:

E-mail: alex.martinez@up.ac.pa (AOMT) and jordi.querol@up.ac.pa (JQA)

[¶]These authors contributed equally to this work and share first authorship.

Abstract

The *Enterobacter cloacae* complex, a prominent bacterium responsible worldwide for most bloodstream infections in the hospital environment, has shown broad-spectrum antibiotic resistance, including carbapenems. Therefore, bacteriophages have again attracted the attention of the science and medical community as an alternative to control Multidrug resistant bacteria. In this study, water samples from Río Abajo River, in Panama City, Panama, were collected, for phage isolation, purification, characterization and propagation against the *E. cloacae* complex. As result, a phage produced clear and round plaque-forming units indicating a lytic phage was isolated. Further analyses concluded that this phage is stable at temperatures between 25°C and 50°C, it remains infective in a pH range between 7 to 11, with high sensitivity to Ultraviolet light. Remarkedly, it exhibits a narrow host specificity only infecting *E. cloacae*. Whole genome sequencing revealed that is a myovirus with a genome size of 178,477 bp, a G-C content of 45.8%, and containing approximately 294 genes. Among them, protein-encoding genes involved in morphology, inactivation, adsorption to cells, DNA injection and lytic enzymes were identified. Additionally, the genome contained two tRNA sequences. Genes that encode holins and endolysins, typical of lytic bacteriophages, were also present. A whole-genome sequencing analysis indicated that, according to the genus demarcation criteria, this phage belongs to a novel species within the Family *Straboviridae*, called genus *Pseudotevenvirus*.

Introduction

Enterobacteriaceae are ubiquitous in nosocomial settings and can play important roles in the burden of disease in the urinary tract, lower respiratory tract, intra-abdominal organs, and skin infections. The most relevant members of this Gram-negative bacterial group are *Klebsiella pneumoniae*, *Escherichia coli*, and *Enterobacter* spp. The severity of the infections is intensified by the resistance level to most available antibiotics, including carbapenem and cephalosporins [1]. Special attention to *Enterobacter* is due to the intrinsic resistance to ampicillin and broad-spectrum cephalosporin which reduces the alternatives for antibiotic treatments. *Enterobacter* belongs to the ESKAPE group (acronym for *Enterococcus faecium*, *Staphylococcus aureus*, *Klebsiella pneumoniae*, *Acinetobacter baumannii*, *Pseudomonas aeruginosa*, and *Enterobacter* spp), related worldwide to severe nosocomial infections with a highly increasing multi-drug resistance. A problem of epidemiological importance is the presence of AmpC-producing strains together with BLEE-encoding plasmids and colistin co-resistance in *E. cloacae* complex (ECC) strains **[2–4]**. The continuous emergence of multi-drug resistant strains have hampered the effectiveness of antibiotic treatments. Therefore, one possible approach to address this problem is the use of bacteriophages. In itself, the

magnitude of the impact of phage predation on pathogenic bacteria has been demonstrated. In recent years, phage therapy has been successfully applied to *E. coli* O157:H7, causative agent of hemorrhagic colitis and hemolytic uremic syndrome **[5–7]**. The use of phages has also been studied in the treatment of peritoneal infections, otitis externa, in the eradication of *P. aeruginosa* in patients with cystic fibrosis [8,9], in the treatment of urinary infections or osteomyelitis caused by *S. aureus* [10]. The favorable effects of phage therapy may even exceed its antibacterial activity. A surprising number of data has demonstrated the *in vivo* activity of phages such as the adenovirus growth suppression mediated by bacteriophage T4 [11] or development inhibition of *P. aeruginosa* by phage Pf4 [12].

In recent years, several studies have been published on the use of bacteriophages against *E. cloacae*. Isolation and Characterization of Lytic Phages: A recent study focused on the isolation of the phage ECLFM1 from sewage water using a carbapenem-resistant clinical strain as the host. ECLFM1 exhibited rapid adsorption and a 15-minute latent period, with a burst size of approximately 75 PFU/infected cell. This phage belongs to the Karamvirus family and demonstrated high effectiveness in therapeutic applications, increasing survival rates in zebrafish infected with E. cloacae [13]. Another study identified and characterized the phage vB_EclM_Q7622, isolated from sewage in a poultry farm. This phage showed remarkable capability to infect and destroy multi-drug-resistant strains of *E. cloacae*, highlighting its potential as a therapeutic agent in clinical infections [14]. Phages PϕEn-CL and PϕEn-HO demonstrated the ability to eliminate multi-drug-resistant *E. cloacae* strains. The study emphasized the specificity of these phages and their efficacy in destroying bacterial biofilms, which is crucial for treating persistent infections in hospital settings [15].

The principal advantage of employing phages in therapy and biological control lies in their specificity towards bacterial targets. Those that do not infect commensal bacteria can be selected, therefore posing no threat to humans and animals. Their widespread presence in nature, their versatility, and their inherent resilience have elevated their status from mere research organisms to potentially viable and indispensable tools in the battle against growing antimicrobial resistance. However, the transfer of resistance and virulence genes, and other genes associated with negative outcomes through bacteriophages, constituted to an entire research domain aimed at mitigating any potential adverse impacts [16,17]. For these reasons, this study is aimed to isolate and biologically and genetically characterize a phage against the *E. cloacae* complex.

Methods

Bacterial Strains and Growth Conditions.

The host strain ATCC 23355 of *E. cloacae* complex used for the isolation and proliferation of the phage was the same as previously reported [18]. In addition, *S. aureus*, *E. coli*, *E. aerogenes*, *E. cloacae, and P. aeruginosa*, provided by the Human Microbiology Department, Faculty of Medicine, Universidad de Panamá; Hospital Santo Tomas, and Complejo Hospitalario Dr. Arnulfo Arias Madrid, were used for host range determination. The *E. cloacae* host strain, along with twenty-seven clinical isolates of *E. cloacae*, were tested for antibiotic susceptibility using the Vitek II Automated Microbiological System (BioMérieux, Marcy l'Étoile, France) (results shown in Supplementary Table 1). The breakpoints were applied according to the Clinical and Laboratory Standards Institute (CLSI) guidelines [19].

Ethics Statement.

The Bioethics Committee of the Universidad de Panamá with the endorsement of the Dirección General de Salud Pública of the Ministerio de Salud (Ministry of Health), approved this study (Sequential: 617).

Phage isolation.

The Enterobacter phage vB_EcRAM-01 was isolated from waters of Rio Abajo river in Panama City, Panamá (coordinates, 9°00′55.2″N 79°29′25.6″W), using *E. cloacae* strain ATCC 23355 as a propagation host, which was grown previously in tryptic soy broth at 37°C for 24 h. Phage isolation, propagation, and purification were performed using the soft-agar overlay method [20]. Briefly, phage purification process involved serial dilution of filtrates exhibiting lytic activity against *E. cloacae*. Specifically, 100 µL of the phage dilution was mixed with an overnight culture of the host bacterium in 3 mL of soft agar (TSB with 0.4% agar), poured onto TSA plates, and incubated at 37 °C for 24 h. The phage plates, selected based on size and clarity, were transferred to microtubes containing 1 mL of nanopure water and the whole process was repeated at least three times to confirm that the isolated phages were descendants of a single virion.

Phage concentration.

The soft-agar overlay method was used to propagate the isolated phage. 5 mL of phage buffer (40 mM Tris-Cl, 150 mM NaCl, 10 mM $MgSO_4$) were added to each Petri dish and left for 24 hours at 4°C. Then the buffer was recovered with a serological pipette and subsequently, the top agar was scraped. Sample was centrifuged at 4,000 rpm for 15 minutes and the supernatant was recovered and centrifugated again was. The supernatant was recovered and filtered using a 0.22 µm filter. The phage sample was then incubated with PEG8000 (8%w/v final concentration) in an automatic tube mixer O/N at 4°C. Sample was

centrifuged at 6,000 rpm for 15-30 minutes, the supernatant was discarded, and the pellet was gently resuspended with 1 mL of sterile PBS buffer.

Host range of vB_EcRAM-01 against clinical *E. cloacae* isolates: Spot test and efficiency of plating (EOP).

Clinical isolates of *E. cloacae, S. aureus, E. coli, P. aeruginosa*, and *E. aerogenes*, along with *E. cloacae* and *E. aerogenes* ATCC reference strains, were cultured until reaching the exponential growth phase (OD_{600nm} 0.6). Subsequently, 200 - 500 µL of each culture were mixed with 3 mL of top agar containing 10 mM $CaCl_2$ and plated onto a 1.5% nutrient agar plate. After solidification, a single droplet (10 µL) from each phage dilution (10^{-1} to 10^{-8}) was applied onto the agar [21].The droplets were allowed to dry for 10 min and plates were incubated at 37°C for 24 h - 48 h. A phage titer in PFU/mL was calculated for each strain tested [22]. Phage virulence was assessed using the efficiency of the plating (EOP) method. The EOP was determined by dividing the phage titer at the final dilution on the test strain by the phage titer on its original isolation strain. Based on these calculations, phages were categorized into highly virulent (0.1 < EOP > 1.00), avirulent but still active (EOP < 0.001), or completely avirulent (no plaques were observed).

Morphological characterization.

The isolated phage underwent examination using transmission electron microscopy (TEM). A formvar-coated grid (400 mesh copper grid) was utilized, onto which a drop of 10^9 PFU/mL phage stock was applied. Subsequently, negative staining was performed with 2 % phosphotungstic acid (pH 7.2) for 5 minutes, and excess stain was eliminated with filter paper. Electron micrographs were captured at varying magnifications using a JEOL JEM-1011 transmission electron microscope (20).

Sodium dodecyl sulphate polyacrylamide gel electrophoresis (SDS-PAGE).

SDS-PAGE electrophoresis was performed following the Laemmli method [23]. Separation was carried out in 10% resolving gel in standard Tris–glycine buffer at a constant current of 20 mA. A molecular weight standard (Precision Plus protein ladder, BioRad) was used in the electrophoretic separation, after which the gels were stained with Coomassie blue

(Sigma, Ge). After incubation with a solution containing 20% ethanol and 7% acetic acid to remove the excess of stain, images were recorded using a BioDoc-it Imaging System (UVP).

Effects of UV light, temperature and pH on the viability of phage vB_EcRAM-01.

Purified vB_EcRAM-01 was exposed to different physicochemical conditions (UV light, temperature and pH) Suspensions were exposed to ultraviolet light at a distance of 50 cm for 10, 20, 40, 60, 70, and 90 s. To determine the effect of temperature, phage suspensions were placed at 25 ºC, 37 °C, 50 ºC and 70 ºC, respectively. pH stability was determined by mixing assay vB_EcRAM-01 phage samples resuspended in SM-gelatine buffer (50 mM Tris–HCl pH 7.5, 100 mM NaCl, 8 mM $MgSO_4$ and gelatin 2% (w/v)) with a pH ranging from 2 to 14. Phage viability was determined by counting the number of lysis PFU using the double-layer agar technique and the experiments were carried out in triplicates [24,25].

Bacterial reduction assay.

Bacteriolytic activity was evaluated using a spectrophotometer, targeting *E. cloacae* ATCC 23355. The bacterial strain's overnight culture, adjusted to 1.0 OD_{600nm}, served as the inoculum. A concentration of 8 log PFU/mL was tested with appropriate phage dilutions. Each tube, including negative and positive controls, contained 20 mL of TSB. Experimental tubes received an additional 200 µL of the phage dilution, followed by the addition of 1000 µL of bacterial culture to each tube (excluding the negative control). Incubation was carried out for 180 minutes at 37 °C, with measurements at 600 nm taken every 15 minutes. The experiment was conducted in triplicates.

Bacteriophage DNA isolation and genome sequencing.

The QIAamp MinElute Virus Spin kit (QIAGEN, Valencia, CA) was used to extract DNA, according to the manufacturer´s instructions. The purity was assessed spectrophotometrically at 260 and 280 nm, using a BioSpec-nano Micro-volume UV-Vis Spectrophotometer (Shimadzu, Kyoto, Japan).

The DNA libraries were prepared using the Nextera XT DNA Library Preparation Kit (Illumina, San Diego, CA, USA) through fragmentation, PCR amplification, PCR cleanup, and library normalization. The genome was sequenced at the Genomics and Proteomics Research Department of ICGES using the MiSeq Illumina sequencer (2 x 250 bp paired-end reads) (ThermoFisher

Scientific Waltham, Massachusetts USA). Raw reads (629,372 reads in total) were checked for quality using FastQC (www.bioinformatics.Babraham.ac.UK/projects/fastqc) and trimmed using Timmomatic v0.39 [26].

Genome annotations, comparative genomics and phylogenetic analyses.

The genome was assembled through SPAdes v3.15.1 [27], resulting in a single contig with 8.96-fold coverage [27]. We mapped the reads against the contig obtained using Bowtie v0.9.6 (http://bowtie-bio.sourceforge.net/index.shtml) to corroborate the sequence ends [28]. The phage genome annotation was performed using RASTk v2.0 [29–31]. Gene calling was performed through GeneMarkS v4.28 (http://exon.gatech.edu/GeneMark/genemarks.cgi) [32], and Glimmer v3.02 (http://ccb.jhu.edu/software/glimmer/index.shtml) [33,34].

Several methods were used to annotate the ORF functions, including the protein basic local alignment search tool (blastp) on the NCBI server (https://blast.ncbi.nlm.nih.gov/Blast.cgi) based on the search of the non-redundant protein sequence database [35], Hhpred using structural/domain database (https://toolkit.tuebingen.mpg.de/tools/hhpred) [36–39] and HMMER v2.41.1 through HMM database [40,41]. (https://toolkit.tuebingen.mpg.de/tools/hhpred). Using a non-redundant protein matrix, employing Markov hidden model profiles [42], the annotations were manually curated using the Geneious v9.1.8 platform employing default parameters. Identification of transfer RNAs was performed using tRNA scan-SE v2.07 (http://lowelab.ucsc.edu/tRNAscan-SE/) and ARAGORN v1.2.41 [43] software (http://www.ansikte.se/ARAGORN/). Comparative analysis between genomes was performed using Mauve progressive alignment v2.3.1 [44] (http://darlinglab.org/mauve/mauve.html) employing default parameters.

Pairwise comparison of nucleotide sequences was performed using the Genome-BLAST Distance Phylogeny (GBDP) method of the VICTOR (Virus Classification and Tree Building Online Resource) platform [45] (https://ggdc.dsmz.de/victor.php), in the recommended environments for prokaryotic viruses. The resulting intergenomic distances were used to infer a branch-supported balanced minimum evolution tree using FASTME, including SPR post-processing [46] using D6 formula. Branch support was inferred from 100 pseudo-bootstrap replicates each. Trees were rooted at the midpoint [47], visualized using FigTree; personalized with taxon boundaries at species, genus, and family scales; and estimated with the iTOL program [48] (https://itol.embl.de/). The recommended clustering threshold is thirthy nine [45] and an F-value (fraction of links required for cluster fusion) of 0.5 [45].

Determination of taxonomy and viral replication cycle type was achieved using PATRIC v3.6.9 [49] (https://patricbrc.org/) and PhageAI (https://phage.ai/) server [50]. The genome comparison between Enterobacter phage

vB_EcRAM-01 and close relatives was performed using EasyFig v2.2.2 [51]. At the end, the intergenomic similarities amongst complete viral genomes were calculated through the VIRIDIC web tool [52] with blastn default settings.

Statistical analysis.

Statistically significant differences were determined using ANOVA. Differences in group means were compared using Tukey's HSD test. A P-value below 0.05 was considered statistically significant. Statistical analysis was conducted using R version 4.3.1 [53].

Results

Bacteriophage isolation, host range analysis and morphology characterization

In recent years, several studies have been published on the use of bacteriophages against E. *cloacae,* an opportunistic pathogen posing a clinical challenge due to its resistance to multiple antibiotics[13–15]. In this study, the Enterobacter phage vB_EcRAM-01 was isolated from the waters of the Rio Abajo river in Panama City, Panama. Using *E. cloacae* strain ATCC 23355 as the host, the phage was isolated and purified. The isolation involved a series of dilutions and the use of the soft-agar overlay technique to propagate the phage. Clear plaques indicating phage lytic activity were selected and purified to ensure that the isolated phages originated from a single virion.

In order to evaluate the host specificity of vB_EcRAM-01, a comprehensive assessment was conducted involving a total of thirty-three strains: two ATCC strains alongside a diverse selection of, both, Gram-negative and Gram-positive clinical isolates (Fig 1).

Fig 1. Host range specificity and efficiency of plating. The strain code followed by the efficiency value are shown in parentheses. Color range code associated with EOP is indicated on the right.

Notably, vB_EcRAM-01 exhibited the capacity to infect all the *E. cloacae* isolates with varying degrees of efficacy while displaying negligible antimicrobial activity (efficiency < 0.10) against the other species tested, which included *E. coli, E. aerogenes, A. baumannii, S. aureus*, and *P. aeruginosa*. Upon infecting the reference strain, the clear plaque-forming units (PFUs), characterized by smooth perimeters, measured within the range of 3 mm to 7 mm in diameter, a hallmark indicative of its lytic phage attributes (S1 Fig). TEM micrographs of the phage showed a morphology typical of a myovirus, a phage with a

tail, belonging to the class *Caudoviricetes*. The size measurements of its icosahedral head are about 104 x 76 nm, and its contractile tail is approximately 110 nm (S2 Fig). Previously, phage morphology was sufficient to classify them taxonomically, but nowadays bioinformatics tools are used to perform similarity analysis and group them by evolutionary distances.

Environmental stress resistance: stability against UV light, temperature and pH

Exposure to UV light (365 nm) decreased the infectivity of phage vB_ECRAM-01 over time. Results showed an exponential decay resulting in complete inactivation when incubated for more than one minute (Fig 2A).

Fig 2. Stability of vB_ECRAM-01 to (A) UV radiation, (B) temperature, and (C) pH. Data is expressed as mean ± standard error. Treatments with the same letters in panel (C) are not significantly different (Tukey's HSD test, $p>0.05$).

The phage exhibited lytic activity when subjected to temperatures ranging from 25 °C to 50 °C, even after being incubated for 90 minutes (Fig 2B). However, when exposed to 70 °C, the phage became completely inactivated after only 20 min of incubation and, at 80 °C, it lost its infectivity entirely (data not shown for clarity).

The stability of vB_EcRAM-01 was determined at various pH values. It maintained its infectivity in a pH range between pH 7.5 and pH 11. The best conditions were found between pH 9 and 10 (Fig 2C). Incubation under acidic pH resulted in a significant reduction of viral proliferation. The phage was not able to replicate in a pH greater than 11 or less than 3. Overall, the best activity was found in alkaline media.

Bacteriolytic activity

The evaluated phage demonstrated a robust, complete bacteriolytic effect in the initial 105 minutes of the assay. Following this, the OD curve started to rise due to an increase in bacterial cell numbers. This trend persisted in subsequent measurements, although the rate of increase was lower than that observed in the positive control (Fig 3).

Fig 3. Effect of vB_EcRAM-01 on the growth of the E. cloacae complex. The light blue line indicates the bacterial concentration without being infected with the virus at 37°C, showing an absorbance of 1.6. On the other hand, the orange line shows the concentration of the bacteria being infected from the beginning, with a reduced absorbance of up to 0.77 (ANOVA $F = 613.62$. g.l = 1 and $p<0.0001$).

Comparative genomics and phylogenetic analysis

The genomic attributes of phage vB_EcRAM-01 were delineated as follows: the genome spans 178,477 base pairs with a 45.0 % of GC content, hosting a total of 293 genes. Among these, 86 genes were implicated in anticipated functions encompassing morphology, inactivation, cellular adsorption, DNA injection, and host lysis. Through a UniProt database blast search, we were able to identify to structural proteins, one sharing 97% homology with the Major capsid protein (ID: A0A1B1IX95) of Citrobacter phage vB_CfrM_CfP1, having a mass of 56.37 kDa [54] while the other protein exhibits 96.7% homology with the Baseplate wedge subunit protein (ID: M9V1F2) from Escherichia phage Lw1, with a mass of 38.01 kDa [55]. In fact, analysis by polyacrylamide gel electrophoresis revealed two prominent protein bands with molecular masses close to 50 and 37 kDa, respectively (S3 Fig). In bacteriophages possessing both capsid and tail structures, SDS-PAGE typically reveals multiple proteins, including capsid, tail, and baseplate proteins. Capsid proteins form the outer shell encasing the phage genome, while baseplate proteins are situated at the tail base, facilitating recognition and binding to specific receptors on host cell surfaces [56]. Hence, it is plausible that the observed proteins serve these functions; however, precise identification through LC-MS/MS analysis would provide a more definitive comparison [57].

Remarkably, the genome of vB_EcRAM-01 was lacking discernible genes associated with resistance, virulence, or allergenicity. The genomic blueprint encodes two transfer RNAs (tRNAs), one corresponding to methionine (cat) and the other to glycine (tcc). A pertinent insight is the substantial sequence identity—90.11%—shared with other phages (Fig 4) [18].

Fig 4. Comparison of Enterobacter phage vB_EcRAM-01 genome with close relatives. Color scales represent the percentage of nucleotide identity between regions obtained by blastn.

To assign a taxonomic classification to phage vB_EcRAM-01, a phylogenetic tree based on whole-genome was constructed, and intergenomic similarities were calculated between the closely related genomes reported in GenBank. The phylogenetic tree and intergenomic similarity heatmap revealed that phage vB_EcRAM-01 is a new member of the *Pseudotevenvirus* genus within the *Straboviridae* family.

The GBDP phylogenetic tree using the D6 formula with an average support of 31%, identifying ten and nine clusters, respectively, at the genus level. At the family level, there were five and six clusters, respectively (Fig 5).

Fig 5. Phage vB_EcRAM-01 genomic and phylogenetic analyses. Genome Blast Distance Phylogeny (GBDP)-based phylogenetic tree of vB_EcRAM-01 and 28 related Straboviridae members created by iTOL v6.0 platform. The Enterobacter phage vB_EcRAM-01 belongs to the genus Pseudotevenvirus and is a new phage species.

Nucleotide identity analysis (ANI) was done on the 28 genomes with the highest homology in the GenBank database using VIRIDIC. According to the results of the heat mapping, phage vB_EcRAM-01 shares less than 95% nucleotide identity with previously reported genomes, thereby establishing it as a novel species within the Family *Straboviridae*, genus *Pseudotevenvirus*, according to the genus demarcation criteria (>70% nucleotide identity over the entire genome length) recommended by the ICTV (Fig 6).

Fig 6. Taxonomic classification of Enterobacter phage vB_EcRAM-01. A heatmap created by VIRIDIC based on intergenomic similarities between viral genomes, with the proportion of similarity shown by the color scale.

Comparative BLASTn analysis of the complete genome of vB_EcRAM-01 against phages in the GenBank database revealed that vB_EcRAM-01 has a high degree of coverage and identity with the Enterobacter phages ENC22 (OL355128.1), ENC7 (OL355125.1), ENC25 (OL355127.1), EBPL (MT341500.1). In VIRIDIC analysis, the phages shared collinear gene blocks that are identical in length between the two genomes. In comparison, other blocks revealed a high degree of similarity between this phage and others, including Cronobacter phage vB_CsaM IeE (NC_048646.1), Cronobacter phage vB_CsaM IeN (KX431560.1), Citrobacter phage Margaery (KT381880.1), and myoviral members of the subfamily *Tevenvirinae*.

Discussion

In Panama, seven out of the eight bacterial pathogens responsible for 80% of the reported infections concerning the emergence of resistance in a hospital level (ESKAPE) has been identified: *S. aureus* (16%), *Enterococcus* spp (14.4%), *E. coli* (12%), coagulase-negative *Staphylococcus* (11%), *K. pneumoniae* and *K. oxytoca* (8%), *P. aeruginosa* (8%), and *Enterobacter* spp. (5%). Several microorganisms developed antibiotic resistance to the more common antibiotics to treat these bacterial infections like piperacillin-tazobactam, cephalosporins, nitrofurans, and carbapenems [58,59].

Therefore, in comparison to the usage of antibiotics, phage therapy is a viable option. While scientific publications on phage isolation have increased in recent years, only a small number of phages have been commercialized [60]. One of the

primary reasons is a lack of adequate characterization, which is required to detect and mitigate the hazards associated with phage therapy [61,60]. Moreover, the biological characteristics of a phage determines its stability and therefore, its optimum administration forms [61].

As a first step, vB_EcRAM-01was subjected to environmental stress tests, such as temperature, UV radiation and pH, factors that play a role in phage survival [62,63]. The results indicate that vB_EcRAM-01 is stable at temperatures ranging from 25°C to 50°C, although its replication rate is highest at 37°C. This aligns with findings observed in Myoviridae phages [64,65] exhibiting a comparable stability profile within a broad temperature range. However, it deviates from the EBP phage, known for its resilience across a wider temperature span [66]. Notably, inactivation under UV light transpires rapidly, underscoring the necessity of safeguarding possible clinical applications from sunlight exposure. Interestingly, vB_EcRAM-01 maintains its infectivity across expansive pH and temperature ranges, implying potential robustness within the human body. Enhancements through administration systems designed to shield the phage from gastrointestinal pH could potentially further increase its utility and stability in therapeutic applications [67].

The primary objective of this study was to investigate the host specificity (host range) of phage vB_EcRAM-01 concerning isolates within the *E. cloacae* complex. Accurate identification of the host bacteria's specificity for bacteriophage vB_EcRAM-01 is crucial for a comprehensive characterization of this bacteriophage in order to evaluate a potential therapeutic use. Our observations indicate that this bacteriophage displayed reactivity only with its host strain. These results are in line with findings reported for phages TSE1, TSE2, and TSE3 [68]. *E. coli* displayed some sensitivity but did not exhibit complete lysis. Exceptions to this pattern were observed with myophages *Enterobacter* phage ENC22, *Enterobacter* phage ENC7, and *Enterobacter* phage ENC25, which demonstrated the ability to infect diverse *Enterobacter* species [69].

Apart from meeting the requirements of being innocuous to normal microbiota, therapeutic use involves the employment of fully lytic phages that lack undesired virulence factor genes or antibiotic resistance genes [70]. The genomic assessment of the phage vB_EcRAM-01 confirmed its status as a strictly lytic phage according to the PhageAI platform. The genome contained genes coding for holins and endolysins, offering several advantages. These enzymes collaborate to facilitate bacterial lysis, enabling the release of newly assembled viral particles and the termination of the bacterial host [71]. Additionally, holin proteins play a crucial role in regulating the timing of lysis, ensuring that it occurs at the appropriate stage of the phage life cycle [72]. An important aspect to note is that resistance to endolysins has not been reported to date [73]. This suggests a higher probability of success when employing phages containing endolysins. Besides, it lacked genes associated with lysogeny, such as integrases, transposons, and excitons [74].

The evolutionary context designated it as a T4-type myophage spanning a genome length of 178,477 base pairs. Computational analysis further classified it as a viral entity within the Class *Caudoviricetes*, *Strabovviridae* family, and *Pseudotevenvirus* genus [74]. This type of virus is characterized by the presence of tRNA. Due to the compact nature of phage genomes, which constrains their capacity to utilize host resources fully, these genetic elements may serve as compensation for compositional differences between phages and hosts. They have the potential to supplement the translation machinery with their genetic information, thereby improving fitness and fortifying virulence [75].

By scanning the GenBank database for homologous sequences, a 92% sequence homology with myophage EBPL was observed, a phage that also infects *E. cloacae* [66], and 99.16% sequence homology with myophages ENC7, ENC25, and ENC22 [69].

According to genotypic characteristics [76], vB_EcRAM-01, like an EBP phage, is a myovirus-like phage [66]. The vB_EcRAM-01 virus was termed a bacterial infecting virus isolated from Rio Abajo waters that suppressed *E. cloacae*, a myophage, according to the informal phage nomenclature criteria [77–79].

In this study, the genome-wide syntenic analysis indicated that both phages had comparable rearrangements but differed slightly in nucleotide sequence. Additionally, a phylogenetic study of whole-genome sequences showed that both phages diverged from a common ancestor but experienced many nucleotides substitution events since then [80]. Given that EBPL was previously described as a singleton phage in the myophage phylogenetic tree [81], phylogenetic and nucleotide identity analyses indicated that vB_EcRAM-01 might represent a previously unreported species.

Therefore, vB_EcRAM-01 is physiologically and molecularly stable under a wide temperature and pH range. However, it has a restricted host range. Although it had intense lytic activity against the *E. cloacae* complex, isolation was deemed necessary for more efficient eradication.

In Conclusion, this study identified a lytic bacteriophage. The characterization enhances comprehension of this novel phage, and through its phylogenetic relatives, it may exhibit a myovirus-like morphology, as demonstrated by insights from next-generation sequencing. This sequencing elucidates that vB_EcRAM-01 aligns with the Myoviridae family in accordance with the conventional taxonomic classification [82]. According to the phylogenetic analysis previously described, this virus is a new member of the family *Straboviridae* and the genus *Pseudotevenvirus* according to the new classification [83]. The vB_EcRAM-01 strain is also a viable candidate for many clinical applications, including phage therapy and biologic control or decontamination, due to its host specificity, the genetic link, and lack of lysogenic genes.

Data availability statement

The datasets presented in this study can be found in online repositories. The names of the repository/repositories and accession number(s) can be found at https://www.ncbi.nlm.nih.gov/genbank/ , OL551674, SAMN23426491, SRR18344380.

Author Contributions

EEVB and JPGG drafted the manuscript, while JRMS conducted the statistical analysis of the results. NCC and CCHQ, along with JQA, AAM and AOMT, contributed to data analysis and manuscript revisions. EEVB, JQA, AAM and AOMT secured project funding. All authors reviewed and approved the submitted manuscript.

Acknowledgments

Host bacterial strains were provided by José E. Moreno, MSc., of the Microbiology Division of Laboratorio Central de Referencia en Salud Pública, Instituto Conmemorativo Gorgas de Estudios de Salud (ICGES). We extend our gratitude to the staff of the Microbiology Department at the Clinical Laboratory of Hospital Santo Tomas for providing us with the clinical bacteria strains.

Additionally, we would like to thank Claudia González, MSc, Jessica Gondola, MSc, Ambar Moreno, MSc, and Oris Chavarria, MSc, of the Research Department of Genomics and Proteomics of ICGES for providing us with NGS Illumina sequencing. Furthermore, we would like to express our gratitude to Leandra Gómez, MSc, of the Department of Pharmacology at the Faculty of Medicine, Universidad de Panamá, for her invaluable contribution in reviewing the bioethical considerations within this research. Also, thank you to Bs. Tatiana A. Martínez González for her contribution to the English revision of the present work.

Bibliography

1. WHO. Global priority list of antibiotic-resistant bacteria to guide research, discovery, and development of new antibiotics. 2017.

2. Qian W, Fu Y, Liu M, Zhang J, Wang W, Li J, et al. Mechanisms of Action of Luteolin Against Single- and Dual-Species of Escherichia coli and Enterobacter cloacae and Its Antibiofilm Activities. Appl Biochem Biotechnol. 2021;193: 1397–1414. doi:10.1007/s12010-020-03330-w

3. Davin-Regli A, Lavigne J-P, Pagès J-M. Enterobacter spp.: Update on Taxonomy, Clinical Aspects, and Emerging Antimicrobial Resistance. Clin Microbiol Rev. 2019;32. doi:10.1128/CMR.00002-19

4. Mezzatesta ML, Gona F, Stefani S. *Enterobacter cloacae* complex: clinical impact and emerging antibiotic resistance. Future Microbiology. 2012;7: 887–902. doi:10.2217/fmb.12.61

5. Amarillas L, Rubí-Rangel L, Chaidez C, González-Robles A, Lightbourn-Rojas L, León-Félix J. Isolation and Characterization of phiLLS, a Novel Phage with Potential Biocontrol Agent against Multidrug-Resistant Escherichia coli. Frontiers in Microbiology. 2017;8. Available: https://www.frontiersin.org/articles/10.3389/fmicb.2017.01355

6. López-Cuevas O, Castro-Del Campo N, León-Félix J, Valdez-Torres B, Chaidez C. Evaluation of Bacteriophage Av-08 for Simultaneous Biocontrol of Salmonella Montevideo and Escherichia Coli O157:h7 in Experimentally Contaminated Chicken Skin. Journal of Food Safety. 2012;32: 305–310. doi:10.1111/j.1745-4565.2012.00381.x

7. Sharma M, Patel JR, Conway WS, Ferguson S, Sulakvelidze A. Effectiveness of Bacteriophages in Reducing Escherichia coli O157:H7 on Fresh-Cut Cantaloupes and Lettuce. Journal of Food Protection. 2009;72: 1481–1485. doi:10.4315/0362-028X-72.7.1481

8. Morello E, Saussereau E, Maura D, Huerre M, Touqui L, Debarbieux L. Pulmonary Bacteriophage Therapy on Pseudomonas aeruginosa Cystic Fibrosis Strains: First Steps Towards Treatment and Prevention. Aziz R, editor. PLoS ONE. 2011;6: e16963. doi:10.1371/journal.pone.0016963

9. Debarbieux L, Leduc D, Maura D, Morello E, Criscuolo A, Grossi O, et al. Bacteriophages Can Treat and Prevent *Pseudomonas aeruginosa* Lung Infections. J INFECT DIS. 2010;201: 1096–1104. doi:10.1086/651135

10. Abedon ST. Commentary: Phage Therapy of Staphylococcal Chronic Osteomyelitis in Experimental Animal Model. Front Microbiol. 2016;7. doi:10.3389/fmicb.2016.01251

11. Przybylski M, Dzieciątkowski T, Borysowski J, Międzybrodzki R, Górski A. Inhibitory Effects of Bacteriophage Preparations on Adenoviral Replication. INT. 2019;62: 37–44. doi:10.1159/000500540

12. Casto A, Hurwitz A, Kou K, Mansour G, Policke R, Schmidt A, et al. Bacteriophages: The Answer to Antibiotic Resistance? James Madison Undergraduate Research Journal. 20163: 36–41.

13. Ali SF, Teh S-H, Yang H-H, Tsai Y-C, Chao H-J, Peng S-S, et al. Therapeutic Potential of a Novel Lytic Phage, vB_EclM_ECLFM1, against Carbapenem-Resistant Enterobacter cloacae. Int J Mol Sci. 2024;25: 854. doi:10.3390/ijms25020854

14. Nair A, Vyawahare R, Khairnar K. Characterization of a novel, biofilm dispersing, lytic bacteriophage against drug-resistant Enterobacter cloacae. J Appl Microbiol. 2022;132: 2721–2732. doi:10.1111/jam.15420

15. Rahimzadeh Torabi L, Doudi M, Naghavi NS, Monajemi R. Bacteriophages PϕEn-CL and PϕEn-HO can

eliminate MDR *Enterobacter cloacae* and *Enterobacter hormaechei* isolated from burn wound infections without toxicity for human skin cells. FEMS Microbiology Letters. 2021;368: fnab143. doi:10.1093/femsle/fnab143

16. Hassan AY, Lin JT, Ricker N, Anany H. The Age of Phage: Friend or Foe in the New Dawn of Therapeutic and Biocontrol Applications? Pharmaceuticals. 2021;14: 199. doi:10.3390/ph14030199

17. Häusler T. Viruses vs. Superbugs. London: Palgrave Macmillan UK; 2006. doi:10.1007/978-0-230-55228-9

18. Victoria-Blanco EE, González-Gómez JP, Quiroz E, Martínez AA, González C, Castro del Campo N, et al. Complete Genome Sequence of Enterobacter Phage vB_EcRAM-01, a New Pseudotevenvirus against the Enterobacter cloacae Complex. Microbiology Resource Announcements. 10 May 2022: 1–3. doi:10.1128/mra.00045-22

19. CLSI. CLSI Performance Standards for Antimicrobial Susceptibility Testing, CLSI Guideline M100. 28th ed. Wayne, PA: Clinical and Laboratory Standards Institute; 2018.

20. Adams MH, Anderson ES, Kellenberger E. Bacteriophages. New York: Interscience Publishers; 1959.

21. Khan Mirzaei M, Nilsson AS. Isolation of Phages for Phage Therapy: A Comparison of Spot Tests and Efficiency of Plating Analyses for Determination of Host Range and Efficacy. Schuch R, editor. PLoS ONE. 2015;10: e0118557. doi:10.1371/journal.pone.0118557

22. Stephenson, Frank H. Calculations for Molecular Biology and Biotechnology. second edition. USA: Elsevier/AP, Academic Press is an imprint of Elsevier; 2010. Available: Vetbiotech.um.ac.ir

23. Laemmli UK. Cleavage of structural proteins during the assembly of the head of bacteriophage T4. Nature. 1970;227: 680–685. doi:10.1038/227680a0

24. Flores V. Caracterización de bacteriófagos líticos de Salmonella enterica aislados de muestras de pollos. Universidad Mayor San Marcos. 2017.

25. Zaburlin D, Quiberoni A, Mercanti D. Changes in Environmental Conditions Modify Infection Kinetics of Dairy Phages. Food Environ Virol. 2017;9: 270–276. doi:10.1007/s12560-017-9296-2

26. Bolger AM, Lohse M, Usadel B. Trimmomatic: a flexible trimmer for Illumina sequence data. Bioinformatics. 2014;30: 2114–2120. doi:10.1093/bioinformatics/btu170

27. Bankevich A, Nurk S, Antipov D, Gurevich AA, Dvorkin M, Kulikov AS, et al. SPAdes: A New Genome Assembly Algorithm and Its Applications to Single-Cell Sequencing. Journal of Computational Biology. 2012;19: 455–477. doi:10.1089/cmb.2012.0021

28. Langmead B, Trapnell C, Pop M, Salzberg SL. Ultrafast and memory-efficient alignment of short DNA sequences to the human genome. Genome Biology. 2009;10: R25. doi:10.1186/gb-2009-10-3-r25

29. Aziz RK, Bartels D, Best AA, DeJongh M, Disz T, Edwards RA, et al. The RAST Server: rapid annotations using subsystems technology. BMC Genomics. 2008;9: 75. doi:10.1186/1471-2164-9-75

30. Brettin T, Davis JJ, Disz T, Edwards RA, Gerdes S, Olsen GJ, et al. RASTtk: a modular and extensible implementation of the RAST algorithm for building custom annotation pipelines and annotating batches of genomes. Sci Rep. 2015;5: 8365. doi:10.1038/srep08365

31. Overbeek R, Olson R, Pusch GD, Olsen GJ, Davis JJ, Disz T, et al. The SEED and the Rapid Annotation of microbial genomes using Subsystems Technology (RAST). Nucleic Acids Res. 2014;42: D206-214.

doi:10.1093/nar/gkt1226

32. Besemer J, Lomsadze A, Borodovsky M. GeneMarkS: a self-training method for prediction of gene starts in microbial genomes. Implications for finding sequence motifs in regulatory regions. Nucleic Acids Research. 2001;29: 2607–2618. doi:10.1093/nar/29.12.2607

33. Delcher AL, Bratke KA, Powers EC, Salzberg SL. Identifying bacterial genes and endosymbiont DNA with Glimmer. Bioinformatics. 2007;23: 673–679. doi:10.1093/bioinformatics/btm009

34. Salzberg SL, Delcher AL, Kasif S, White O. Microbial gene identification using interpolated Markov models. Nucleic Acids Res. 1998;26: 544–548. doi:10.1093/nar/26.2.544

35. Altschul SF, Madden TL, Schäffer AA, Zhang J, Zhang Z, Miller W, et al. Gapped BLAST and PSI-BLAST: a new generation of protein database search programs. Nucleic Acids Res. 1997;25: 3389–3402.

36. Gabler F, Nam S-Z, Till S, Mirdita M, Steinegger M, Söding J, et al. Protein Sequence Analysis Using the MPI Bioinformatics Toolkit. Current Protocols in Bioinformatics. 2020;72: e108. doi:https://doi.org/10.1002/cpbi.108

37. Zimmermann L, Stephens A, Nam S-Z, Rau D, Kübler J, Lozajic M, et al. A Completely Reimplemented MPI Bioinformatics Toolkit with a New HHpred Server at its Core. Journal of Molecular Biology. 2018;430: 2237–2243. doi:10.1016/j.jmb.2017.12.007

38. Meier A, Söding J. Automatic Prediction of Protein 3D Structures by Probabilistic Multi-template Homology Modeling. PLOS Computational Biology. 2015;11: e1004343. doi:10.1371/journal.pcbi.1004343

39. Hildebrand A, Remmert M, Biegert A, Söding J. Fast and accurate automatic structure prediction with HHpred. Proteins: Structure, Function, and Bioinformatics. 2009;77: 128–132. doi:https://doi.org/10.1002/prot.22499

40. Potter SC, Luciani A, Eddy SR, Park Y, Lopez R, Finn RD. HMMER web server: 2018 update. Nucleic Acids Research. 2018;46: W200–W204. doi:10.1093/nar/gky448

41. Finn RD, Clements J, Arndt W, Miller BL, Wheeler TJ, Schreiber F, et al. HMMER web server: 2015 update. Nucleic Acids Research. 2015;43: W30–W38. doi:10.1093/nar/gkv397

42. Hofmann P, Tashman Z. Hidden Markov Models and Their Application for Predicting Failure Events. In: Krzhizhanovskaya VV, Závodszky G, Lees MH, Dongarra JJ, Sloot PMA, Brissos S, et al., editors. Computational Science – ICCS 2020. Cham: Springer International Publishing; 2020. pp. 464–477. doi:10.1007/978-3-030-50420-5_35

43. Laslett D, Canback B. ARAGORN, a program to detect tRNA genes and tmRNA genes in nucleotide sequences. Nucleic Acids Res. 2004;32: 11–16. doi:10.1093/nar/gkh152

44. Darling ACE, Mau B, Blattner FR, Perna NT. Mauve: Multiple Alignment of Conserved Genomic Sequence With Rearrangements. Genome Res. 2004;14: 1394–1403. doi:10.1101/gr.2289704

45. Meier-Kolthoff JP, Göker M. VICTOR: genome-based phylogeny and classification of prokaryotic viruses. Bioinformatics. 2017;33: 3396–3404. doi:10.1093/bioinformatics/btx440

46. Lefort V, Desper R, Gascuel O. FastME 2.0: A Comprehensive, Accurate, and Fast Distance-Based Phylogeny Inference Program. Mol Biol Evol. 2015;32: 2798–2800. doi:10.1093/molbev/msv150

47. Farris JS. Estimating Phylogenetic Trees from Distance Matrices. The American Naturalist. 1972;106:

645–668.

48. Letunic I, Bork P. Interactive Tree Of Life (iTOL) v4: recent updates and new developments. Nucleic Acids Research. 2019;47: W256–W259. doi:10.1093/nar/gkz239

49. Davis JJ, Wattam AR, Aziz RK, Brettin T, Butler R, Butler RM, et al. The PATRIC Bioinformatics Resource Center: expanding data and analysis capabilities. Nucleic Acids Res. 2020;48: D606–D612. doi:10.1093/nar/gkz943

50. Tynecki P, Guziński A, Kazimierczak J, Jadczuk M, Dastych J, Onisko A. PhageAI - Bacteriophage Life Cycle Recognition with Machine Learning and Natural Language Processing. 2020 [cited 11 Apr 2021]. doi:10.1101/2020.07.11.198606

51. Sullivan MJ, Petty NK, Beatson SA. Easyfig: a genome comparison visualizer. Bioinformatics. 2011;27: 1009–1010. doi:10.1093/bioinformatics/btr039

52. Moraru C, Varsani A, Kropinski AM. VIRIDIC—A Novel Tool to Calculate the Intergenomic Similarities of Prokaryote-Infecting Viruses. Viruses. 2020;12: 1268. doi:10.3390/v12111268

53. R Core Team. R: A Language and Environment for Statistical Computing. Vienna, Austria: R Foundation for Statistical Computing; 2023.

54. Oliveira H, Pinto G, Oliveira A, Oliveira C, Faustino MA, Briers Y, et al. Characterization and genome sequencing of a Citrobacter freundii phage CfP1 harboring a lysin active against multidrug-resistant isolates. Appl Microbiol Biotechnol. 2016;100: 10543–10553. doi:10.1007/s00253-016-7858-0

55. Kushkina AI, Tovkach FI, Comeau AM, Kostetskii IE, Lisovski I, Ostapchuk AM, et al. Complete Genome Sequence of Escherichia Phage Lw1, a New Member of the RB43 Group of Pseudo T-Even Bacteriophages. Genome Announcements. 2013;1: 10.1128/genomea.00743-13. doi:10.1128/genomea.00743-13

56. Kutter E, Sulakvelidze A, editors. Bacteriophages: biology and applications. Boca Raton, FL: CRC Press; 2005.

57. Al-Wrafy F, Brzozowska E, Górska S, Drab M, Strus M, Gamian A. Identification and characterization of phage protein and its activity against two strains of multidrug-resistant Pseudomonas aeruginosa. Sci Rep. 2019;9: 13487. doi:10.1038/s41598-019-50030-5

58. Moreno J. Antibiotic resistance profiles in Panama: Trends from 2007 to 2013. PPIJ. 2018;6. doi:10.15406/ppij.2018.06.00200

59. MINSA. 18400_2011_31657_INFORME RESISTENCIA ANTIBIOTICOS-USO RACIONAL -036.pdf. Panama: ICGES; 2015 p. 48.

60. Abedon ST. Detection of Bacteriophages: Phage Plaques. In: Harper DR, Abedon ST, Burrowes BH, McConville ML, editors. Bacteriophages. Cham: Springer International Publishing; 2018. pp. 1–32. doi:10.1007/978-3-319-40598-8_16-1

61. Hyman P, Abedon ST. Bacteriophage: Overview. Reference Module in Biomedical Sciences. Elsevier; 2019. p. B978012801238302506X. doi:10.1016/B978-0-12-801238-3.02506-X

62. Phothichaisri W, Ounjai P, Phetruen T, Janvilisri T, Khunrae P, Singhakaew S, et al. Characterization of Bacteriophages Infecting Clinical Isolates of Clostridium difficile. Front Microbiol. 2018;9. doi:10.3389/fmicb.2018.01701

63. Silva YJ, Costa L, Pereira C, Cunha Â, Calado R, Gomes NCM, et al. Influence of environmental variables in the efficiency of phage therapy in aquaculture. Microbial Biotechnology. 2014;7: 401–413. doi:10.1111/1751-7915.12090

64. Litt PK, Jaroni D. Isolation and Physiomorphological Characterization of Escherichia coli O157:H7-Infecting Bacteriophages Recovered from Beef Cattle Operations. In: International Journal of Microbiology [Internet]. 2017 [cited 9 Jul 2019]. doi:10.1155/2017/7013236

65. Yamaki S, Kawai Y, Yamazaki K. Characterization of a novel bacteriophage, Phda1, infecting the histamine-producing Photobacterium damselae subsp. damselae. Journal of Applied Microbiology. 2015;118: 1541–1550. doi:https://doi.org/10.1111/jam.12809

66. Asif M, Naseem H, Alvi IA, Basit A, Rehman S-. Characterization of a lytic EBP bacteriophage with large size genome against Enterobacter cloacae. APMIS. 2021;129: 461–469. doi:10.1111/apm.13138

67. Dąbrowska K. Phage therapy: What factors shape phage pharmacokinetics and bioavailability? Systematic and critical review. Med Res Rev. 2019;39: 2000–2025. doi:10.1002/med.21572

68. Khawaja KA, Abbas Z, Rehman S ur. Isolation and characterization of lytic phages TSE1-3 against Enterobacter cloacae. Open Life Sciences. 2016;11. doi:10.1515/biol-2016-0038

69. Finney AG, Perry JM, Evans DR, Westbrook KJ, McElheny CL, Iovleva A, et al. Isolation and Characterization of Lytic Bacteriophages Targeting Diverse Enterobacter spp. Clinical Isolates. PHAGE. 2022;3: 50–58. doi:10.1089/phage.2021.0011

70. Soohey RL. Identifyng Genes Essential For Lysogeny in Cluster e Mycobacteriophage Ukulele. The University of Maine. 2017.

71. Pollenz RS, Bland J, Pope WH. Bioinformatic characterization of endolysins and holin-like membrane proteins in the lysis cassette of phages that infect Gordonia rubripertincta. PLOS ONE. 2022;17: e0276603. doi:10.1371/journal.pone.0276603

72. Zhang M, Wang Y, Chen J, Hong X, Xu X, Wu Z, et al. Identification and Characterization of a New Type of Holin-Endolysin Lysis Cassette in Acidovorax oryzae Phage AP1. Viruses. 2022;14: 167. doi:10.3390/v14020167

73. Larpin Y, Oechslin F, Moreillon P, Resch G, Entenza JM, Mancini S. In vitro characterization of PlyE146, a novel phage lysin that targets Gram-negative bacteria. PLOS ONE. 2018;13: e0192507. doi:10.1371/journal.pone.0192507

74. Ríos-Sandoval M. Caracterización Genómica, Morfológica y Replicativa del Bacteriófago ΦXaF18 de Xanthomonas vesicatoria. Centro De Investigación Y Asistencia En Tecnología Y Diseño Del Estado De Jalisco, A. C. 2019.

75. Hatfull GF, Dedrick RM, Schooley RT. Phage Therapy for Antibiotic-Resistant Bacterial Infections. Annu Rev Med. 2022;73: 197–211. doi:10.1146/annurev-med-080219-122208

76. Hatfull GF. Bacteriophage genomics. Current Opinion in Microbiology. 2008;11: 447–453. doi:10.1016/j.mib.2008.09.004

77. Turner D, Kropinski AM, Adriaenssens EM. A Roadmap for Genome-Based Phage Taxonomy. Viruses. 2021;13: 506. doi:10.3390/v13030506

78. Adriaenssens EM, Sullivan MB, Knezevic P, van Zyl LJ, Sarkar BL, Dutilh BE, et al. Taxonomy of prokaryotic viruses: 2018-2019update from the ICTV Bacterial and Archaeal Viruses Subcommittee. Arch Virol. 2020;165: 1253–1260. doi:10.1007/s00705-020-04577-8

79. Adriaenssens E, Brister JR. How to Name and Classify Your Phage: An Informal Guide. Viruses. 2017;9: 70. doi:10.3390/v9040070

80. Leskinen K, Blasdel BG, Lavigne R, Skurnik M. RNA-Sequencing Reveals the Progression of Phage-Host Interactions between φR1-37 and Yersinia enterocolitica. Viruses. 2016;8: 111. doi:10.3390/v8040111

81. Jacquemot L, Bettarel Y, Monjol J, Corre E, Halary S, Desnues C, et al. Therapeutic Potential of a New Jumbo Phage That Infects Vibrio coralliilyticus, a Widespread Coral Pathogen. Front Microbiol. 2018;0. doi:10.3389/fmicb.2018.02501

82. Adams MJ, Lefkowitz EJ, King AMQ, Harrach B, Harrison RL, Knowles NJ, et al. 50 years of the International Committee on Taxonomy of Viruses: progress and prospects. Arch Virol. 2017;162: 1441–1446. doi:10.1007/s00705-016-3215-y

83. Turner D, Shkoporov AN, Lood C, Millard AD, Dutilh BE, Alfenas-Zerbini P, et al. Abolishment of morphology-based taxa and change to binomial species names: 2022 taxonomy update of the ICTV bacterial viruses subcommittee. Arch Virol. 2023;168: 74. doi:10.1007/s00705-022-05694-2

Supplementary figure legends

S1 Fig. Lytic effect of bacteriophage vB_EcRAM-01. According to the double agar plate method, the bacterial lawn of the *E. cloacae* complex is infected by bacteriophages. After purification, lysis PFU measuring approximately 9 mm (shown in the figure), rough-edged and completely clear, were obtained.

S2 Fig. Overall morphology of vB_EcRAM-01. A representative micrograph of a purified sample of the bacteriophage is shown (scale bar: 100nm).

S3 Fig. Electrophoretic profiles (SDS-PAGE) of phage EcRAM-01. M: Precision Plus protein ladder., S: phage sample. Ladder molecular weights are indicated on the left.

Response to the reviewers:

We appreciate your valuable feedback on our manuscript entitled **Characterization of Enterobacter phage vB_EcRAM-01, a new *Pseudotevenvirus* against *Enterobacter cloacae*, isolated in an urban river in Panama.** We are grateful for the opportunity to address your comments.

Reviewer #1: 1. In introduction section; the authors do not go into E. cloacae complex-infecting phages quite enough for this paper. Write more reports about phages specific for E. cloacae complex.
We thank the reviewer for pointing out this missing piece of information. A full paragraph has been included mentioning recent studies about phages specific for E. cloacae complex in the current version (lines 56-65)

2. Have you done bacterial reduction assay at different MOI?
We acknowledge the importance of investigating the impact of varying MOIs on phage-mediated bacterial reduction. Here we present an exploratory experiment and we have already begun planning subsequent experiments to analyze the dose-response relationship between MOI and bacterial clearance, particularly in combination with different antibiotics that we intend to address in a future paper.

3. In Methods: state the procedure of phage concentration.
The reviewer is right. A section describing the phage concentration procedure has been included in the Methods section in this new version (lines 94-101)
.
4. In Methods: Please add SDS-PAGE analysis protocol.
A section describing the SDS-PAGE analysis protocol has been included in the Methods section in this new version (lines 119-124).

5. In result section: you did not write about phage isolation.
Thank you for pointing this out. A paragraph describing phage isolation has been included in the results section (lines 184-188).
.
6. S2 Fig and S3 Fig are not clear. Please provide clear images with correct resolution.
We acknowledge that the resolution of the TEM image may not meet modern standards due to the limitations of the equipment used. However, we would like to emphasize that nowadays, the classification and identification of bacteriophages are increasingly reliant on genomic analysis rather than on morphological characteristics. Recent studies have demonstrated the importance of complete genome sequencing in the accurate classification of bacteriophages within specific genera. This approach has been widely adopted in the field and has led to a more precise and systematic classification of bacteriophages, overcoming the limitations associated with morphological variability and resolution in TEM images.
Regarding S3 Fig, we agree with the reviewer that the protein bands are quite faint. We know that the precipitation method used in this study doesn’t allow to obtain a

more concentrated phage sample. Unfortunately, we do not have access to an ultracentrifuge in Panama that would allow us to concentrate the viruses in order to get a better SDS-PAGE sample.

Reviewer #2: Please revise the representation of the values of the ordinate in Fig.2. And check carefully through out the manuscript.

The values of the ordinates in Figure 2 have been modified.

Once again, we appreciate your constructive feedback, which has strengthened the scientific rigor of our work. We are committed to addressing all concerns raised by the reviewers and ensuring the clarity and completeness of our manuscript.

Thank you for your consideration.
