# Supplementary material for: Characterization of Enterobacter phage vB_EcRAM-01, a new Pseudotevenvirus against Enterobacter cloacae, isolated in an urban river in Panama
Source: PLoS One. 2024 Dec 31;19(12):e0310824. doi: 10.1371/journal.pone.0310824 (PMC11687723; doi:10.1371/journal.pone.0310824)
Supplement: Attachment — Submitted filename: Rebuttal letter 280524.docx [file pone.0310824.s007.docx]

Response to the reviewers:

We appreciate your valuable feedback on our manuscript entitled **Characterization of Enterobacter phage vB_EcRAM-01, a new *Pseudotevenvirus* against *Enterobacter cloacae*, isolated in an urban river in Panama.** We are grateful for the opportunity to address your comments.

**Reviewer #1**: 1. In introduction section; the authors do not go into E. cloacae complex-infecting phages quite enough for this paper. Write more reports about phages specific for E. cloacae complex.

We thank the reviewer for pointing out this missing piece of information. A full paragraph has been included mentioning recent studies about phages specific for E. cloacae complex in the current version (lines 56-65)

2. Have you done bacterial reduction assay at different MOI?

We acknowledge the importance of investigating the impact of varying MOIs on phage-mediated bacterial reduction. Here we present an exploratory experiment and we have already begun planning subsequent experiments to analyze the dose-response relationship between MOI and bacterial clearance, particularly in combination with different antibiotics that we intend to address in a future paper.

3. In Methods: state the procedure of phage concentration.

The reviewer is right. A section describing the phage concentration procedure has been included in the Methods section in this new version (lines 94-101)

.
4. In Methods: Please add SDS-PAGE analysis protocol.

A section describing the SDS-PAGE analysis protocol has been included in the Methods section in this new version (lines 119-124).

5. In result section: you did not write about phage isolation.

Thank you for pointing this out. A paragraph describing phage isolation has been included in the results section (lines 184-188).

.
6. S2 Fig and S3 Fig are not clear. Please provide clear images with correct resolution.

We acknowledge that the resolution of the TEM image may not meet modern standards due to the limitations of the equipment used. However, we would like to emphasize that nowadays, the classification and identification of bacteriophages are increasingly reliant on genomic analysis rather than on morphological characteristics. Recent studies have demonstrated the importance of complete genome sequencing in the accurate classification of bacteriophages within specific genera. This approach has been widely adopted in the field and has led to a more precise and systematic classification of bacteriophages, overcoming the limitations associated with morphological variability and resolution in TEM images.

Regarding S3 Fig, we agree with the reviewer that the protein bands are quite faint. We know that the precipitation method used in this study doesn’t allow to obtain a more concentrated phage sample. Unfortunately, we do not have access to an ultracentrifuge in Panama that would allow us to concentrate the viruses in order to get a better SDS-PAGE sample.

**Reviewer #2**: Please revise the representation of the values of the ordinate in Fig.2. And check carefully through out the manuscript.

The values of the ordinates in Figure 2 have been modified.

Once again, we appreciate your constructive feedback, which has strengthened the scientific rigor of our work. We are committed to addressing all concerns raised by the reviewers and ensuring the clarity and completeness of our manuscript.

Thank you for your consideration.
