# Supplementary material for: Characterization of Enterobacter phage vB_EcRAM-01, a new Pseudotevenvirus against Enterobacter cloacae, isolated in an urban river in Panama
Source: PLoS One. 2024 Dec 31;19(12):e0310824. doi: 10.1371/journal.pone.0310824 (PMC11687723; doi:10.1371/journal.pone.0310824)
Supplement: Attachment — Submitted filename: Response to reviewers 030724.docx [file pone.0310824.s009.docx]

Panama, Panama, July 3, 2024

Response to the Editor and reviewers:

Please find below our response to the reviewers.

Reviewer #1:

1. Add the genomic organization of Enterobacter phage vB_EcRAM-01 in the Figure 5.

Thank you for your valuable feedback. The genome organization you requested to be added in Figure 5 is already shown in Figure 3, where it is compared with the phage genomes showing higher homology, and the locations of the major genes are indicated. We hope this addresses your concern.

2. How much microliters of concentrated phage solution were used in SDS-PAGE

Ten microliters of the concentrated phage sample were used.

3. Correct the plaque size in S1 Fig.

Plaque size has been corrected in the new version of S1 Fig.

Reviewer #2: The manuscript"Characterization of Enterobacter phage vB_EcRAM-01, a new Pseudotevenvirus against Enterobacter cloacae , isolated in an urban river in Panama." revealed a new phage, which should be a potential phage agent. Hope you can do more researches about it.

Thank you. We plan to isolated new phages against the Enterobacter cloacae complex and use them as cocktails in combination with antibiotics.
