# Supplementary material for: Characterization of Enterobacter phage vB_EcRAM-01, a new Pseudotevenvirus against Enterobacter cloacae, isolated in an urban river in Panama
Source: PLoS One. 2024 Dec 31;19(12):e0310824. doi: 10.1371/journal.pone.0310824 (PMC11687723; doi:10.1371/journal.pone.0310824)
Supplement: Attachment — Submitted filename: Response to reviewers 310724.docx [file pone.0310824.s010.docx]

Panama, Panama, July 31, 2024

Response to the Editor and reviewers:

Please find below our response to the reviewers.

Reviewer #1:

1. Add the genomic organization of Enterobacter phage vB_EcRAM-01 in the Figure 5.

The genome organization you requested has been added as Figure 4 and the rest of the figures have been renamed accordingly. Also, a separate table (sup. Table 1) with the predicted functions of ORFs derived from the phage genomic sequence has been included.
