# Supplementary material for: Characterization of Enterobacter phage vB_EcRAM-01, a new Pseudotevenvirus against Enterobacter cloacae, isolated in an urban river in Panama
Source: PLoS One. 2024 Dec 31;19(12):e0310824. doi: 10.1371/journal.pone.0310824 (PMC11687723; doi:10.1371/journal.pone.0310824)
Supplement: Attachment — Submitted filename: Response to reviewers 170824.docx [file pone.0310824.s011.docx]

Panama, Panama, August 17, 2024

Response to the Editor and reviewers:

Please find below our response to the reviewers.

Reviewer #1:

Reviewer #1: 1.In Fig .5, where is the name of the isolated phage? Please add the name of phage vB_EcRAM-01.

The name of phage vB_EcRAM-01 has been now highlighted in red for clarity instead of black.

2.Please arrange the Figures numbers according to the text of manuscript.

We thank the reviewer for noticing that. Figure numbers in the legends have been corrected in this new version of the manuscript

3.In S1 Table, add the numbers of ORFs and check the total numbers of it, add GenBank accession No. and start and stop of each ORF by nucleotide (nt).

Numbers of the ORFs as well as the GenBank accession numbers and start and stop codons for each of the ORFs are now included in S1 table.
